# Bioactive compounds from *Beilschmiedia percoriacea* leaves: isolation, structural characterization, and biological evaluations

**DOI:** 10.1039/d6ra04523e

**Published:** 2026-08-20

**Authors:** Hieu Tran-Trung, Dau Xuan Duc, Nguyen Thi Chung, Phan Quoc Hung, Hoang Van Trung, Nguyen Van Quoc, Hieu Nguyen-Ngoc, Cao Van Anh, Duc-Vinh Pham, Nguyen Xuan Ha, Le Duc Giang

**Affiliations:** a Department of Chemistry, Vinh University Nghean Vietnam leducgiang@gmail.com; b School of Chemistry, Biology and Environment, Vinh University Nghean Vietnam; c Faculty of Pharmacy, School of Medicine and Pharmacy, Phenikaa University Hanoi Vietnam; d Institute of Pharmaceutical Education, Vietnam Military Medical University Hanoi Vietnam; e Department of Pharmacology, Hanoi University of Pharmacy Hanoi Vietnam; f Institute of Chemistry, Vietnam Academy of Science and Technology Hanoi Vietnam

## Abstract

In this work, two previously undescribed sesquiterpenes, beilschmiedins A–B (1–2), as well as twelve known compounds (3–14), were obtained from the methanol extract of the *Beilschmiedia percoriacea* (*B. percoriacea*) leaves. Their structures were fully elucidated using a combination of spectroscopic techniques, including HR-ESI-MS, UV, and 1D/2D NMR, and compared with literature data. Furthermore, the absolute configurations of the new compounds 1 and 2 were determined by quantum chemical ECD calculations. Biologically, the fourteen isolates were evaluated for their anti-inflammatory, enzyme inhibitory, and antioxidant activities. Eleven compounds inhibited NO production more strongly than the positive control l-NAME. Among them, phillygenin (4), *epi*-pinoresinol (5), fargesin (6), and schisandlignan A (11) showed the most potent inhibitory effects (IC_50_ = 9.8–17.2 µM). In enzyme inhibition assays, schisandlignan A (11) and 3′,4′-dimethoxybenzoic acid (3″,4″-dimethoxyphenyl)-2-methyl-3-oxobutyl ester (14) exhibited exceptional α-glucosidase inhibitory potential (IC_50_ = 6.2 ± 0.38 and 5.0 ± 0.46 µM, respectively), significantly outperforming acarbose, while kobusin (7), forsythialan B (8), and nectandrin E (13) showed moderate xanthine oxidase inhibition (IC_50_ = 74.7–127.6 µM). For antioxidant activity, compound 5 possessed the strongest radical scavenging capacity, revealing that free phenolic hydroxyl groups enhanced potency. Furthermore, this study provides the first report of the anti-inflammatory activity of 11 and the α-glucosidase inhibition of 11 and 14, whereas the two new sesquiterpenes, 1 and 2, displayed only moderate anti-inflammatory effects and were inactive in the other assays. Molecular docking studies demonstrated that these bioactive compounds exhibit favorable binding affinities toward COX-2, XO, α-glucosidase, and Keap1, supporting their potential anti-inflammatory, antioxidant, antidiabetic, and anti-gout activities. The 200 ns molecular dynamics simulations further revealed stable complex behavior and limited structural deviation of the selected ligands within their respective binding sites.

## Abstract 2

Two new sesquiterpenes and twelve known isolates from *Beilschmiedia percoriacea* displayed potent anti-inflammatory, antidiabetic, and antioxidant activities.

## Graphical abstract

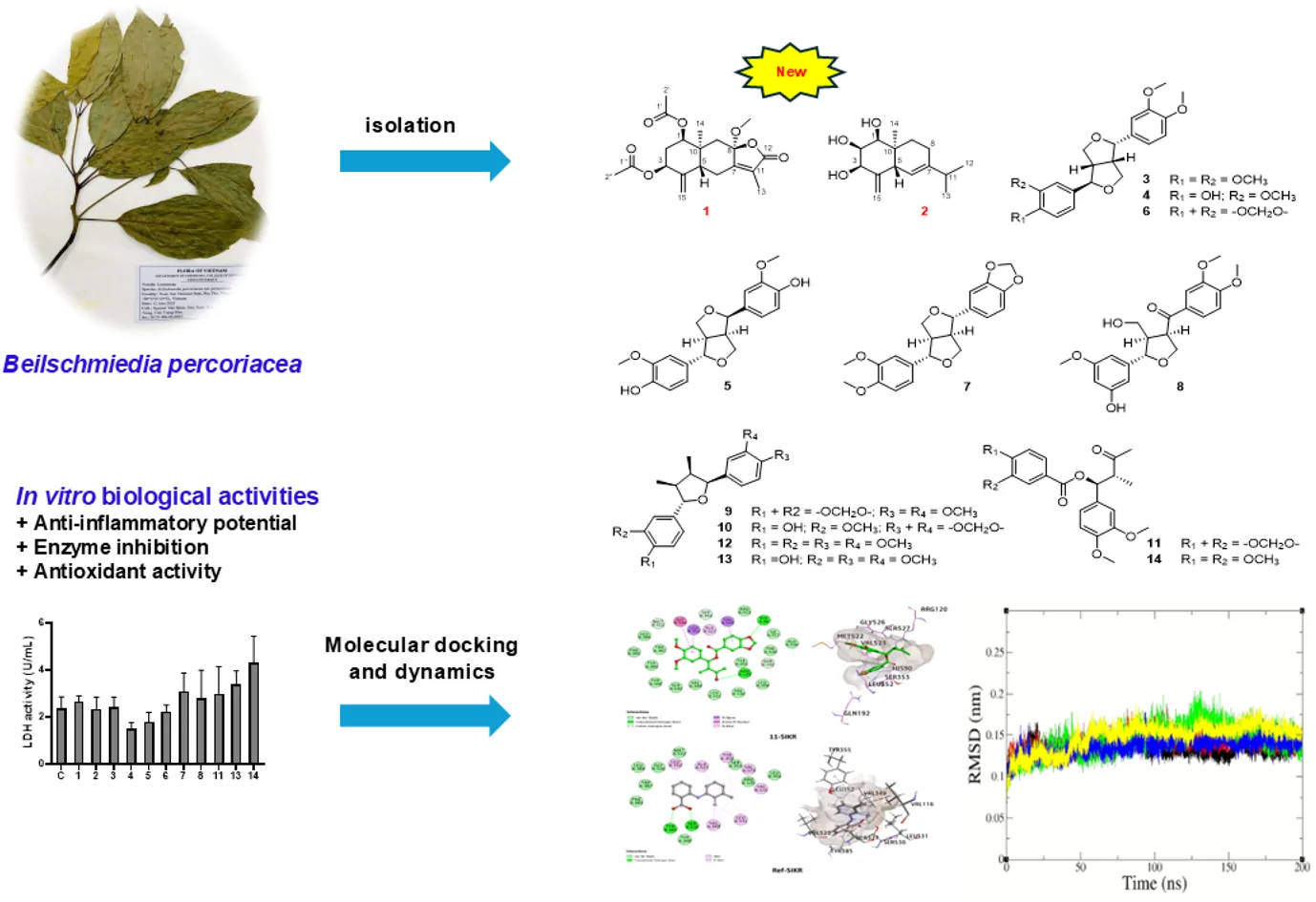

## 1 Introduction

The genus *Beilschmiedia*, belonging to the Lauraceae family, comprises approximately 250–300 species predominantly distributed throughout the tropical and subtropical regions of Southeast Asia and Africa.^1^ The timber from several *Beilschmiedia* species has been utilized in construction. Furthermore, certain species within the genus have been employed in traditional Asian and African medicine for the treatment of various ailments, including uterine tumors, rheumatism, pulmonary disorders, bacterial infections, malaria, and tuberculosis.^1^

A comprehensive review of the genus *Beilschmiedia* indicates that its main chemical constituents include terpenoids, lignans/neolignans, flavonoids, alkaloids, and endiandric acid derivatives.^1,2^ Overall, the phytochemical profiles of *Beilschmiedia* exhibit considerable diversity, varying significantly among different native species. Extracts and isolated compounds from *Beilschmiedia* species have demonstrated promising biological activities in various *in vitro* models, including cytotoxicity against cancer cell lines, antibacterial effects, and anti-inflammatory properties. For instance, beilschmiedic acids L, I, and K exhibited potent cytotoxic activity against the NCI-H460 lung carcinoma cell line, with IC_50_ values ranging from 4.4 to 5.9 µM.^3^ Beilschmiedic acid C displayed strong antibacterial activity against *M. luteus* (MIC = 5.58 µM) and *B. subtilis* (MIC = 11.1 µM).^3^ Furthermore, endiandramides A and B, along with endiandric acid M isolated from the roots of *B. tsangii*, showed potent inhibition of inducible nitric oxide synthase (iNOS), with IC_50_ values ranging from 16.4 to 31.7 µM.^4^

**Fig. 1:**
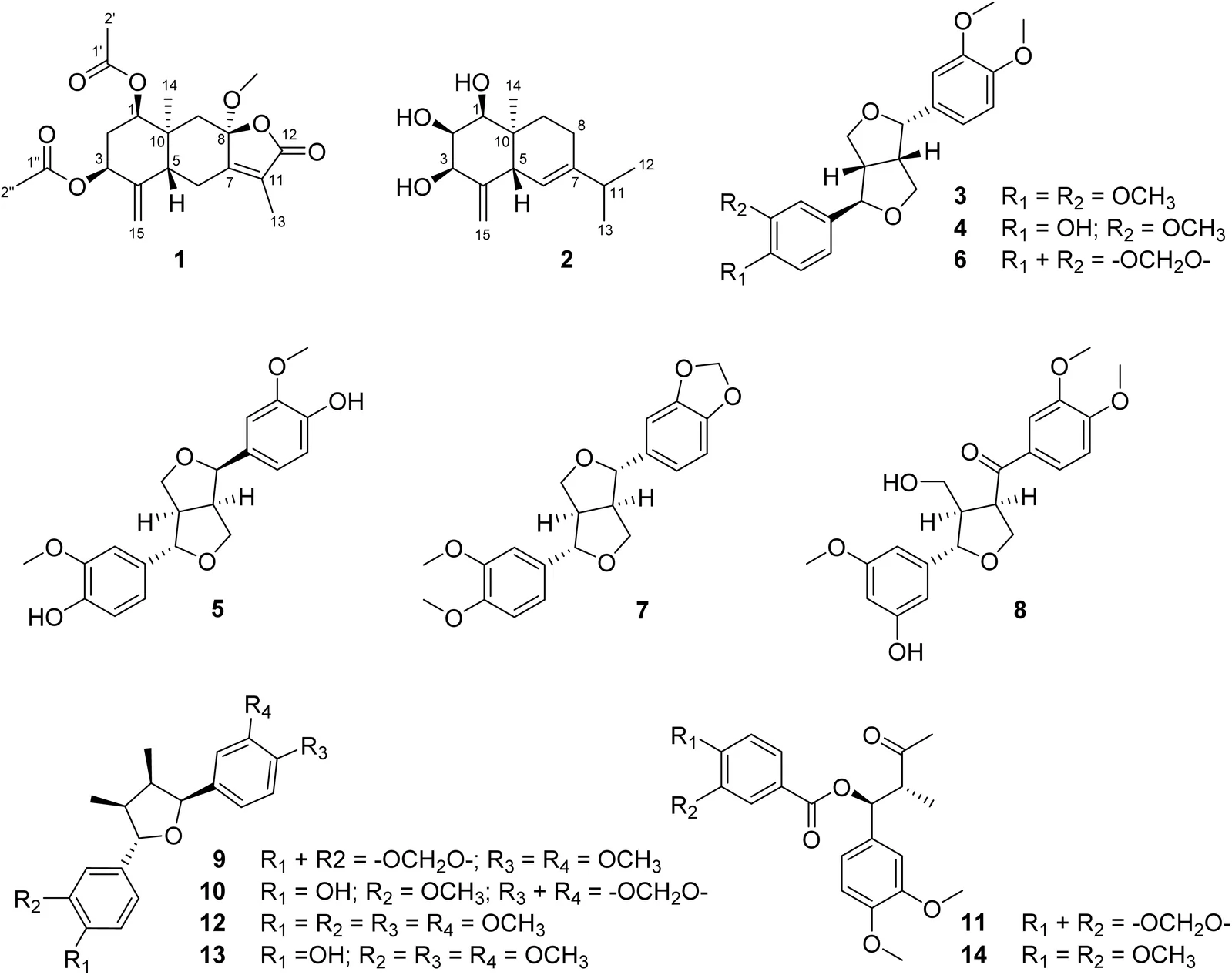
Structures of isolated compounds (1–14) from *B. percoriacea* leaves.

*B. percoriacea* (commonly known in Vietnam as “Chap dai”) is a large tree species reaching 15–18 m in height, with a trunk diameter of up to 1.5 m. It is mainly distributed in southern China, Vietnam, Thailand, and Malaysia.^5^ To date, no scientific studies have been conducted on the chemical composition or biological activities of this species. As part of our continuing investigation into potential natural products from newly studied Vietnamese medicinal herbs, *B. percoriacea* was subjected to a phytochemical and bioactivity evaluation study. These efforts afforded two novel sesquiterpenoids, beilschmiedins A–B (1–2), together with twelve known constituents comprising epieudesmin (3), phillygenin (4), *epi*-pinoresinol (5), fargesin (6), kobusin (7), forsythialan B (8), zuihonin C (9), (2*S*,3*S*,4*R*,5*S*)-talaumidin (10), schisandlignan A (11), ganschisandrine (12), nectandrin E (13), and 3′,4′-dimethoxybenzoic acid (3″,4″-dimethoxyphenyl)-2-methyl-3-oxobutyl ester (14) (Fig. 1). Additionally, this study represents the first evaluation of the biological activities of the isolated constituents from *B. percoriacea* leaves using a comprehensive battery of assays. These include DPPH, ABTS, and hydroxyl radical scavenging (*via* the CuSO_4_/ascorbate/3-CCA assay), nitric oxide production, lactate dehydrogenase (LDH) release, as well as the inhibition of xanthine oxidase and α-glucosidase. Furthermore, molecular docking and molecular dynamics (MD) simulations were performed to provide deeper insights into the inhibitory mechanisms and dynamic stability of the protein–ligand complexes.

## 2 Results and discussion

### 2.1 Structural data of isolated compounds

Compound 1 was isolated as a white amorphous powder. Its molecular formula was established as C_20_H_26_O_7_ by HR-ESI-MS, which exhibited a protonated molecular ion peak at *m*/*z* 379.17557 [M + H]^+^ (calcd for [C_20_H_27_O_7_]^+^, 379.17513), corresponding to eight degrees of unsaturation. In the UV spectrum of 1, maximum absorption was observed at 211.5 nm. The ^1^H NMR spectrum of 1 exhibited the characteristic proton signals of two oxymethine protons at *δ*_H_ (ppm) 5.41 (1H, dd, *J* = 4.2, 1.8 Hz, H-3) and 4.65 (1H, t, *J* = 3.0 Hz, H-1); two *exo*-methylene protons at *δ*_H_ 5.34 (1H, d, *J* = 1.8 Hz, H-15) and 4.98 (1H, d, *J* = 1.8 Hz, H-15); one methoxy group at *δ*_H_ 3.16 (3H, s, 8-OCH_3_); four methyl groups at *δ*_H_ 2.07 (3H, s, H-2′), 2.02 (3H, s, H-2″), 1.93 (3H, d, *J* = 1.8 Hz, H-13) and 1.04 (3H, s, H-14); one methine proton at *δ*_H_ 2.64 (1H, m, H-5); and three methylene groups at *δ*_H_ 2.70 (1H, dd, *J* = 13.2, 3.6 Hz, H-6a), 2.27 (1H, td, *J* = 13.2, 1.8 Hz, H-6b), 2.18 (1H, m, H-2a), 2.10 (1H, d, *J* = 13.8 Hz, H-9a), 2.09 (1H, m, H-2b), and 1.88 (1H, d, *J* = 13.8 Hz, H-9b). The ^13^C NMR and HSQC spectra of 1 indicated the presence of 20 carbon resonances including three ester carbonyls at *δ*_C_ (ppm) 171.5 (C-12), 170.2 (C-1′) and 169.8 (C-1″), three non-protonated sp^2^ carbons at *δ*_C_ 157.6 (C-7), 144.1 (C-4) and 124.9 (C-11), an exocyclic methylene at *δ*_C_ 114.6 (C-15), one dioxyquaternary carbon at *δ*_C_ 106.2 (C-8), two oxymethines at *δ*_C_ 74.7 (C-1) and 73.0 (C-3), as well as one methoxy group, three methyls, three methylenes, and an aliphatic quaternary carbon. Based on the combination of these data and comparisons with compounds previously isolated from the family Lauraceae, compound 1 was identified as an eudesmane-type sesquiterpene lactone.^6^

**Fig. 2:**
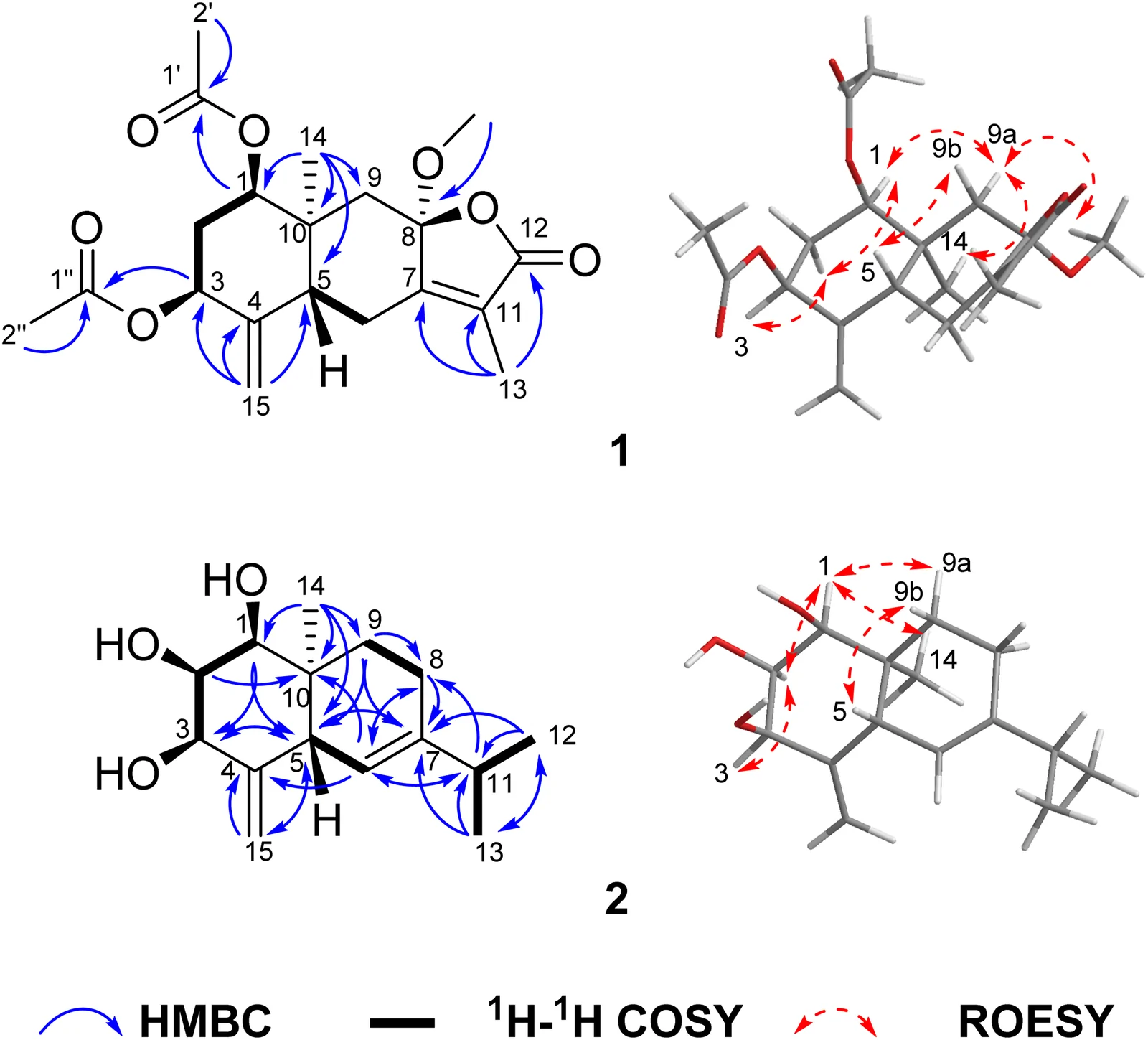
Key 2D (COSY, HMBC, and ROESY) correlations of 1 and 2.

**Fig. 3:**
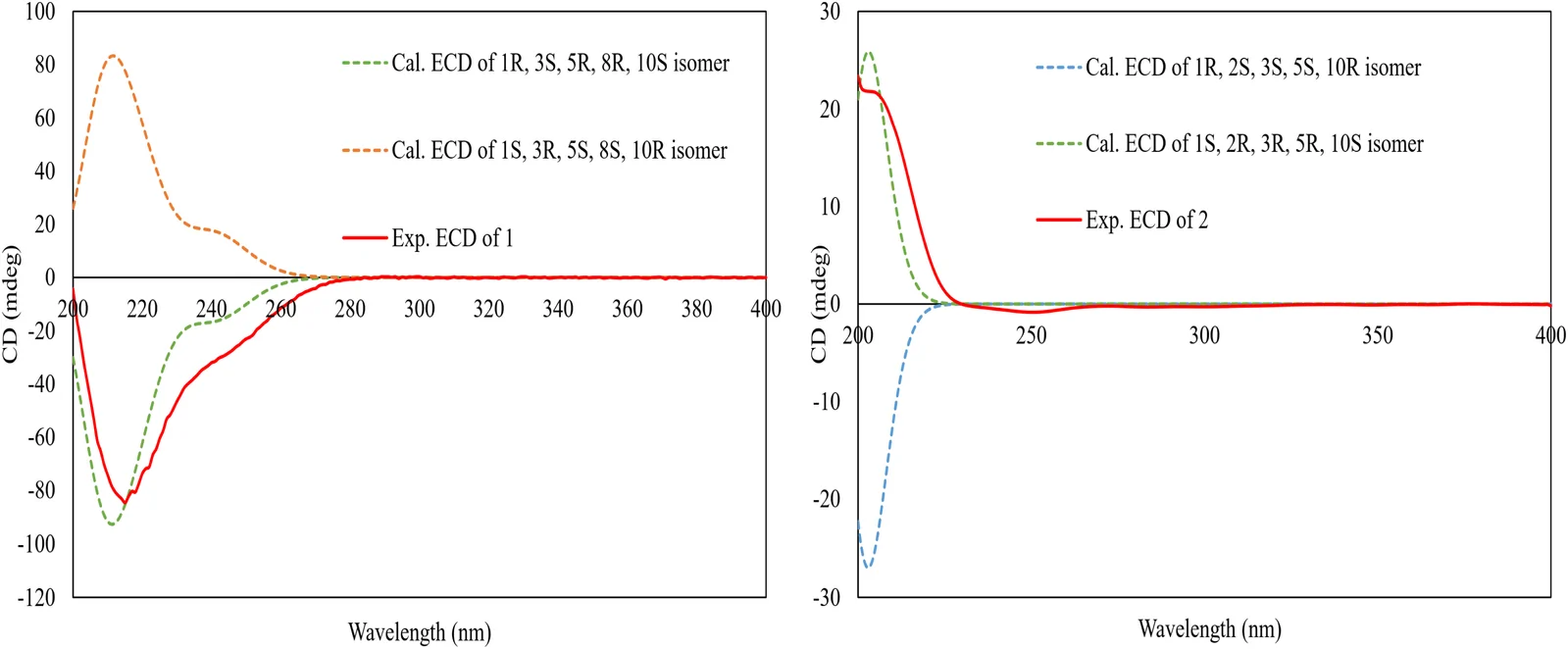
Calculated and experimental ECD spectra of 1 and 2 in MeOH.

Comprehensive analysis of its HMBC correlations further revealed the presence of a methoxy, an *exo*-methylene, and two acetoxy groups attached to this skeleton (as shown in Fig. 2). The positions of these functional groups were confirmed based on key cross-peaks in the HMBC spectrum. Specifically, the HMBC correlation from the proton signal at *δ*_H_ 3.16 to C-8 (*δ*_C_ 106.2) revealed the location of the methoxy group at C-8. The two acetoxy groups were assigned to C-1 and C-3, respectively, evidenced by the cross-peaks from H-1/H-2′ to C-1′, and from H-3/H-2″ to C-1″. The position of the *exo*-methylene at C-15 was established based on the HMBC correlations from H_2_-15 to C-3/C-4/C-5. Furthermore, the locations of the two methyl groups linked to C-10 and C-11 were also confirmed by the long-range correlations from H_3_-14 to C-1/C-5/C-9/C-10 and from H_3_-13 to C-7/C-11/C-12. Consequently, the planar structure of 1 was established based on a combination of 1D (^1^H and ^13^C NMR) and 2D (^1^H–^1^H COSY, HSQC, and HMBC) NMR spectra. The relative configuration of 1 was elucidated through ROESY correlations, ^1^H–^1^H coupling constants (*J*), as well as ECD data, showing close similarity to plebeiolide A, a sesquiterpene isolated from *Salvia plebeia* whose structure was confirmed by X-ray analysis.^7^ In the ROESY spectrum (Fig. 2), the correlations between H-1/H_3_-14/H-9a/8-OCH_3_ and H-5/H-9b, coupled with the absence of an H-14/H-5 interaction, confirmed that H-5 and H-9b are co-facial and assigned as β-oriented, while H_3_-14 and H-1 were determined to be α-oriented. Furthermore, the half-chair conformation of the cyclohexene ring, along with the small coupling constants (dd, *J* = 4.2, 1.8 Hz) between H-3 and vicinal methylene protons, indicated that H-3 is equatorial, which corresponds to an axially β-oriented 3-acetoxy group.^8^ The negative Cotton effects observed at 218 and 241 nm in the CD spectrum substantiated the *R* configuration at C-8, consistent with previously reported compounds.^9,10^ Furthermore, the absolute configuration of 1 was determined by comparing its experimental ECD spectrum with calculated data (Fig. 3). The results showed excellent agreement with the calculated ECD spectrum of (1*R*,3*S*,5*R*,8*R*,10*S*)-1. Accordingly, compound 1 was determined as a new eudesmanolide and named beilschmiedin A.

Compound 2, obtained as a white amorphous powder, was assigned the molecular formula C_15_H_24_O_3_ based on HR-ESI-MS data, which exhibited a sodium-adduct ion peak at *m*/*z* 275.16207 [M + Na]^+^ (calcd for [C_15_H_24_O_3_Na]^+^, 275.16177) with corresponding to four degrees of unsaturation. In the UV spectrum of 2, maximum absorptions were observed at 212.0, 245.0, and 296.0 nm. The ^1^H NMR spectrum of 2 displayed the signals for three olefinic protons at *δ*_H_ (ppm) 5.44 (1H, br s, H-6), 5.14 (1H, br s, H-15a) and 4.87 (1H, br s, H-15b); three oxymethine protons at *δ*_H_ 4.34 (1H, d, *J* = 3.0 Hz, H-3), 3.84 (1H, dd, *J* = 3.0, 1.8 Hz, H-2), and 3.56 (1H, d, *J* = 1.8 Hz, H-1); three methyl groups at *δ*_H_ 1.04 (3H, d, *J* = 7.2 Hz, H-12), 1.03 (3H, d, *J* = 7.2 Hz, H-13), and 0.61 (3H, s, H-14); two methine proton at *δ*_H_ 3.29 (1H, br s, H-5) and 2.24 (1H, m, H-11); and two methylene groups at *δ*_H_ 2.10–1.30 ppm. The ^13^C NMR and HSQC spectra of 2 revealed 15 carbon signals, including four olefinic carbons at *δ*_C_ (ppm) 147.7 (C-4), 144.0 (C-7), 116.8 (C-6), and 111.4 (C-15); three oxymethines at *δ*_C_ 79.4 (C-1), 78.3 (C-3), and 69.4 (C-2); three methyls at *δ*_C_ 21.8 (C-13), 21.5 (C-12), and 15.4 (C-14); two methylenes at *δ*_C_ 31.0 (C-9) and 22.9 (C-8); two methines at *δ*_C_ 35.0 (C-5) and 34.9 (C-11), along with one quaternary sp^3^ carbon at *δ*_C_ 38.0 (C-10). The ^1^H–^1^H COSY correlations of H-1/H-2/H-3, together with the HMBC correlations from H-1 to C-2/C-3/C-5, H-2 to C-10, and H-3 to C-1/C-4/C-5, confirmed the placement of the hydroxyl groups at C-1, C-2, and C-3 (Fig. 2). The HMBC correlations from H_3_-14 to C-1/C-5/C-9/C-10, H_3_-12 to C-7/C-11/C-13, and H_3_-13 to C-7/C-11/C-12 indicated that one methyl group (C-14) was attached to C-10, while two methyl groups (C-12 and C-13) were linked to C-11. Additionally, the ^1^H–^1^H COSY correlation of H-5/H-6, together with the HMBC cross-peaks from the exomethylene protons (H_2_-15) to C-3/C-4/C-5 and from H-6 to C-4/C-8/C-10/C-11, confirmed the positions of the two double bonds at C-4(15) and C-6(7). Detailed analysis of the spectral data above suggested that 2 was an eudesmane derivative, and its planar structure was established as shown in Fig. 2. The relative configuration of 2 was determined through a detailed analysis of ^1^H–^1^H coupling constants and ROESY correlations. Specifically, the small coupling constants observed for H-1 (*J* = 1.8 Hz) and H-3 (*J* = 3.0 Hz) indicated a close structural analogy with 1 regarding their specific spatial orientations. Accordingly, both H-1 and H-3 were assigned an α-orientation. Moreover, the observed mutual ROESY correlations among H-1, H-2, H-3, and H_3_-14 indicated these protons to be on the same face of the structure. Moreover, the observed mutual ROESY correlations among H-1, H-2, H-3, and H_3_-14 indicated that these protons in 2 are on the same side of the structure. Conversely, the absence of ROESY correlations between these protons and H-5 suggested that H-5 occupies the opposite face. Consequently, H-1, H-2, H-3, and H_3_-14 were assigned to the α-oriented, while H-5 was designated as β-oriented. The absolute configuration of 2 was achieved by comparing the calculated ECD data with experimental data, which showed good agreement with the (1*S*,2*R*,3*R*,5*R*,10*S*)-2 possibility (Fig. 3). Accordingly, the structure of compound 2 was elucidated as shown and was named beilschmiedin B (Table 1).

**Table 1:** ^1^H NMR and ^13^C NMR data of compounds 1 and 2

Position	1^a^		2^a^	
	*δ*_C_ (type)	*δ*_H_ (*J* in Hz)	*δ*_C_ (type)	*δ*_H_ (*J* in Hz)
1	74.7 (CH)	4.65, t (3.0)	79.4 (CH)	3.56, d (1.8)
2	30.2 (CH_2_)	2.18, m	69.4 (CH)	3.84, dd (3.0, 1.8)
		2.09, m		
3	73.0 (CH)	5.41, dd (4.2, 1.8)	78.3 (CH)	4.34, d (3.0)
4	144.1 (C)	—	147.7 (C)	—
5	40.8 (CH)	2.64, m	35.0 (CH)	3.29, br s
6	23.8 (CH_2_)	2.70, dd (13.2, 3.6)	116.8 (CH)	5.44, br s
		2.27, td (13.2, 1.8)		
7	157.6 (C)	—	144.0 (C)	—
8	106.2 (C)	—	22.9 (CH_2_)	2.08–2.05, m^b^
9	42.6 (CH_2_)	2.10, d (13.8)	31.0 (CH_2_)	2.08–2.05, m^b^
		1.88, d (13.8)		1.38, m
10	39.7 (C)	—	38.0 (C)	—
11	124.9 (C)	—	34.9 (CH)	2.24, m
12	171.5 (C)	—	21.5 (CH_3_)	1.04, d (7.2)
13	8.5 (CH_3_)	1.93, d (1.8)	21.8 (CH_3_)	1.03, d (7.2)
14	16.5 (CH_3_)	1.04, s	15.4 (CH_3_)	0.61, s
15	114.6 (CH_2_)	5.34, d (1.8)	111.4 (CH_2_)	5.14, br s
		4.98, d (1.8)		4.87, br s
1′	170.2 (C)	—		
2′	21.3 (CH_3_)	2.07, s		
1″	169.8 (C)	—		
2″	21.4 (CH_3_)	2.02, s		
8-OCH_3_	50.2 (CH_3_)	3.16, s		

a ^1^H NMR (600 MHz) and ^13^C NMR (150 MHz) measured in CDCl_3_.

b Overlapped.

In addition, twelve known compounds were identified for the first time from *B. percoriacea* as epieudesmin (3),^11^ phillygenin (4),^12^*epi*-pinoresinol (5),^13^ fargesin (6),^12^ kobusin (7),^14^ forsythialan B (8),^15^ zuihonin C (9),^16,17^ (2*S*,3*S*,4*R*,5*S*)-talaumidin (10),^18^ schisandlignan A (11),^19^ ganschisandrine (12),^20^ nectandrin E (13),^21^ and 3′,4′-dimethoxybenzoic acid (3″,4″-dimethoxyphenyl)-2-methyl-3-oxobutyl ester (14).^19^ Their structures were confirmed by comparison their spectroscopic data with literature reports.

### 2.2 *In vitro* biological activities

Anti-inflammatory activities of the fourteen isolates were evaluated in lipopolysaccharide (LPS)-stimulated RAW 264.7 macrophages using a nitric oxide (NO) release assay. With the exception of 9 and 10, for which IC_50_ values could not be determined because their inhibitory effects did not exceed 50% at the highest non-cytotoxic concentrations tested, and 12, which showed weak activity (IC_50_ > 100 µM), the remaining isolates inhibited NO production more strongly than the positive control, l-NAME (Table 2). Among them, 4, 5, 6, and 11 were the most potent NO inhibitors, with IC_50_ values of 9.8, 11.9, 17.2, and 10.2 µM, respectively. Notably, 4, 5, and 6 are structurally related lignans, and previous studies have shown that these lignans suppress inflammatory responses in macrophages at concentrations ranging from 5 to 25 µM,^22–24^ in agreement with the present results. In contrast, to the best of our literature search, anti-inflammatory activity of 11 in macrophages has not been clearly documented. The two new sesquiterpenoids, 1 and 2, displayed moderate anti-inflammatory activity, with IC_50_ values of 78.4 and 55.3 µM, respectively. To further exclude cytotoxicity-mediated NO reduction, concentration-dependent cell viability was evaluated up to 300 µM, the highest concentration compatible with compound solubility and/or acceptable vehicle levels. CC_50_ values and selectivity indices (SI = CC_50_/IC_50_ for NO inhibition) were calculated and are provided in Table S1. Compounds 4, 5, and 6 showed favourable selectivity, with SI values of >30.61, >25.21, and 17.05, respectively. In addition, LDH release was assessed at the corresponding NO IC_50_ concentrations of active compounds. No marked LDH release was observed for all active compounds compared with the control, supporting that their NO inhibitory effects were not primarily attributable to cytotoxicity (Fig. S85, SI).

**Table 2:** NO production inhibitory activity of isolated compounds from *B. percoriacea* in RAW 264.7 cells^a^

Compounds	NO inhibition (IC_50_, µM)
1	78.4 ± 16.05
2	55.3 ± 0.39
3	42.8 ± 1.88
4	9.8 ± 0.13
5	11.9 ± 1.38
6	17.2 ± 3.77
7	69.0 ± 3.61
8	59.2 ± 6.08
9	N.D
10	N.D
11	10.2 ± 0.27
12	>100
13	29.6 ± 2.03
14	22.9 ± 2.96
Positive control (l-NAME)	113.7 ± 12.00

a Data are expressed as mean ± SE (*n* = 3). N.D: not be determined.

Following that, the effects of the isolates on the enzymatic activities of xanthine oxidase (XO) and α-glucosidase, which are two important molecular targets associated with metabolic disorders such as hyperuricemia and diabetes, respectively, were also evaluated. As shown in Table 3, compounds 7, 8, and 13 exhibited moderate XO inhibitory activity, whereas 9 showed only weak inhibition. In contrast, 10, 11, 13, and 14 effectively inhibited α-glucosidase. Respectively, compounds 11 and 14 displayed particularly potent activity, with IC_50_ values of 6.2 and 5.0 µM, respectively. Notably, these values were markedly lower than that of the positive control acarbose, indicating strong α-glucosidase inhibitory potential. Based on our literature search, direct XO-inhibitory data for 7, 8, 9, and 13 remain limited, and the present results therefore provide additional information on the XO-related bioactivity of these isolates. To the best of our literature search, this is the first report of the α-glucosidase inhibitory activities of 11 and 14. In contrast, two new sesquiterpenoids, 1 and 2, did not exhibit significant inhibitory effects against either XO or α-glucosidase (IC_50_ > 200 µM).

**Table 3:** Inhibitory activity of isolated compounds from *B. percoriacea* against XO and α-glucosidase

Compounds	XO inhibition (IC_50_, µM)	α-Glucosidase inhibition (IC_50_, µM)
1	>200	>200
2	>200	>200
3	>200	>200
4	>200	>200
5	>200	>200
6	>200	>200
7	74.7 ± 0.26	>200
8	99.4 ± 1.93	>200
9	172.6 ± 20.83	>200
10	>200	14.5 ± 3.04
11	>200	6.2 ± 0.38
12	>200	>200
13	127.6 ± 11.02	30.1 ± 3.55
14	>200	5.0 ± 0.46
Positive control^a^	27.4 ± 0.97	1720 ± 200.0

a Positive control used were quercetin and acarbose for XO and α-glucosidase inhibition assays, respectively. Data are expressed as mean ± SE (*n* = 3).

Finally, the antioxidant activities of the isolates, which are thought to be involved in the pathogenesis of inflammatory and metabolic disorders,^25^ were evaluated using ABTS, DPPH, and hydroxyl radical scavenging assays. As shown in Table 4, 5 showed the strongest ABTS radical scavenging activity, followed by 4, 8, 13, and 10. 11 was only weakly active, whereas the remaining isolates were inactive (IC_50_ > 200 µM). In the DPPH assay, the overall activity was weaker. 5 exhibited the most potent activity, comparable to that of Trolox, followed by 4 and 8, while 10 showed only weak activity. The other compounds were inactive in this assay. In the hydroxyl radical scavenging assay using the CuSO_4_/ascorbate/3-CCA system, compound 5 was again the most active compound, followed by 4, 10, and 8. The remaining isolates showed no detectable activity at the highest tested concentration.

**Table 4:** Antioxidant activity of isolated compounds from *B. percoriacea*

Compounds	Free radical scavenging activity (IC_50_, µM)		
	ABTS	DPPH	Hydroxyl^a^
1	>200	>200	>200
2	>200	>200	>200
3	>200	>200	>200
4	17.2 ± 0.16	69.9 ± 13.02	63.8 ± 1.83
5	13.0 ± 0.62	35.6 ± 1.61	23.01 ± 2.71
6	>200	>200	>200
7	>200	>200	>200
8	21.4 ± 1.07	83.3 ± 6.39	107.2 ± 9.2
9	>200	>200	>200
10	31.6 ± 0.54	179.0 ± 23.65	106.85 ± 16.44
11	193.6 ± 11.07	>200	>200
12	>200	>200	>200
13	29.5 ± 1.15	>200	>200
14	>200	>200	>200
Trolox	20.0 ± 0.78	35.9 ± 2.92	98.29 ± 9.61

a Hydroxyl radicals were generated by the CuSO_4_/ascorbate system and detected using 3-coumarin carboxylic acid (3-CCA). Data are expressed as mean ± SE (*n* = 3).

Based on the biological evaluation results, a detailed analysis of the structure–activity relationships (SAR) of the fourteen isolated compounds (1–14) provides a better understanding of how their main functional groups and skeletons affect their biological activities. Among the furofuran lignans (3–8), the presence of free phenolic hydroxy groups plays a key role in both antioxidant and anti-inflammatory activities. Compounds with free hydroxy groups, such as phillygenin (4) and epi-pinoresinol (5), showed the strongest radical scavenging activity and potent NO inhibition (IC_50_ = 9.8 and 11.9 µM, respectively). In contrast, when these hydroxy groups were methylated, as in epieudesmin (3) and fargesin (6), the antioxidant activity was completely lost (IC_50_ > 200 µM). This proves that phenolic hydroxy groups are necessary for their antioxidant capacity. Interestingly, compound 6 still showed strong anti-inflammatory activity (IC_50_ = 17.2 µM), suggesting that its anti-inflammatory mechanism does not depend on direct radical scavenging. Furthermore, while all furofuran lignans (3–8) failed to inhibit α-glucosidase (IC_50_ > 200 µM) due to their rigid structures, compounds with a methylenedioxy group (7) selectively inhibited xanthine oxidase (IC_50_ = 74.7 µM).

On the other hand, flexible open-chain compounds showed strong α-glucosidase inhibition. Compounds 14 (IC_50_ = 5.0 µM) and 11 (IC_50_ = 6.2 µM) were much more active than acarbose. Molecular docking results confirmed that their flexible structures allow them to fit well into the active site of α-glucosidase to form key interactions with Arg526 and Trp406.

In general, the 7,7′-epoxylignans (2,5-diaryl-tetrahydrofuran lignan, 9, 10, 12, and 13) showed weak or insignificant activity in most tests. Their biological activities depend significantly on their functional groups, especially free phenolic hydroxy groups. In compound 12, all hydroxy groups are replaced by methoxy groups, which results in a complete loss of activity in all assays. Similarly, compound 9 lacks free hydroxy groups, so it only shows weak activity against xanthine oxidase due to its methylenedioxy group. On the other hand, compounds 10 and 13 contain free hydroxy groups, allowing them to show strong α-glucosidase inhibition, with compound 13 inhibiting both target enzymes. In summary, free phenolic hydroxyl groups are essential for the biological activity of 7,7′-epoxylignans, while converting them to methoxy groups removes their activity.

Regarding the two new sesquiterpenes (1 and 2), both were inactive in antioxidant, α-glucosidase, and xanthine oxidase assays. However, both showed moderate anti-inflammatory activity, where compound 2 (IC_50_ = 55.3 µM) was more active than compound 1 (IC_50_ = 78.4 µM). The lower activity of 1 can be explained by the presence of large acetoxy groups and a rigid lactone ring, which create steric hindrance and reduce binding affinity.

In conclusion, the structure–activity relationship (SAR) analysis shows that biological activities depend greatly on structural flexibility and functional groups. Free phenolic hydroxy groups are essential for antioxidant and anti-inflammatory activities, whereas replacing them with methoxy groups leads to a reduction or complete loss of activity. In addition, flexible open-chain structures allow better inhibition of α-glucosidase compared to rigid skeletons. However, further studies are essential to provide a better explanation for these findings.

### 2.3 Molecular docking and molecular dynamics (MD) studies

**Fig. 4:**
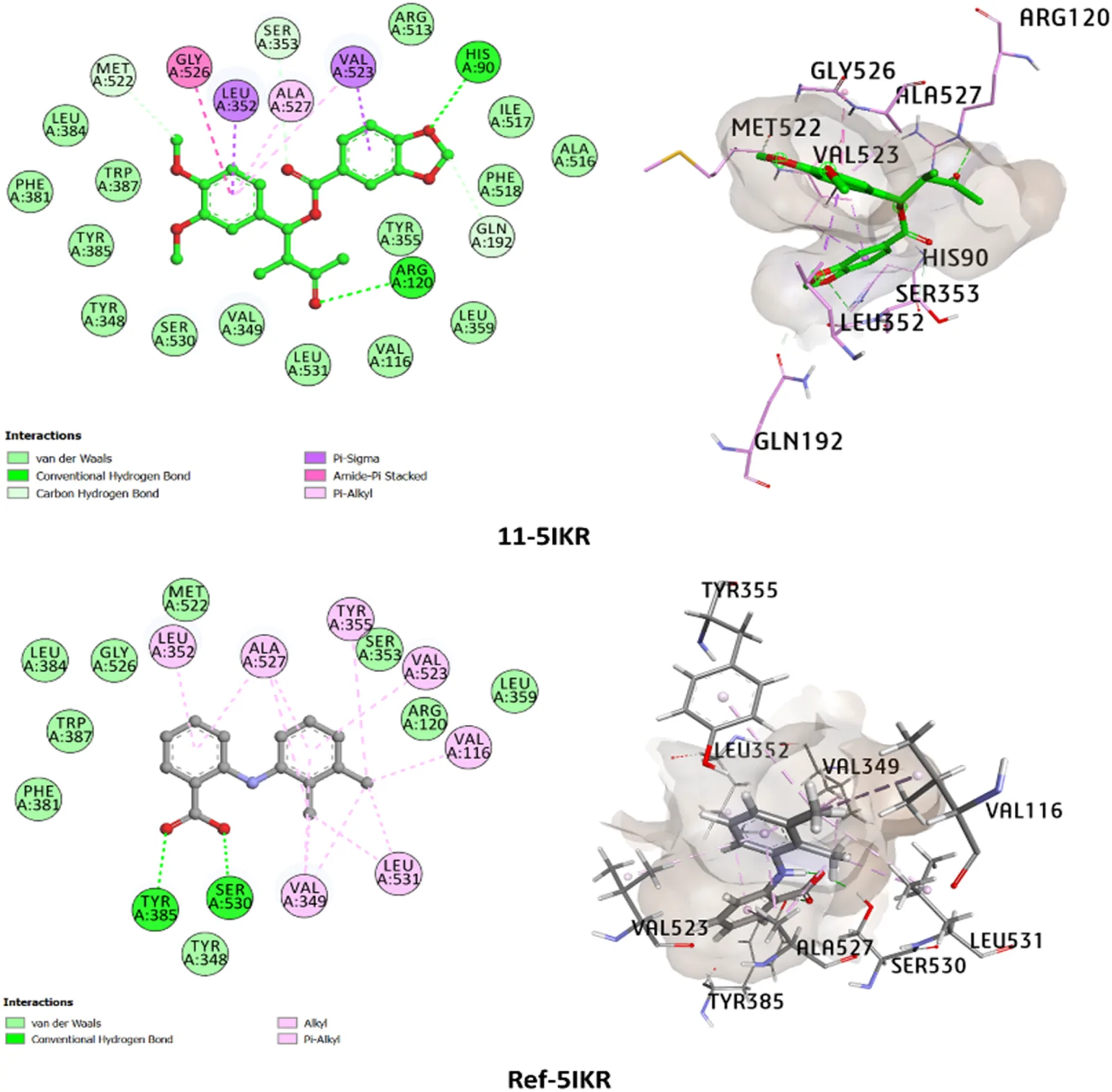
2D and 3D interaction profiles of selected potential compounds bound to the active site of the COX-2 protein (PDB ID: 5IKR).

**Fig. 5:**
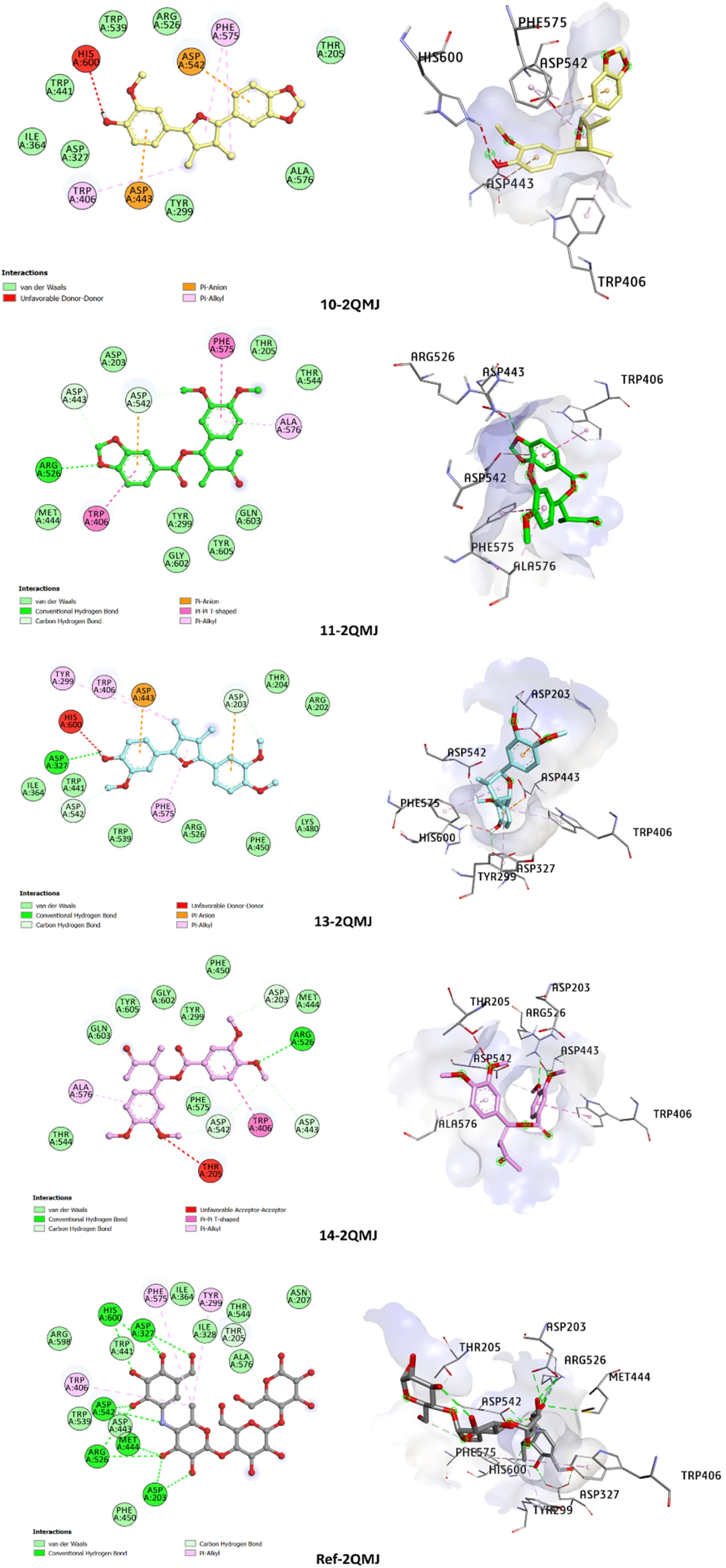
2D and 3D interaction profiles of selected potential compounds bound to the active site of the α-glucosidase protein (PDB ID: 2QMJ).

**Fig. 6:**
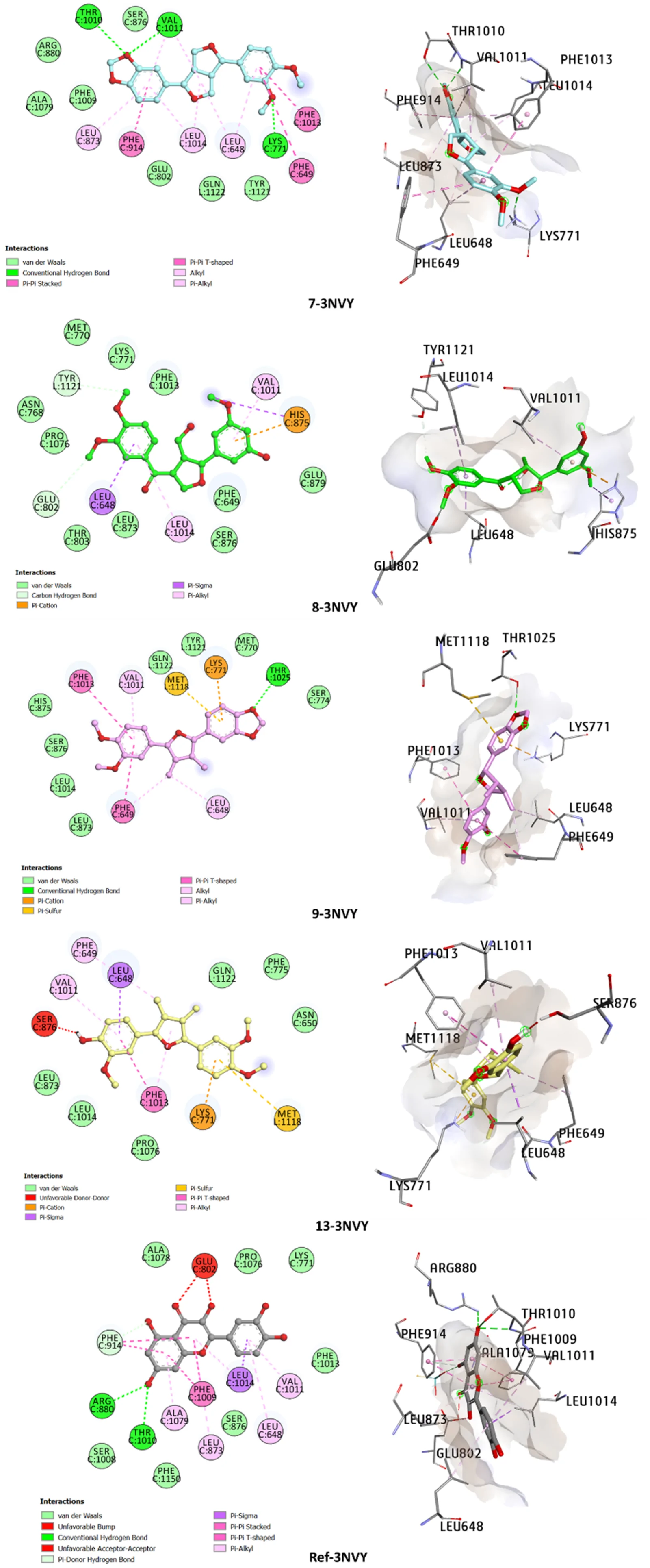
2D and 3D interaction profiles of selected potential compounds bound to the active site of the XO protein (PDB ID: 3NVY).

**Fig. 7:**
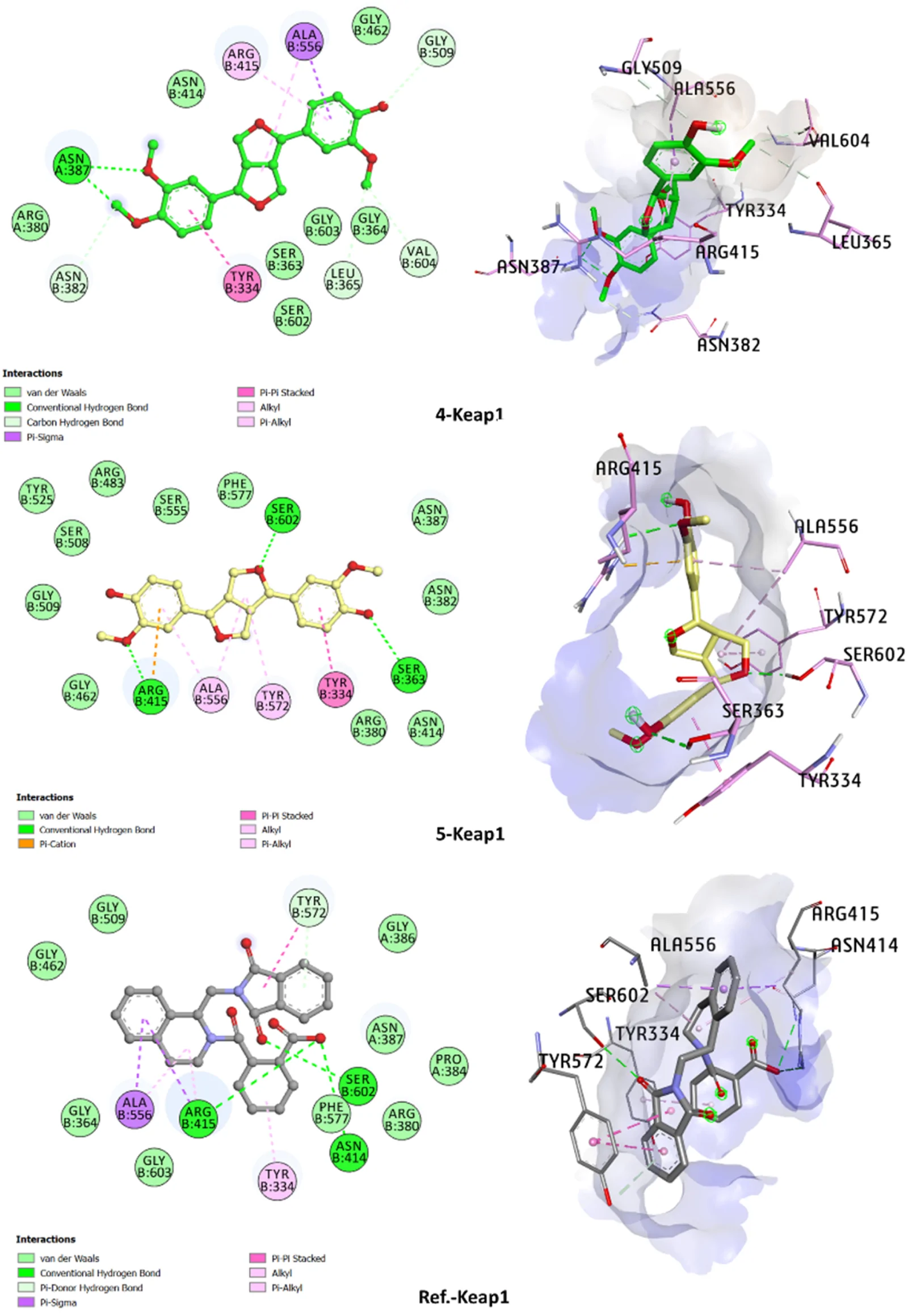
2D and 3D interaction profiles of selected potential compounds bound to the active site of the Keap1 protein (PDB ID: 4L7B).

Based on the *in vitro* assay results, the isolated compounds from *B. percoriacea* that exhibited inhibitory effects on NO production, XO, α-glucosidase, and antioxidant activity were subjected to *in silico* molecular docking studies. This was done to predict their interactions with key biological targets, specifically cyclooxygenase-2 (COX-2), XO, α-glucosidase, and Keap1. The COX-2 is a key enzyme involved in the inflammatory pathway and is responsible for the production of prostaglandins, making it an important target for anti-inflammatory agents.^26^ The XO plays a crucial role in purine metabolism and is associated with the generation of uric acid and reactive oxygen species, thus being a target for anti-gout and antioxidant therapies.^27^ α-Glucosidase is an essential enzyme in carbohydrate digestion that catalyzes the breakdown of oligosaccharides into glucose, and its inhibition is widely used in the management of postprandial hyperglycemia in diabetes.^28^ Keap1 is a regulatory protein that controls the activity of the Nrf2 pathway, which is involved in cellular defense against oxidative stress, making it a promising target for antioxidant and cytoprotective agents.^29^ Therefore, these targets were selected for the present study to further investigate the molecular interactions and potential pharmacological mechanisms of the active compounds. The molecular docking results are presented in Fig. S86 (SI) and Fig. 4–7, illustrating both the binding affinities and detailed interaction patterns within the active sites of the respective target proteins. For the COX-2 target, compounds 1–8, 11, 13, and 14 were docked into the active site and showed binding affinities ranging from −2.059 to −7.631 kcal mol^−1^ (Fig. S86). Among them, compound 11 exhibited the strongest predicted binding affinity of −7.631 kcal mol^−1^. Detailed interaction analysis revealed that compound 11 forms hydrogen bonds with His90 and Arg120, amide–π interaction with Gly526, π–alkyl interaction with Ala527, and π–sigma interactions with Leu352 and Val523 (Fig. 4). These interactions are consistent with key residues observed in the reference ligand, particularly Val523, Leu352, and Ala527, suggesting a similar inhibitory mechanism. In the case of α-glucosidase, compounds 10, 11, 13, and 14 demonstrated binding affinities ranging from −6.142 to −8.238 kcal mol^−1^ (Fig. S86). Compound 10 forms π–anion interactions with Asp443 and Asp542 and π–alkyl interactions with Phe575 and Trp406. Compound 11 forms a hydrogen bond with Arg526, π–π T-shaped interactions with Trp406 and Phe575, π–alkyl interaction with Ala576, and π–anion interaction with Asp542. Compound 13 interacts *via* hydrogen bonding with Asp327, π–alkyl interactions with Tyr299, Trp406, and Phe575, and π–anion interactions with Asp443 and Asp203. Compound 14 forms a hydrogen bond with Arg526, π–alkyl interaction with Ala576, and π–π T-shaped interaction with Trp406. The interaction with Arg526 is also observed in the reference system, indicating a similar binding pattern (Fig. 5). Docking against the XO protein showed that compounds 7–9 and 13 bind within the active site with affinities ranging from −6.112 to −8.265 kcal mol^−1^ (Fig. S86). Compound 7 forms hydrogen bonds with Thr1010, Val1011, and Lys771, along with π–π stacked and π–π T-shaped interactions with Phe914, Phe1013, and Phe649, and π–alkyl and alkyl interactions with Leu873, Leu1014, and Leu648. Compound 8 exhibits π–alkyl interactions with Val1011 and Leu1014, π–sigma interactions with Leu648 and His875, and π–cation interaction with His875. Compound 9 forms a hydrogen bond with Thr1025, π–sulfur interaction with Met1118, π–cation interaction with Lys771, and π–alkyl and alkyl interactions with Val1011 and Leu648, as well as π–π T-shaped interactions with Phe1013 and Phe649. Compound 13 shows π–alkyl interactions with Phe649, Val1011, and Phe1013, π–sulfur interaction with Met1118, π–cation interaction with Lys771, and π–sigma interaction with Leu648. Key residues such as Thr1010, Val1011, Leu1014, and Leu873 are also involved in the reference ligand complex, indicating comparable binding modes (Fig. 6). Regarding the Keap1 protein, compounds 4, 5, 8, 10, 11, 13, and 14 exhibited strong binding affinities ranging from −8.138 to −9.244 kcal mol^−1^ (Fig. S86). Compounds 4 and 5 showed binding energies lower than −9.0 kcal mol^−1^, with 4 demonstrating the strongest predicted affinity of −9.244 kcal mol^−1^. Detailed interaction analysis revealed that compound 4 forms a hydrogen bond with Asn387, π–sigma interaction with Ala556, alkyl and π–alkyl interactions with Arg415, and π–π stacked interaction with Tyr334. Compound 5 forms hydrogen bonds with Ser602, Ser363, and Arg415, along with π–alkyl and alkyl interactions with Ala556 and Tyr572, π–π stacked interaction with Tyr334, and π–cation interaction with Arg415. These interactions are consistent with those observed in the reference complex involving Arg415, Ser602, Tyr334, Ala556, and Tyr572, suggesting a similar inhibitory potential within the Keap1 active site (Fig. 7).

To further assess the dynamic stability of the binding modes obtained from molecular docking, the selected protein–ligand complexes were subjected to 200 ns molecular dynamics (MD) simulations. The stability of the complexes was primarily evaluated by monitoring the RMSD of the protein backbone and the bound ligands (Fig. S87–S90). Overall, the backbone RMSD profiles showed relatively small fluctuations throughout the simulations, suggesting that ligand binding did not induce substantial conformational changes in the investigated proteins. For COX-2 (PDB ID: 5IKR), the complex containing compound 11 exhibited an average backbone RMSD of 0.190 nm, close to that of the reference complex (0.200 nm), indicating overall stability of the protein structure throughout the simulation. The average ligand RMSD of compound 11 was 0.192 nm, compared with 0.119 nm for the reference ligand, suggesting somewhat greater positional flexibility of compound 11 within the binding site while maintaining overall complex stability. A similarly stable backbone profile was observed for α-glucosidase (PDB ID: 2QMJ). Complexes with compounds 10, 11, 13, and 14 exhibited average backbone RMSD values of 0.141, 0.143, 0.136, and 0.148 nm, respectively, demonstrating limited structural deviation during the simulations. Their corresponding average ligand RMSD values were 0.165, 0.234, 0.121, and 0.261 nm, respectively, compared with 0.053 nm for the reference ligand. Among these compounds, compound 13 exhibited the lowest ligand RMSD, followed by compound 10, suggesting better retention of their initial docked conformations than compounds 11 and 14. For xanthine oxidase (XO; PDB ID: 3NVY), the average backbone RMSD values of the complexes with compounds 7–9, and 13 were 0.241, 0.194, 0.211, and 0.250 nm, respectively, compared with 0.254 nm for the reference complex. The corresponding average ligand RMSD values were 0.167, 0.175, 0.146, and 0.109 nm, respectively, whereas the reference ligand exhibited an average value of 0.089 nm. Notably, compound 13 displayed the lowest ligand RMSD among the investigated compounds, indicating relatively limited deviation from its initial binding conformation during the 200 ns simulation. Finally, in the Keap1 system (PDB ID: 4L7B), compounds 4 and 5 showed average backbone RMSD values of 0.159 and 0.186 nm, respectively, which were comparable to that of the reference complex (0.170 nm). Their average ligand RMSD values were also relatively low, at 0.165 and 0.153 nm, respectively, compared with 0.171 nm for the reference ligand. These results indicate that both compounds remained stably accommodated within the Keap1 binding region over the 200 ns simulation period.

Overall, the combined docking and MD results provided complementary structural support for the possible molecular mechanisms underlying the observed biological activities and enabled the prioritisation of promising compounds for further investigation. Future studies should focus on target-specific enzyme inhibition assays and mechanistic evaluation of the Keap1–Nrf2 pathway to experimentally verify the predicted interactions and establish their contribution to the observed anti-inflammatory, antioxidant, anti-XO, and α-glucosidase inhibitory activities.

## 3 Experimental

### 3.1 General experimental procedures

HR-ESI-MS data were acquired on a Q Exactive Orbitrap mass spectrometer (Thermo Scientific, San Jose, CA) coupled with a Thermo Dionex UltiMate 3000 LC system equipped with a heated electrospray ionization (HESI) source. UV spectra were recorded on a Shimadzu UV-2401 UV/vis spectrometer. ECD spectra were acquired on an Applied Photophysics Chirascan spectrometer. NMR spectra were obtained using Bruker Avance 500 and 600 MHz spectrometers, with chemical shifts reported in *δ* (ppm). Column chromatography (CC) was performed using silica gel (Merck, 70–230 mesh), RP-18 (Merck, 75 µm), and Sephadex LH-20 (Merck, 140–550 mesh) as stationary phases. Preparative HPLC was conducted on an Agilent 218 Purification System equipped with two ZORBAX SB-C18 columns (100 mm × 21.2 mm × 5 µm and 250 mm × 9.4 mm × 5 µm). Fractions were monitored by TLC, and spots were visualized by spraying with 5% H_2_SO_4_ in ethanol followed by heating.

### 3.2 Plant material

Fresh leaves of *B. percoriacea* were collected from Xuan Son National Park, Phu Tho Province, Vietnam (21°07′26.10″N 104°57′27.71″E), in January 2025. The scientific name of the plant was authenticated by Assoc. Prof. Dr Nguyen Hoang Tuan (a botanist from the Faculty of Pharmacognosy and Traditional Medicine, Hanoi University of Pharmacy, Vietnam). A voucher specimen (HC051-BP2025) has been deposited at the Department of Chemistry, Vinh University, Nghean Province, Vietnam.

### 3.3 Extraction and isolation

Dried and ground leaves of *B. percoriacea* (BPL, 4.0 kg) were extracted by ultrasonication with MeOH (20 L × 4) at room temperature, then the filtered solution was evaporated to yield a crude residue (387 g). This residue was suspended in distilled water and successively partitioned with *n*-hexane, EtOAc, and *n*-BuOH to afford the corresponding fractions: *n*-hexane (BPLH, 152.4 g), EtOAc (BPLE, 103.6 g), *n*-BuOH (BPLB, 48.0 g), and a water-soluble residue. The BPLH fraction was subjected to silica gel column chromatography (CC), eluting with a gradient of *n*-hexane/EtOAc (100 : 1 to 1 : 1, v/v) to yield 15 fractions (Fr. BPLH1-Fr. BPLH15). Fraction BPLH4 (8.1 g) was chromatographed using a gradient of *n*-hexane/acetone (60 : 1 to 1 : 1, v/v) to give seven subfractions (Fr. BPLH4.1-Fr. BPLH4.7). Purification of fraction BPLH4.3 (0.9 g) by silica gel CC (CH_2_Cl_2_/acetone, 18 : 1 to 1 : 1, v/v) yielded 10 (27.5 mg). Compound 11 (12.8 mg) was obtained by purifying subfraction BPLH4.5 (0.7 g) eluted by CH_2_Cl_2_/acetone (14 : 1 to 1 : 1, v/v). Fraction BPLH5 (4.5 g) was separated *via* silica gel CC and eluted with *n*-hexane/acetone (30 : 1 to 1 : 1, v/v) to yield five subfractions (Fr. BPLH5.1-Fr. BPLH5.5). Fraction BPLH5.3 (1.0 g) was further separated on silica gel CC (*n*-hexane/EtOAc, 20 : 1 to 1 : 1, v/v) and subsequently purified by preparative HPLC system (ACN/H_2_O, 50 : 50, v/v) to yield 1 (10.4 mg). Fraction BPLH7 (6.0 g) was divided into ten subfractions *via* silica gel CC (*n*-hexane/EtOAc, 30 : 1 to 1 : 1, v/v). Compound 8 (74.1 mg) was obtained by purifying subfraction BPLH7.3 (0.5 g) eluted by *n*-hexane/EtOAc (15 : 1 to 1 : 1, v/v). Compound 12 (51.5 mg) was obtained from fraction BPLH7.6 (0.3 g) using a similar procedure. Fraction BPLH7.8 (0.6 g) was purified by silica gel CC (CH_2_Cl_2_/acetone, 10 : 1 to 1 : 1, v/v) followed by preparative TLC to give 13 (10.6 mg). Fraction BPLH8 (2.2 g) was separated on silica gel CC (CH_2_Cl_2_/acetone, 12 : 1 to 1 : 1, vv) and then purified using a Sephadex LH-20 column (CH_2_Cl_2_/MeOH, 1 : 4, v/v) to obtain 14 (83.6 mg). Fraction BPLH12 (3.9 g) was fractionated on silica gel CC (CH_2_Cl_2_/acetone, 10 : 1 to 1 : 1, v/v), then subfraction BPLH12.2 (92.5 mg) was purified by a preparative HPLC system (ACN/H_2_O, 40 : 60, v/v) to yield 6 (52.7 mg). The BPLE fraction (90 g) was chromatographed on silica gel CC (CH_2_Cl_2_/MeOH, 50 : 1 to 1 : 1, v/v) to give seven fractions (Fr. BPLE1-Fr. BPLE7). Fraction BPLE2 (11.4 g) was further separated on silica gel CC (CHCl_3_/MeOH, 45 : 1 to 1 : 1, v/v) to give five subfractions (Fr. BPLE2.1-Fr. BPLE2.5). Fraction BPLE2.2 (0.7 g) was purified by Sephadex LH-20 column (CH_2_Cl_2_/MeOH, 1 : 5, v/v) to yield 7 (58.2 mg). Compound 2 (12.5 mg) was obtained from BPLE2.3 (0.4 g) *via* a preparative HPLC (ACN/H_2_O, 40 : 60, v/v). Fraction BPLE3 (9.5 g) was separated by silica gel CC (CH_2_Cl_2_/MeOH, 30 : 1 to 1 : 1, v/v) and then fraction BPLE3.6 (0.5 g) was purified by a preparative HPLC system (ACN/H_2_O, 55 : 45, v/v) to afford 9 (96 mg). Fraction BPLE4 (7.7 g) was fractionated on silica gel CC (EtOAc/MeOH, 50 : 1 to 1 : 1, v/v). Subsequent silica gel CC of fraction BPLE4.2 (1.8 g) (CH_2_Cl_2_/MeOH, 20 : 1 to 1 : 1, v/v) yielded 4 (48.3 mg), while fraction BPLE4.4 (0.7 g) gave 3 (20.6 mg) after purification *via* silica gel CC and preparative TLC. Fraction BPLE6 (14.8 g) was separated by silica gel CC (EtOAc/MeOH, 50 : 1 to 1 : 1, v/v) to give nine fractions (Fr. BPLE6.1-Fr. BPLE6.9). Fraction BPLE6.2 (1.0 g) was chromatographed on an RP-18 column (acetone/H_2_O, 30 : 70 to 90 : 10, v/v), and subfraction BPLE6.2.3 (0.2 g) was purified by preparative HPLC (ACN/H_2_O, 45 : 55, v/v) to yield 5 (40.1 mg).

#### 3.3.1 Beilschmiedin A (1)

White amorphous powder; UV (MeOH) *λ*_max_ (log *ε*): 211.5 (4.2) nm; C_20_H_26_O_7_; HR-ESI-MS *m*/*z* 379.17557 [M + H]^+^ (calcd for [C_20_H_27_O_7_]^+^, 379.17513); ^1^H NMR (600 MHz, CDCl_3_, *δ*, ppm, *J*/Hz): 5.41 (1H, dd, *J* = 4.2, 1.8 Hz, H-3), 5.34, (1H, d, *J* = 1.8 Hz, H-15a), 4.98 (1H, d, *J* = 1.8 Hz, H-15b), 4.65 (1H, t, *J* = 3.0 Hz, H-1), 3.16 (3H, s, 8-OCH_3_), 2.70 (1H, dd, *J* = 13.2, 3.6 Hz, H-6a), 2.64 (1H, m, H-5), 2.18 (1H, m, H-2a), 2.09 (1H, m, H-2b), 2.27 (1H, td, *J* = 13.2, 1.8 Hz, H-6b), 2.10 (1H, d, *J* = 13.8 Hz, H-9a), 2.07 (3H, s, H-2′), 2.02 (3H, s, H-2″), 1.93 (3H, d, *J* = 1.8 Hz, H-13), 1.88 (1H, d, *J* = 13.8 Hz, H-9b), 1.04 (3H, s, H-14); ^13^C-NMR (150 MHz, CDCl_3_, *δ*, ppm): 171.5 (C-12), 170.2 (C-1′), 169.8 (C-1″), 157.6 (C-7), 144.1 (C-4), 124.9 (C-11), 114.6 (C-15), 106.2 (C-8), 74.7 (C-1), 73.0 (C-3), 50.2 (8-OCH_3_), 42.6 (C-9), 40.8 (C-5), 39.7 (C-10), 30.2 (C-2), 23.8 (C-6), 21.4 (C-2″), 16.5 (C-14), 8.5 (C-13).

#### 3.3.2 Beilschmiedin B (2)

White amorphous powder; UV (MeOH) *λ*_max_ (log *ε*): 212.0 (4.5), 245.0 (3.9), 296.0 (3.5) nm; C_15_H_24_O_3_; HR-ESI-MS *m*/*z* 275.16207 [M + Na]^+^ (calcd for [C_15_H_24_O_3_Na]^+^, 275.16177); ^1^H NMR (600 MHz, CDCl_3_, *δ*, ppm, *J*/Hz): 5.44 (1H, br s, H-6), 5.14 (1H, br s, H-15a), 4.87 (1H, br s, H-15b), 4.34 (1H, d, *J* = 3.0 Hz, H-3), 3.84 (1H, dd, *J* = 3.0, 1.8, Hz, H-2), 3.56 (1H, d, *J* = 1.8 Hz, H-1), 3.29 (1H, br s, H-5), 2.24 (1H, m, H-11), 2.08–2.05 (3H, m, H_2_-8/H-9a), 1.38 (1H, m, H-9b), 1.04 (3H, d, *J* = 7.2 Hz, H-12), 1.03 (3H, d, *J* = 7.2 Hz, H-13), 0.61 (3H, s, H-14); ^13^C-NMR (150 MHz, CDCl_3_, *δ*, ppm): 147.7 (C-4), 144.0 (C-7), 116.8 (C-6), 111.4 (C-15), 79.4 (C-1), 78.3 (C-3), 69.4 (C-2), 38.0 (C-10), 35.0 (C-5), 34.9 (C-11), 31.0 (C-9), 22.9 (C-8), 21.8 (C-13), 21.5 (C-12), 15.4 (C-14).

### 3.4 ECD calculations

The ECD calculations were carried out following the recommendations of previous studies,^30,31^ with a slight modification. The conformational searches of each possible isomer were performed by employing MAESTRO software (Schrodinger LLC, New York, NY, USA), using an energy window of 21 kJ mol^−1^, applying 10 000 steps of the Monte Carlo multiple minimum method with PRCG energy minimization using the Merck Molecular Force Field (MMFF) in the gas phase. Those conformers were then subjected to geometrical optimization and vibrational frequencies calculation using the DFT/wB97XD/TZVP (IEFPCM, MeOH) level with the Gaussian 16 A.03 package (Gaussian Inc., Wallingford, CT, USA). The optimized conformers were then subjected to ECD calculation at TD-DFT/CAM-B3LYP/TZVP (IEFPCM, MeOH) level. ECD curves were Boltzmann averaged, extracted and UV-corrected by SpecDis v.1.7 software with a half-band of 0.3 eV.

### 3.5 Determination of nitric oxide production

The inhibitory effects of the test compounds on NO production were evaluated in RAW 264.7 macrophages (ATCC, Manassas, VA, USA). Cells were seeded in 96-well plates at a density of 5 × 10^4^ cells per well and pretreated with the test compounds or l-NAME (used as a positive control for iNOS inhibition) for 2 h, followed by stimulation with LPS (100 ng mL^−1^) for 24 h. NO levels in the culture supernatants were determined using the Griess reaction.^32^ Absorbance was measured at 535 nm using a Varioskan LUX microplate reader (Thermo Scientific). IC_50_ values were calculated based on the inhibition proportion at five concentrations that maintained cell viability above 80%, as determined by the MTT assay. When the inhibition remained below 50% at the highest concentration without significant cytotoxicity, the IC_50_ was recorded as not determined (N.D).

### 3.6 Xanthine oxidase assay

Xanthine oxidase (XO) inhibitory activity was evaluated as previously described.^33^ The reaction mixture containing the test compounds and XO (0.01 U mL^−1^, pH 7.5) was incubated at 25 °C, followed by the addition of xanthine (150 µM). After incubation, the reaction was terminated by adding HCl, and absorbance was measured at 290 nm using a microplate reader. Quercetin was used as a positive control.

### 3.7 α-Glucosidase inhibition assay

α-Glucosidase inhibitory activity was determined using a modified spectrophotometric method.^34^ The reaction mixture containing phosphate buffer (100 mM), α-glucosidase (1 U mL^−1^), and the sample solution was pre-incubated at 37 °C for 15 min, followed by the addition of 4-nitrophenyl α-d-glucopyranoside and further incubation for 20 min. The reaction was terminated by adding Na_2_CO_3_, and absorbance was measured at 405 nm using a microplate reader. Acarbose was used as a positive control.

### 3.8 Antioxidant assay

ABTS radical scavenging activity was evaluated using a colorimetric method with slight modifications to the previously described procedure.^35^ The ABTS˙^+^ solution was prepared and diluted to an absorbance of 0.70 ± 0.02 at 734 nm. Test compounds were mixed with the ABTS˙^+^ solution and incubated in the dark at room temperature for 5 min. Absorbance was then measured at 734 nm using a microplate reader (Epoch, BioTek, USA). Trolox was used as a positive control.

DPPH radical scavenging activity was determined using a colorimetric method, with slight modifications to the previously described procedure.^36^ Test compounds were mixed with DPPH solution (0.1 mM in methanol) and incubated in the dark at room temperature for 30 min. Absorbance was measured at 517 nm using a microplate reader (Epoch, BioTek, USA). Trolox was used as a positive control.

Hydroxyl radical scavenging activity was evaluated using the CuSO_4_/ascorbate/3-coumarin carboxylic acid (3-CCA) assay, with slight modifications from the reported method.^37^ Hydroxyl radicals were generated from CuSO_4_ (40 µM) and l-ascorbic acid (400 µM) and detected by 3-CCA (100 µM). Test compounds or Trolox were incubated with CuSO_4_ and 3-CCA in phosphate buffer, and the reaction was initiated by adding l-ascorbic acid. Fluorescence was monitored kinetically at Ex/Em = 395/450 nm, and the reaction rate was calculated from the fluorescence slope (ΔF min^−1^). The hydroxyl radical scavenging activity was calculated from the decrease in ΔF min^−1^ relative to the control without test compound, and IC_50_ values were obtained from concentration–response curves.

### 3.9 LDH release assay

RAW 264.7 cells were treated with the test compounds for 24 h. The culture media were then collected, and extracellular lactate dehydrogenase (LDH) levels were determined using a commercial LDH assay kit (Promega) according to the manufacturer's instructions and as previously described.^38^ Briefly, culture medium and LDH detection reagent were mixed at a 1 : 1 ratio and incubated for 30 min at room temperature. Absorbance was measured at 490 nm, and LDH activity was calculated using a standard curve prepared in parallel under identical conditions.

### 3.10 Molecular docking and dynamics

Molecular docking was performed to evaluate the binding affinity of the investigated compounds toward multiple protein targets, including Keap1 (PDB ID: 4L7B), xanthine oxidase (PDB ID: 3NVY), α-glucosidase (PDB ID: 2QMJ), and COX-2 (PDB ID: 5IKR), using the AutoDock Vina version 1.2.3 program.^39–43^ The three-dimensional structures of all proteins were obtained from the RCSB Protein Data Bank and prepared by removing crystallographic water molecules, adding polar hydrogens, and assigning Kollman charges using AutoDockTools software. Co-crystallized compounds such as (1*S*,2*R*)-2-{[(1*S*)-1-[(1,3-dioxo-1,3-dihydro-2*H*-isoindol-2-yl)methyl]-3,4-dihydroisoquinolin-2(1H)-yl]carbonyl}cyclohexanecarboxylic acid (for PDB ID: 4L7B), quercetin (for PDB ID: 3NVY), acarbose (for PDB ID: 2QMJ), and mefenamic acid (for PDB ID: 5IKR) were considered as reference inhibitors. Ligands were optimized geometrically, protonated, and assigned Gasteiger charges before conversion into the PDBQT format for docking, following procedures similar to those reported in previous studies.^44,45^ For COX-2 (PDB ID: 5IKR), the docking grid was centered at *X* = 38.4, *Y* = 1.8, *Z* = 61.1 with a grid size of 24 × 24 × 24 Å and a spacing of 1 Å. For xanthine oxidase (PDB ID: 3NVY), the grid center was set at *X* = 39.4, *Y* = 21.9, *Z* = 20 with identical grid dimensions and spacing. In the case of α-glucosidase (PDB ID: 2QMJ), the grid box was centered at *X* = −21.7, *Y* = −6.3, *Z* = −5.3, while for Keap1 (PDB ID: 4L7B), it was centered at *X* = −2.6, *Y* = 3.3, *Z* = −27.4; both used a grid size of 24 × 24 × 24 Å and spacing of 1 Å. For all docking simulations, the exhaustiveness parameter was set to 400 to ensure extensive conformational sampling. The resulting docking poses were ranked based on their predicted binding affinities (kcal mol^−1^), and the best conformations were selected for further analysis of intermolecular interactions between the ligands and key residues within the active sites of the respective proteins using Discovery Studio Visualizer software.

To further evaluate the dynamic stability of the selected protein–ligand complexes under explicit-solvent conditions, molecular dynamics (MD) simulations were performed using GROMACS version 2023.3.^46,47^ The docked conformations with the most favorable binding poses were used as the initial structures for MD simulations. The protein–ligand systems were parameterized using an AMBER99SB-ILDN force field and placed in a triclinic simulation box, followed by solvation with explicit TIP3P water molecules.^48^ Counterions were added as required to neutralize the net charge of each system. Following energy minimization, the systems were equilibrated under canonical (NVT) and isothermal–isobaric (NPT) ensembles before the production simulations. Each protein–ligand complex was subsequently subjected to a 200 ns MD simulation under periodic boundary conditions. The resulting trajectories were analyzed to assess the structural stability of the complexes, including protein backbone root-mean-square deviation (RMSD) and ligand RMSD.

## 4 Conclusion

The present study focuses on the chemical constituents and multi-target biological profiles of *B. percoriacea* leaves collected in Vietnam. Two novel sesquiterpenes, beilschmiedins A–B (1–2), along with twelve known compounds (3–14), were successfully isolated and structurally elucidated. The isolated compounds demonstrated promising *in vitro* anti-inflammatory, antioxidant, and α-glucosidase inhibitory activities, supported by molecular docking and 200 ns MD simulations. However, these conclusions are bounded by the *in vitro* and computational nature of the current dataset. The lack of cell-based enzyme validation, unknown pharmacokinetic parameters, and the absence of *in vivo* studies represent major limitations to establishing genuine therapeutic applications. Subsequent studies involving cellular assays and *in vivo* evaluations are required to confirm the bioactivity and bioavailability of these lead compounds.

## Author contributions

Conceptualization: Hieu Tran-Trung, Le Duc Giang; isolation and identification: Hieu Tran-Trung, Phan Quoc Hung, Hoang Van Trung, Dau Xuan Duc, Hieu Nguyen-Ngoc, Cao Van Anh; bioassay: Nguyen Thi Chung, Hoang Van Trung, Nguyen Van Quoc, Duc-Vinh Pham; computational studies: Duc-Vinh Pham, Nguyen Xuan Ha; writing – original draft preparation: Hoang Van Trung, Nguyen Van Quoc, Hieu Nguyen-Ngoc; writing – review and editing: Hieu Tran-Trung, Le Duc Giang; supervision: Dau Xuan Duc; project administration: Le Duc Giang. All authors have read and agreed to the published version of the manuscript.

## Conflicts of interest

The authors declare no competing financial interest.

## Supplementary Material

RA-OLF-D6RA04523E-s001

## Data Availability

The data supporting the findings of this study are available within the article and its supplementary information (SI) file. Any additional data related to this study are available from the corresponding author upon reasonable request at leducgiang@gmail.com. Supplementary information is available. See DOI: https://doi.org/10.1039/d6ra04523e.

## References

[cit1] Salleh W. M. N. H. W., Ahmad F., Yen K. H., Zulkifli R. M. (2015). Trop. J. Pharm. Res..

[cit2] Dao T. B. P., Linh N. N., Ngoc T. N. H., Hanh T. N., Anh V. C., Son N. T. (2026). Med. Chem. Res..

[cit3] Williams R. B., Martin S. M., Hu J. F., Norman V. L., Goering M. G., Loss S., Johnson M. O., Eldridge G. R., Starks C. M. (2012). J. Nat. Prod..

[cit4] Huang Y. T., Chang H. S., Wang G. J., Cheng M. J., Chen C. H., Yang Y. J., Chen I. S. (2011). J. Nat. Prod..

[cit5] Beilschmiedia, Beilschmiedia percoriacea in Flora of China @ efloras.org, http://www.efloras.org/florataxon.aspx?flora_id=2&taxon_id=200008672, accessed 25 March 2026

[cit6] Feng H., Jiang Y., Cao H., Shu Y., Yang X., Zhu D., Shao M. (2022). Heliyon.

[cit7] Dai Y., Liu L., Xie G., Chen Y., Qin X., Wang Q., Li J., Qin M. (2014). Fitoterapia.

[cit8] Jiang Z. P., Su R., Chen M. T., Li J. Y., Chen H. Y., Yang L., Liu F. F., Liu J., Xu C. J., Li W. S., Rao Y., Huang L. (2024). Phytochemistry.

[cit9] Snatzke G. (1968). Angew. Chem., Int. Ed. Engl..

[cit10] Bodensieck A., Fabian W. M., Kunert O., Belaj F., Jahangir S., Schühly W., Bauer R. (2010). Chirality.

[cit11] Cunha C. L., Antonio P. V. G. D., Lustosa M. D. C. G., Cruz Á. B., Holzbach J. C., Pereira D. H., Nascimento I. R. (2023). J. Braz. Chem. Soc..

[cit12] Messiano G. B., Vieira L., Machado M. B., Lopes L. M., Bortoli S. A. D., Schpector J. Z. (2008). J. Agric. Food Chem..

[cit13] Tung T. H., Hue C. T., Giap T. H., Thoa H. T., Dung N. A., Hang N. T. M., Hung N. V., Thanh L. N. (2017). Vietnam J. Chem..

[cit14] Peng W., Yang C., Zhan R., Chen Y. (2016). Nat. Prod. Res..

[cit15] Piao X. L., Jang M. H., Cui J., Piao X. (2008). Bioorg. Med. Chem. Lett..

[cit16] Song Q. Y., Zhang C. J., Li Y., Wen J., Zhao X. W., Liu Z. L., Gao K. (2013). Phytochem. Lett..

[cit17] Liu J. S., Huang M. F., Gao Y. L., Findlay J. A. (1981). Can. J. Chem..

[cit18] Harada K., Kubo M., Horiuchi H., Ishii A., Esumi T., Hioki H., Fukuyama Y. (2015). J. Org. Chem..

[cit19] Li Y. F., Jiang Y., Huang J. F., Yang G. Z. (2013). J. Asian Nat. Prod. Res..

[cit20] Jian-Min Y., Yao-Zu C., Su-Ming H., Jin-Long C., Yu-Xin C. (1989). Phytochemistry.

[cit21] Silva F. A. A., Albuquerque S., Silva M. L. E., Eberlin M. N., Tomazela D. M., Bastos J. K. (2004). J. Nat. Prod..

[cit22] Zou Y. Y., Wang D. W., Yan Y. M., Cheng Y. X. (2021). Chem. Biodiversity.

[cit23] Ma C., Wang C., Zhang Y., Li Y., Fu K., Gong L., Zhou H., Li Y. (2023). Biomed. Pharmacother..

[cit24] Rosly H. A., Roof A. N. M., Ramli S., Buranasudja V., Rojsitthisak P., Sritularak B., Halim H. (2021). J. Pharm. Res. Int..

[cit25] Vona R., Pallotta L., Cappelletti M., Severi C., Matarrese P. (2021). Antioxidants.

[cit26] Ferrer M. D., Busquets-Cortés C., Capó X., Tejada S., Tur J. A., Pons A., Sureda A. (2019). Curr. Med. Chem..

[cit27] Furuhashi M. (2020). Am. J. Physiol.: Endocrinol. Metab..

[cit28] Bhatnagar A. and Mishra A., in Natural Products as Enzyme Inhibitors, Springer, Singapore, 2022, pp. 269–283

[cit29] Kaspar J. W., Niture S. K., Jaiswal A. K. (2009). Free Radical Biol. Med..

[cit30] Pescitelli G., Bruhn T. (2016). Chirality.

[cit31] Pham G. N., Furno M. F., Garcia-Sanchez J. A., Munro P., Abdoul-Latif F. M., Boyer L., Varese G. C., Mehiri M. (2025). Molecules.

[cit32] Tsikas D. (2007). J. Chromatogr. B.

[cit33] Duong N. T., Vinh P. D., Thuong P. T., Hoai N. T., Thanh L. N., Bach T. T., Nam N. H., Anh N. H. (2017). Asian Pac. J. Trop. Med..

[cit34] Shai L. J., Magano S. R., Lebelo S. L., Mogale A. M. (2011). J. Med. Plants Res..

[cit35] Re R., Pellegrini N., Proteggente A., Pannala A., Yang M., Rice-Evans C. (1999). Free Radical Biol. Med..

[cit36] Blois M. S. (1958). Nature.

[cit37] Gharai P. K., Khan J., Pradhan K., Mallesh R., Garg S., Arshi M. U., Barman S., Ghosh S. (2024). Chem. Neurosci..

[cit38] Gharai P. K., Khan J., Mallesh R., Garg S., Gupta S., Jaisankar P., Ghosh S. (2025). ACS Chem. Neurosci..

[cit39] Jnoff E., Albrecht C., Barker J. J., Barker O., Beaumont E., Bromidge S., Brookfield F., Brooks M., Bubert C., Ceska T., Corden V., Dawson G., Duclos S., Fryatt T., Genicot C., Jigorel E., Kwong J., Maghames R., Mushi I., Pike R., Sands Z. A., Smith M. A., Stimson C. C., Courade J. P. (2014). ChemMedChem.

[cit40] Orlando B. J., Malkowski M. G. (2016). J. Biol. Chem..

[cit41] Cao H., Pauff J. M., Hille R. (2014). J. Nat. Prod..

[cit42] Sim L., Quezada-Calvillo R., Sterchi E. E., Nichols B. L., Rose D. R. (2008). J. Mol. Biol..

[cit43] Eberhardt J., Santos-Martins D., Tillack A. F., Forli S. (2021). J. Chem. Inf. Model..

[cit44] Thuy P. T., Ha N. X. (2024). Free Radical Res..

[cit45] Giang D. T. T., Hieu T. T., Tuan N. H., Ha N. X., Nguyen D. K., Chanh N. M., Hien N. T. T., Thuan V. T., Hang T. T. T., Giang N. T. T., Trung H. V., Essent J. (2024). J. Essent. Oil Bear. Plants.

[cit46] Bekker H. , Berendsen H. J., Dijkstra E. J., Achterop S., Vondrumen R. V., Vanderspoel D., Sijbers A., Keegstra H. and Renardus M. K. R., in Physics Computing 92, World Scientific, 1993, vol. 92, pp. 252–256

[cit47] Berendsen H. J., van der Spoel D., van Drunen R. (1995). Comput. Phys. Commun..

[cit48] Lindorff-Larsen K., Piana S., Palmo K., Maragakis P., Klepeis J. L., Dror R. O., Shaw D. E. (2010). Proteins.

