# Supplementary material for: Bioactive compounds from Beilschmiedia percoriacea leaves: isolation, structural characterization, and biological evaluations
Source: RSC Adv. 2026 Aug 20;16(41):49144–57. doi: 10.1039/d6ra04523e (PMC13491652; doi:10.1039/d6ra04523e)
Supplement: Supplementary file 1 [file RA-016-D6RA04523E-s001.pdf]

Supplementary Information (SI) for RSC Advances.
This journal is © The Royal Society of Chemistry 2026

Supporting information

Bioactive Compounds from *Beilschmiedia percoriacea* leaves: Isolation, Structural Characterization, and Biological Evaluations

Hieu Tran-Trung[a], Dau Xuan Duc[a], Nguyen Thi Chung[a], Phan Quoc Hung[a], Hoang Van Trung[b], Nguyen Van Quoc[b], Hieu Nguyen-Ngoc[c], Cao Van Anh[d], Duc-Vinh Pham[e], Nguyen Xuan Ha[f], Le Duc Giang[a*]

[a]Department of Chemistry, Vinh University, Nghean, Vietnam.

[b]School of Chemistry, Biology and Environment, Vinh University, Nghean, Vietnam.

[c]Faculty of Pharmacy, School of Medicine and Pharmacy, Phenikaa University, Hanoi, Vietnam.

[d]Institute of Pharmaceutical Education, Vietnam Military Medical University, Hanoi, Vietnam.

[e]Department of Pharmacology, Hanoi University of Pharmacy, Hanoi, Vietnam.

[f]Institute of Chemistry, Vietnam Academy of Science and Technology, Hanoi, Vietnam.

*E-mail: leducgiang@gmail.com

List of Figures

Figure S1. HR-ESI-MS of compound 1
Figure S2. 1H-NMR spectrum of compound 1 in CDCl3
Figure S3. Expanded 1H-NMR spectrum of compound 1
Figure S4. Expanded 1H-NMR spectrum of compound 1
Figure S5. Expanded 1H-NMR spectrum of compound 1
Figure S6. 13C-NMR spectrum of compound 1 in CDCl3
Figure S7. Expanded 13C-NMR spectrum of compound 1
Figure S8. Expanded 13C-NMR spectrum of compound 1
Figure S9. HSQC spectrum of compound 1
Figure S10. Expanded HSQC spectrum of compound 1
Figure S11. Expanded HSQC spectrum of compound 1
Figure S12. HMBC spectrum of compound 1
Figure S13. Expanded HMBC spectrum of compound 1
Figure S14. Expanded HMBC spectrum of compound 1
Figure S15. Expanded HMBC spectrum of compound 1
Figure S16. COSY spectrum of compound 1
Figure S17. ROESY spectrum of compound 1
Figure S18. UV spectrum of compound 1
Figure S19. HR-ESI-MS of compound 2
Figure S20. 1H-NMR spectrum of compound 2 in CDCl3
Figure S21. Expanded 1H-NMR spectrum of compound 2
Figure S22. Expanded 1H-NMR spectrum of compound 2
Figure S23. 13C-NMR spectrum of compound 2 in CDCl3
Figure S24. Expanded 13C-NMR spectrum of compound 2
Figure S25. Expanded 13C-NMR spectrum of compound 2
Figure S26. HSQC spectrum of compound 2
Figure S27. Expanded HSQC spectrum of compound 2
Figure S28. Expanded HSQC spectrum of compound 2
Figure S29. HMBC spectrum of compound 2
Figure S30. Expanded HMBC spectrum of compound 2
Figure S31. Expanded HMBC spectrum of compound 2
Figure S32. Expanded HMBC spectrum of compound 2
Figure S33. Expanded HMBC spectrum of compound 2
Figure S34. COSY spectrum of compound 2
Figure S35. ROESY spectrum of compound 2
Figure S36. UV spectrum of compound 2
Figure S37. 1H-NMR spectrum of compound 3
Figure S38. 13C-NMR spectrum of compound 3
Figure S39. HSQC spectrum of compound 3
Figure S40. HMBC spectrum of compound 3
Figure S41. 1H-NMR spectrum of compound 4
Figure S42. 13C-NMR spectrum of compound 4
Figure S43. HSQC spectrum of compound 4
Figure S44. HMBC spectrum of compound 4
Figure S45. 1H-NMR spectrum of compound 5
Figure S46. 13C-NMR spectrum of compound 5
Figure S47. HSQC spectrum of compound 5
Figure S48. HMBC spectrum of compound 5
Figure S49. 1H-NMR spectrum of compound 6
Figure S50. 13C-NMR spectrum of compound 6
Figure S51. HSQC spectrum of compound 6

Figure S52. HMBC spectrum of compound 6
Figure S53. 1H-NMR spectrum of compound 7
Figure S54. 13C-NMR spectrum of compound 7
Figure S55. HSQC spectrum of compound 7
Figure S56. HMBC spectrum of compound 7
Figure S57. 1H-NMR spectrum of compound 8
Figure S58. 13C-NMR spectrum of compound 8
Figure S59. HSQC spectrum of compound 8
Figure S60. HMBC spectrum of compound 8
Figure S61. 1H-NMR spectrum of compound 9
Figure S62. 13C-NMR spectrum of compound 9
Figure S63. HSQC spectrum of compound 9
Figure S64. HMBC spectrum of compound 9
Figure S65. 1H-NMR spectrum of compound 10
Figure S66. 13C-NMR spectrum of compound 10
Figure S67. HSQC spectrum of compound 10
Figure S68. HMBC spectrum of compound 10
Figure S69. 1H-NMR spectrum of compound 11
Figure S70. 13C-NMR spectrum of compound 11
Figure S71. HSQC spectrum of compound 11
Figure S72. HMBC spectrum of compound 11
Figure S73. 1H-NMR spectrum of compound 12
Figure S74. 13C-NMR spectrum of compound 12
Figure S75. HSQC spectrum of compound 12
Figure S76. HMBC spectrum of compound 12
Figure S77. 1H-NMR spectrum of compound 13
Figure S78. 13C-NMR spectrum of compound 13
Figure S79. HSQC spectrum of compound 13
Figure S80. HMBC spectrum of compound 13
Figure S81. 1H-NMR spectrum of compound 14
Figure S82. 13C-NMR spectrum of compound 14
Figure S83. HSQC spectrum of compound 14
Figure S84. HMBC spectrum of compound 14
Figure S85. Effects of isolated compounds on LDH release in Raw 264.7 macrophages.
Figure S86. Binding affinity (kcal/mol) of selected compounds with their corresponding target proteins
Figure S87. Root-mean-square deviation (RMSD) profiles of the protein backbone and ligands during 200 ns molecular dynamics simulations of α-glucosidase (PDB ID: 2QMJ) complexes.
Figure S88. Root-mean-square deviation (RMSD) profiles of the protein backbone and ligands during 200 ns molecular dynamics simulations of COX-2 (PDB ID: 5IKR) complexes.
Figure S89. Root-mean-square deviation (RMSD) profiles of the protein backbone and ligands during 200 ns molecular dynamics simulations of Keap1 (PDB ID: 4L7B) complexes.
Figure S90. Root-mean-square deviation (RMSD) profiles of the protein backbone and ligands during 200 ns molecular dynamics simulations of XO (PDB ID: 3NVY) complexes.
Figure S91. HPLC chromatograms of the isolated compounds 1–14.

List of Table

Table S1. Cytotoxicity of isolated compounds in RAW 264.7 cells and their selectivity indices for NO inhibitory activity.
Table S2. Similarity factor for experimental ECD spectrum of 1 and calculated curve of 1a.
Table S3. Similarity factor for experimental ECD spectrum of 2 and calculated curve of 2b.
Table S4. Gibbs Free Energy and Boltzmann Population of conformers of isomer 1a.
Table S5. Gibbs Free Energy and Boltzmann Population of conformers of isomer 1b.
Table S6. Gibbs Free Energy and Boltzmann Population of conformers of isomer 2a.
Table S7. Gibbs Free Energy and Boltzmann Population of conformers of isomer 2b.
Table S8. The Cartesian coordinates of the optimized conformers.

.

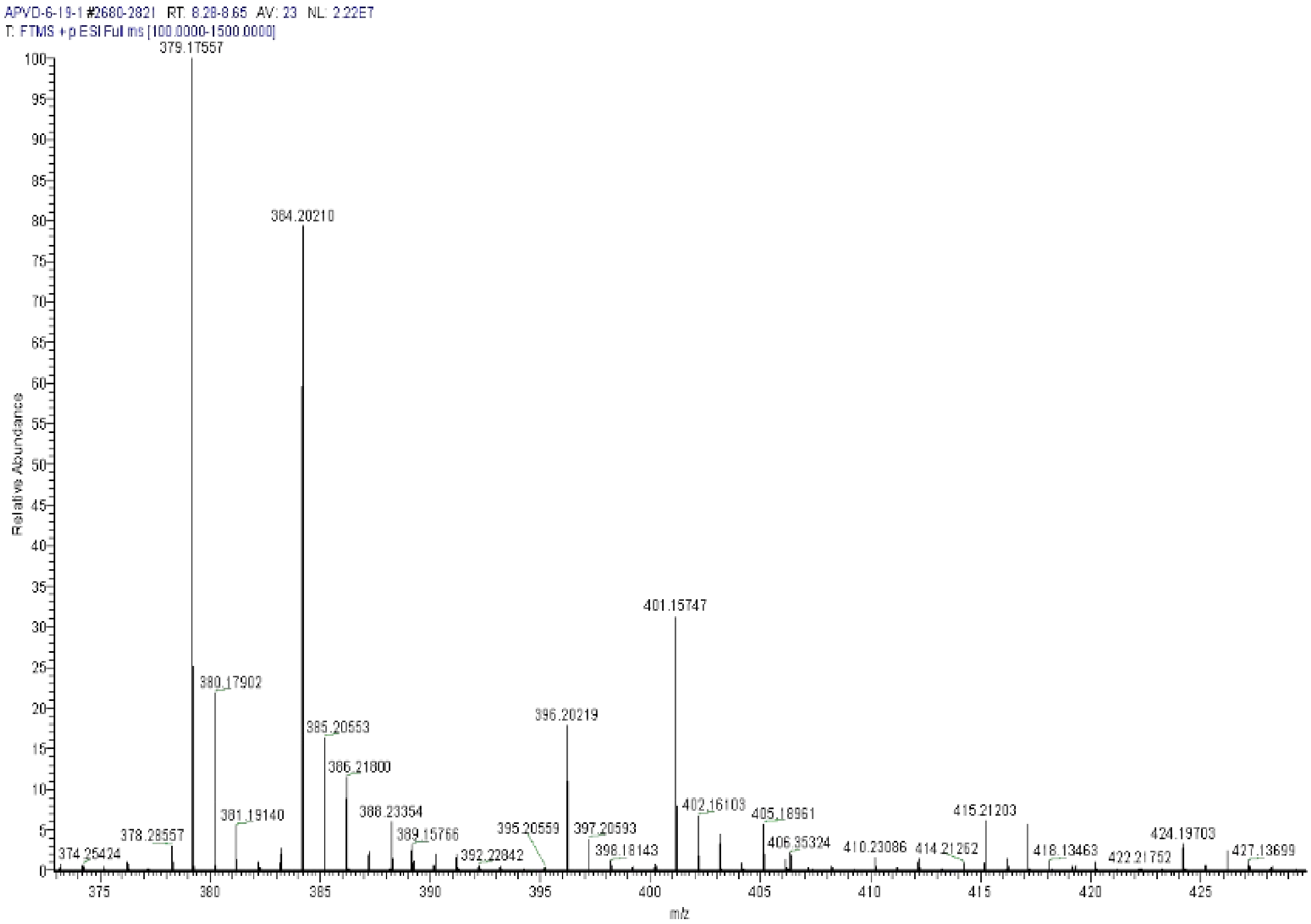

Figure S1. HR-ESI-MS of compound 1

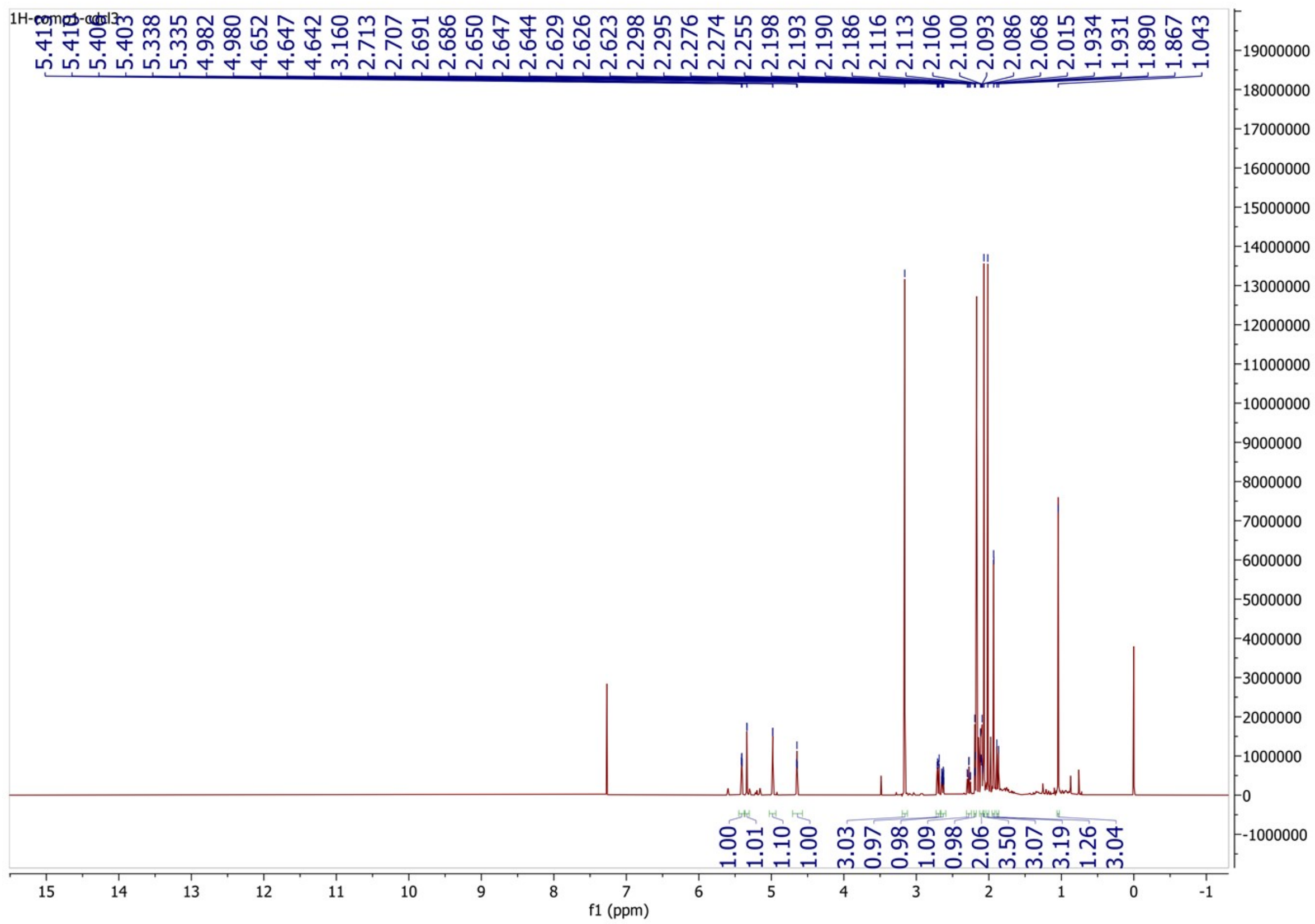

Figure S2. 1H-NMR spectrum of compound 1 in CDCl3

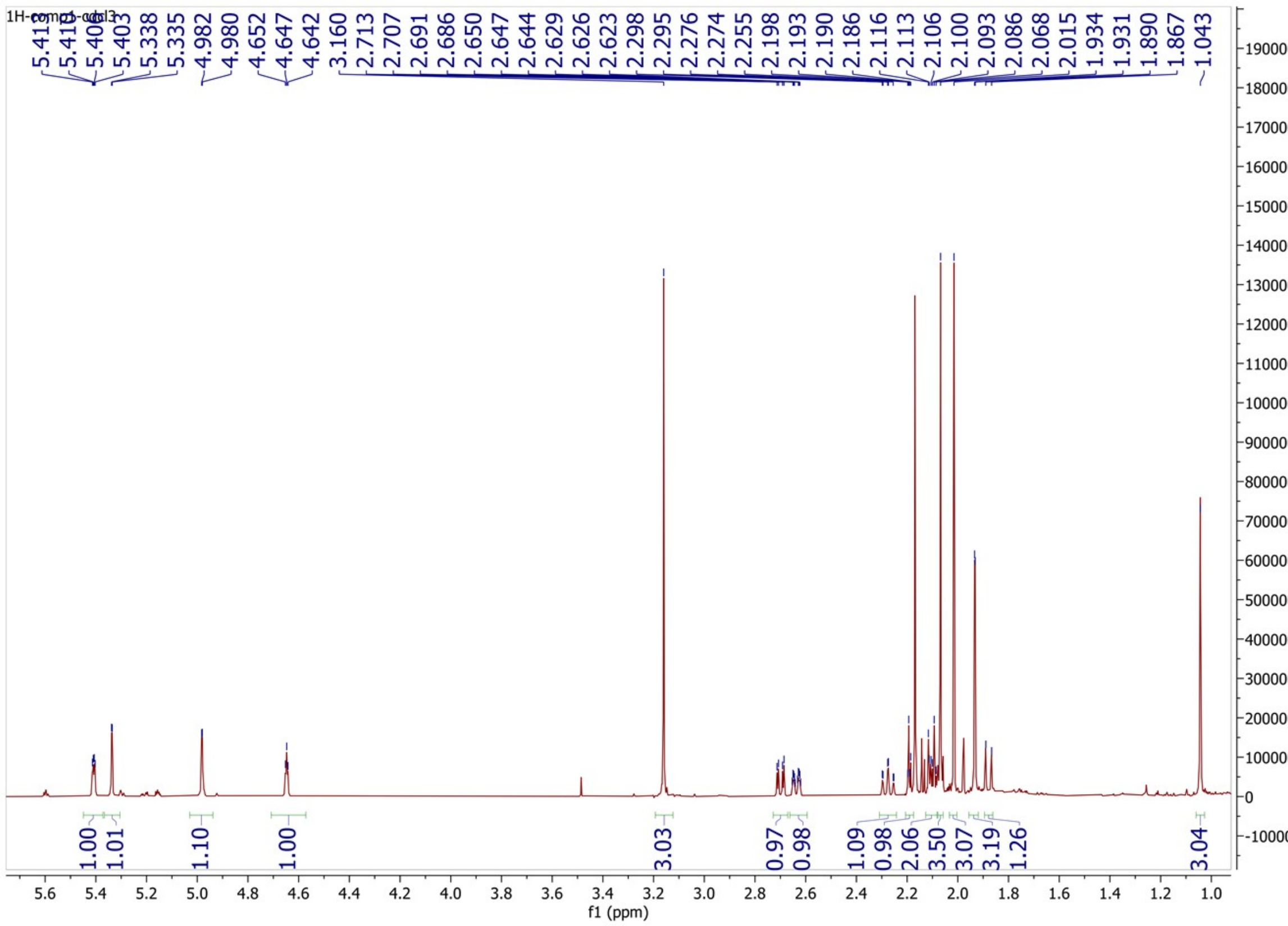

Figure S3. Expanded 1H-NMR spectrum of compound 1

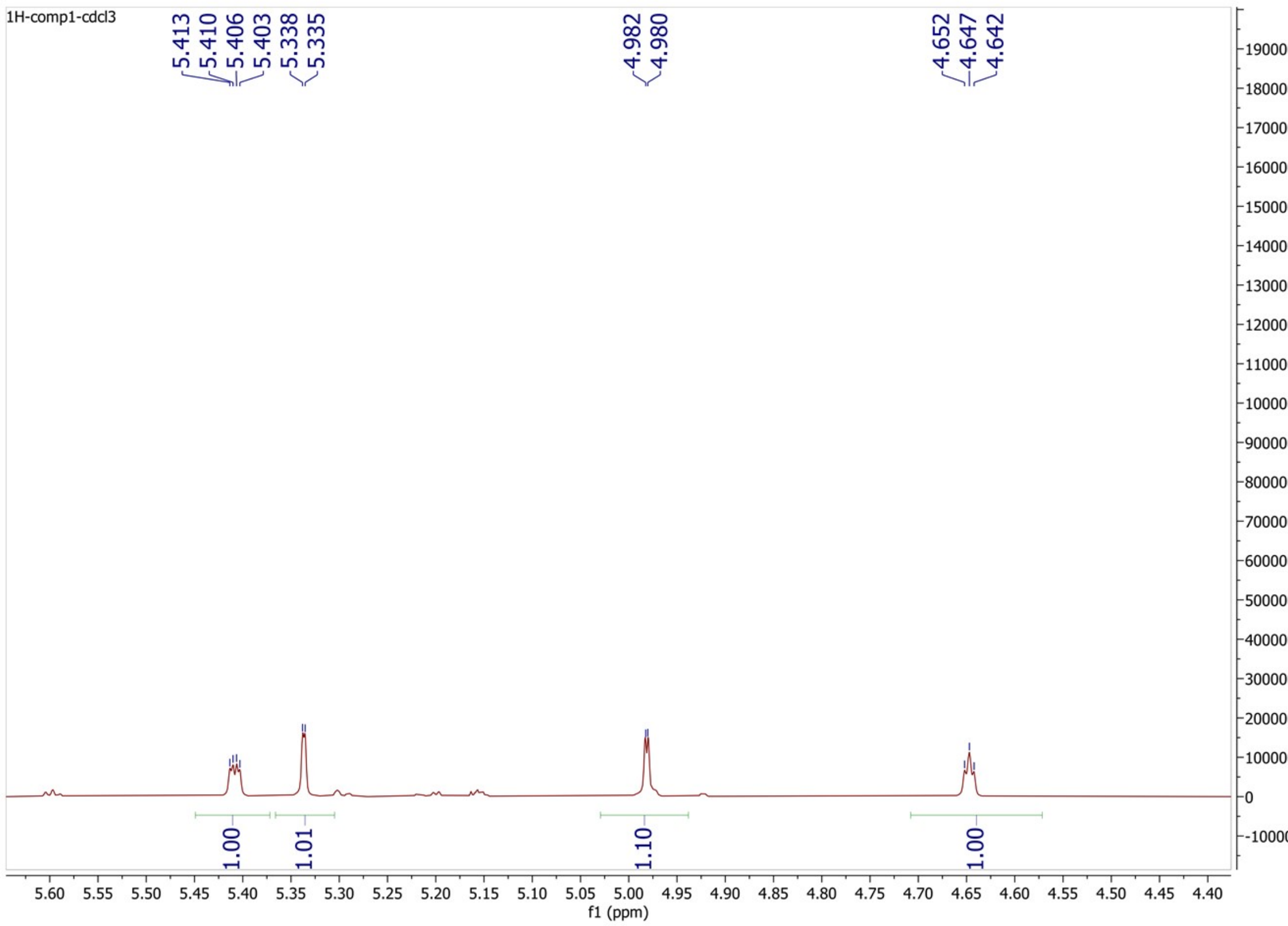

Figure S4. Expanded 1H-NMR spectrum of compound 1

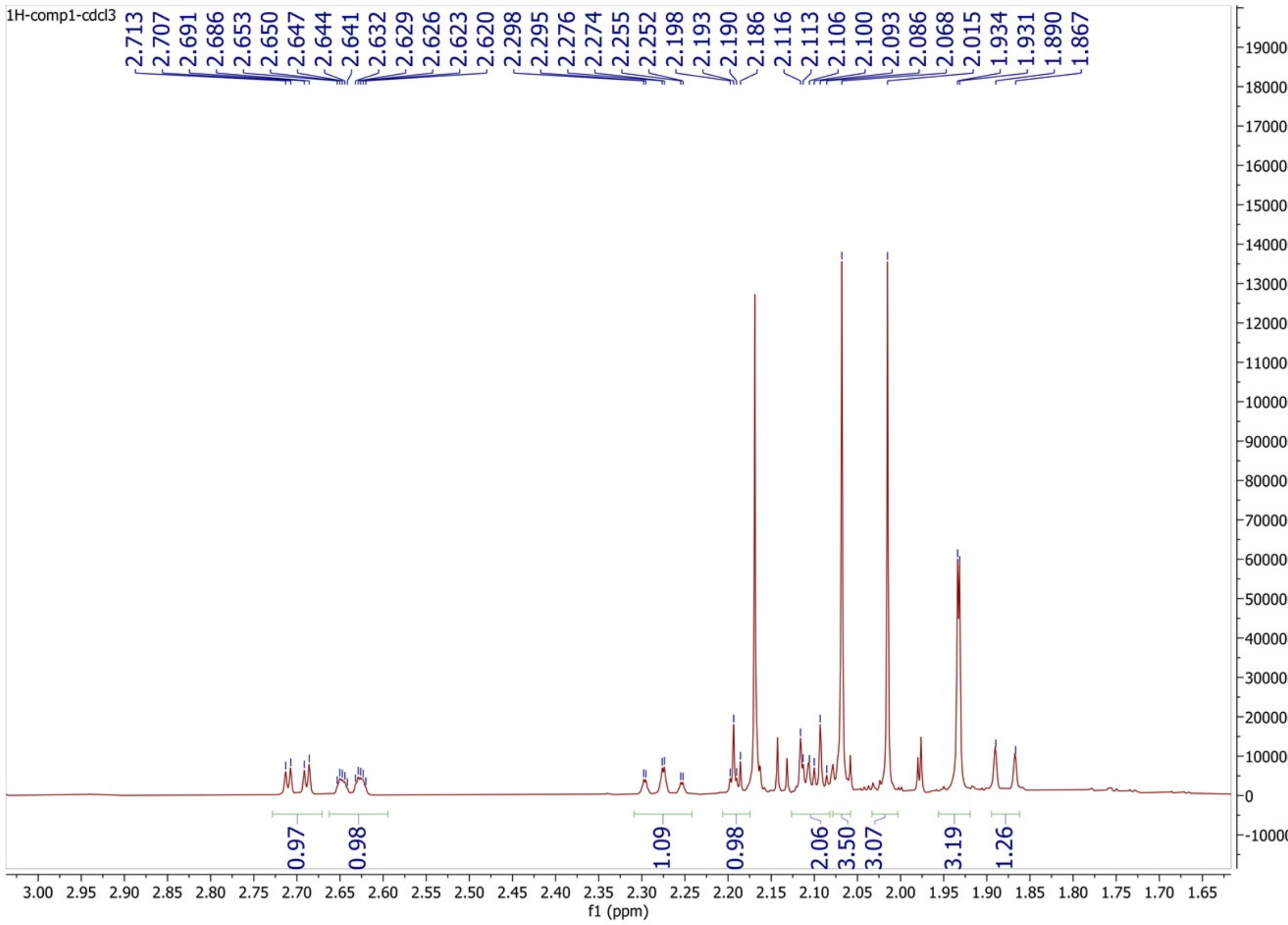

Figure S5. Expanded 1H-NMR spectrum of compound 1

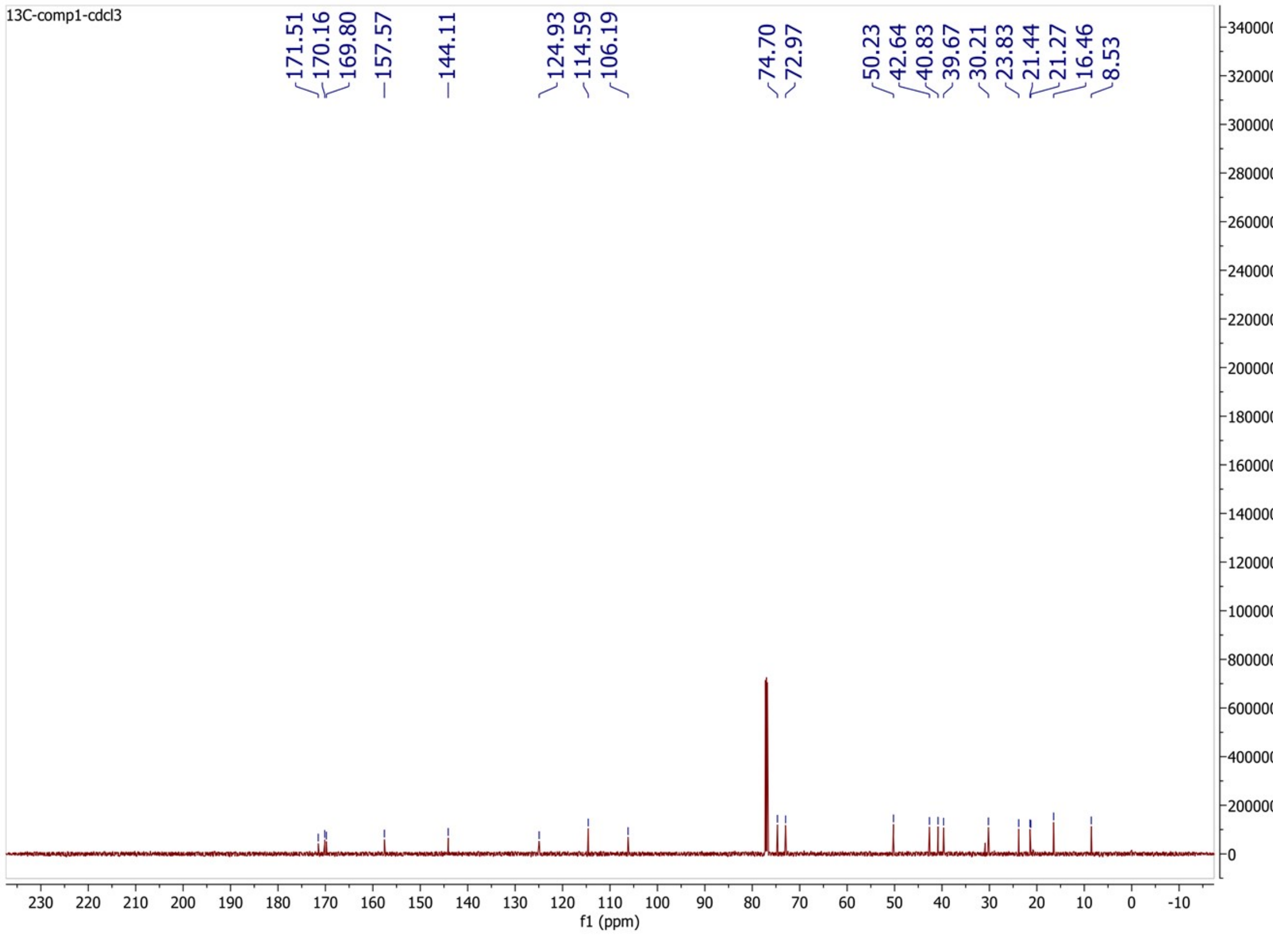

Figure S6. 13C-NMR spectrum of compound 1 in CDCl3

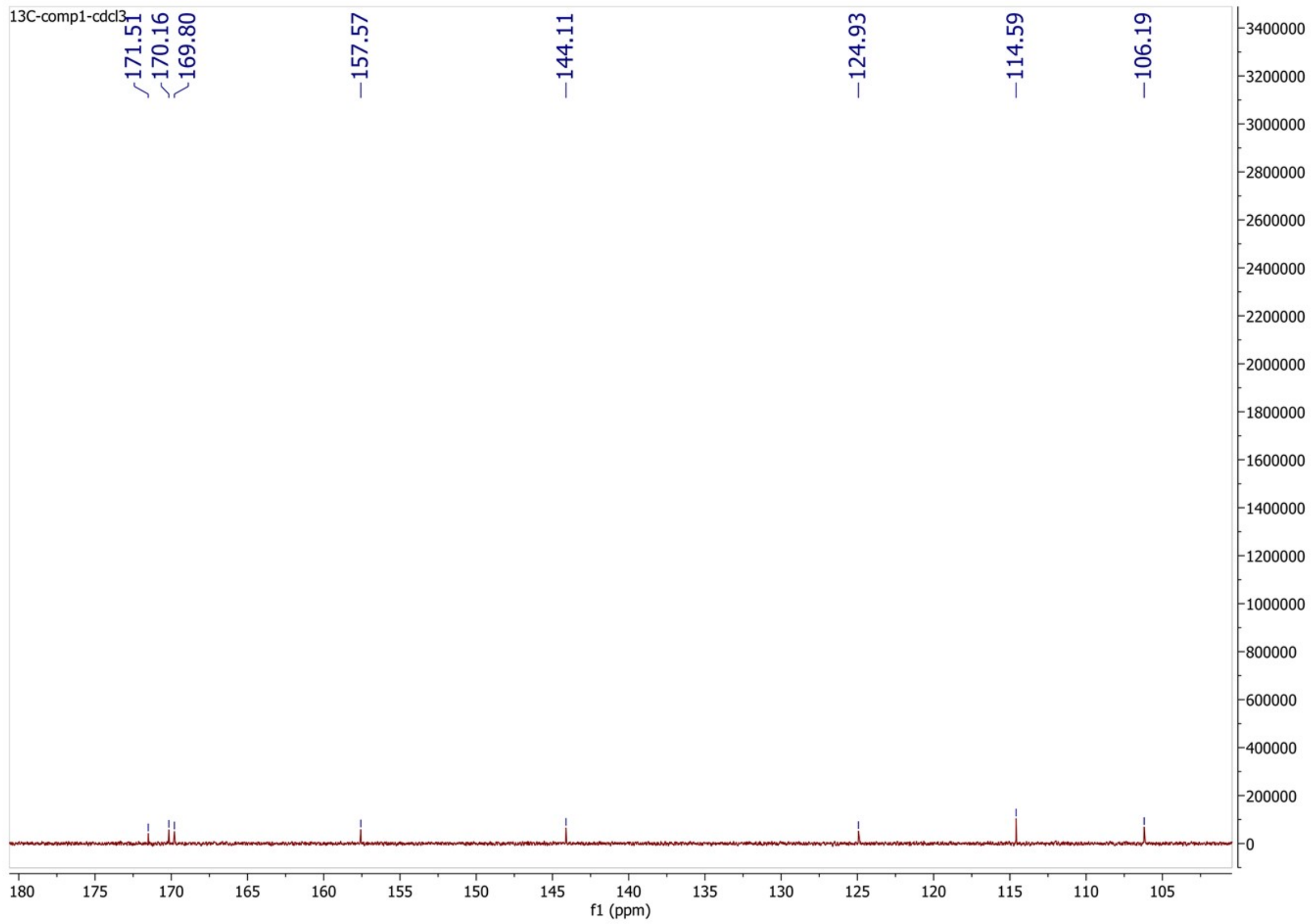

Figure S7. Expanded 13C-NMR spectrum of compound 1

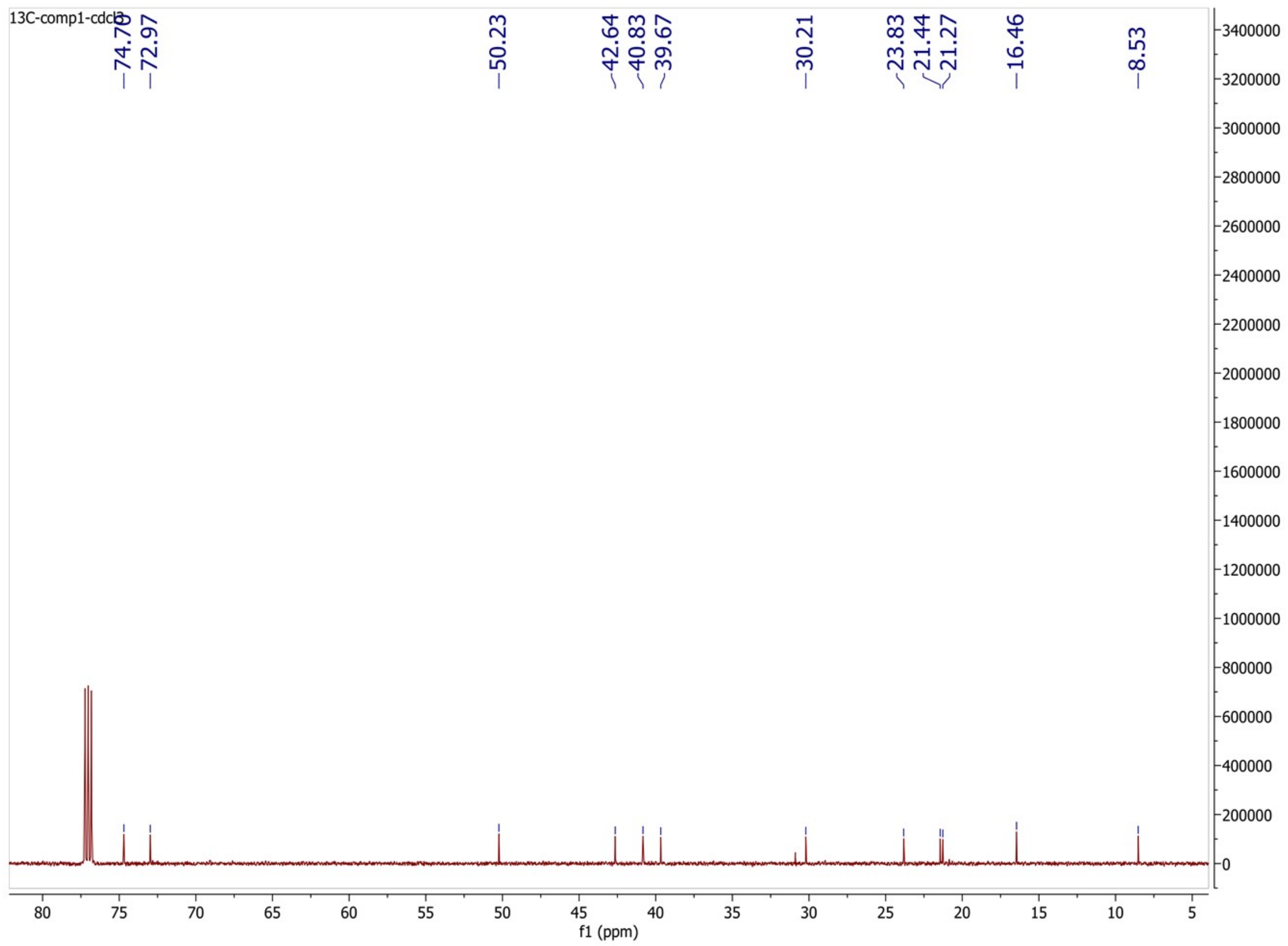

Figure S8. Expanded 13C-NMR spectrum of compound 1

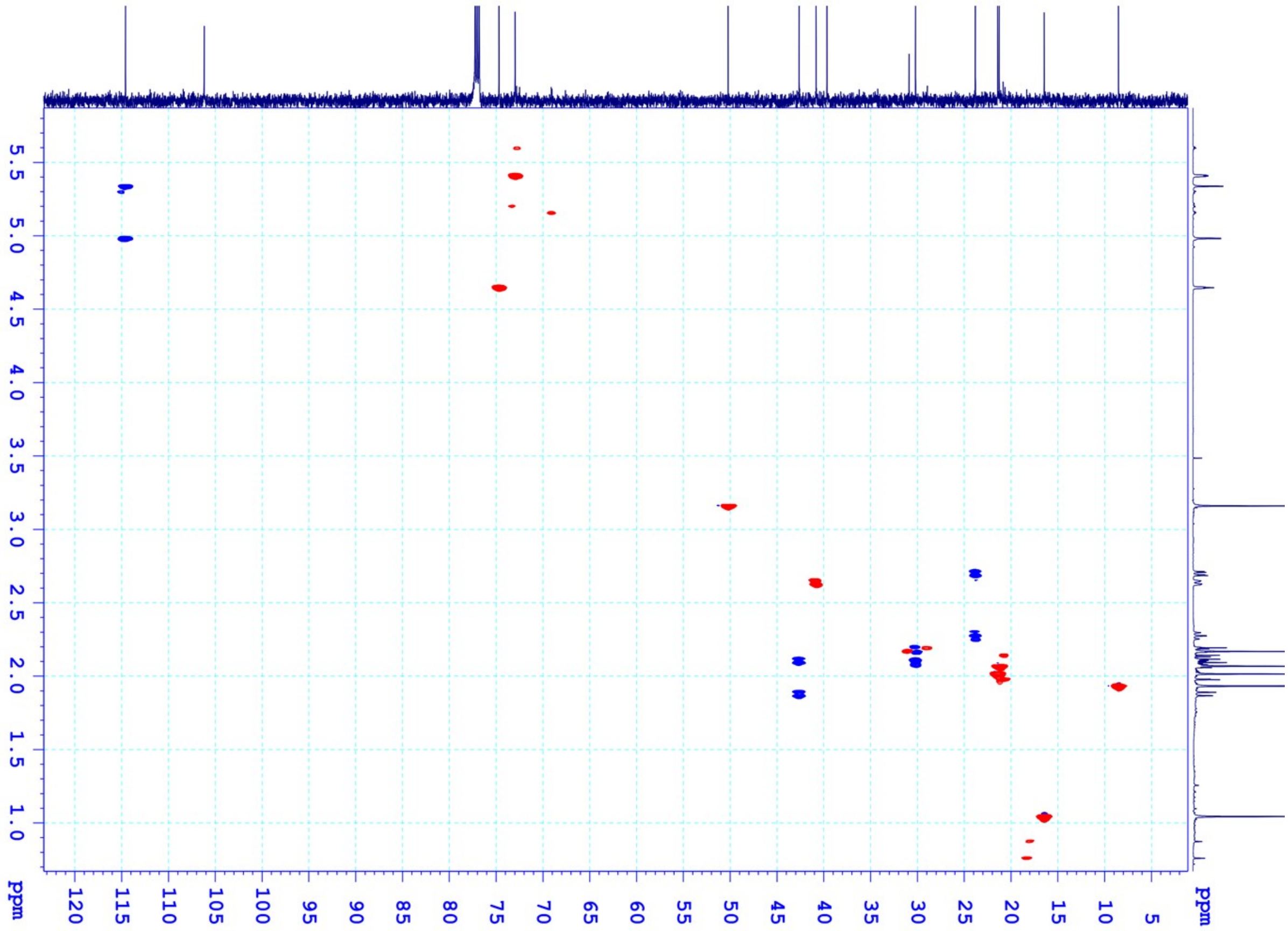

Figure S9. HSQC spectrum of compound 1

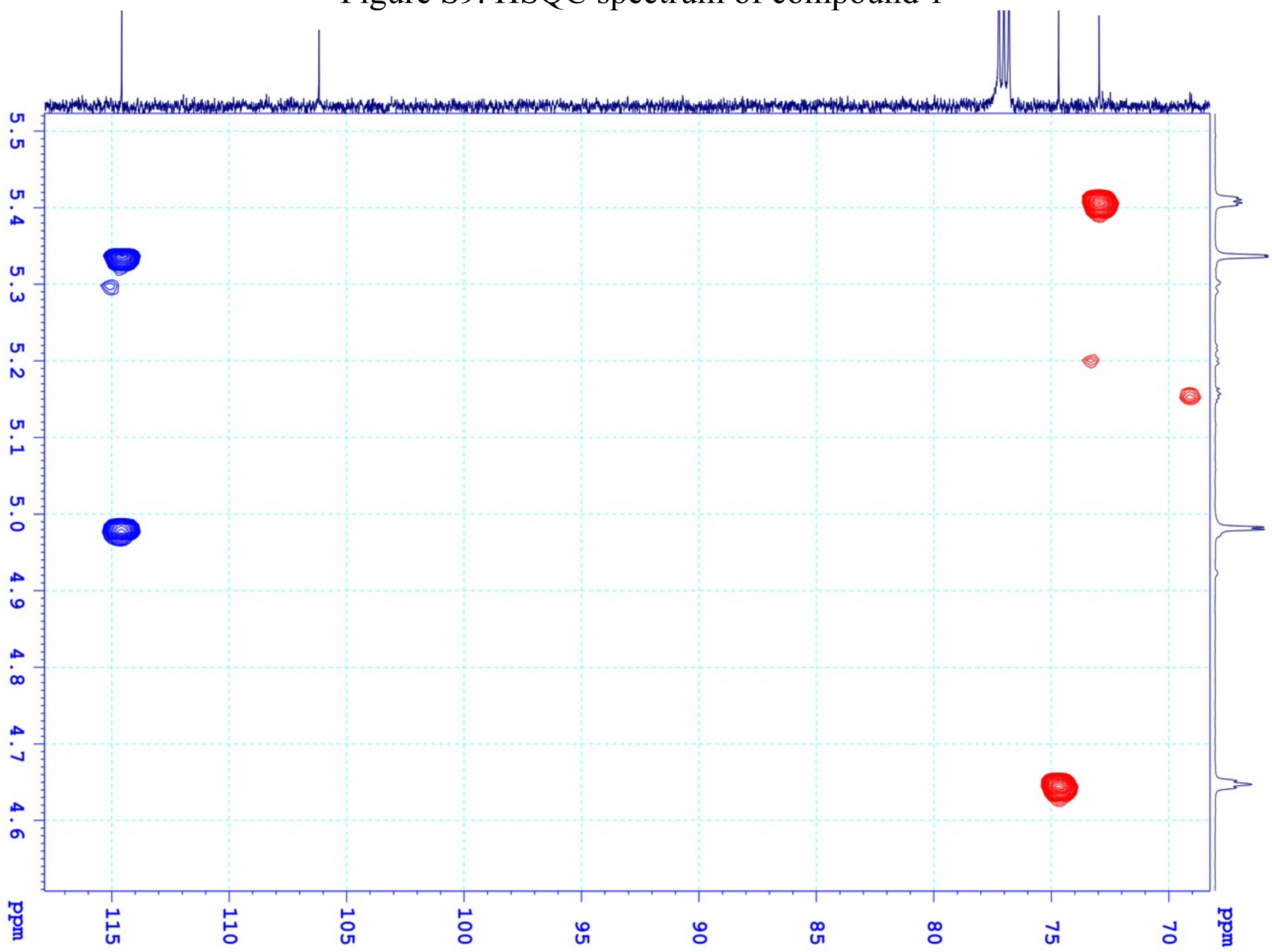

Figure S10. Expanded HSQC spectrum of compound 1

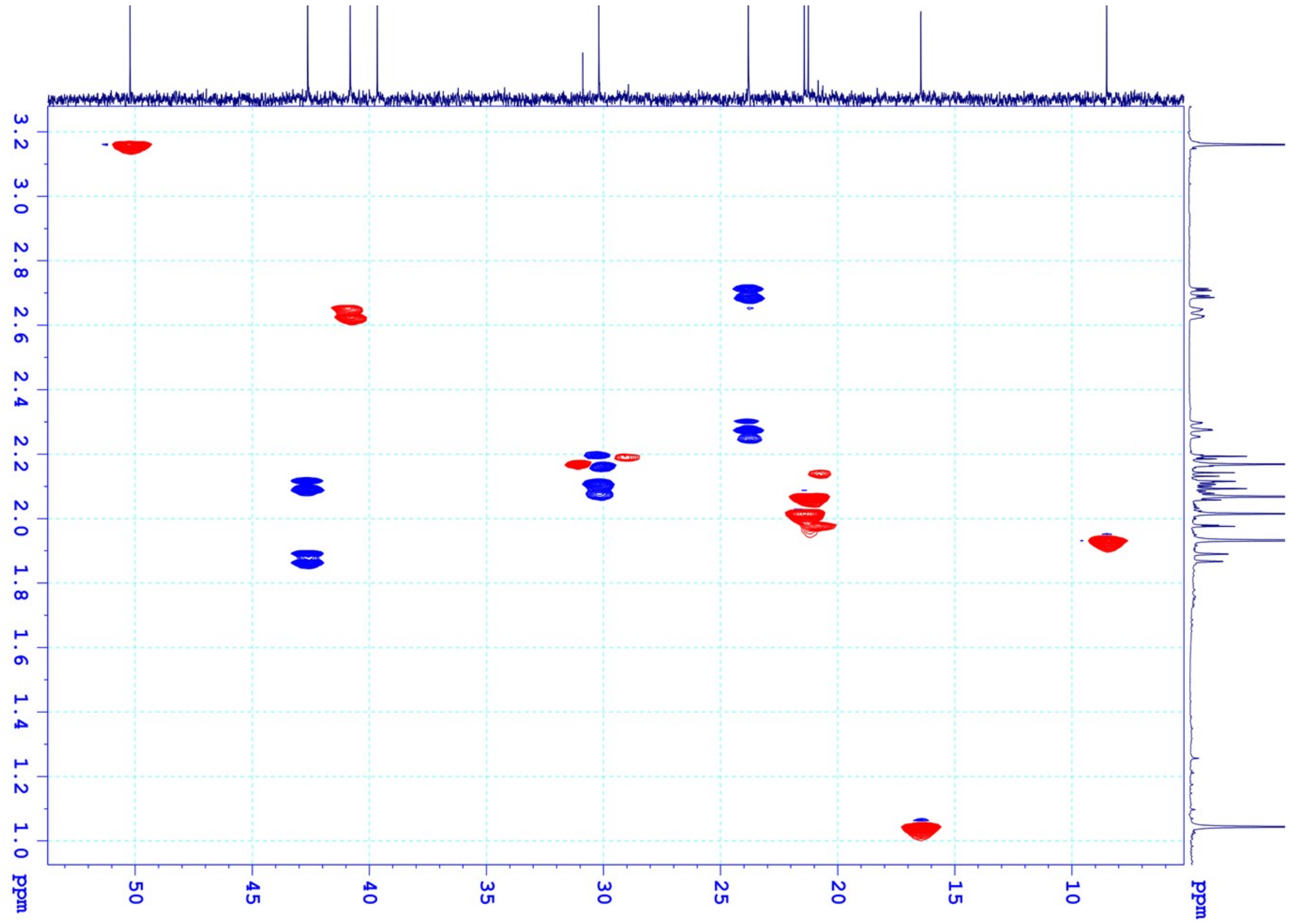

Figure S11. Expanded HSQC spectrum of compound 1

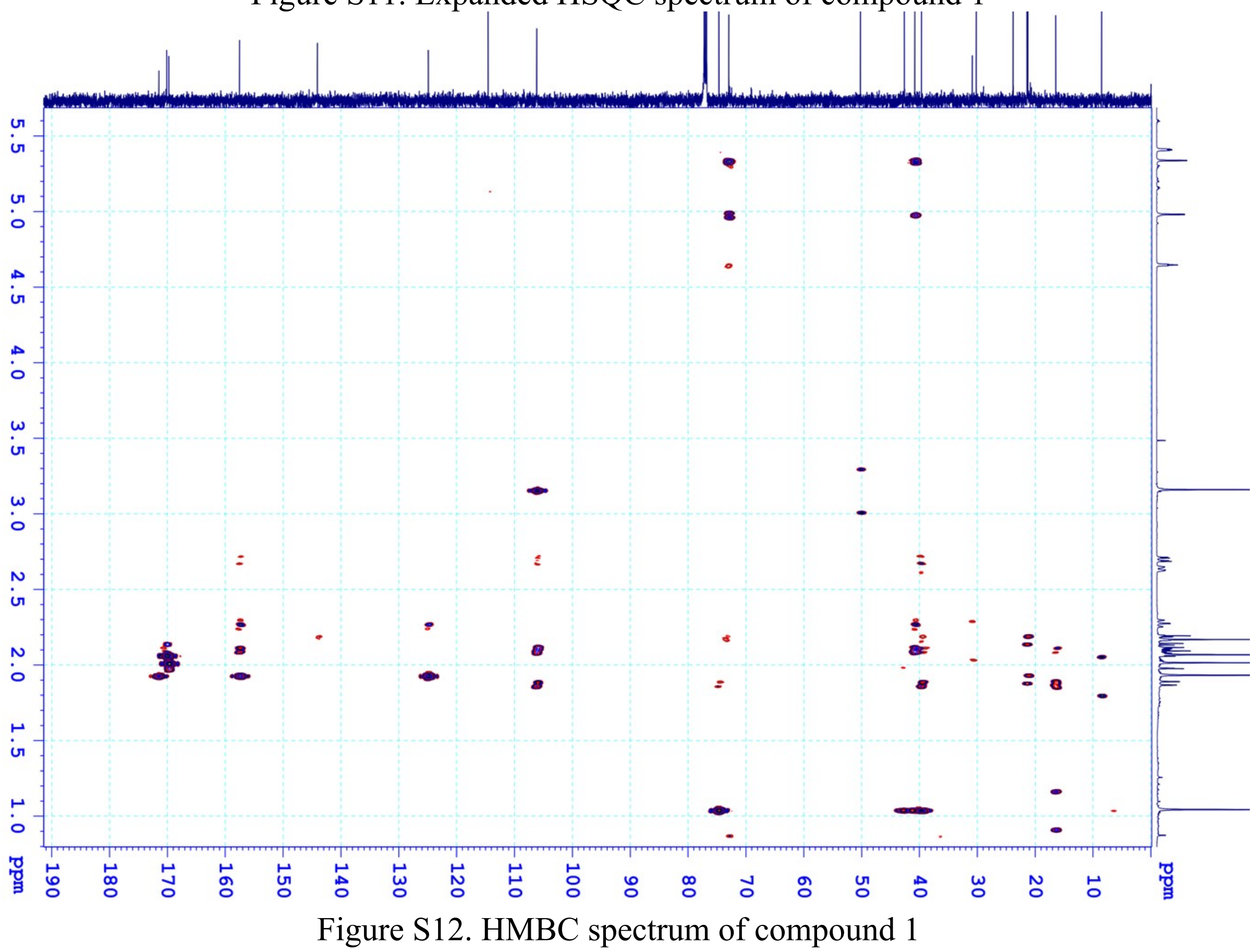

Figure S12. HMBC spectrum of compound 1

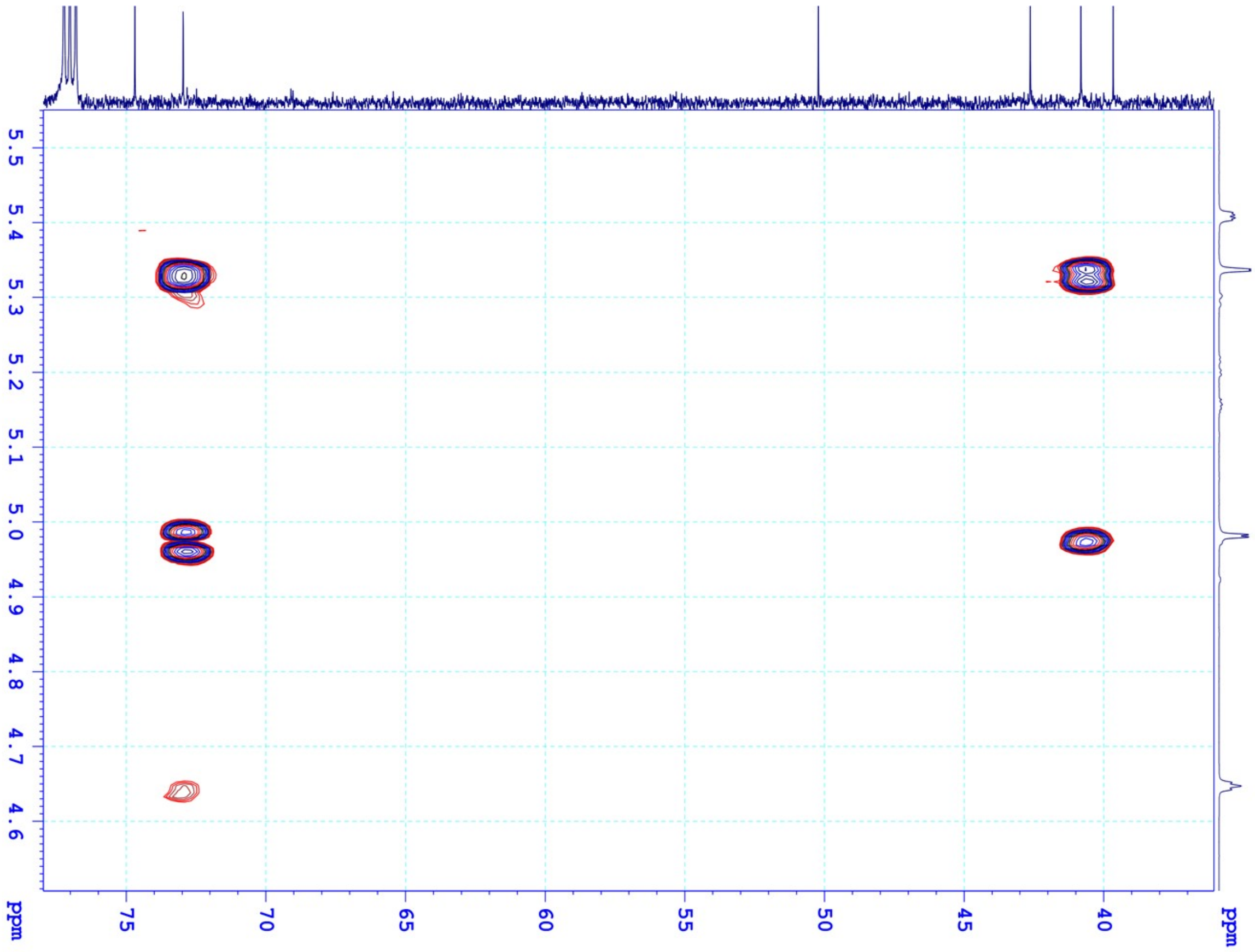

Figure S13. Expanded HMBC spectrum of compound 1

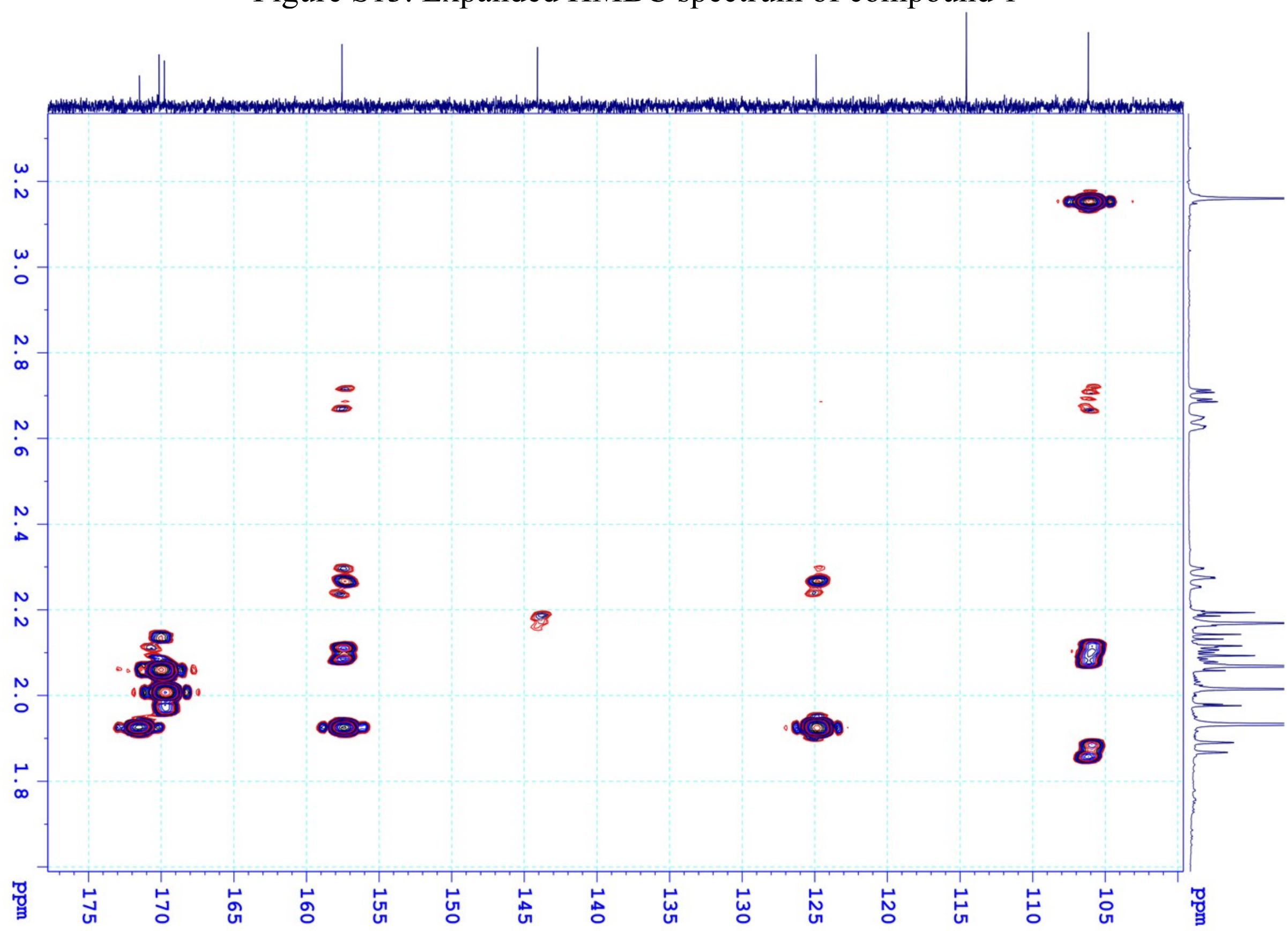

Figure S14. Expanded HMBC spectrum of compound 1

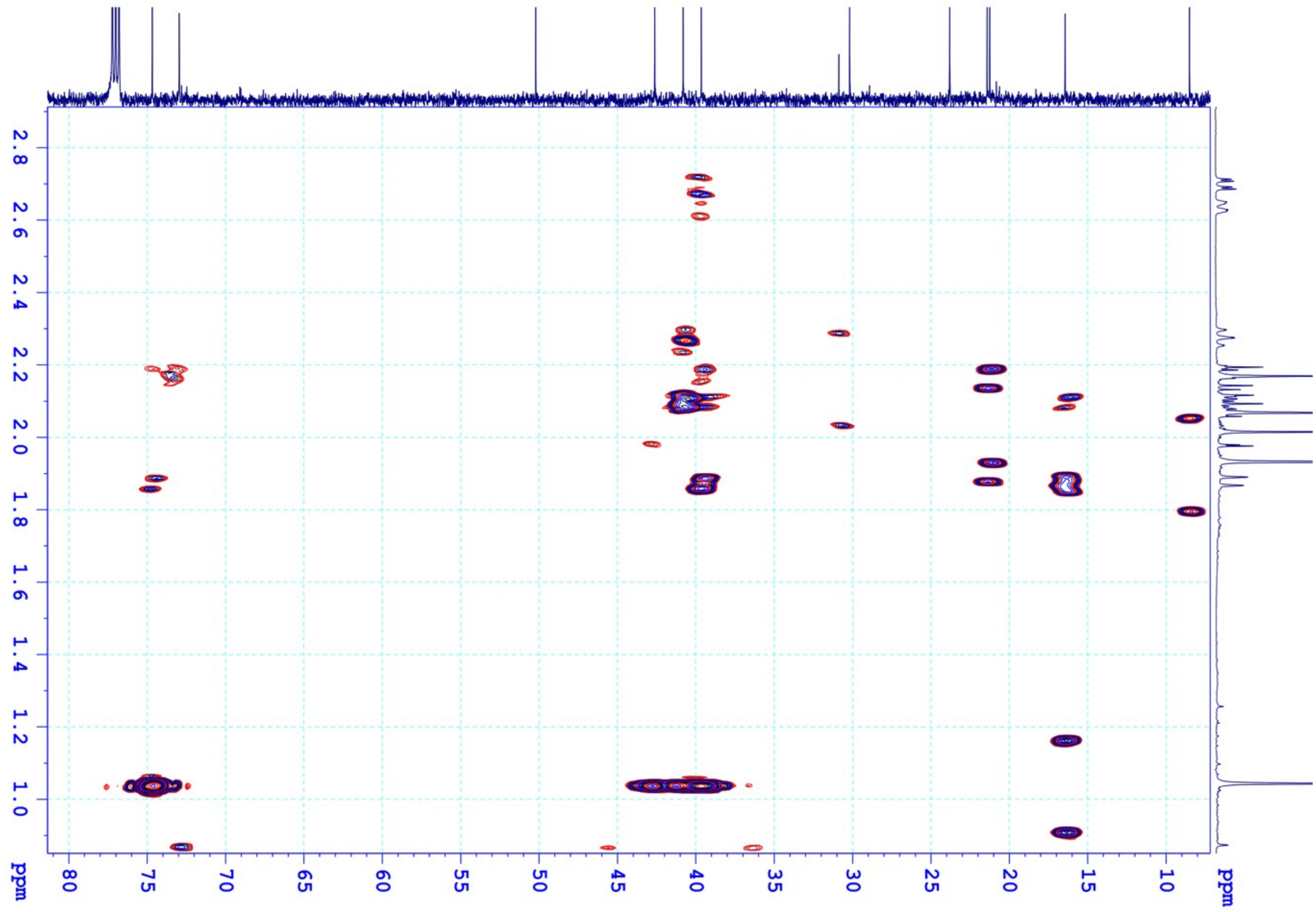

Figure S15. Expanded HMBC spectrum of compound 1

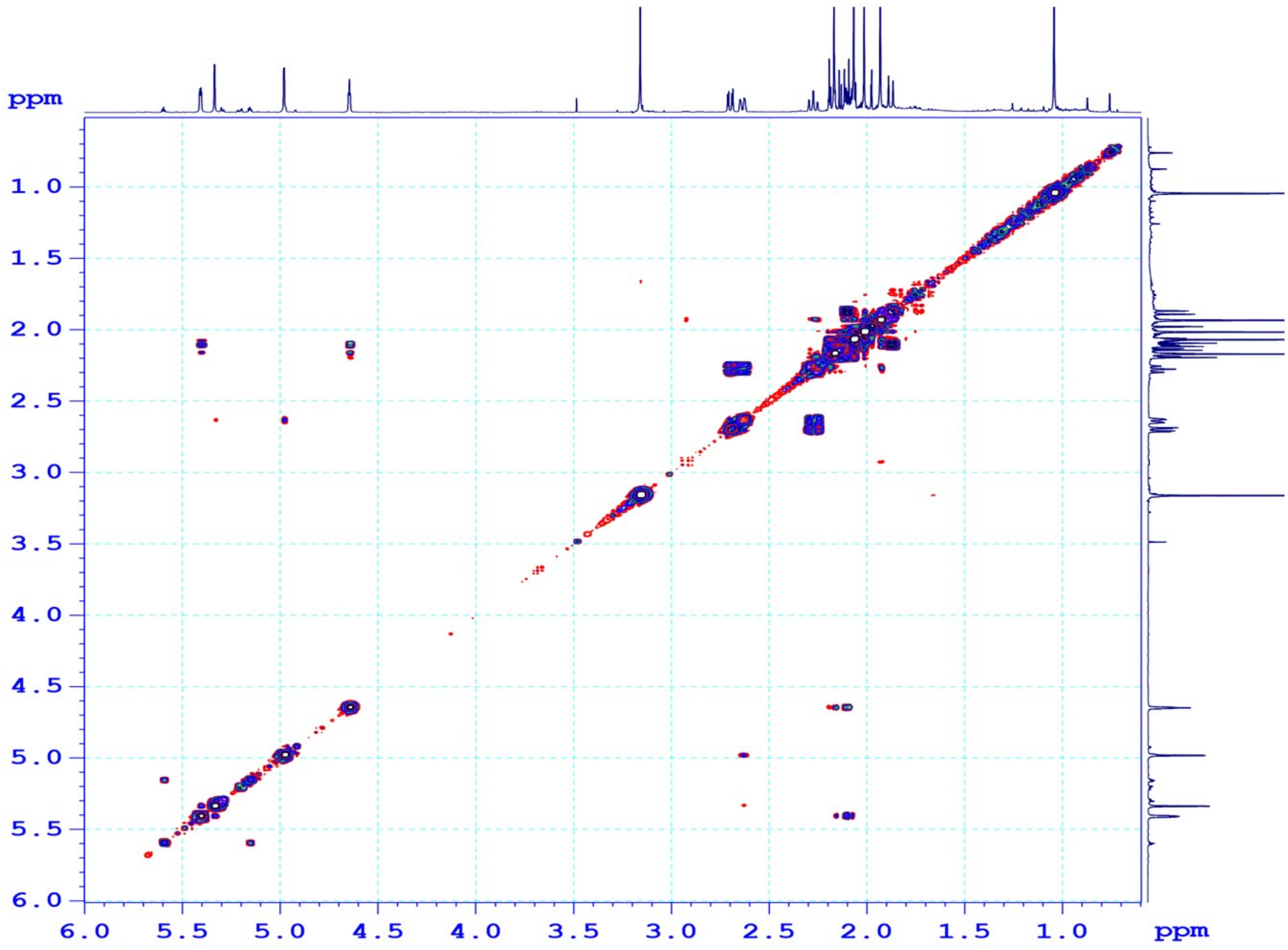

Figure S16. COSY spectrum of compound 1

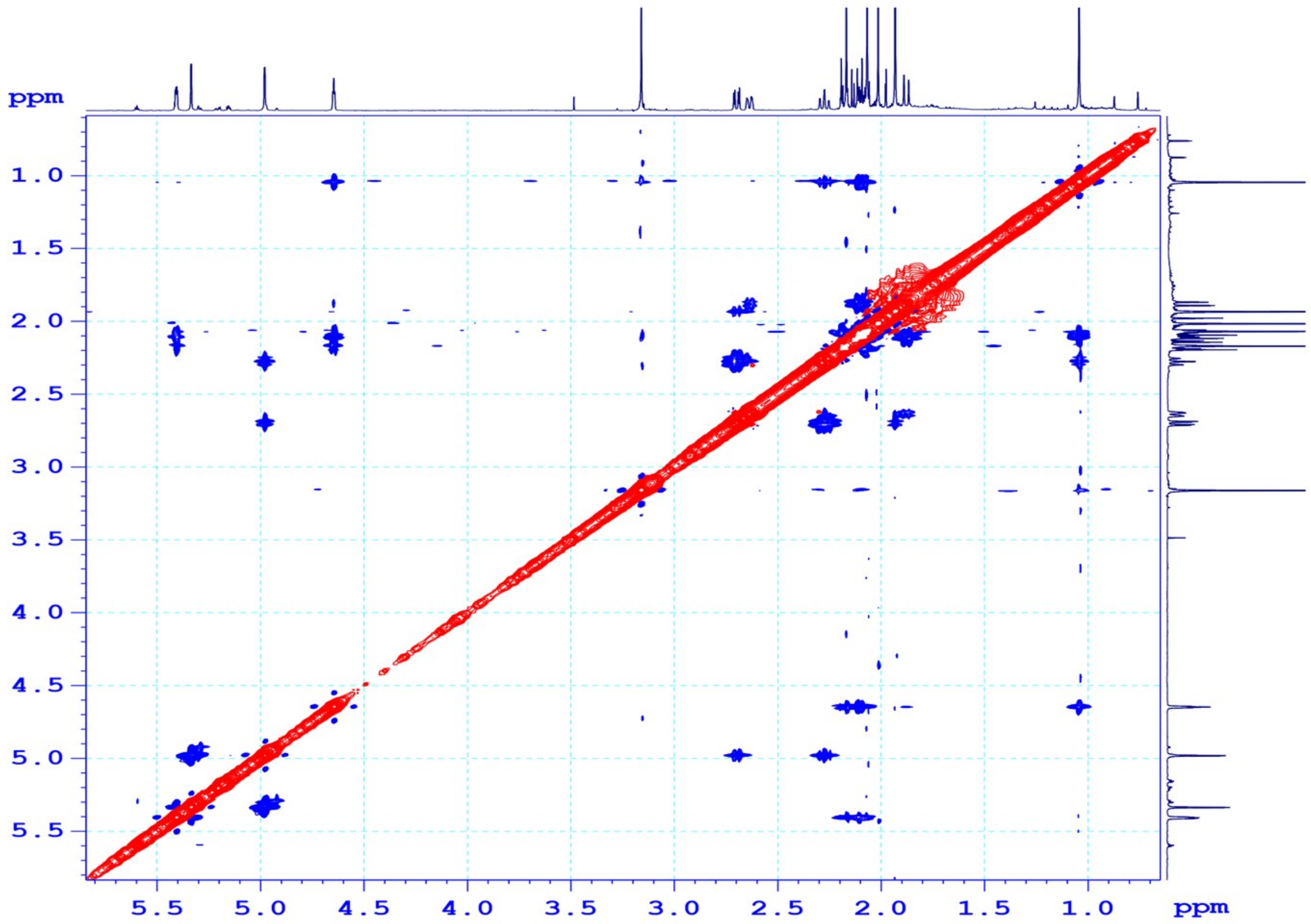

Figure S17. ROESY spectrum of compound 1

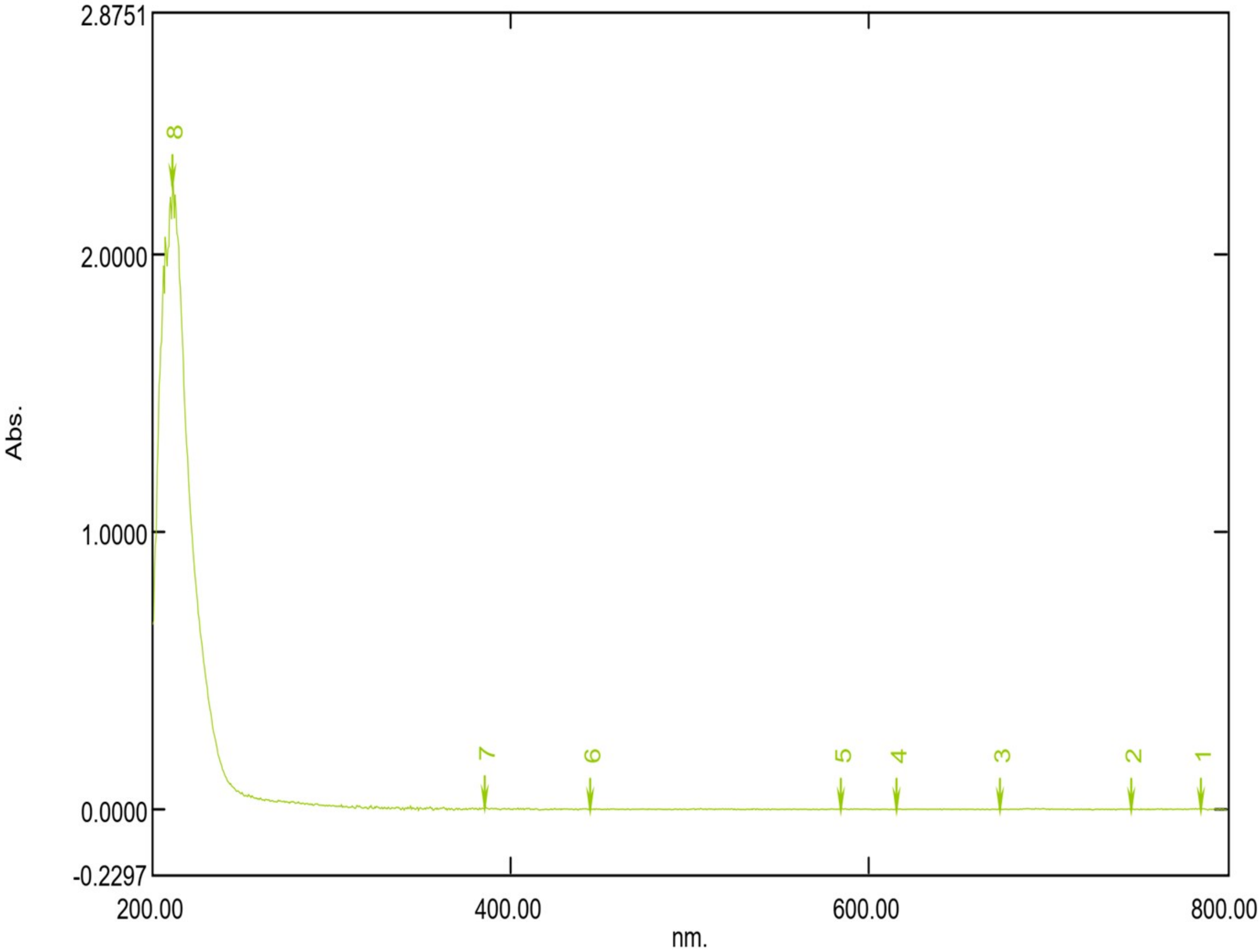

Figure S18. UV spectrum of compound 1

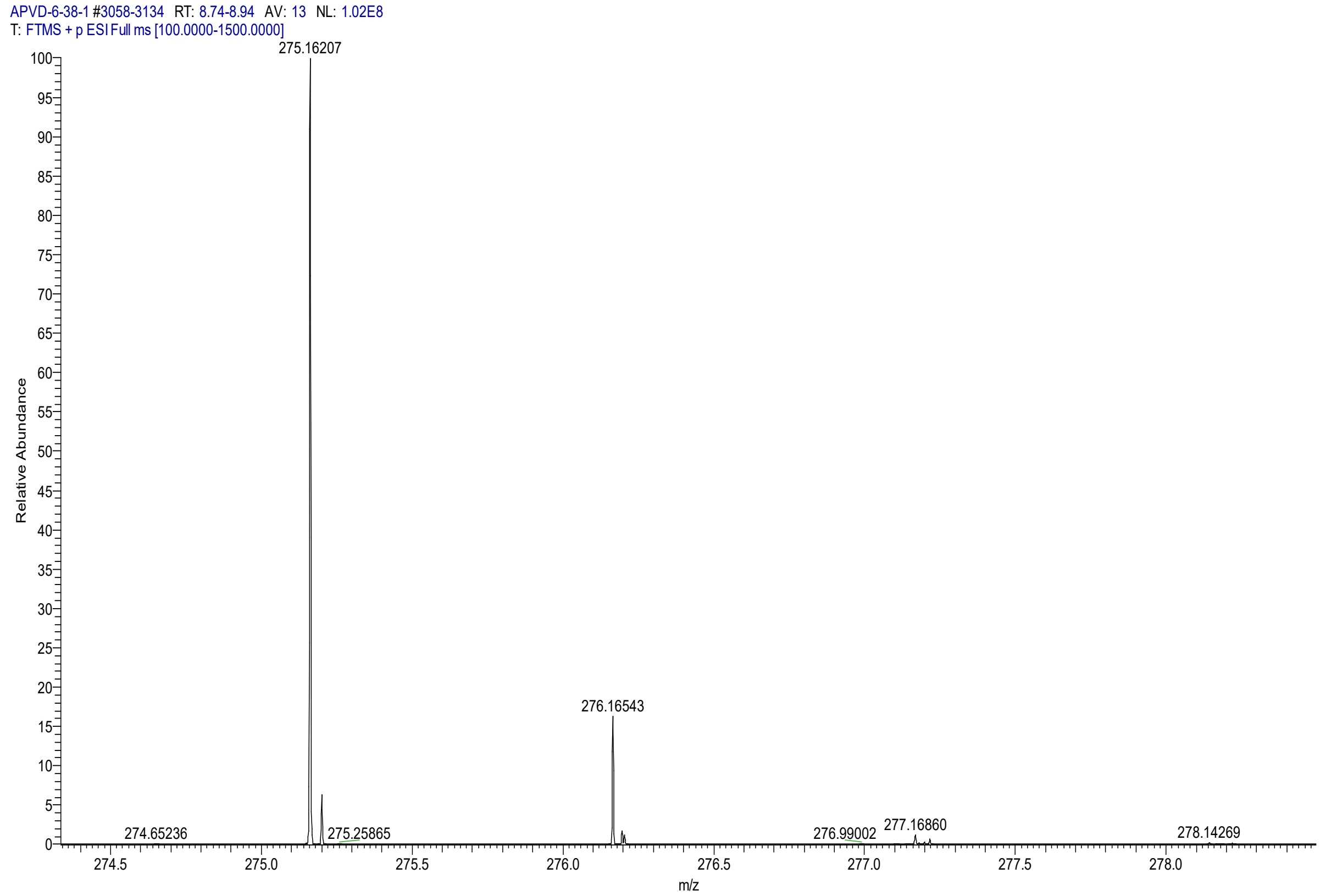

Figure S19. HR-ESI-MS of compound 2

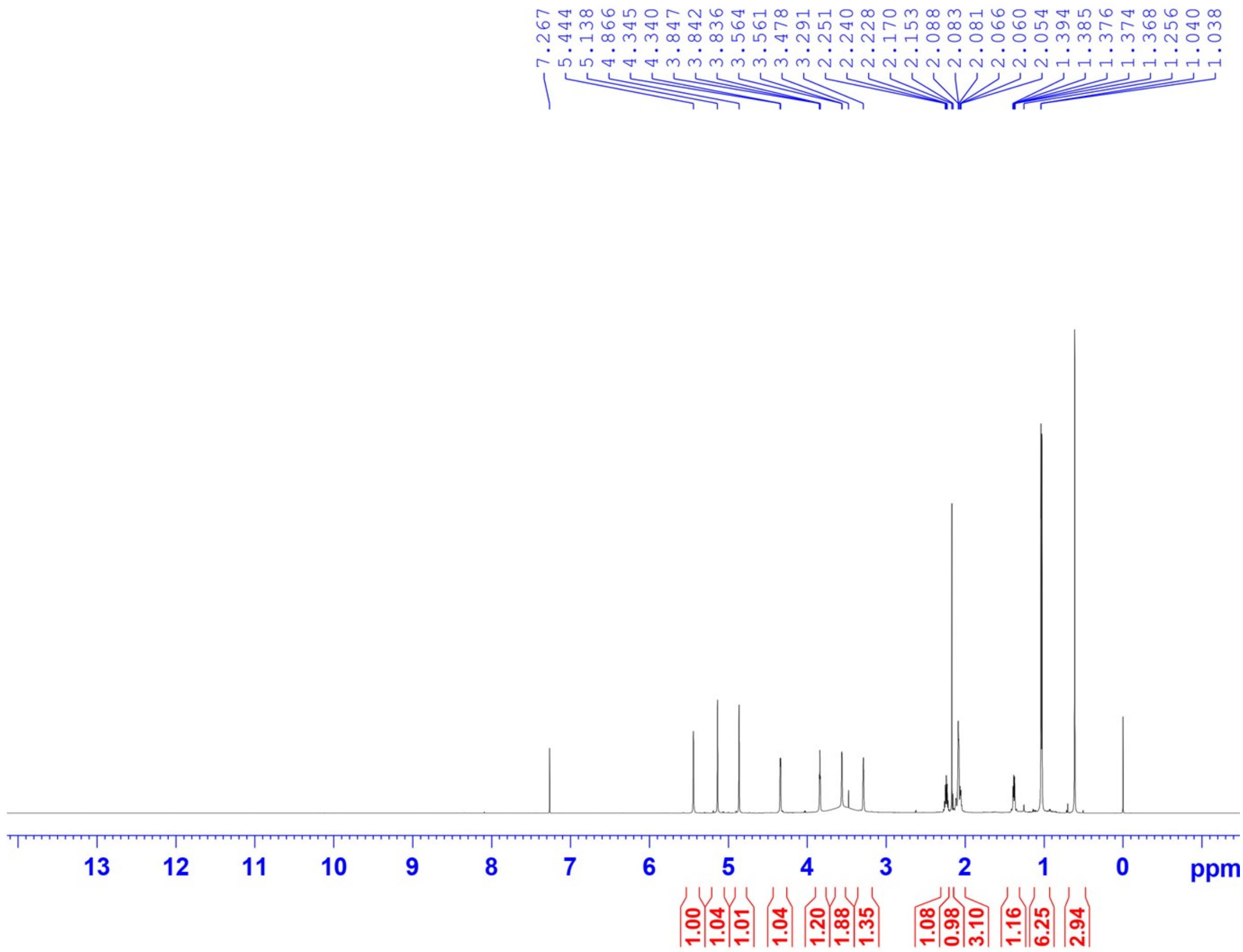

Figure S20. 1H-NMR spectrum of compound 2 in CDCl3

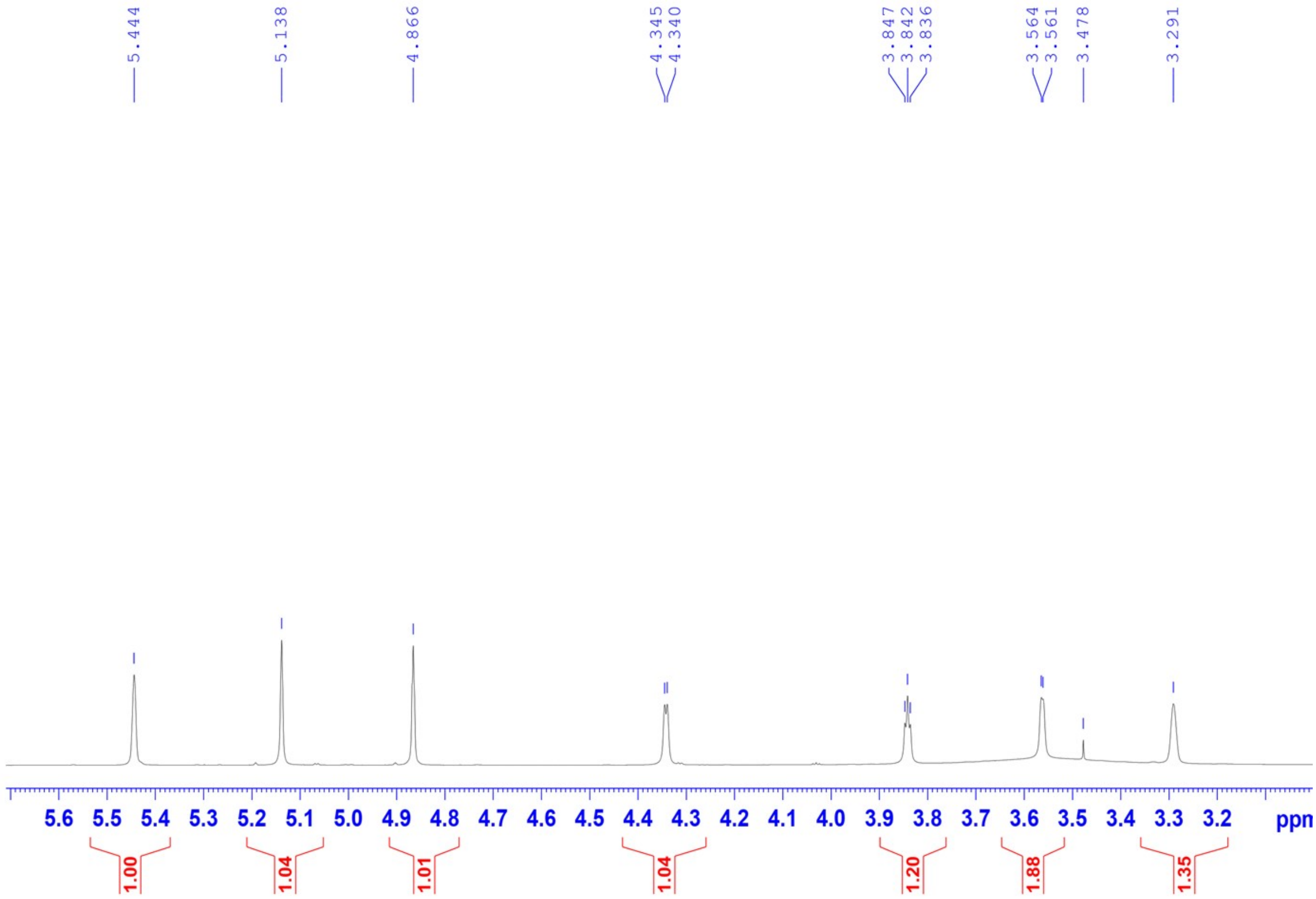

Figure S21. Expanded 1H-NMR spectrum of compound 2

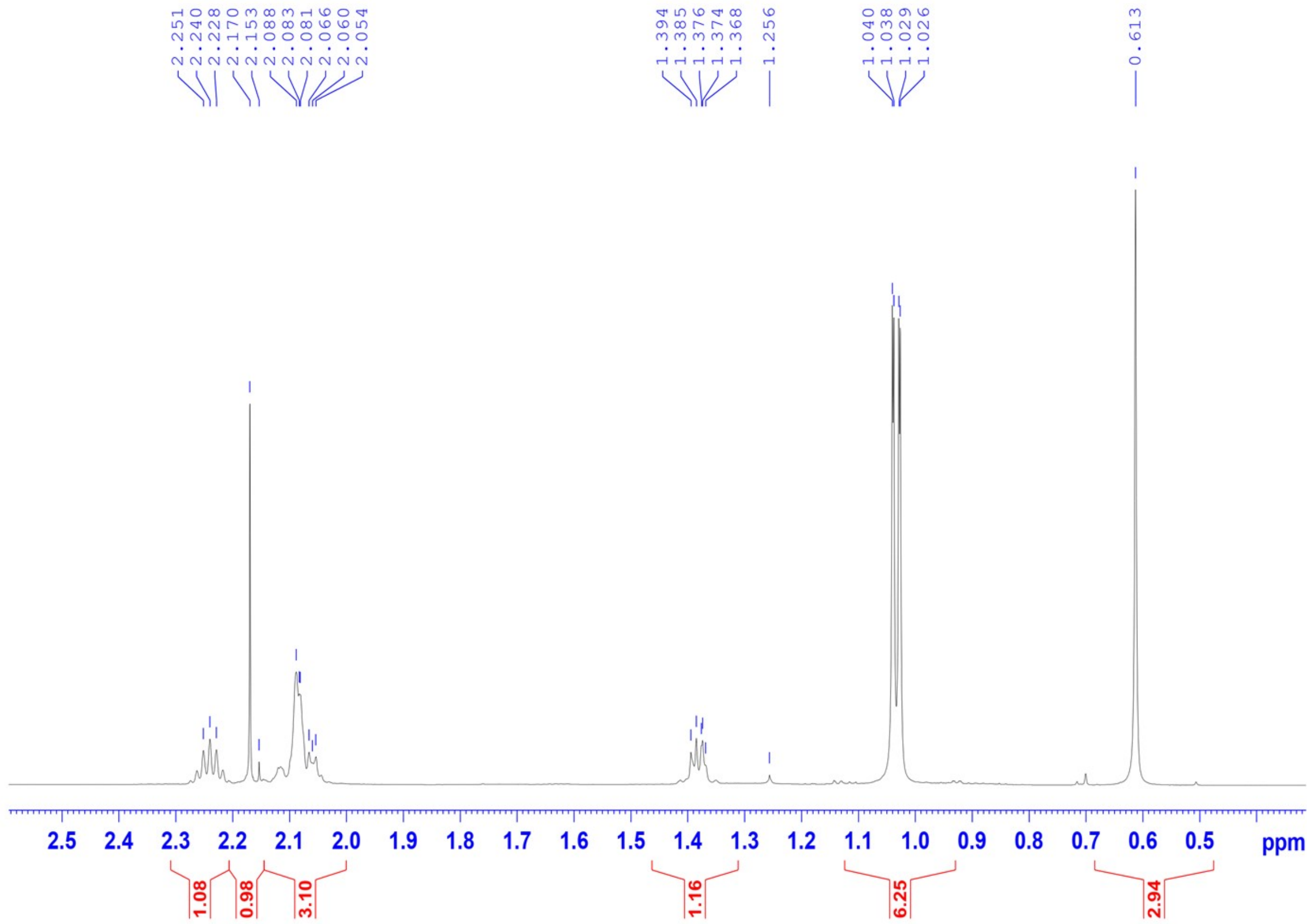

Figure S22. Expanded 1H-NMR spectrum of compound 2

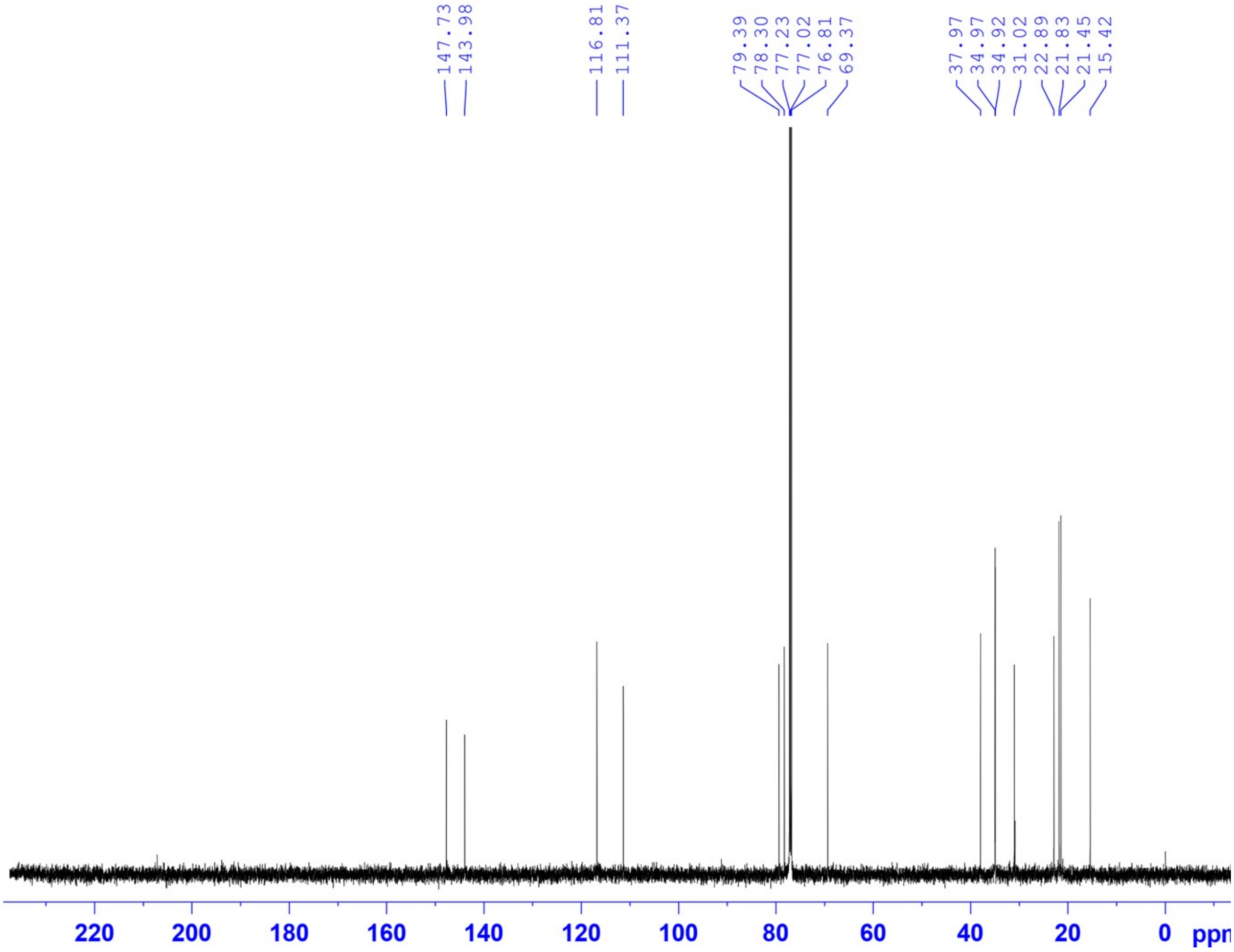

Figure S23. 13C-NMR spectrum of compound 2 in CDCl3

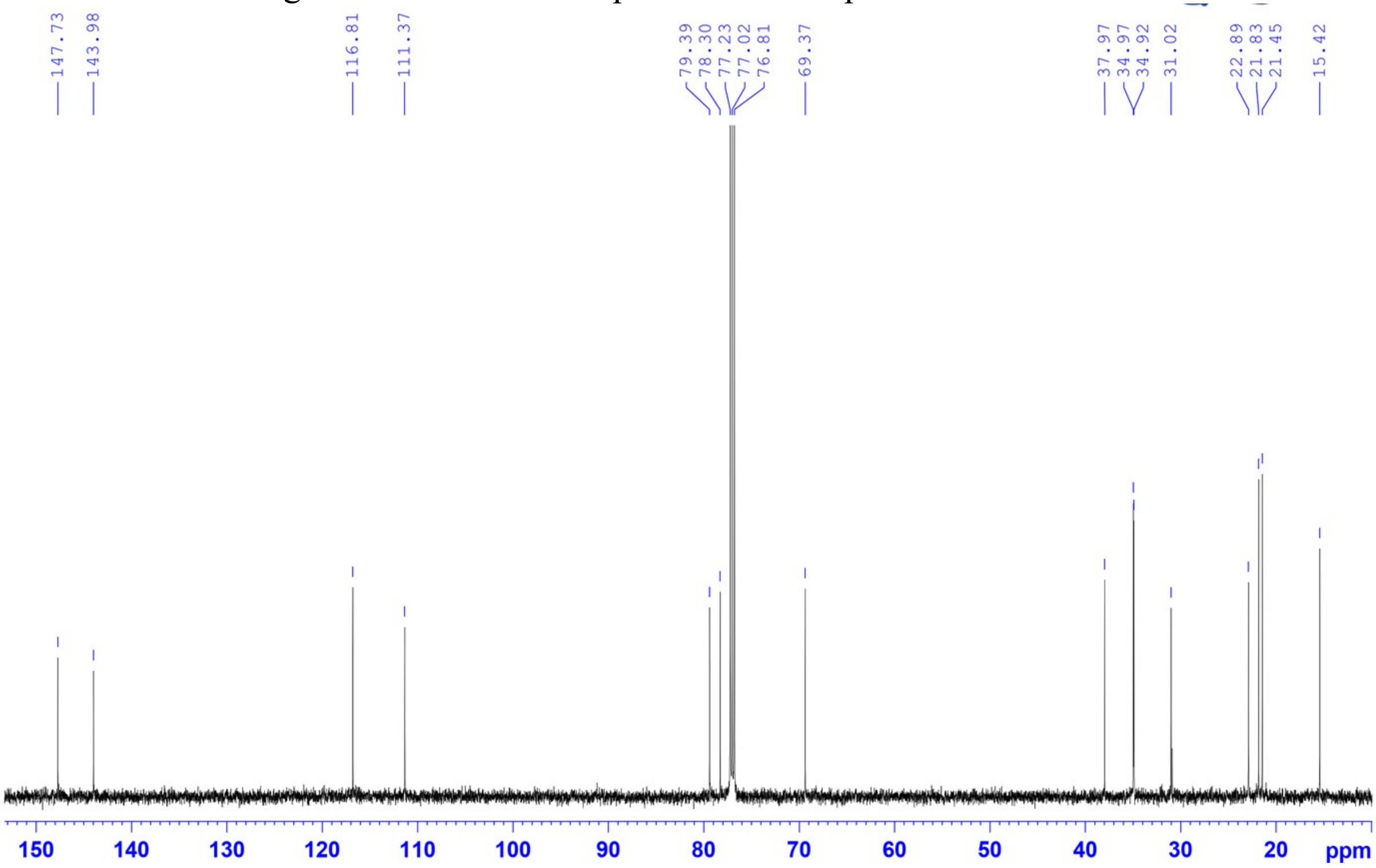

Figure S24. Expanded 13C-NMR spectrum of compound 2

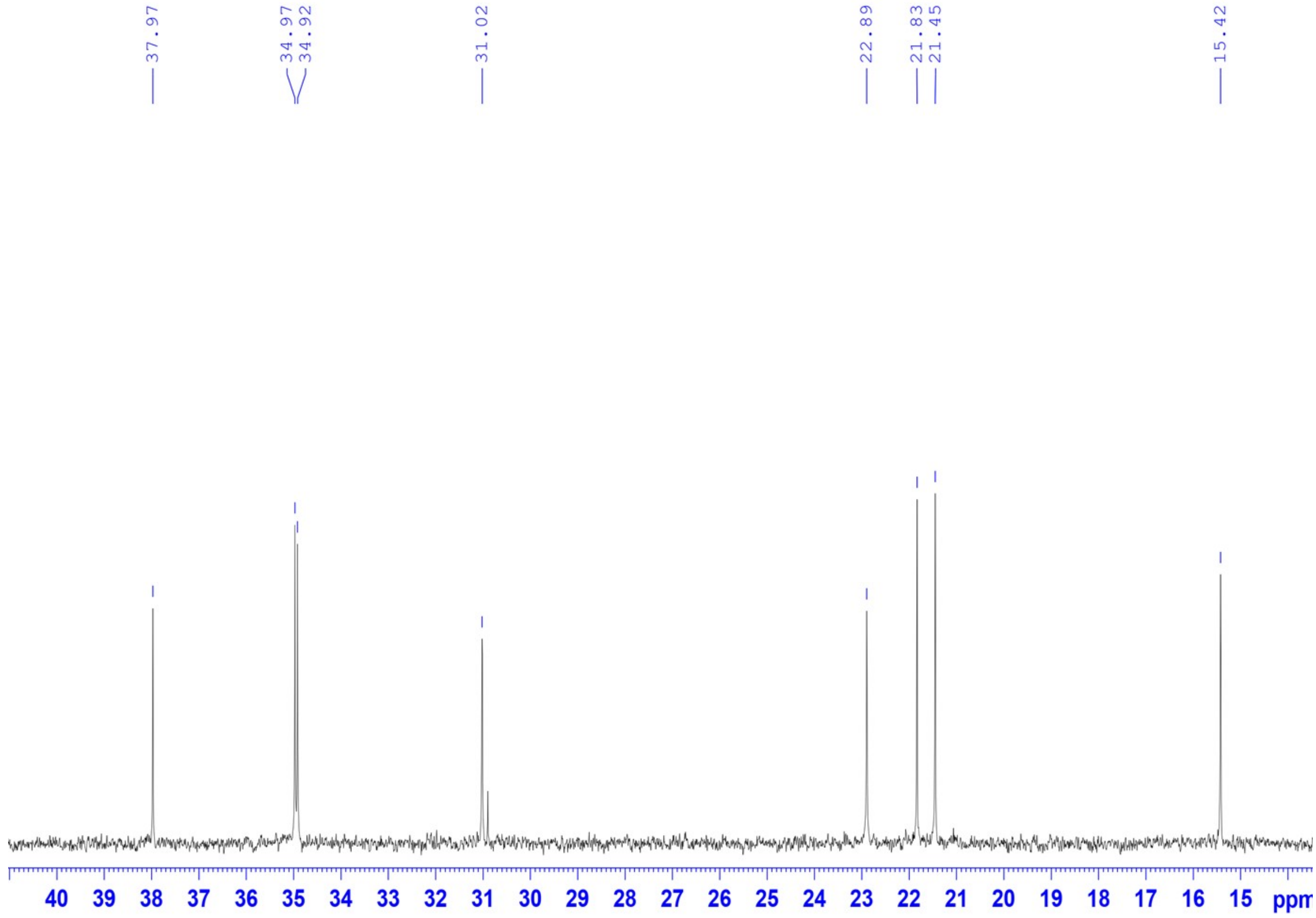

Figure S25. Expanded 13C-NMR spectrum of compound 2

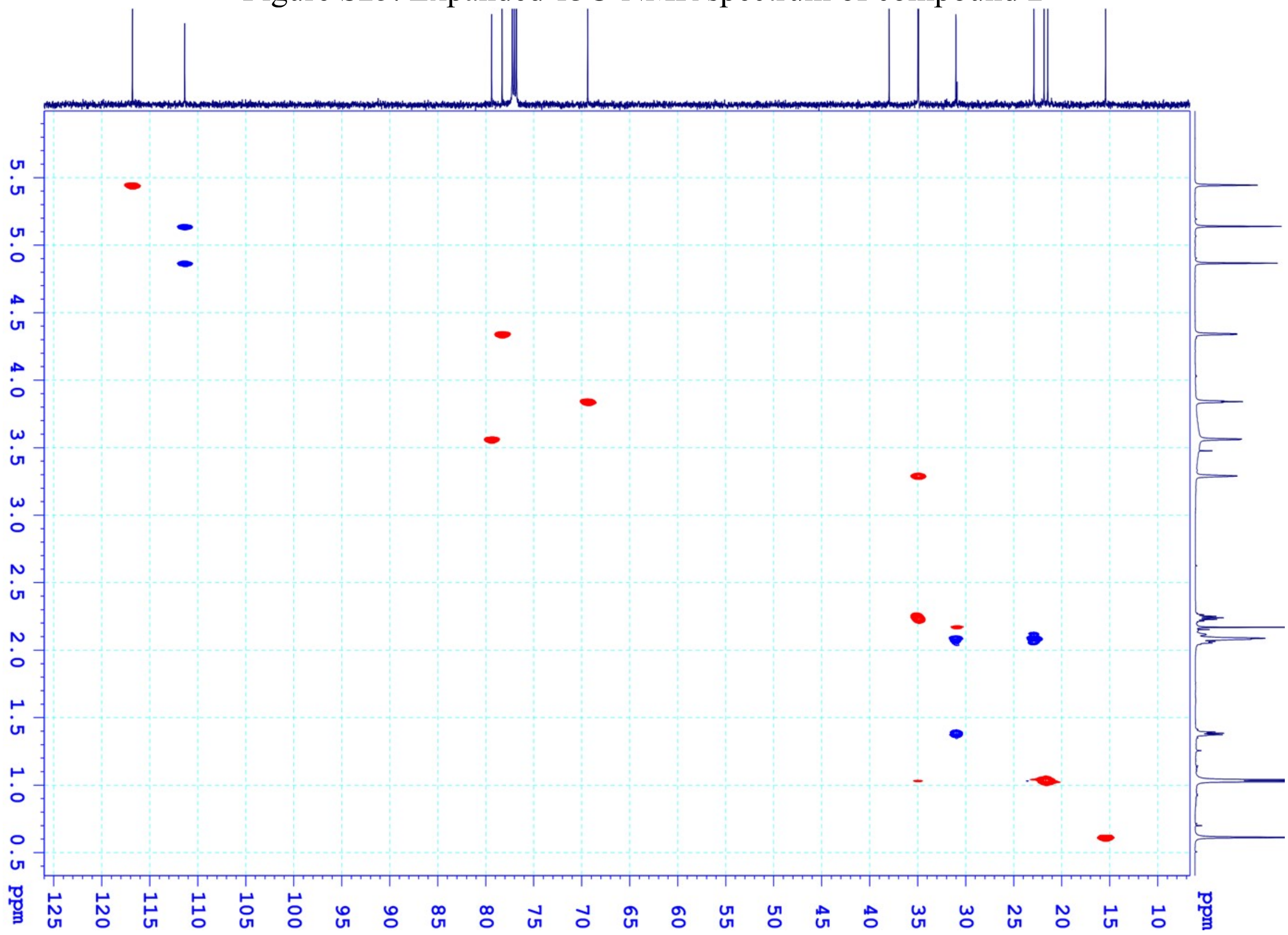

Figure S26. HSQC spectrum of compound 2

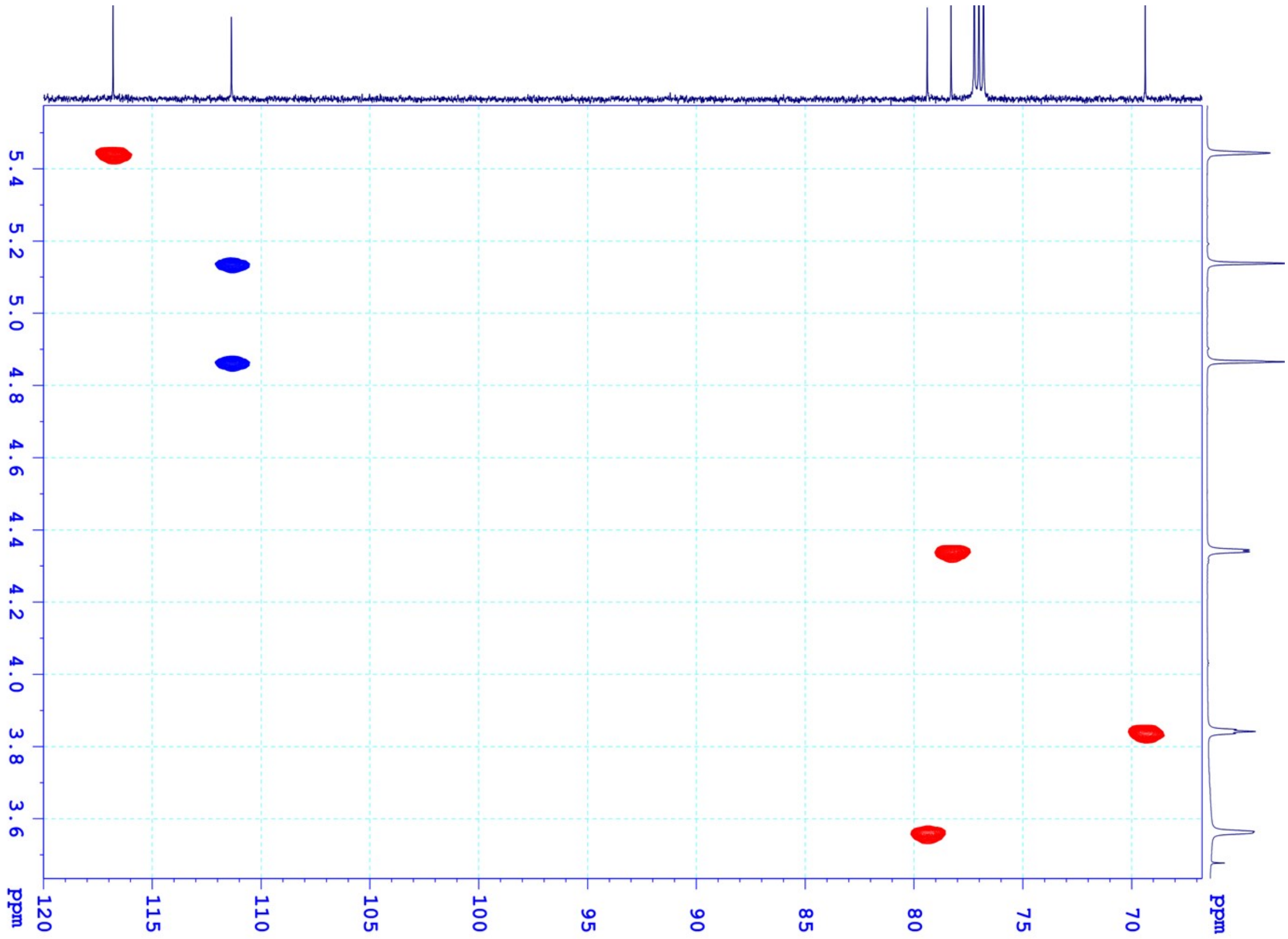

Figure S27. Expanded HSQC spectrum of compound 2

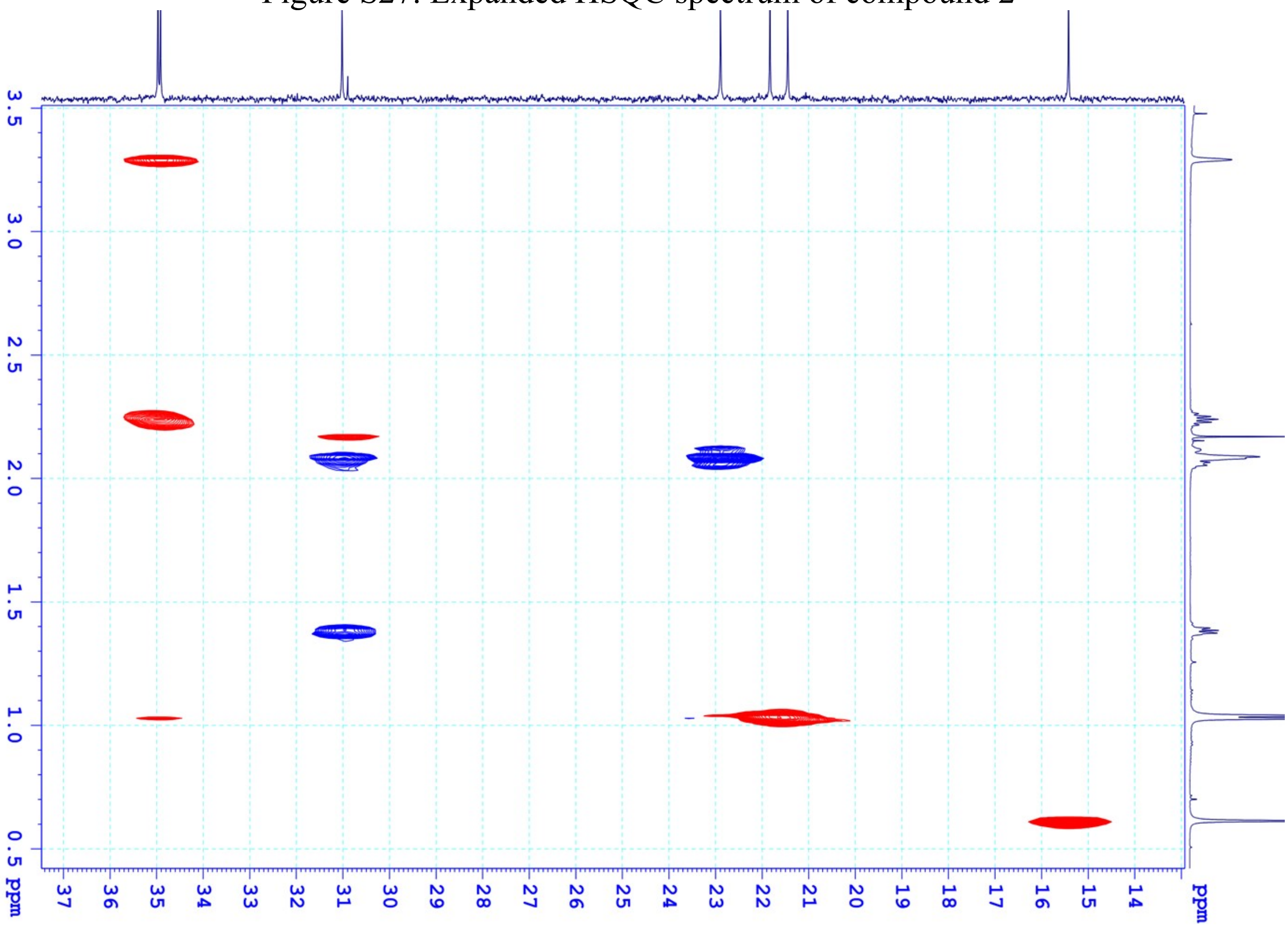

Figure S28. Expanded HSQC spectrum of compound 2

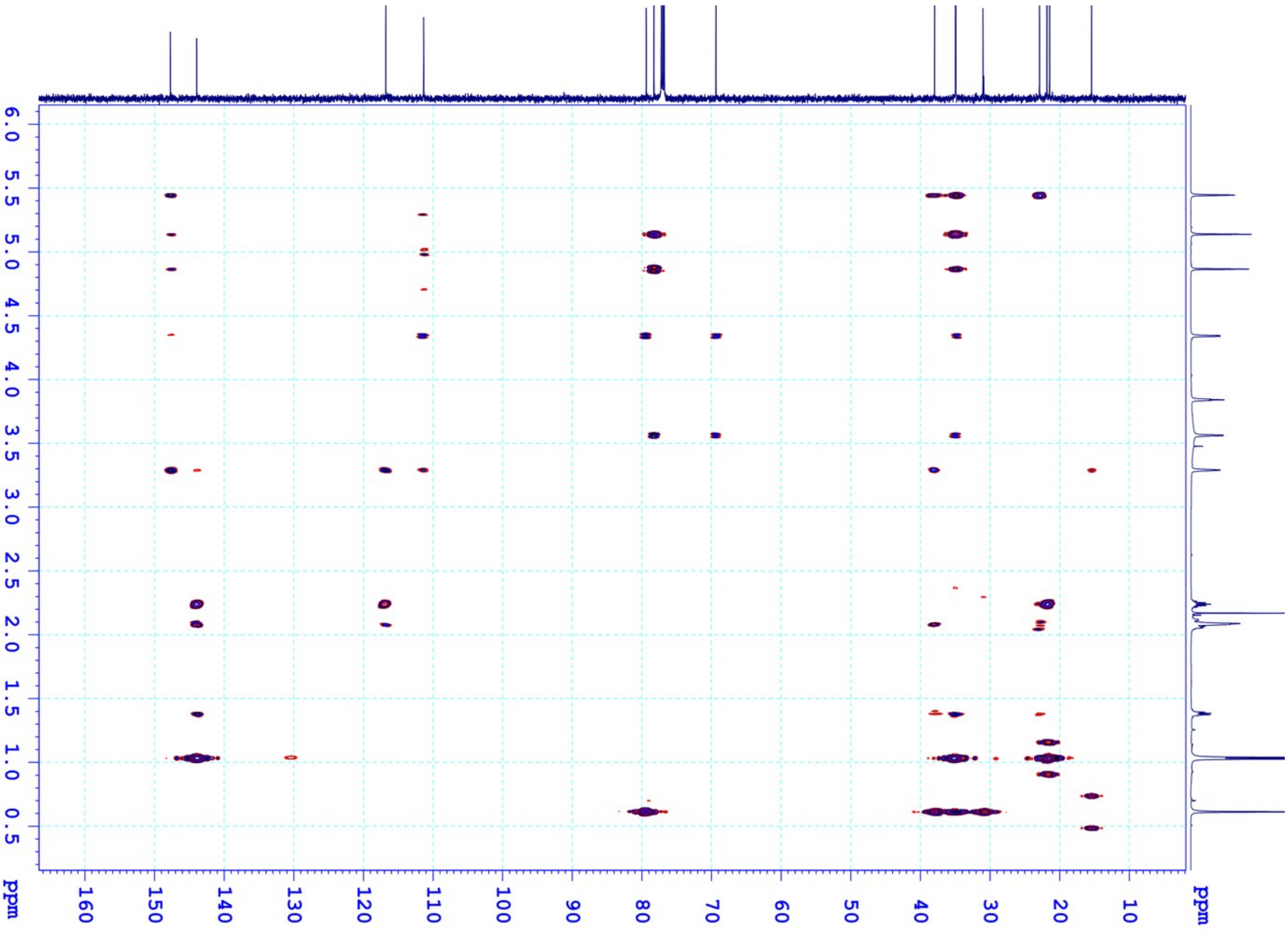

Figure S29. HMBC spectrum of compound 2

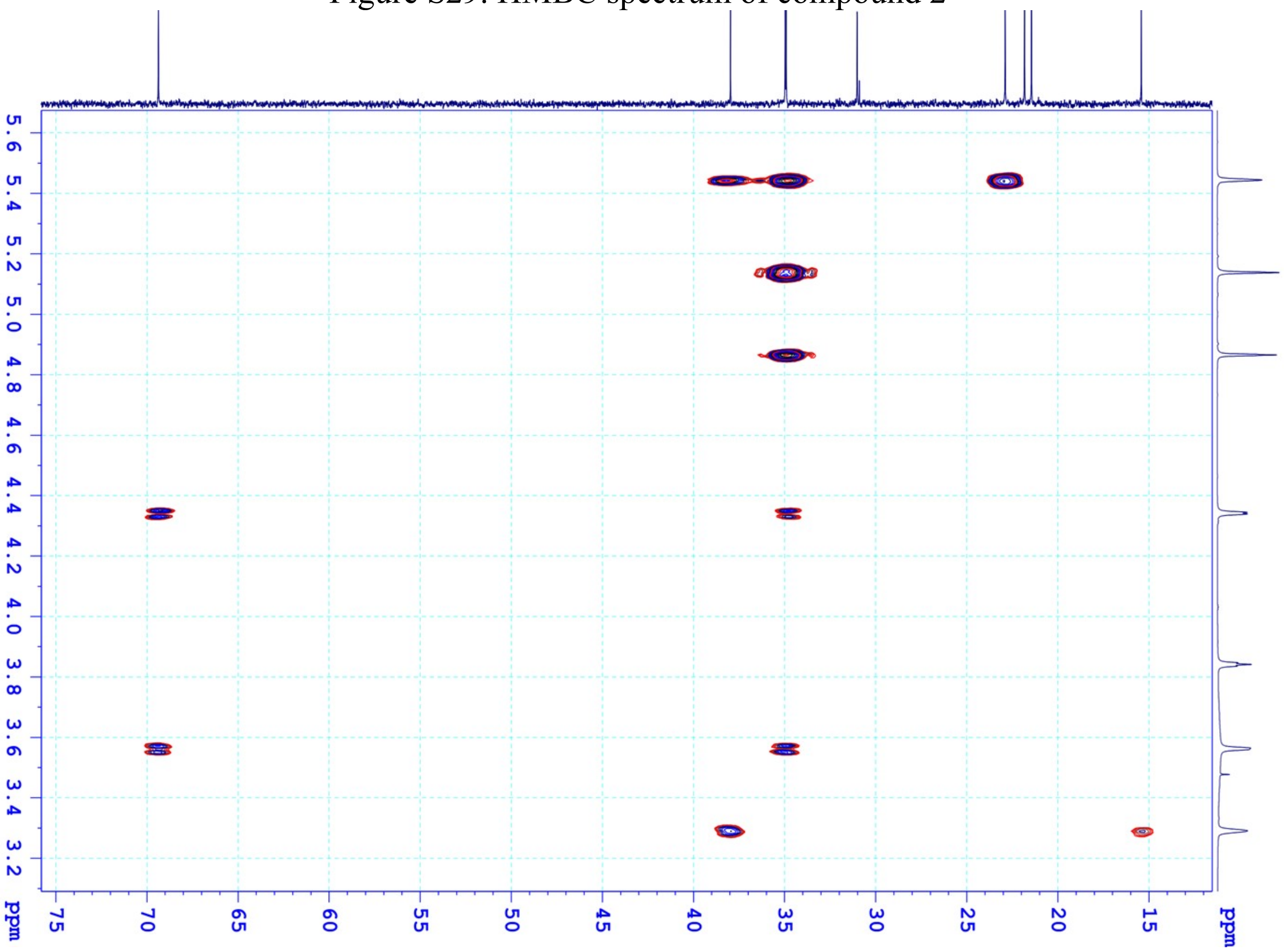

Figure S30. Expanded HMBC spectrum of compound 2

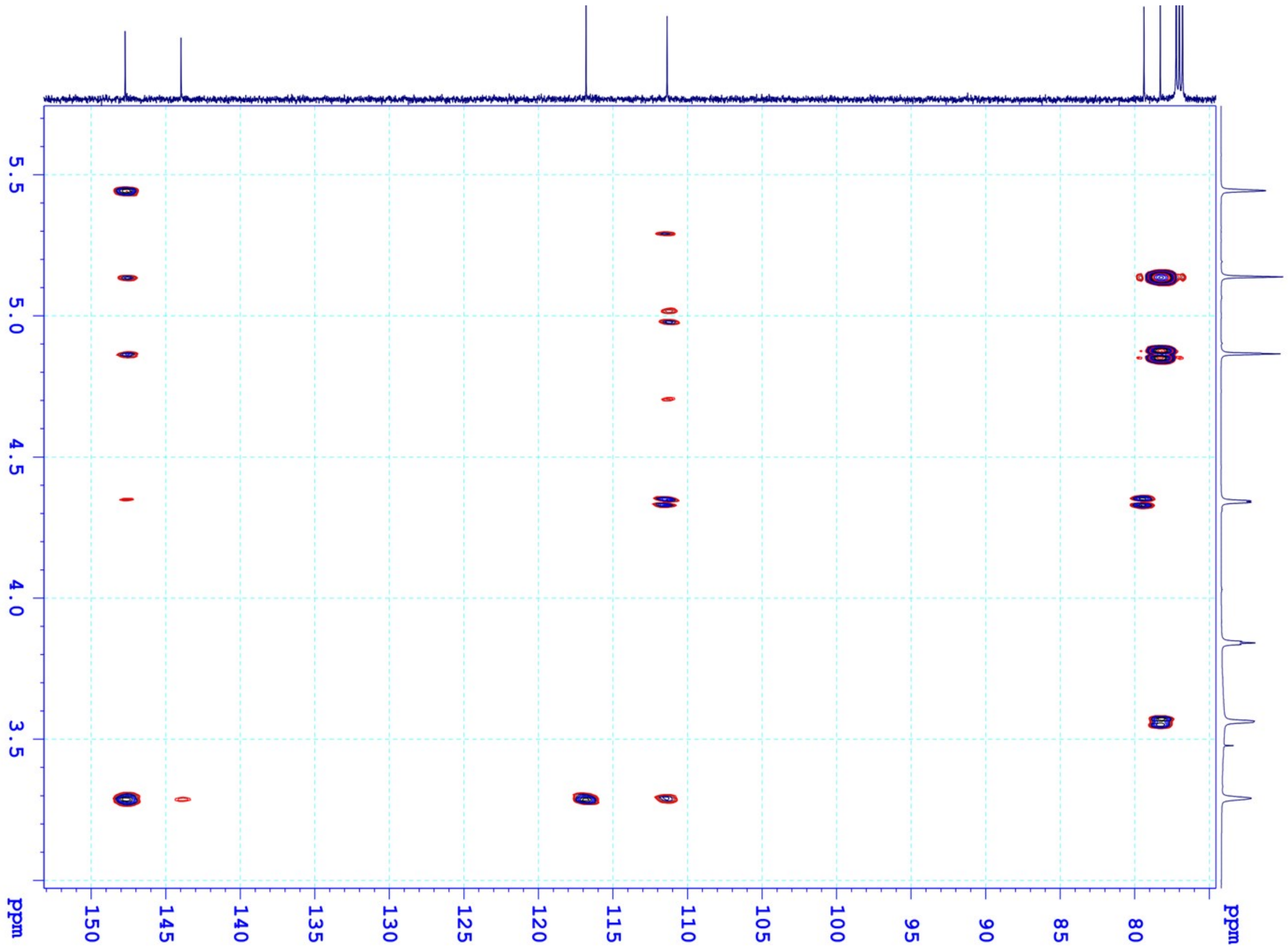

Figure S31. Expanded HMBC spectrum of compound 2

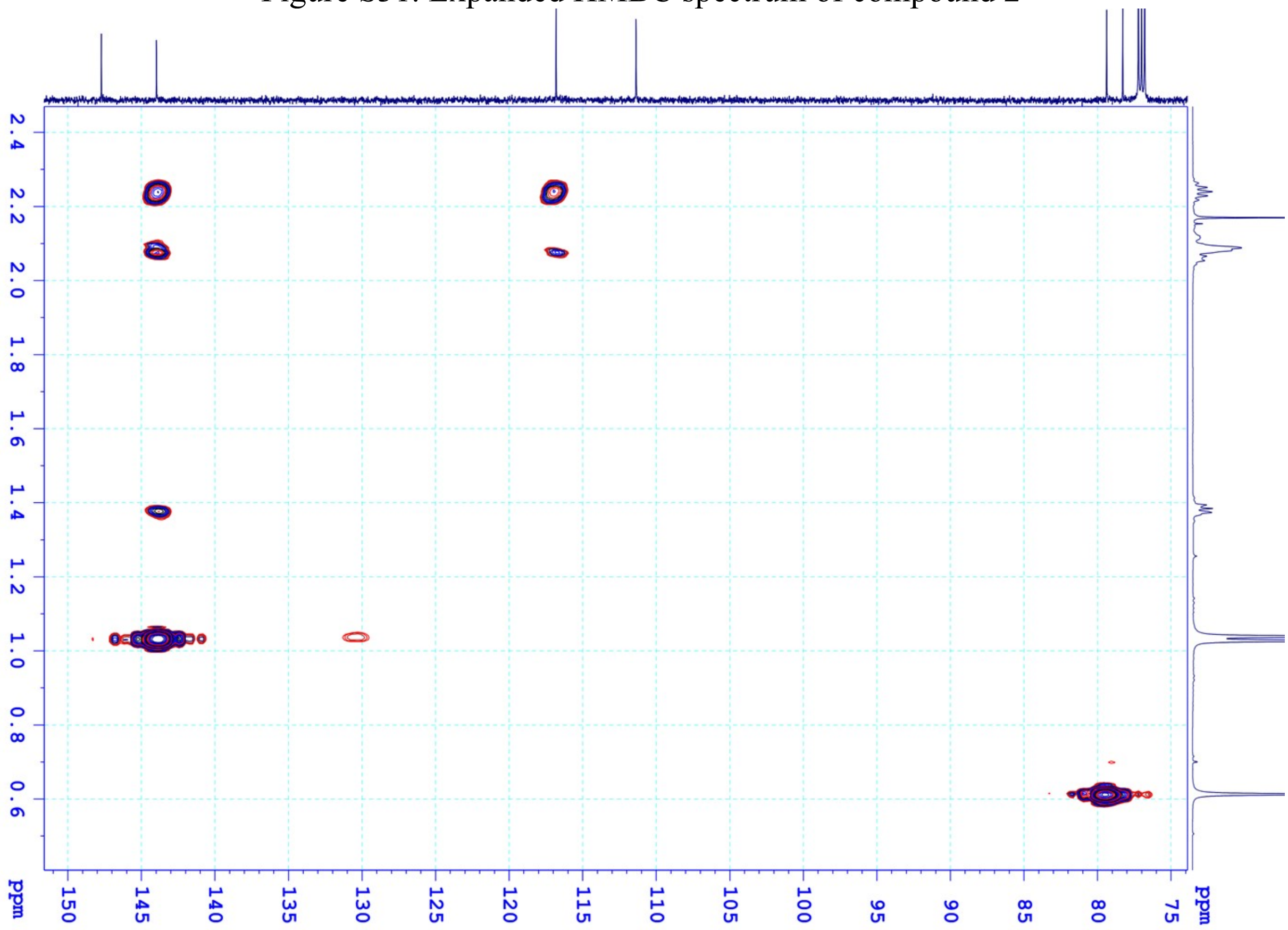

Figure S32. Expanded HMBC spectrum of compound 2

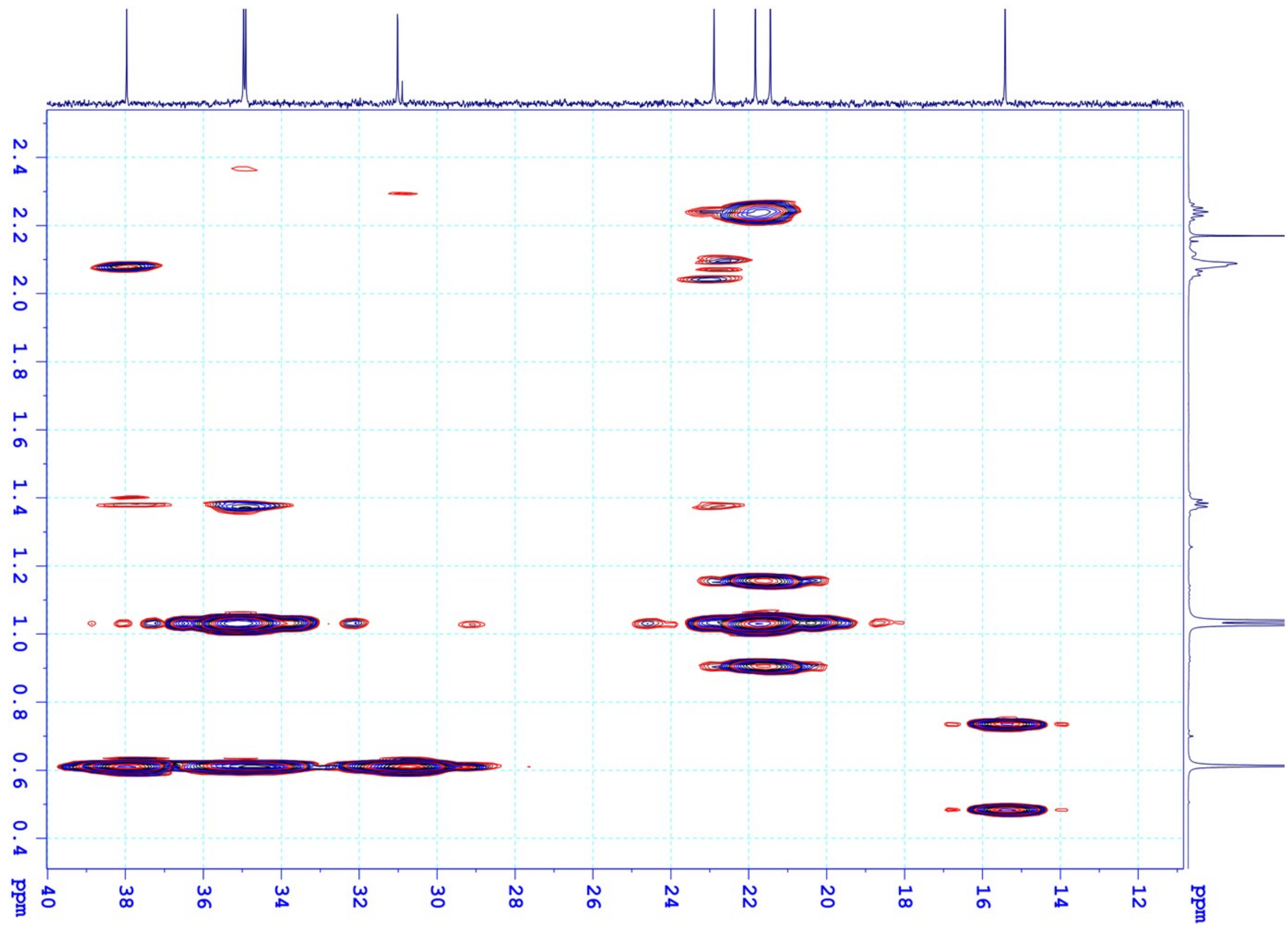

Figure S33. Expanded HMBC spectrum of compound 2

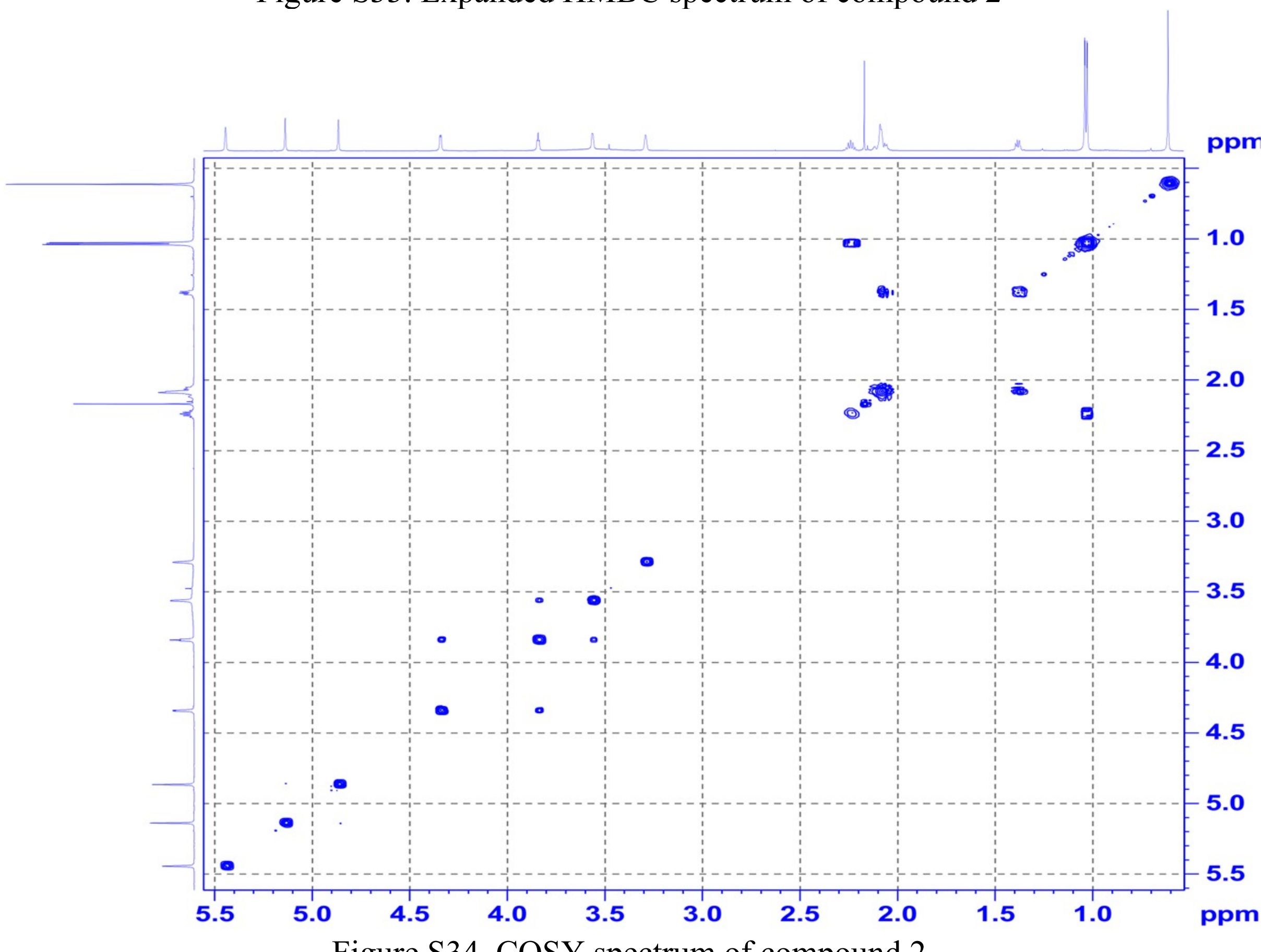

Figure S34. COSY spectrum of compound 2

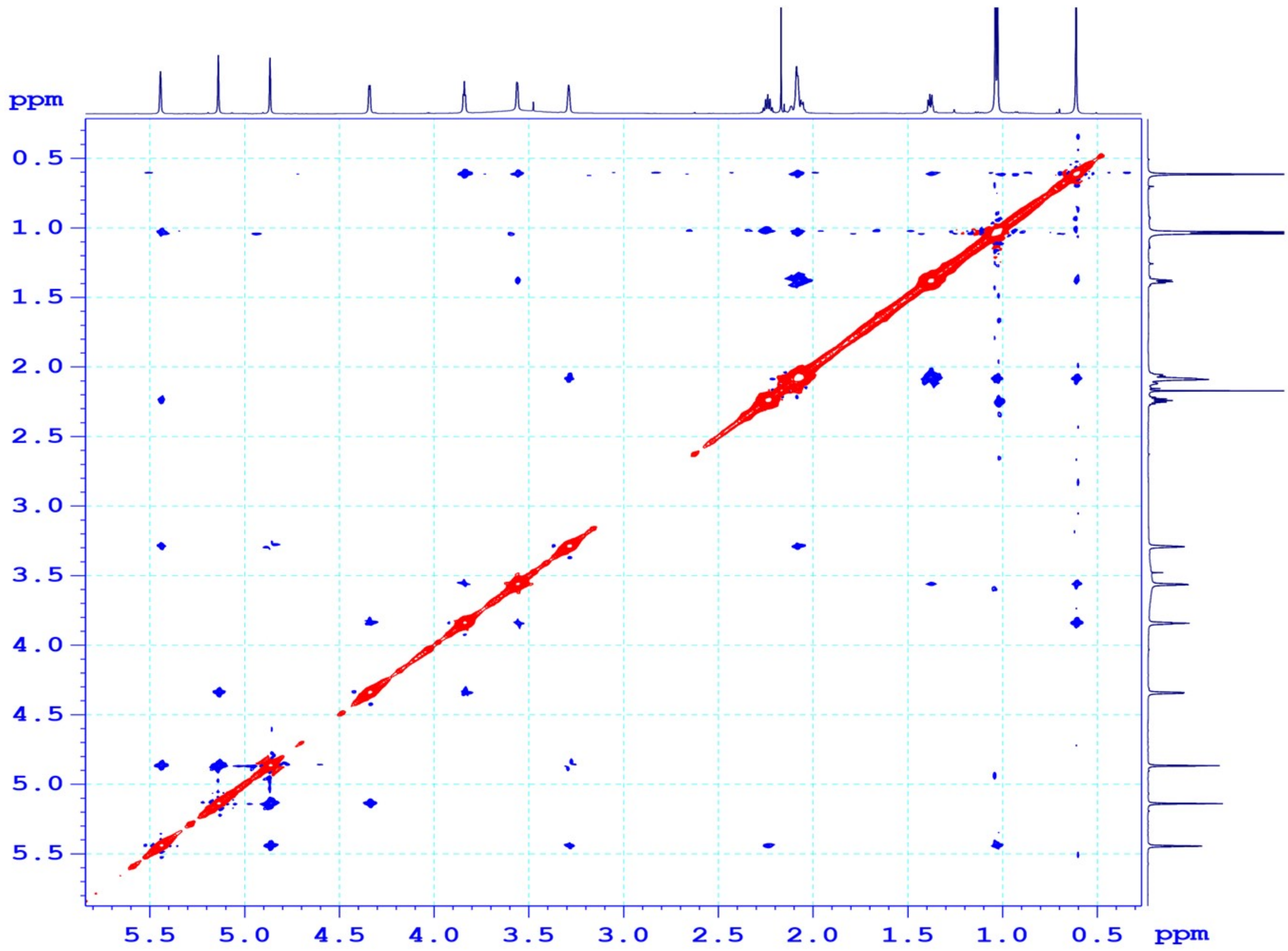

Figure S35. ROESY spectrum of compound 2

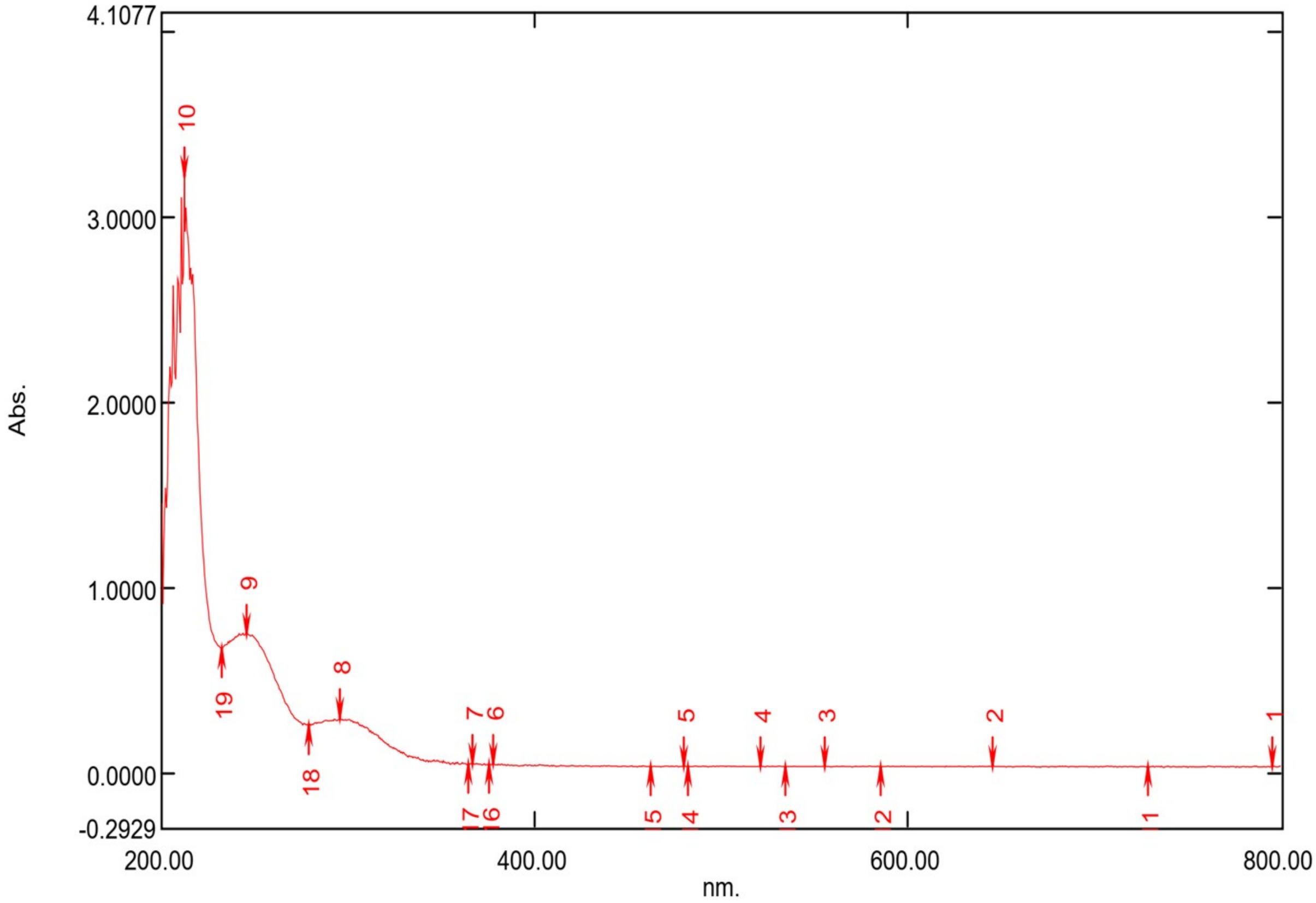

Figure S36. UV spectrum of compound 2

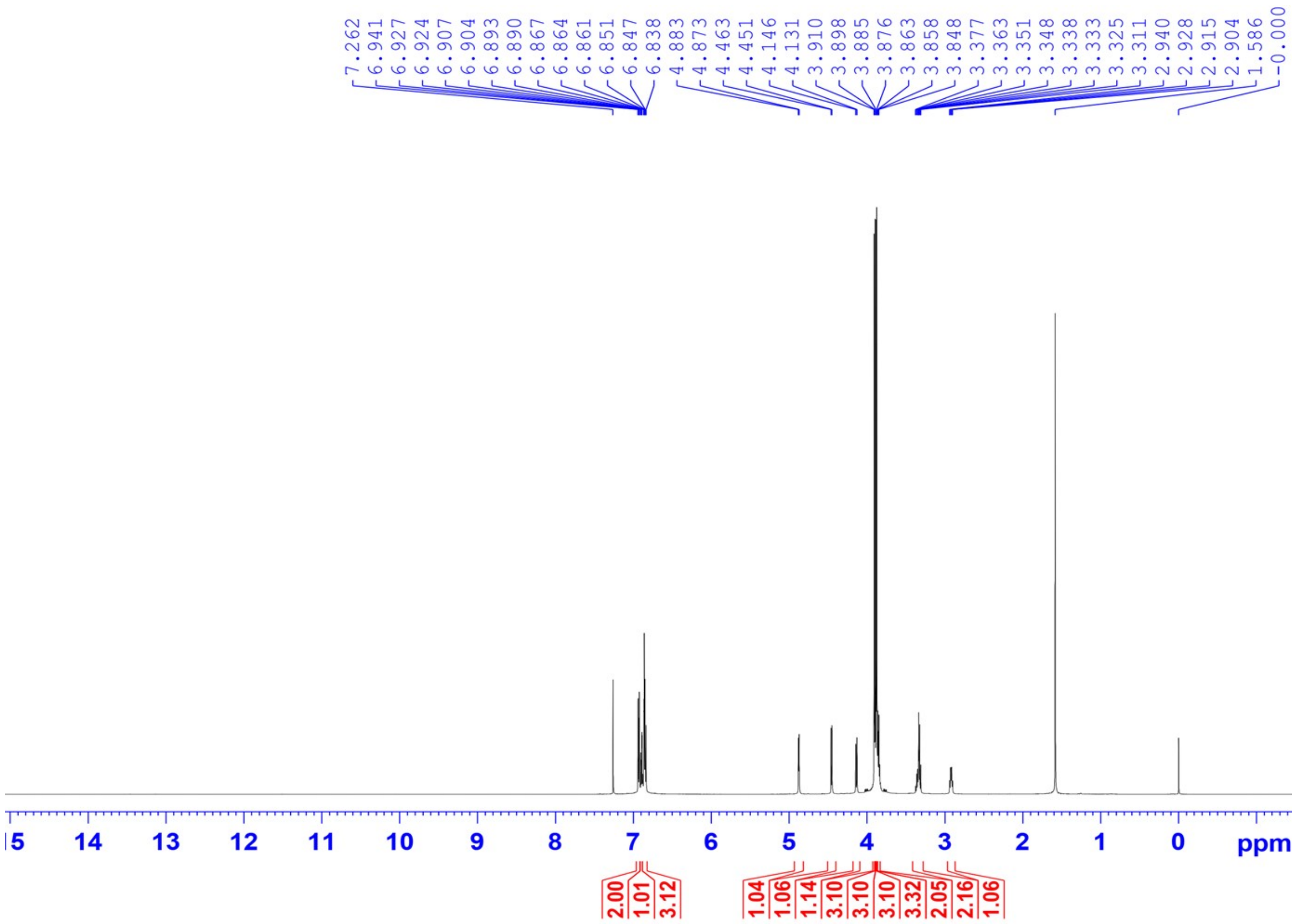

Figure S37. 1H-NMR spectrum of compound 3

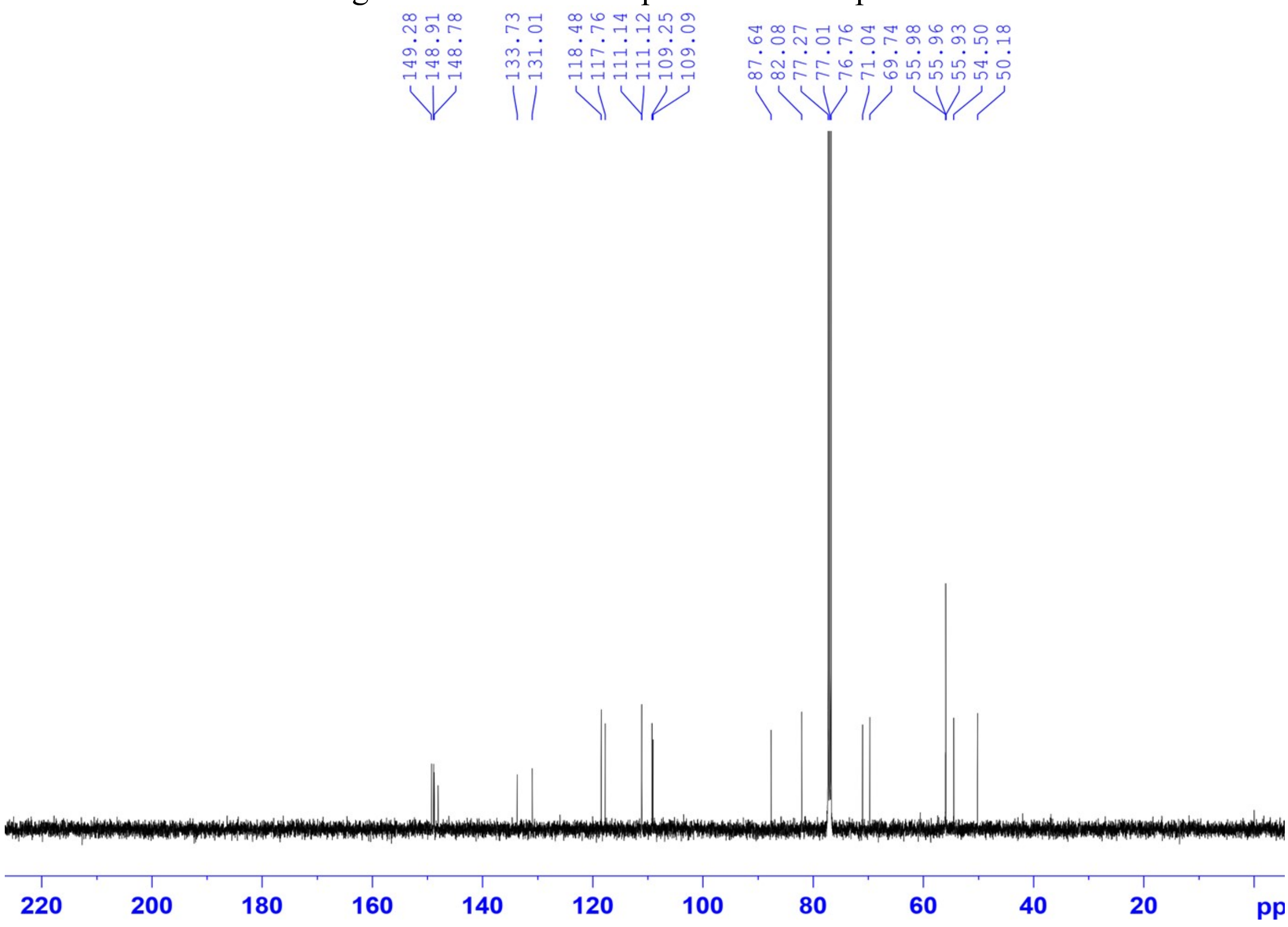

Figure S38. 13C-NMR spectrum of compound 3

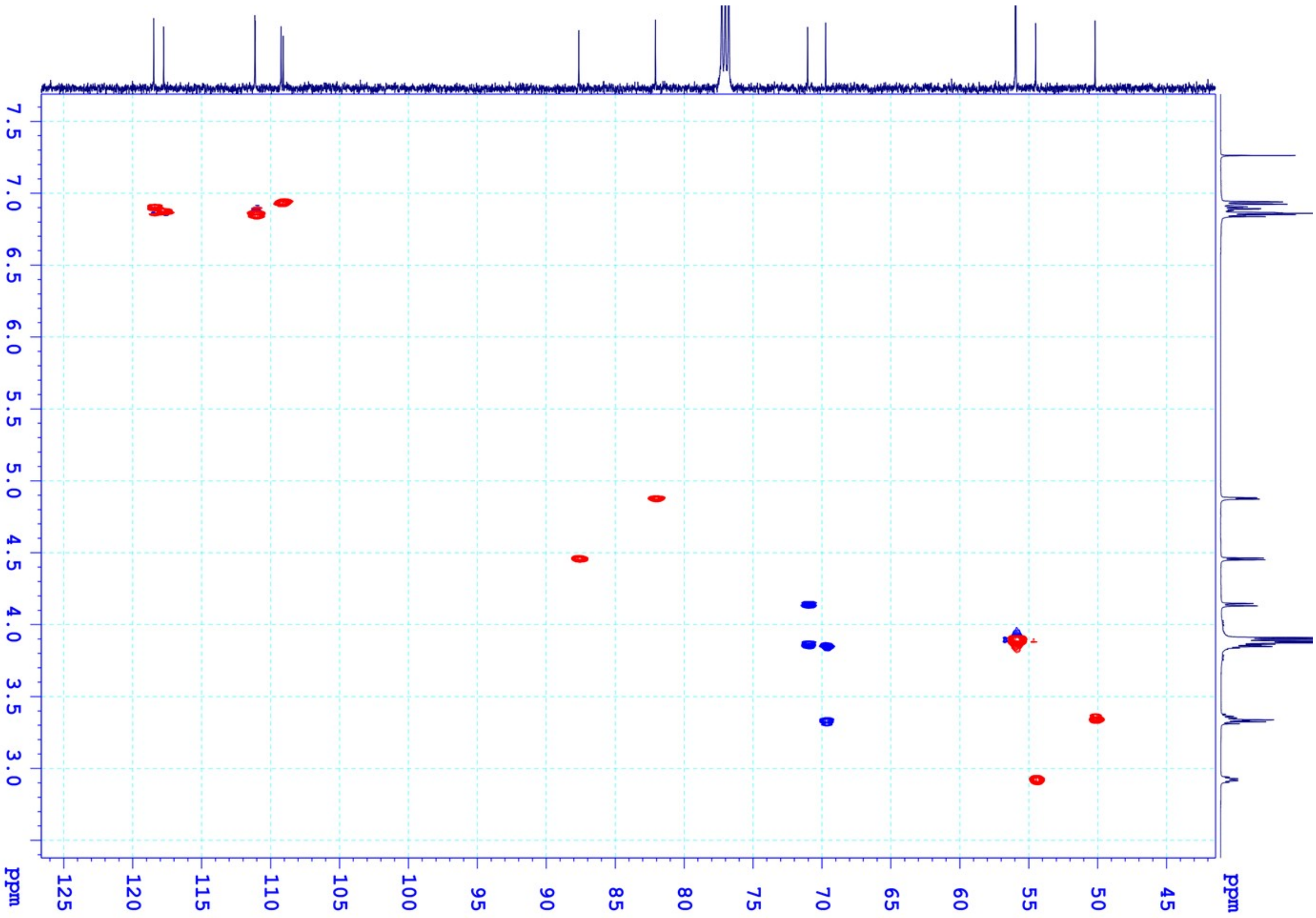

Figure S39. HSQC spectrum of compound 3

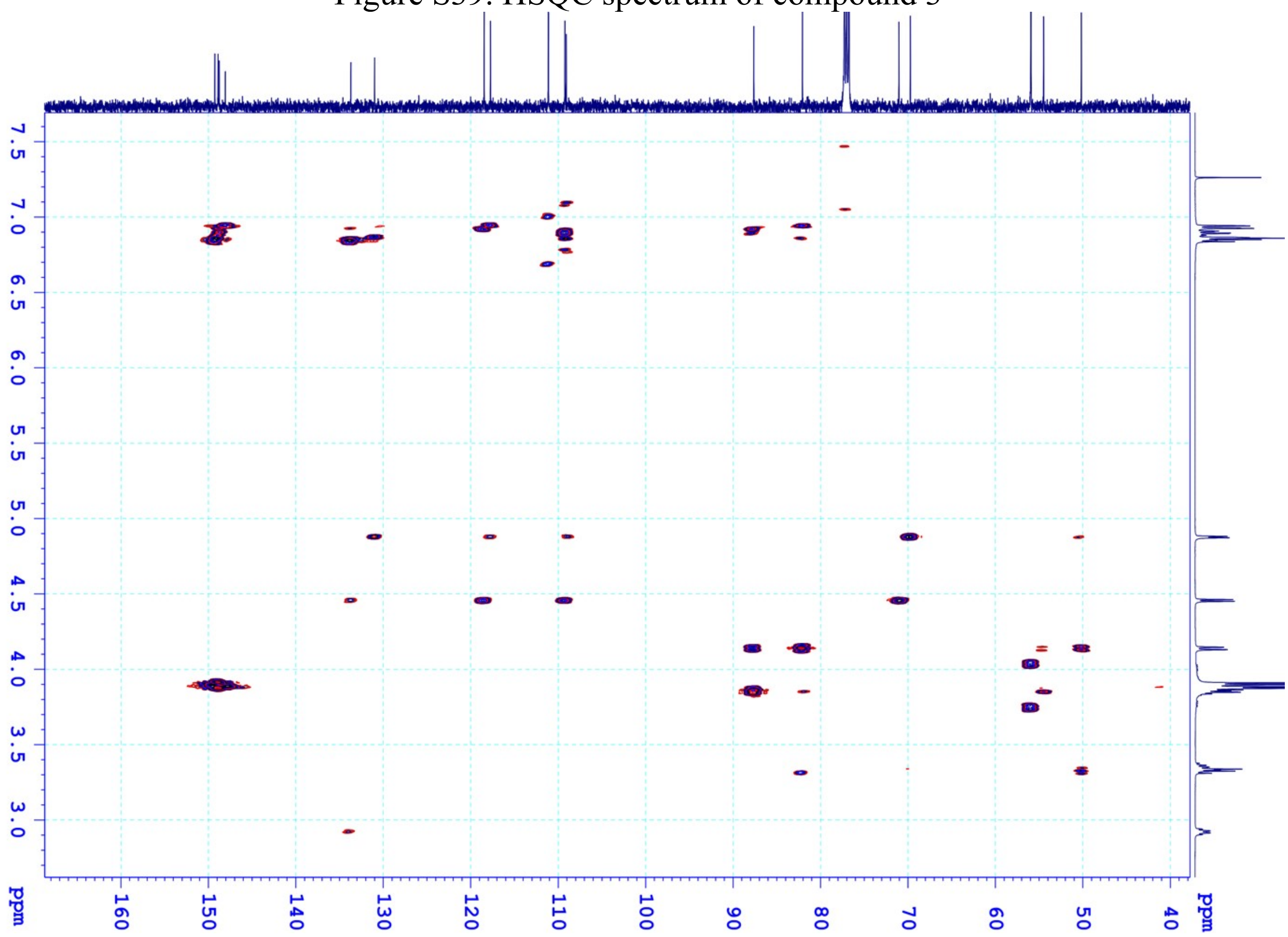

Figure S40. HMBC spectrum of compound 3

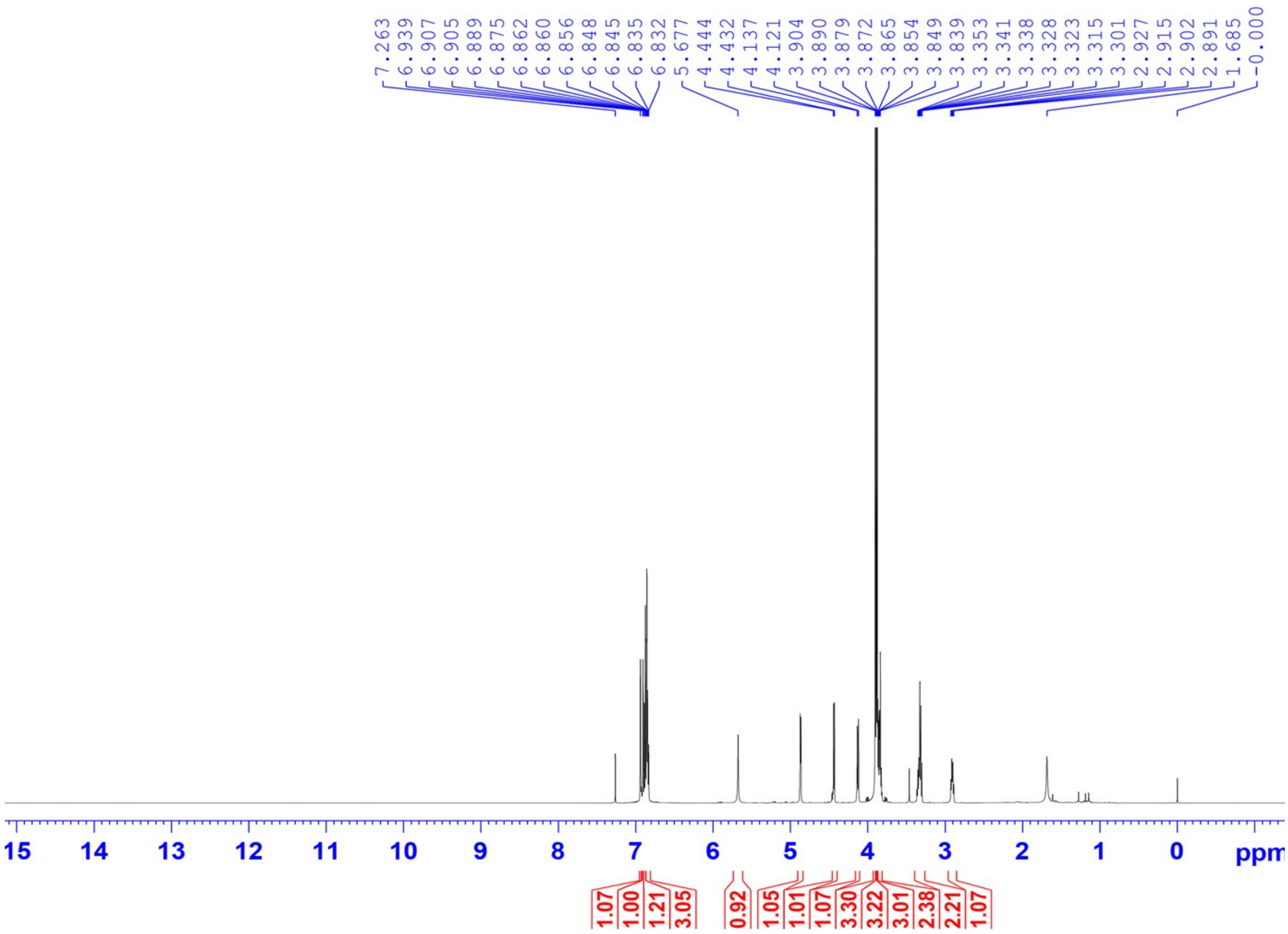

Figure S41. 1H-NMR spectrum of compound 4

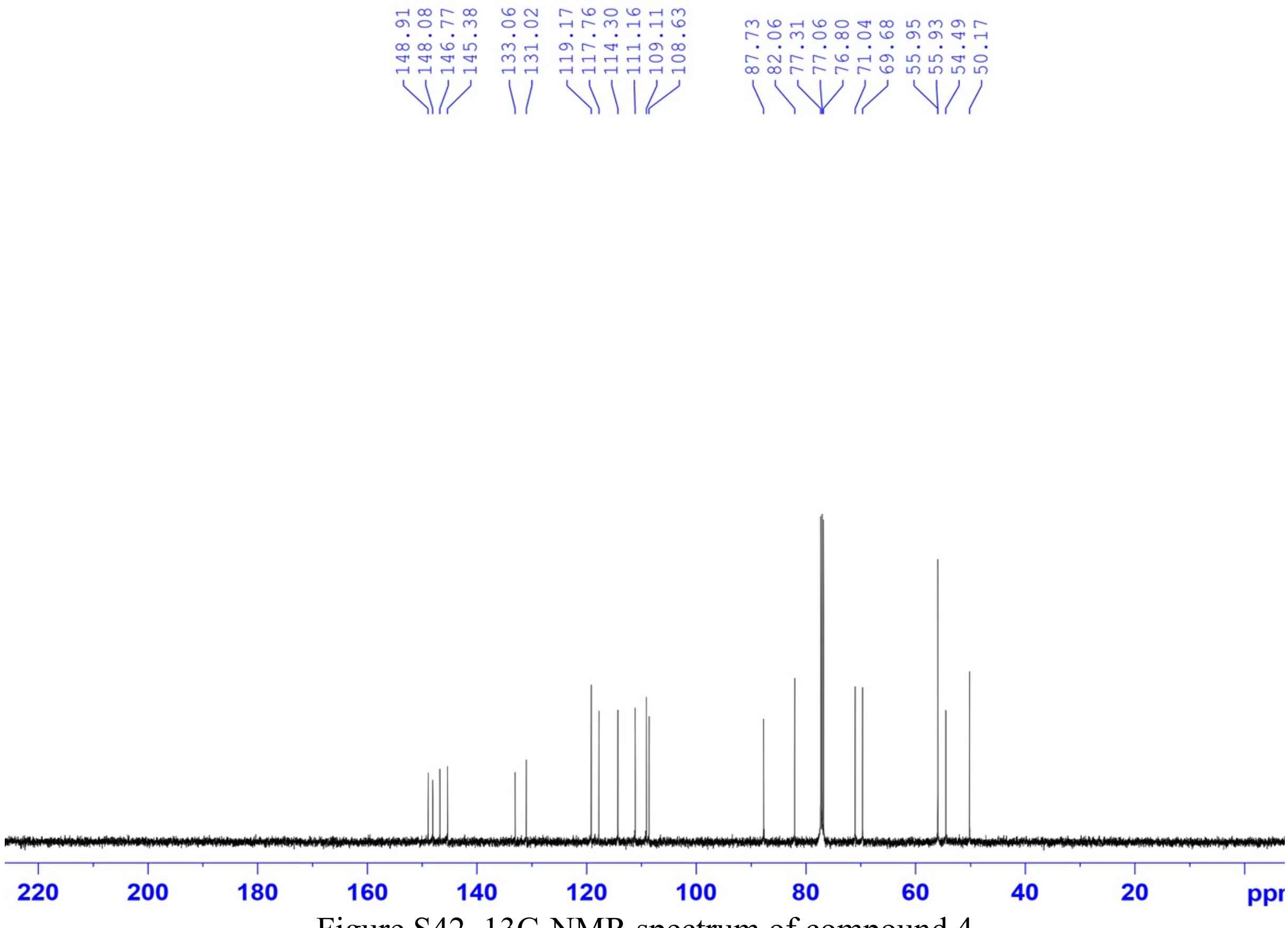

Figure S42. 13C-NMR spectrum of compound 4

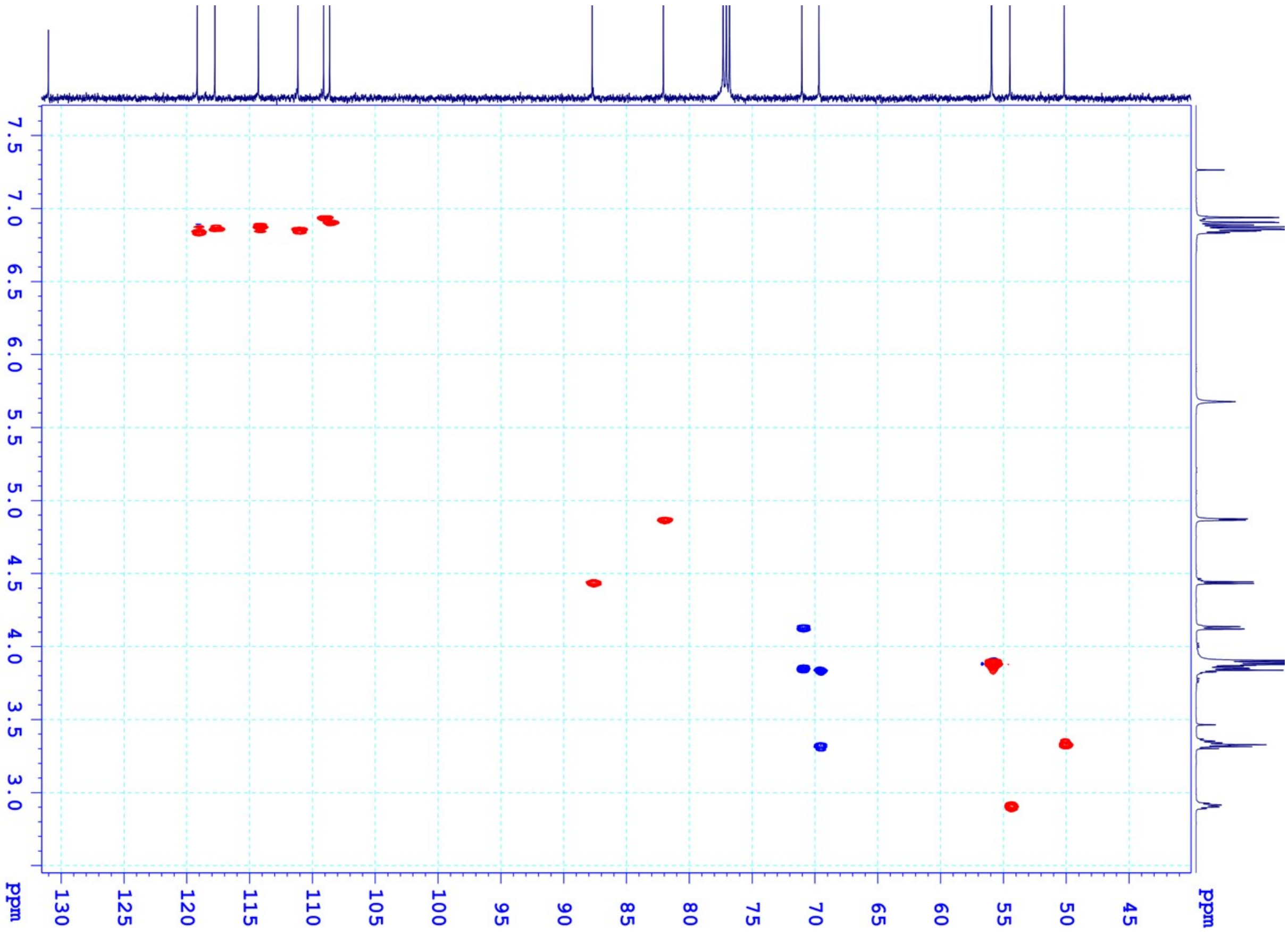

Figure S43. HSQC spectrum of compound 4

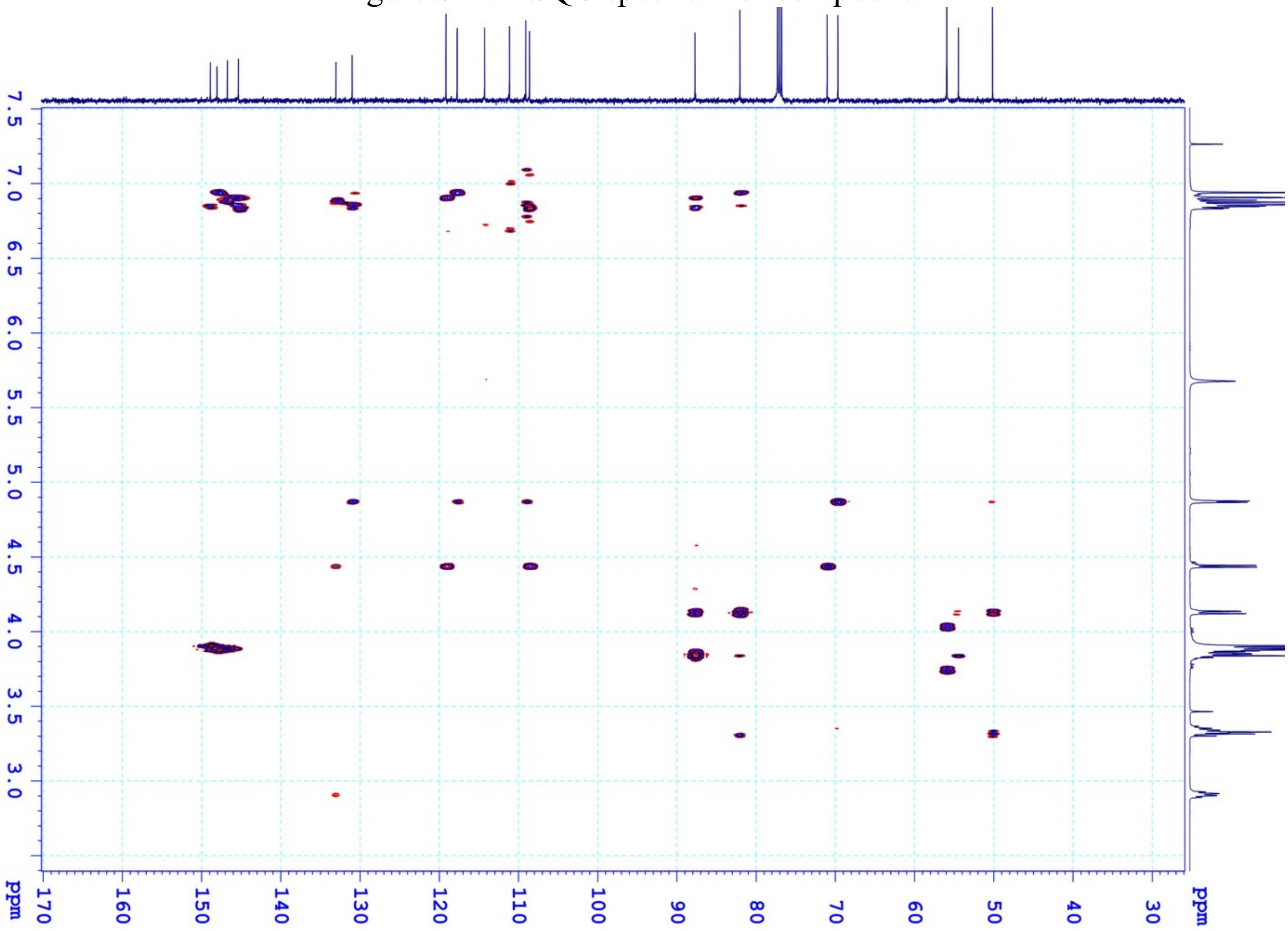

Figure S44. HMBC spectrum of compound 4

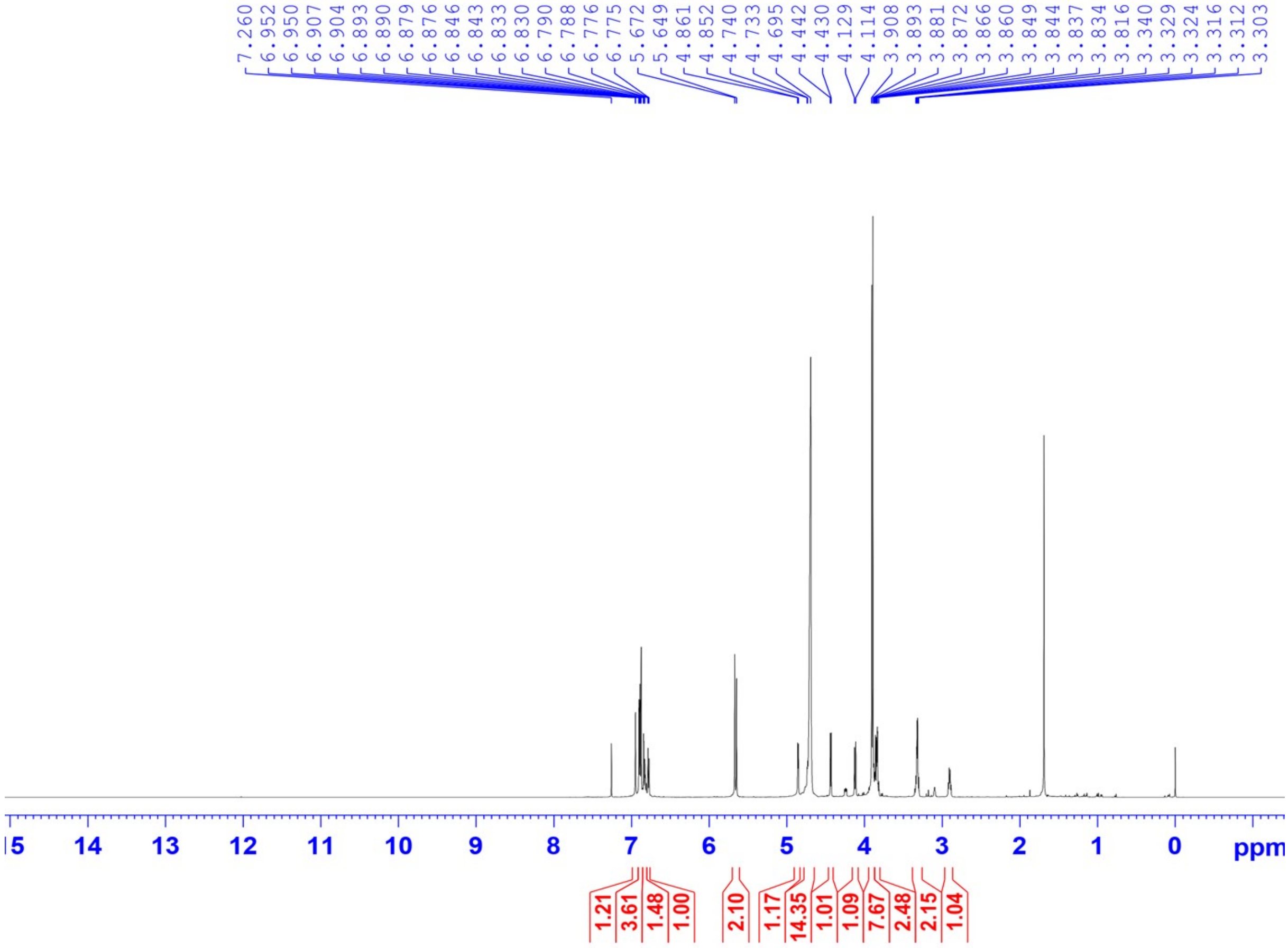

Figure S45. 1H-NMR spectrum of compound 5

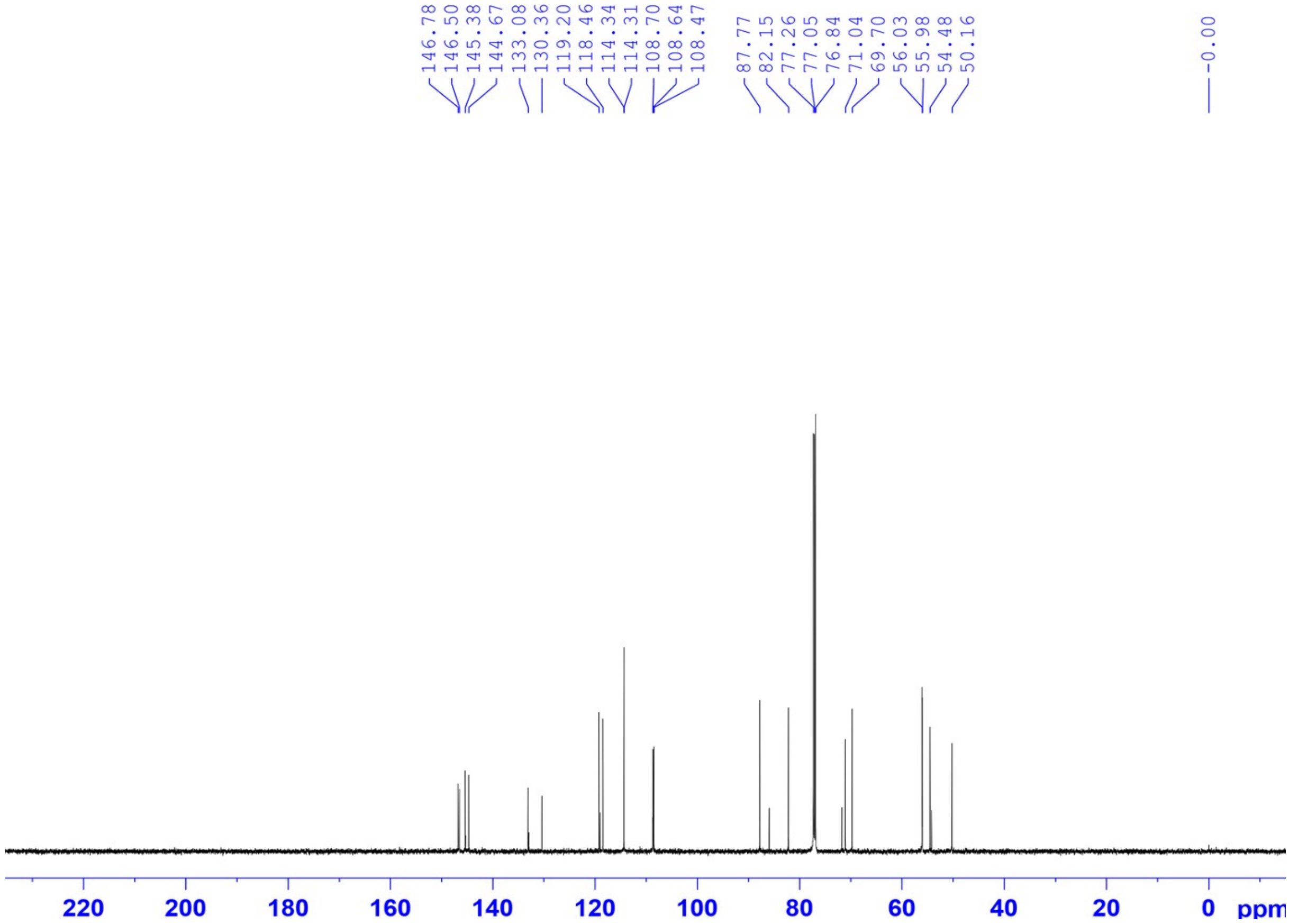

Figure S46. 13C-NMR spectrum of compound 5

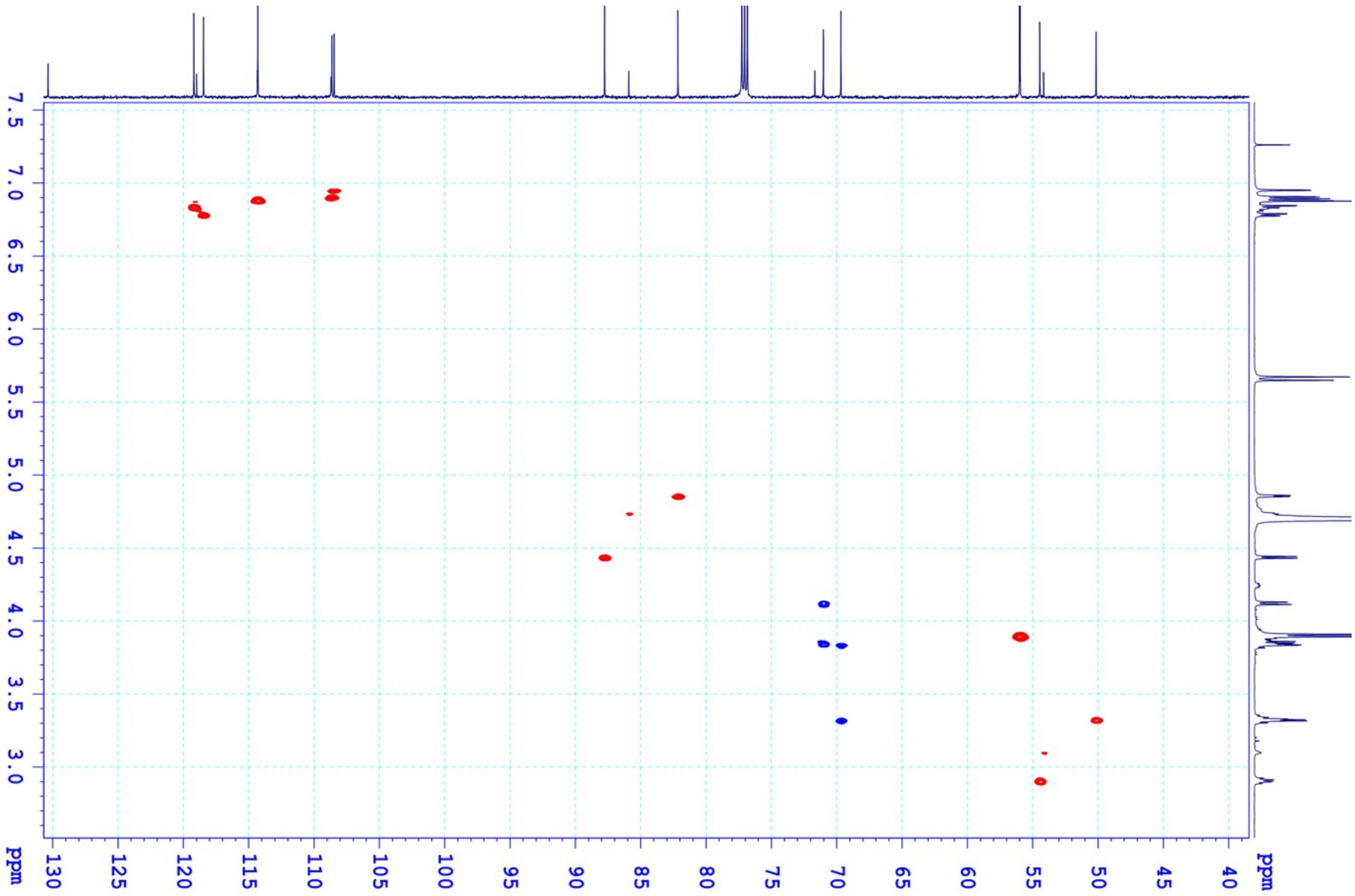

Figure S47. HSQC spectrum of compound 5

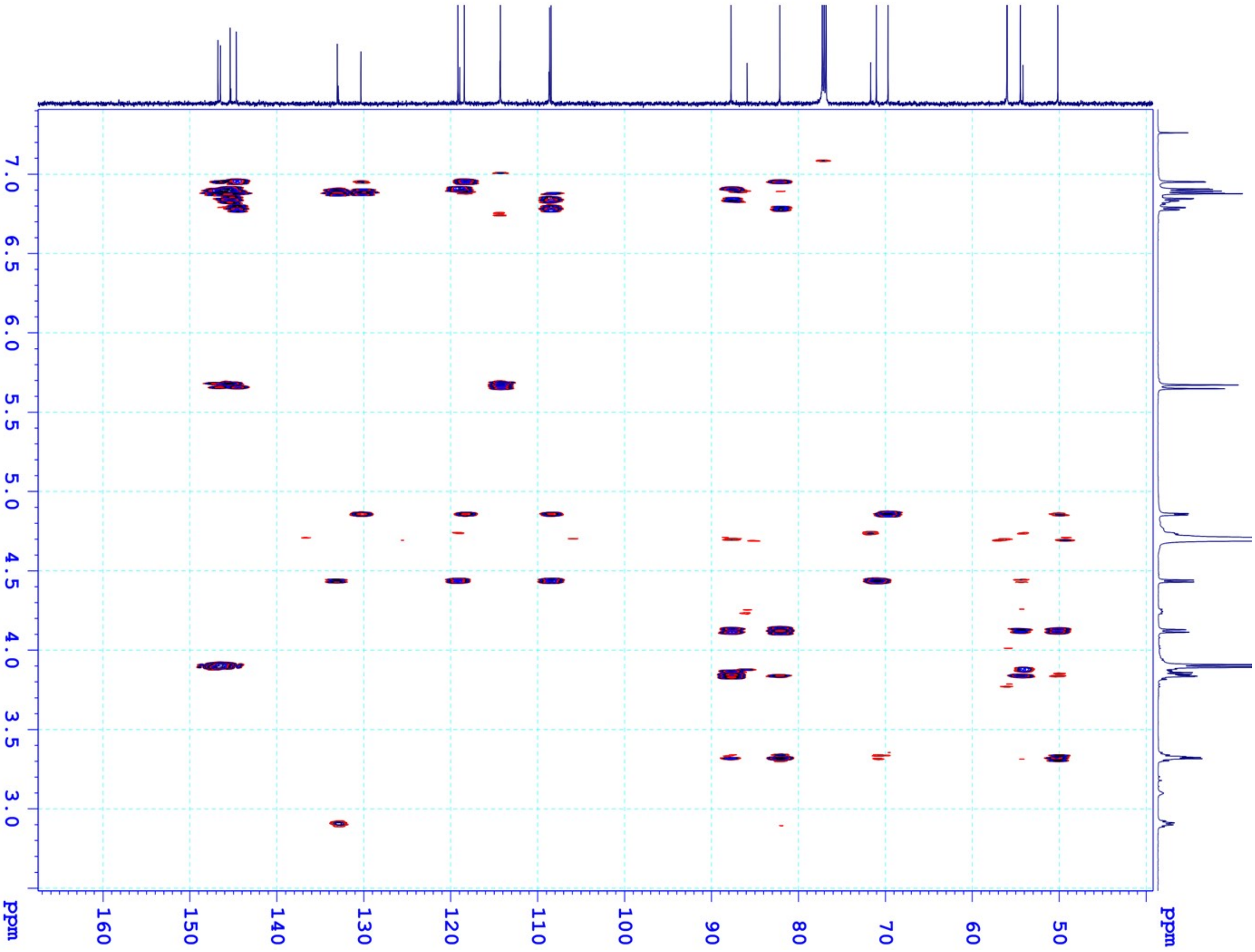

Figure S48. HMBC spectrum of compound 5

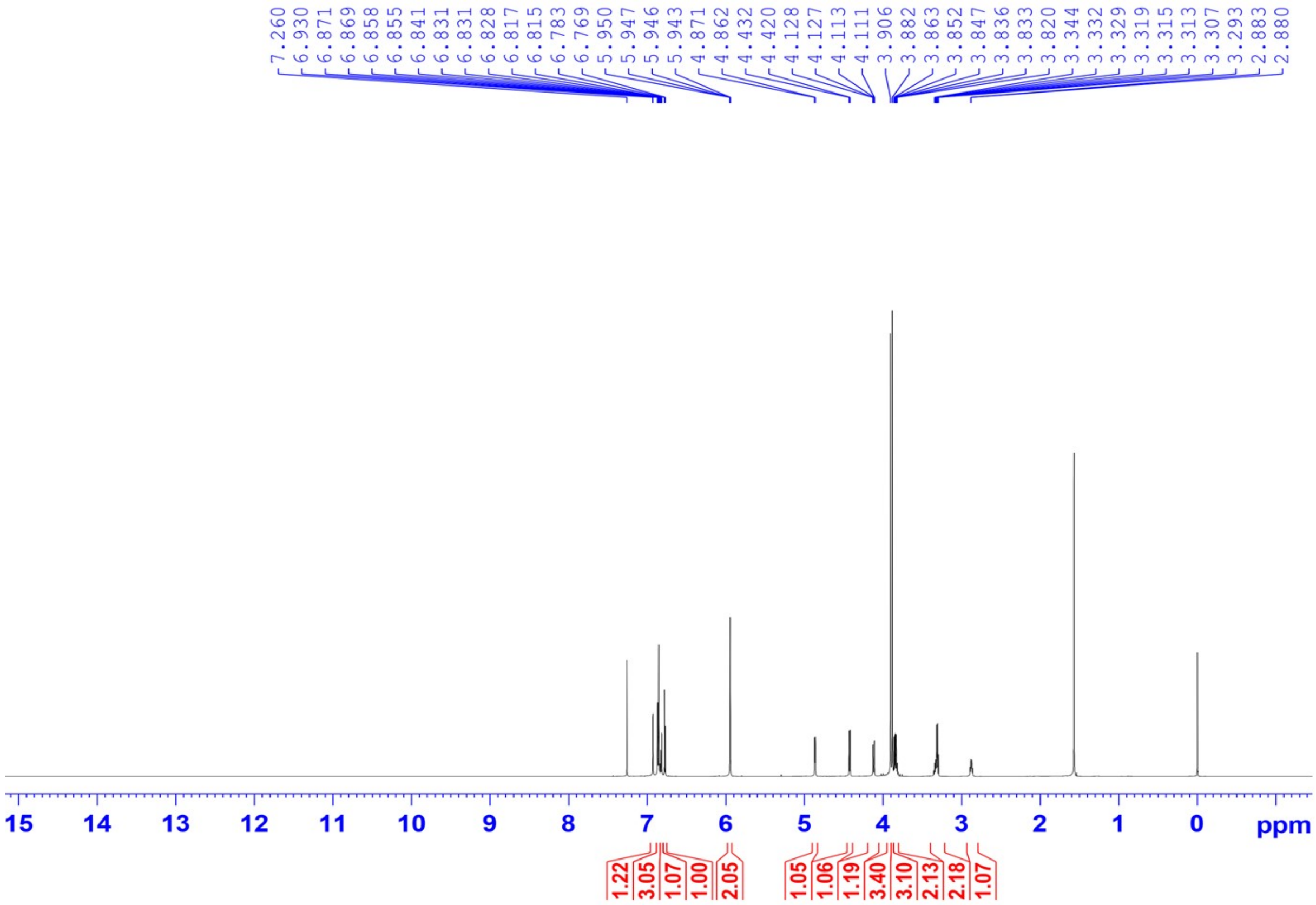

Figure S49. 1H-NMR spectrum of compound 6

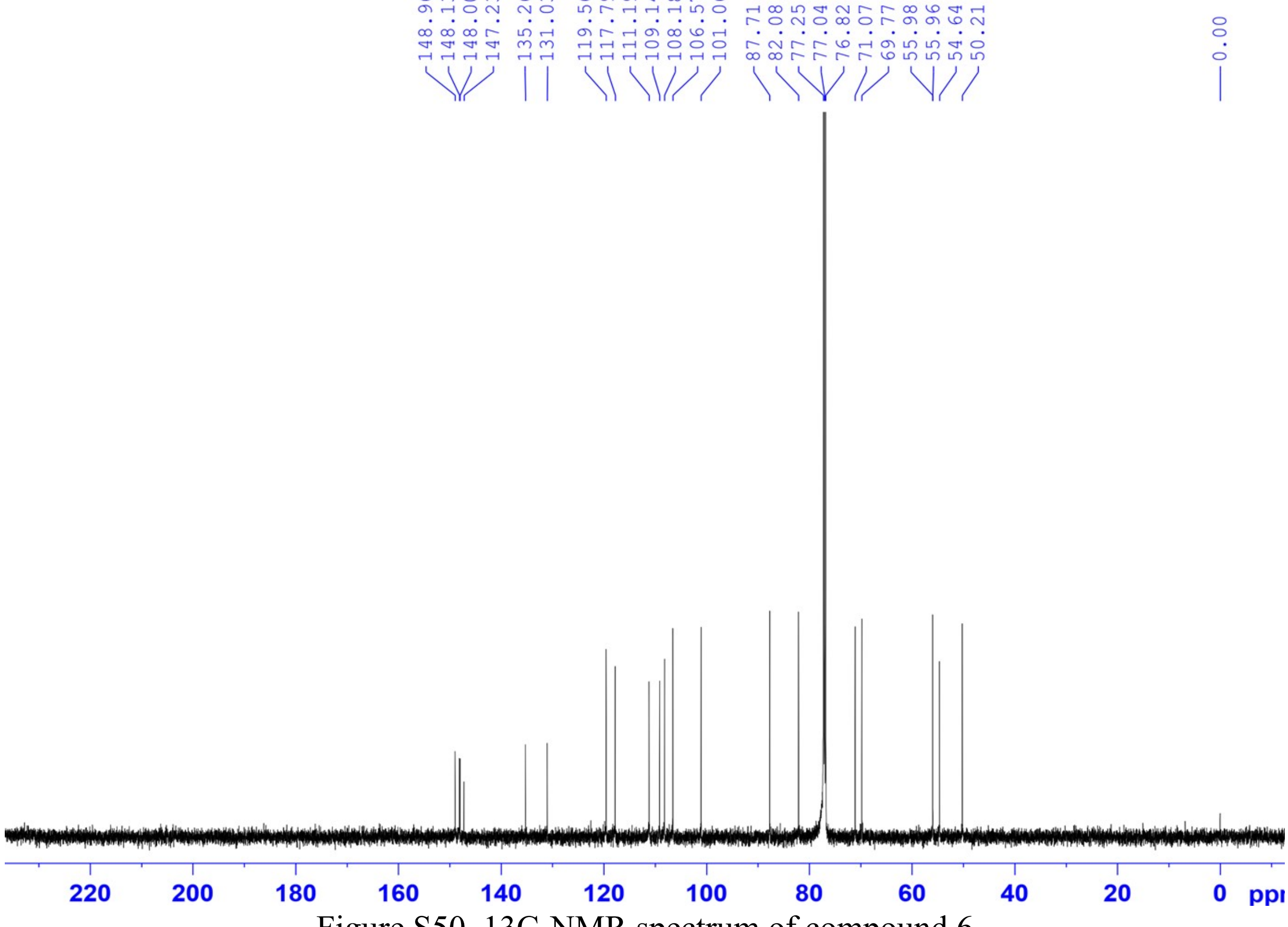

Figure S50. 13C-NMR spectrum of compound 6

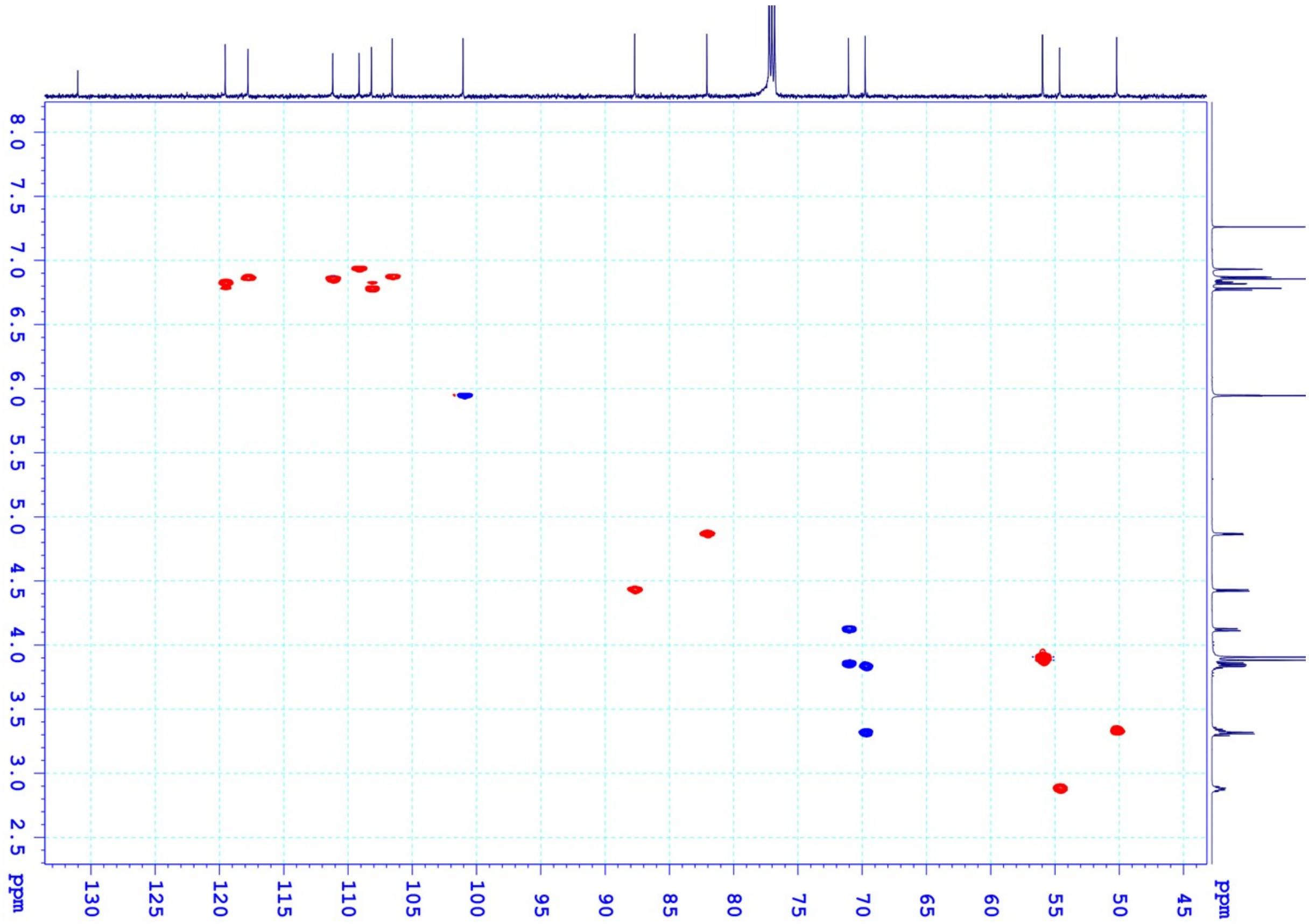

Figure S51. HSQC spectrum of compound 6

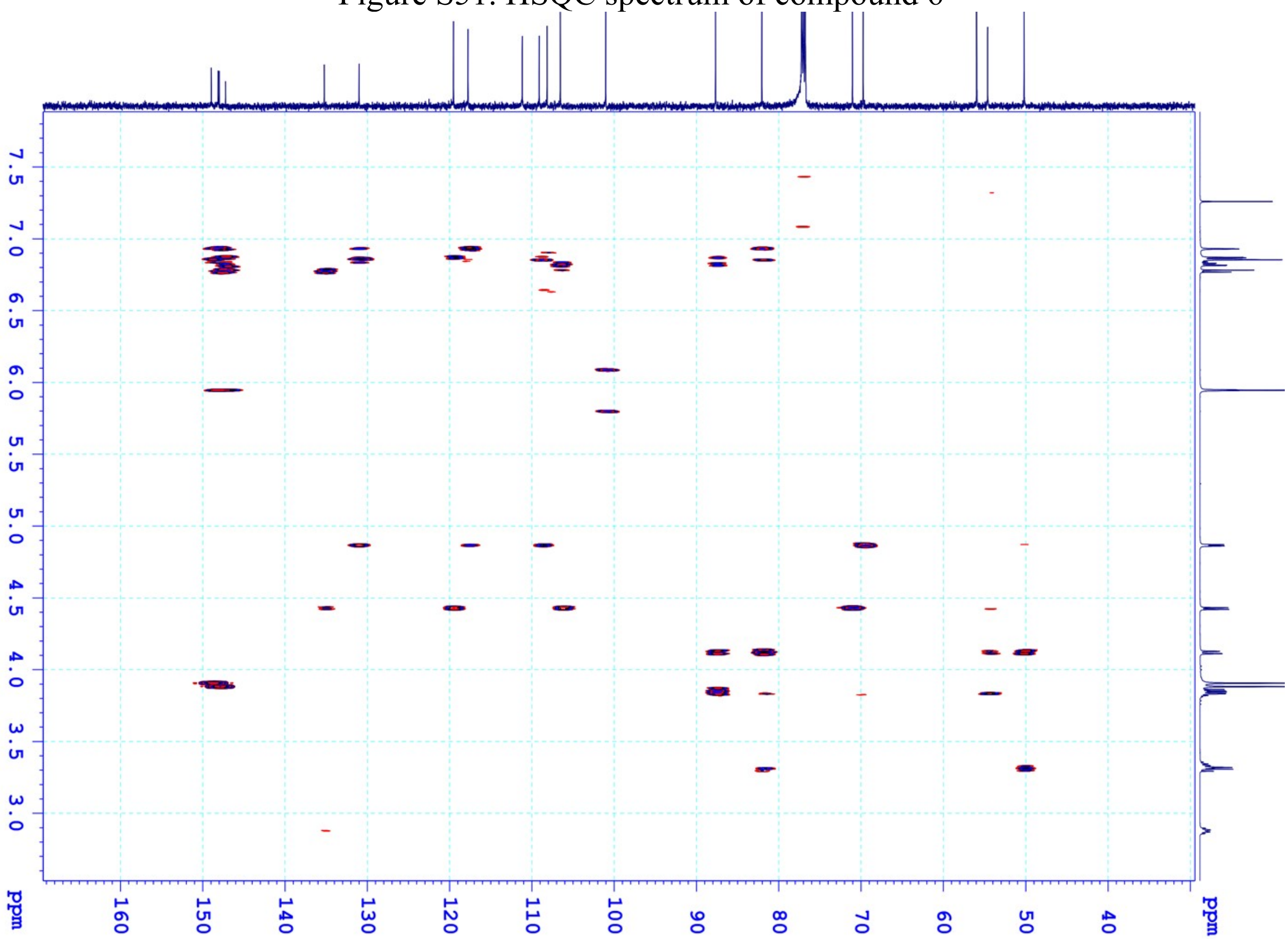

Figure S52. HMBC spectrum of compound 6

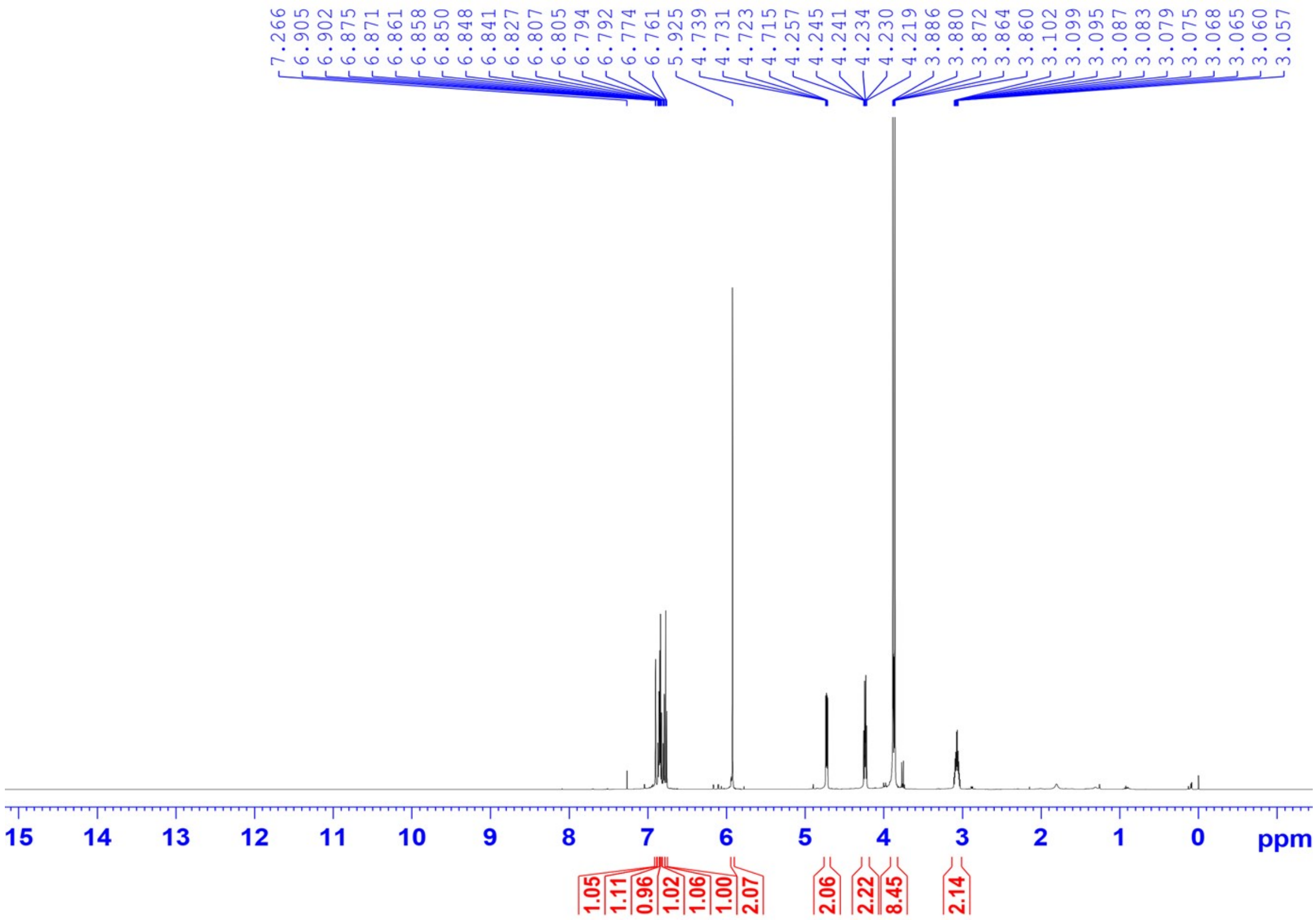

Figure S53. 1H-NMR spectrum of compound 7

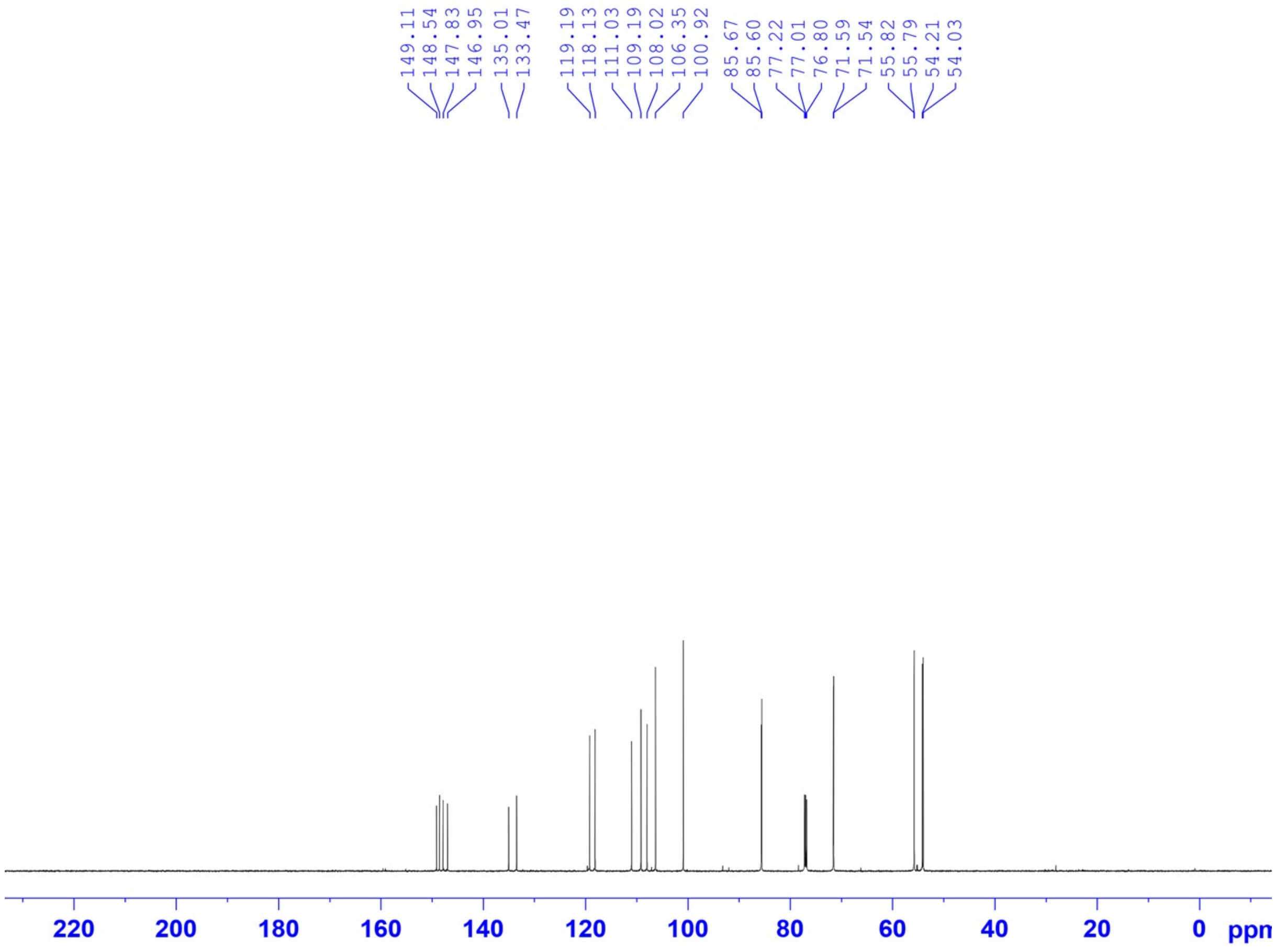

Figure S54. 13C-NMR spectrum of compound 7

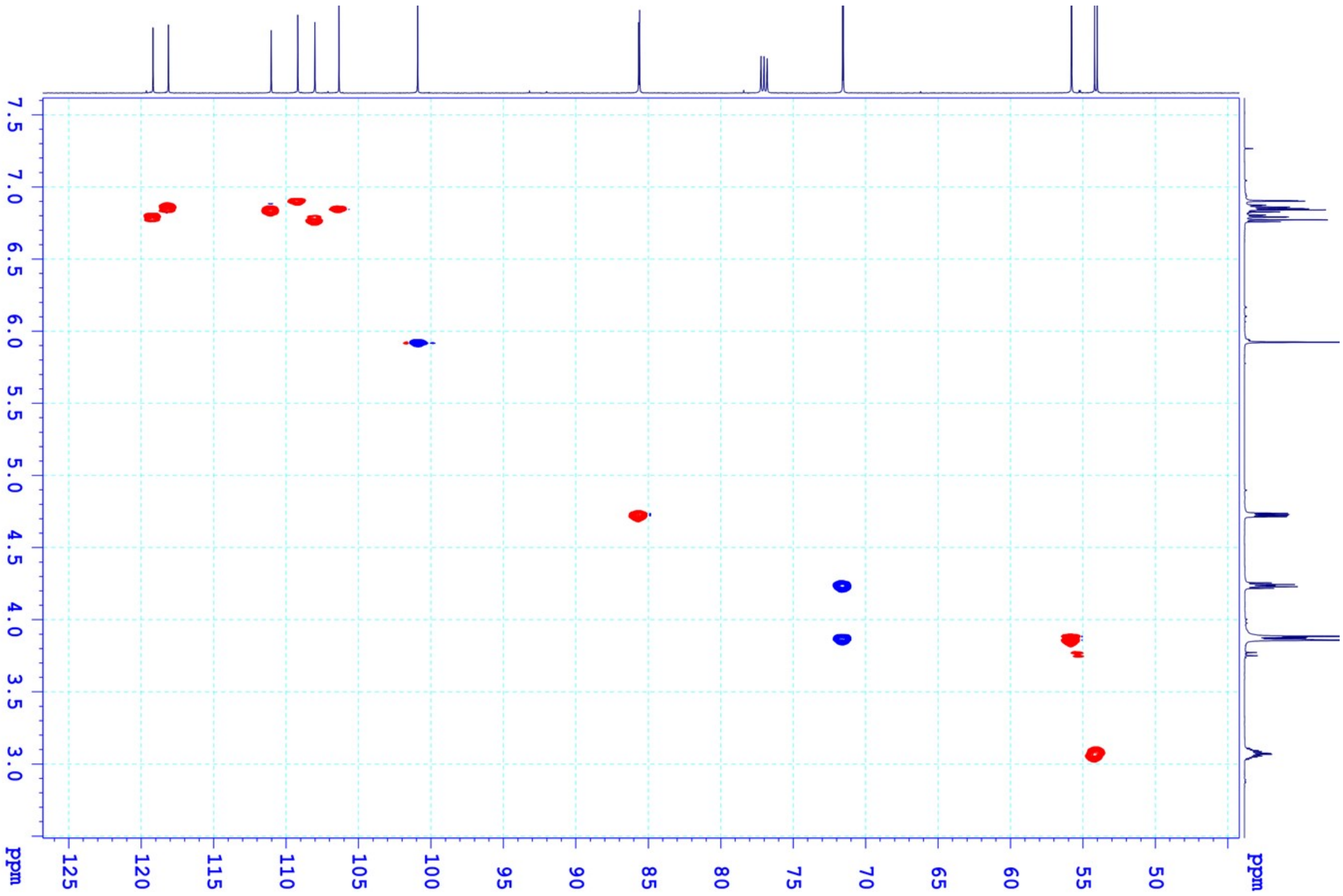

Figure S55. HSQC spectrum of compound 7

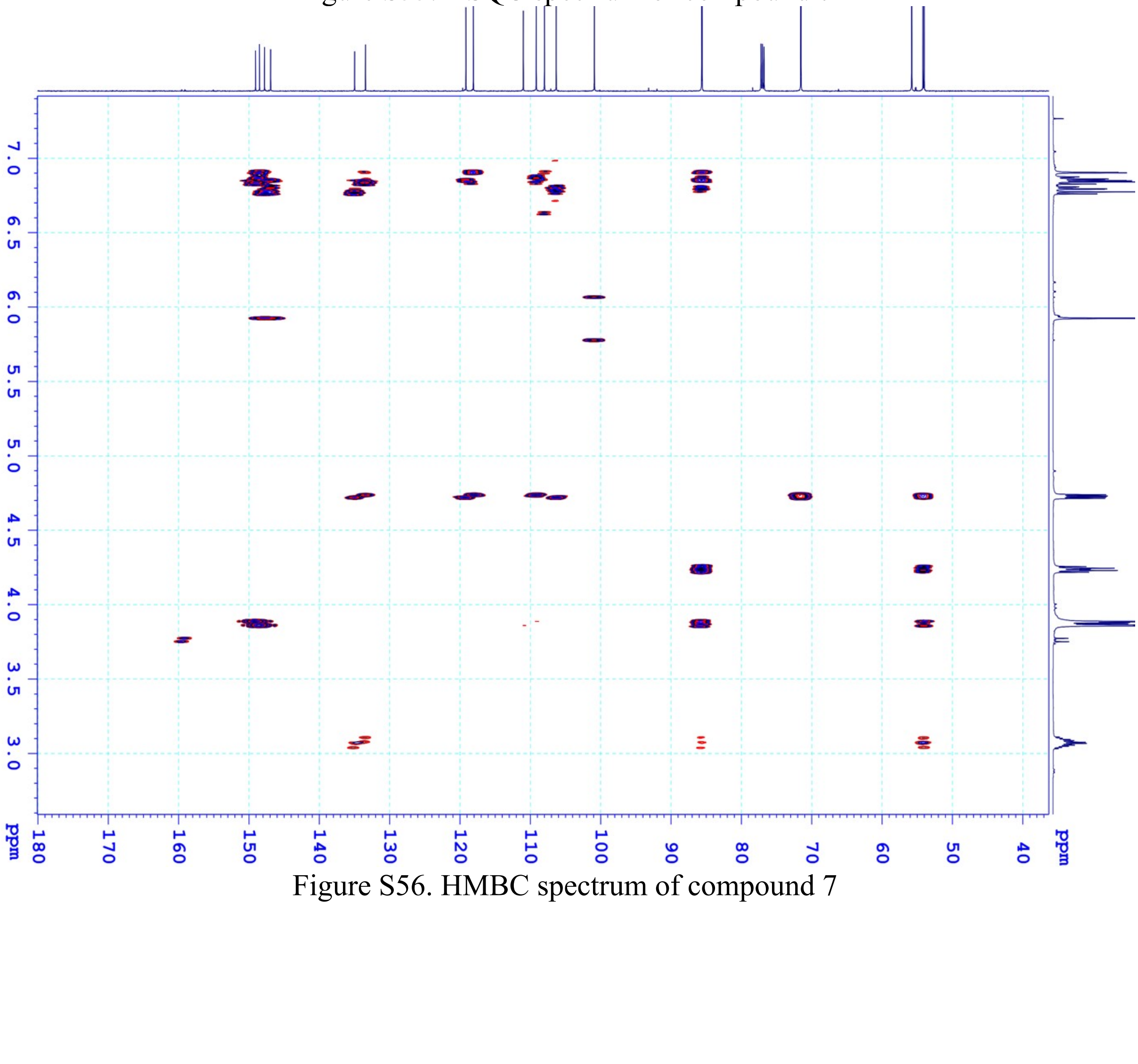

Figure S56. HMBC spectrum of compound 7

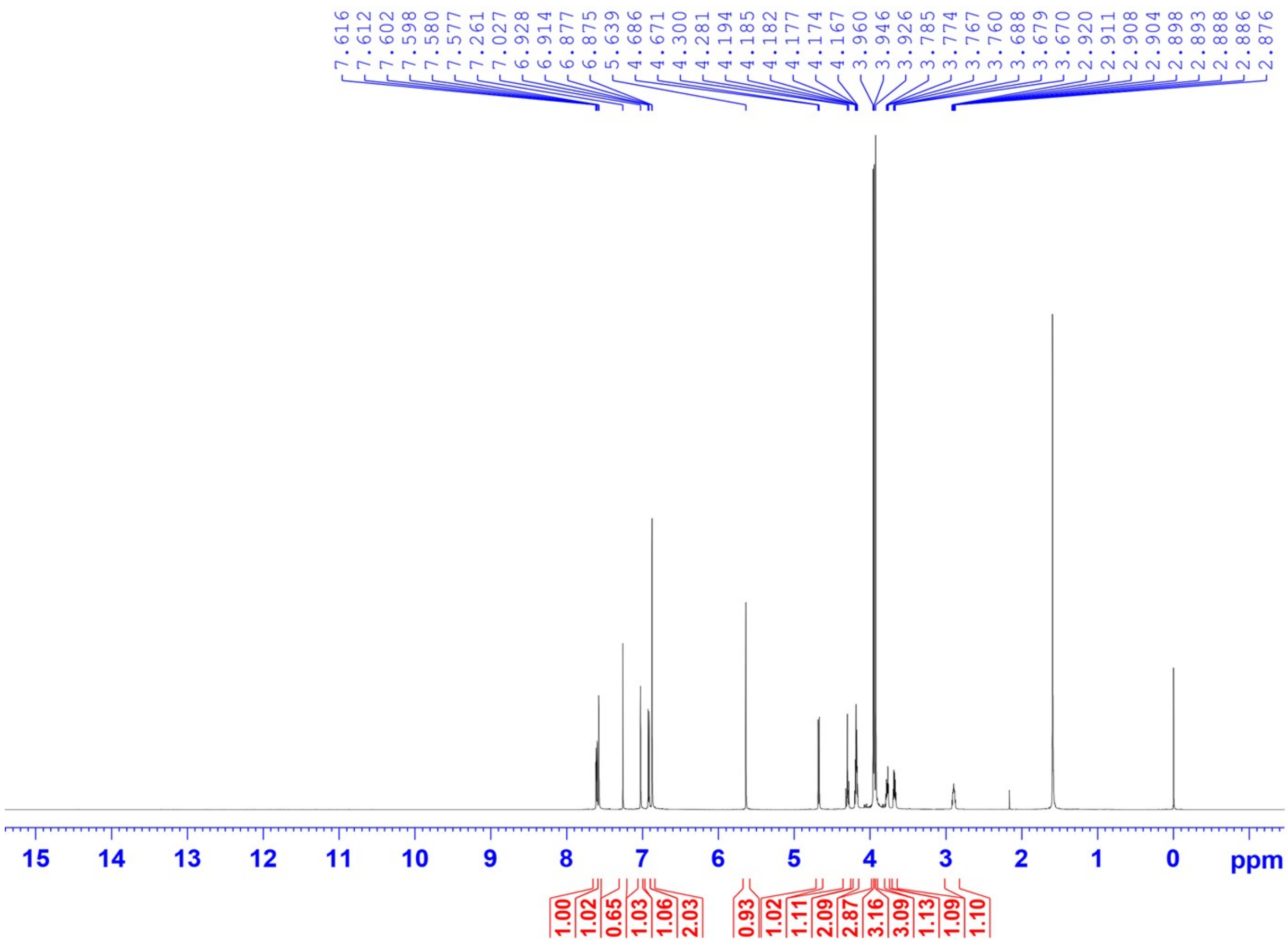

Figure S57. 1H-NMR spectrum of compound 8

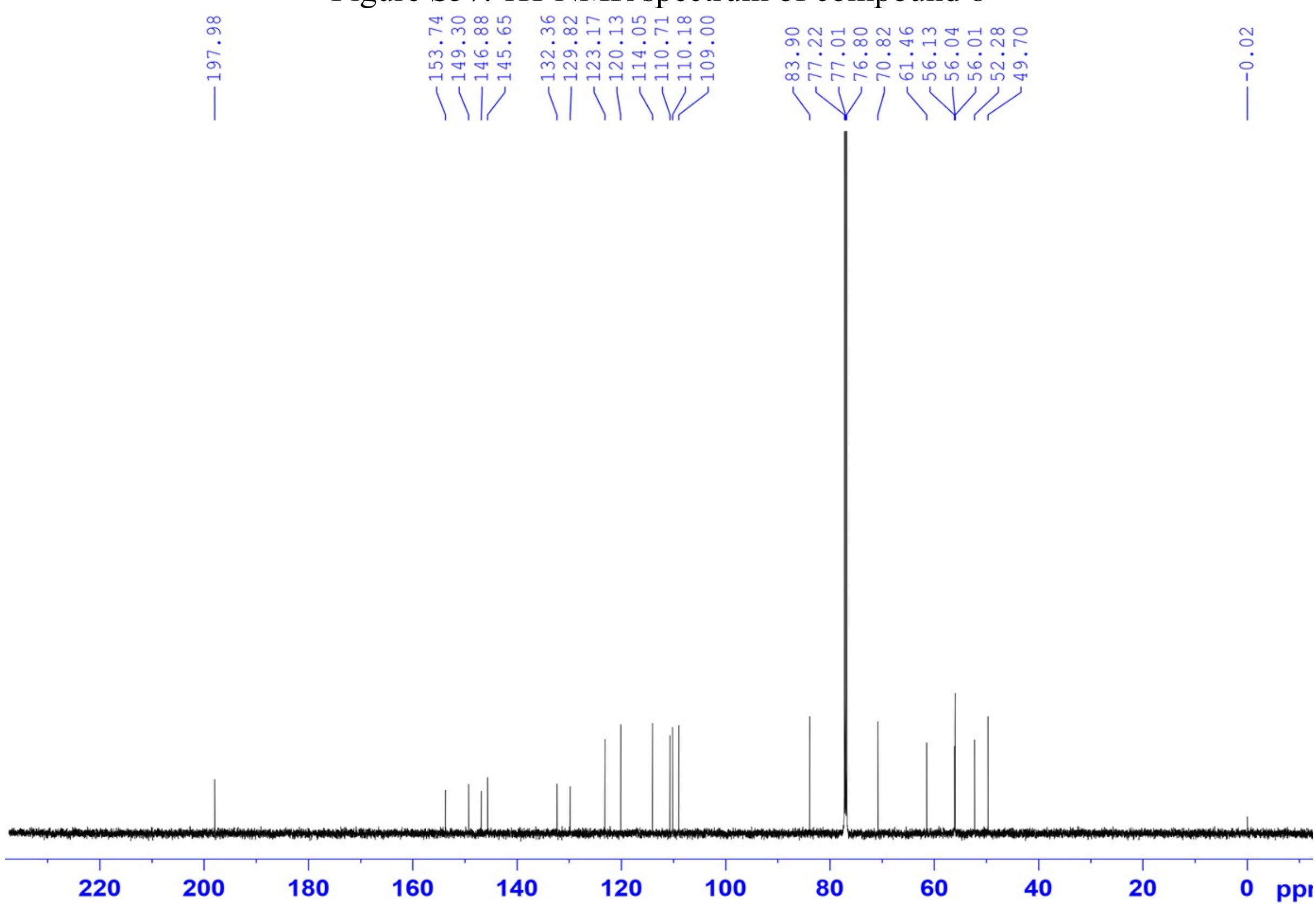

Figure S58. 13C-NMR spectrum of compound 8

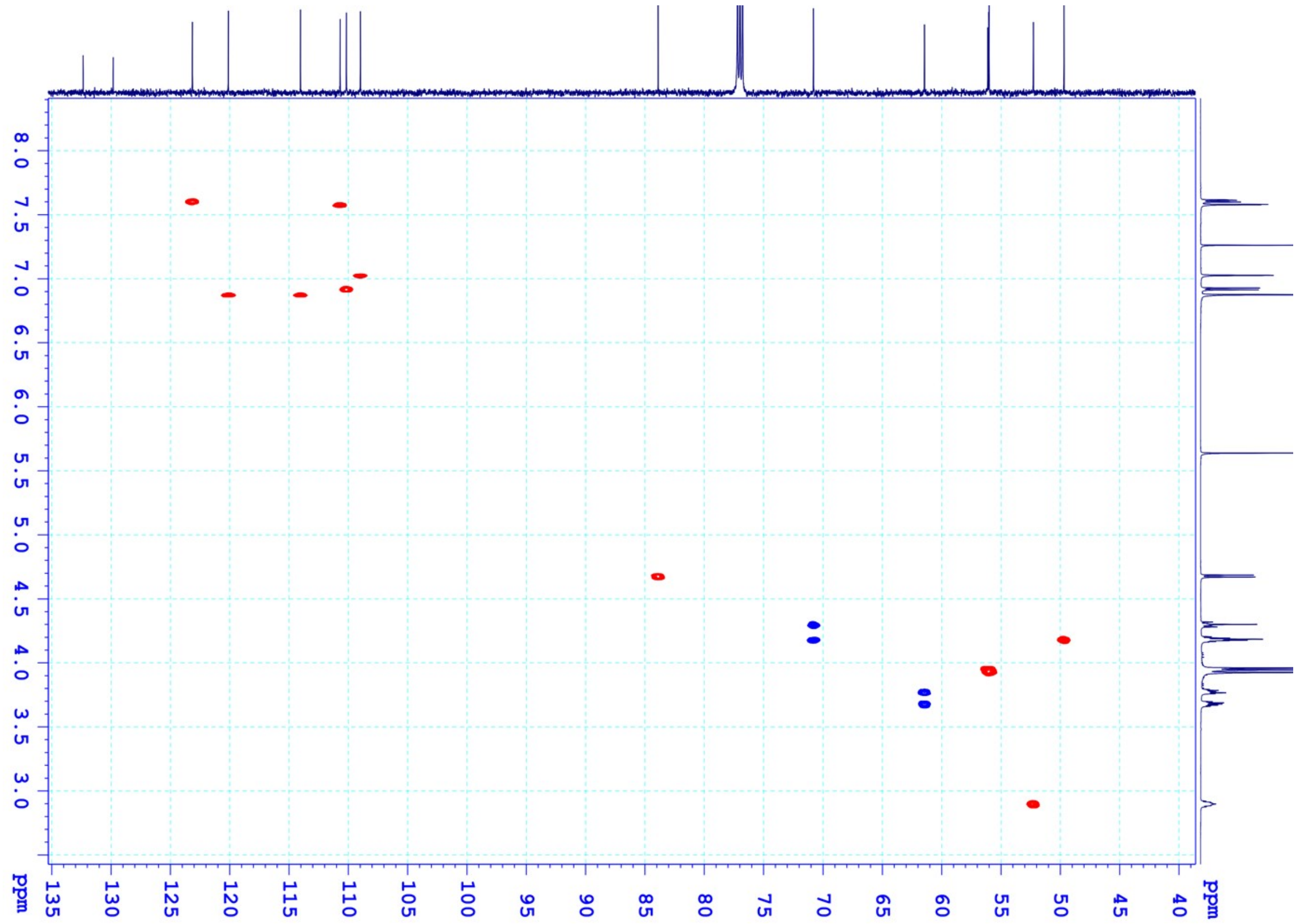

Figure S59. HSQC spectrum of compound 8

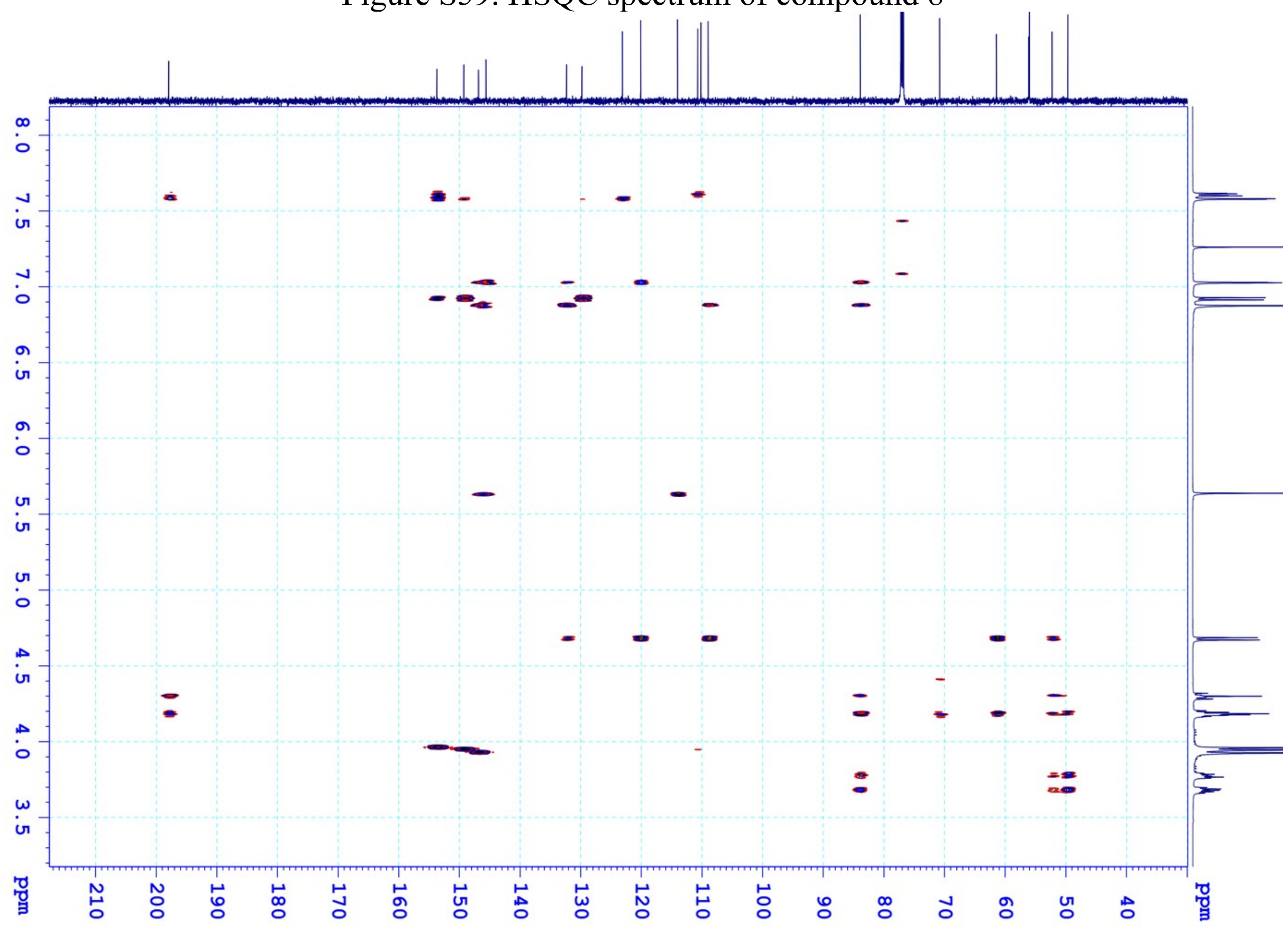

Figure S60. HMBC spectrum of compound 8

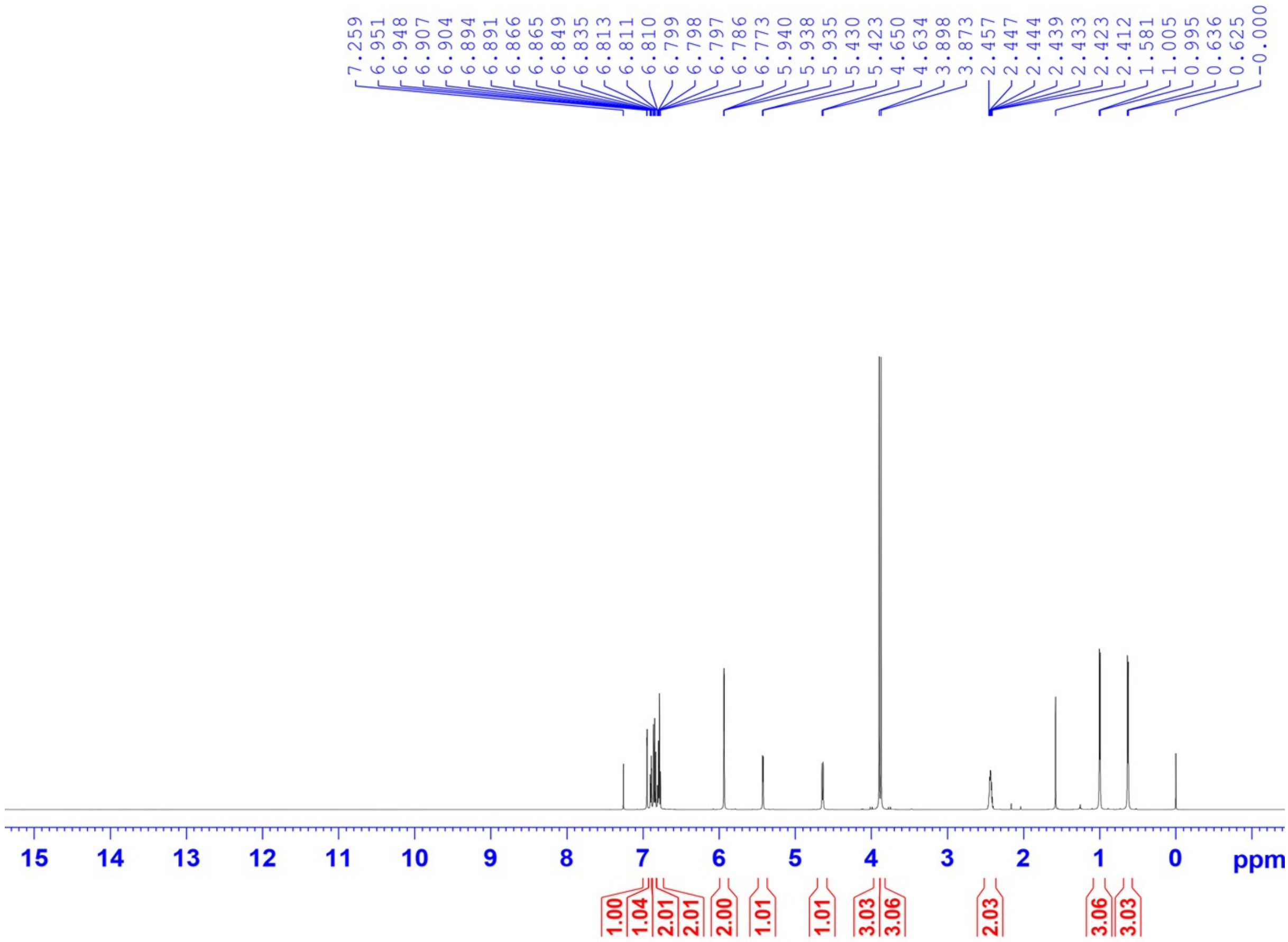

Figure S61. 1H-NMR spectrum of compound 9

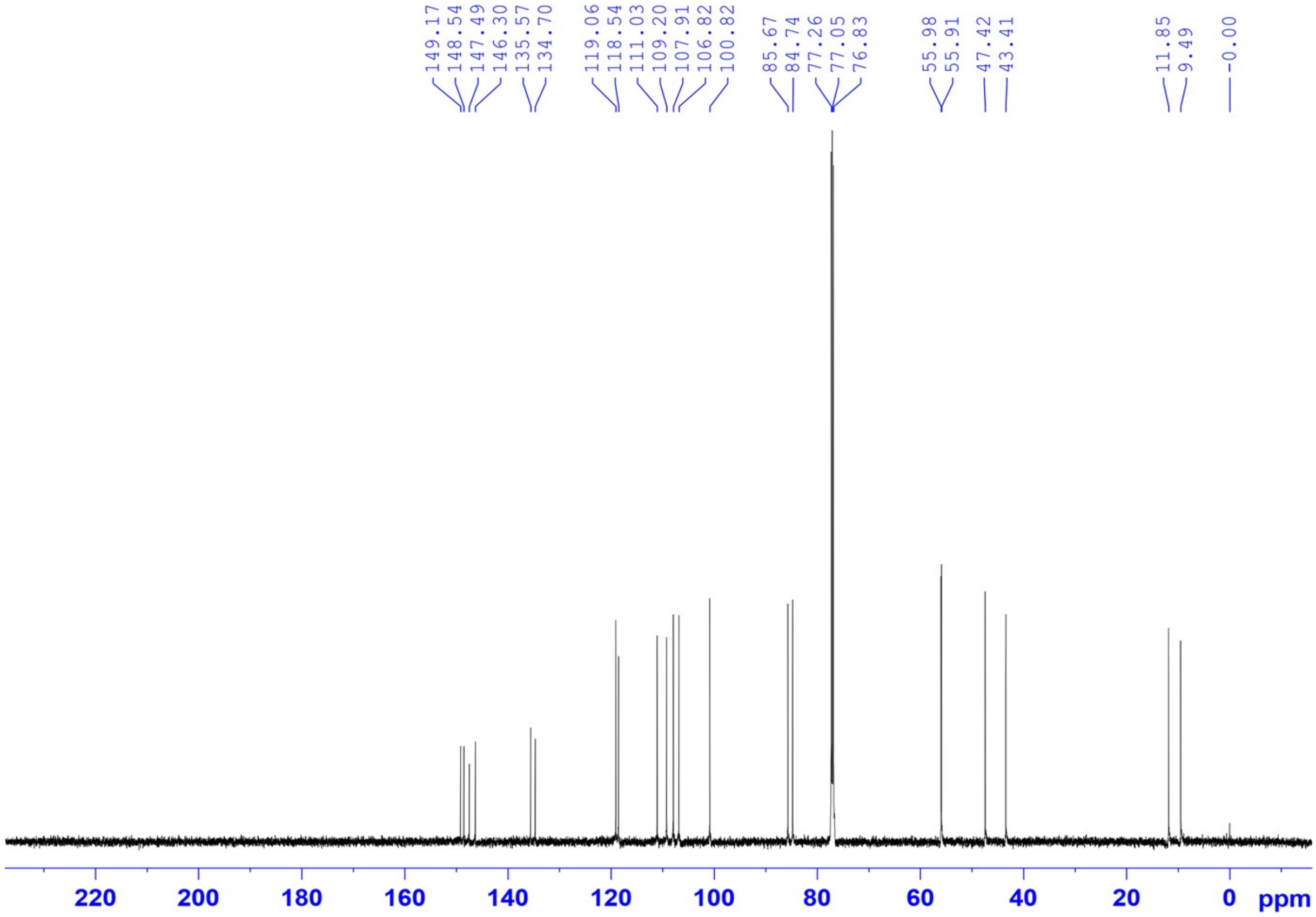

Figure S62. 13C-NMR spectrum of compound 9

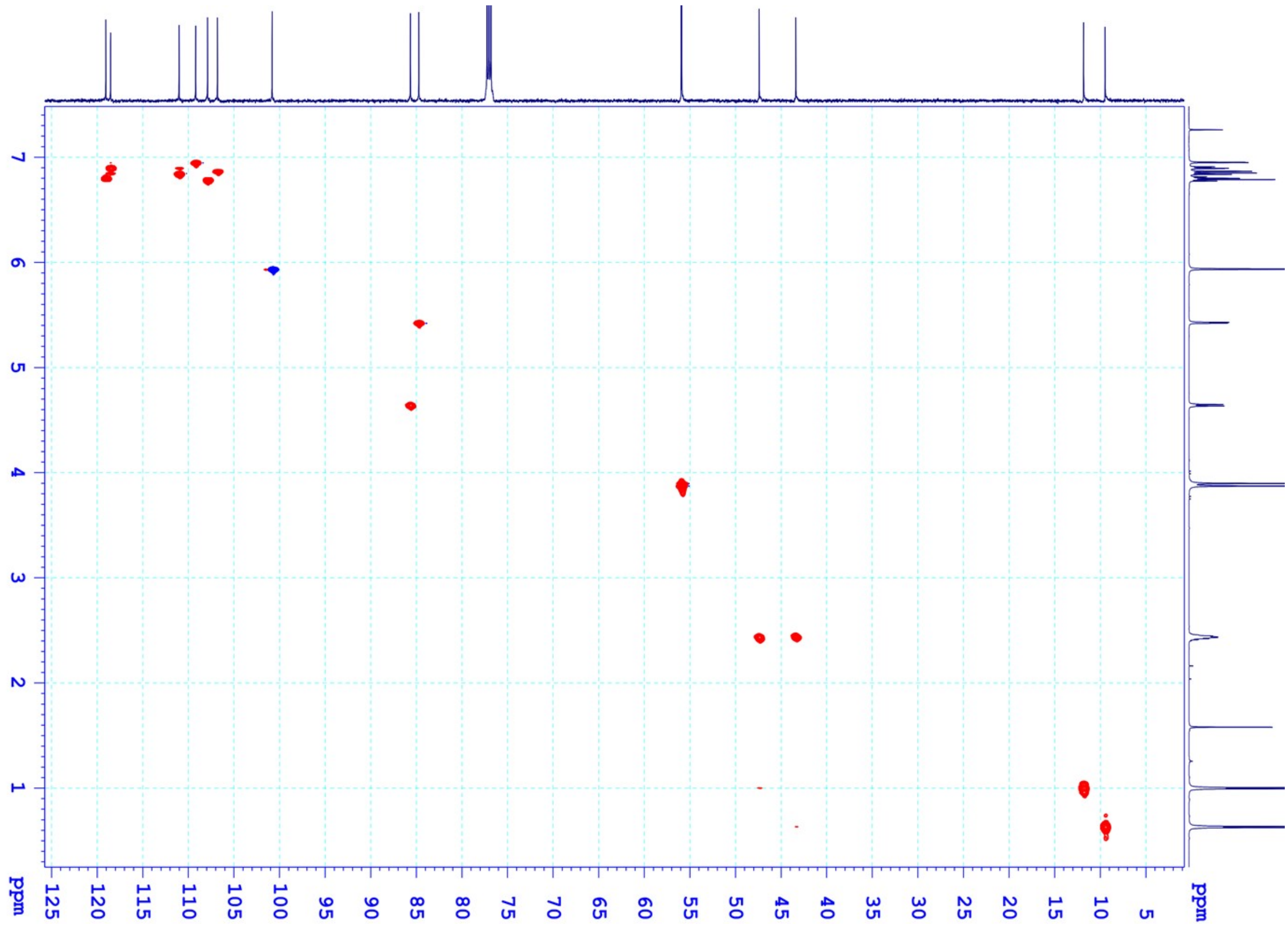

Figure S63. HSQC spectrum of compound 9

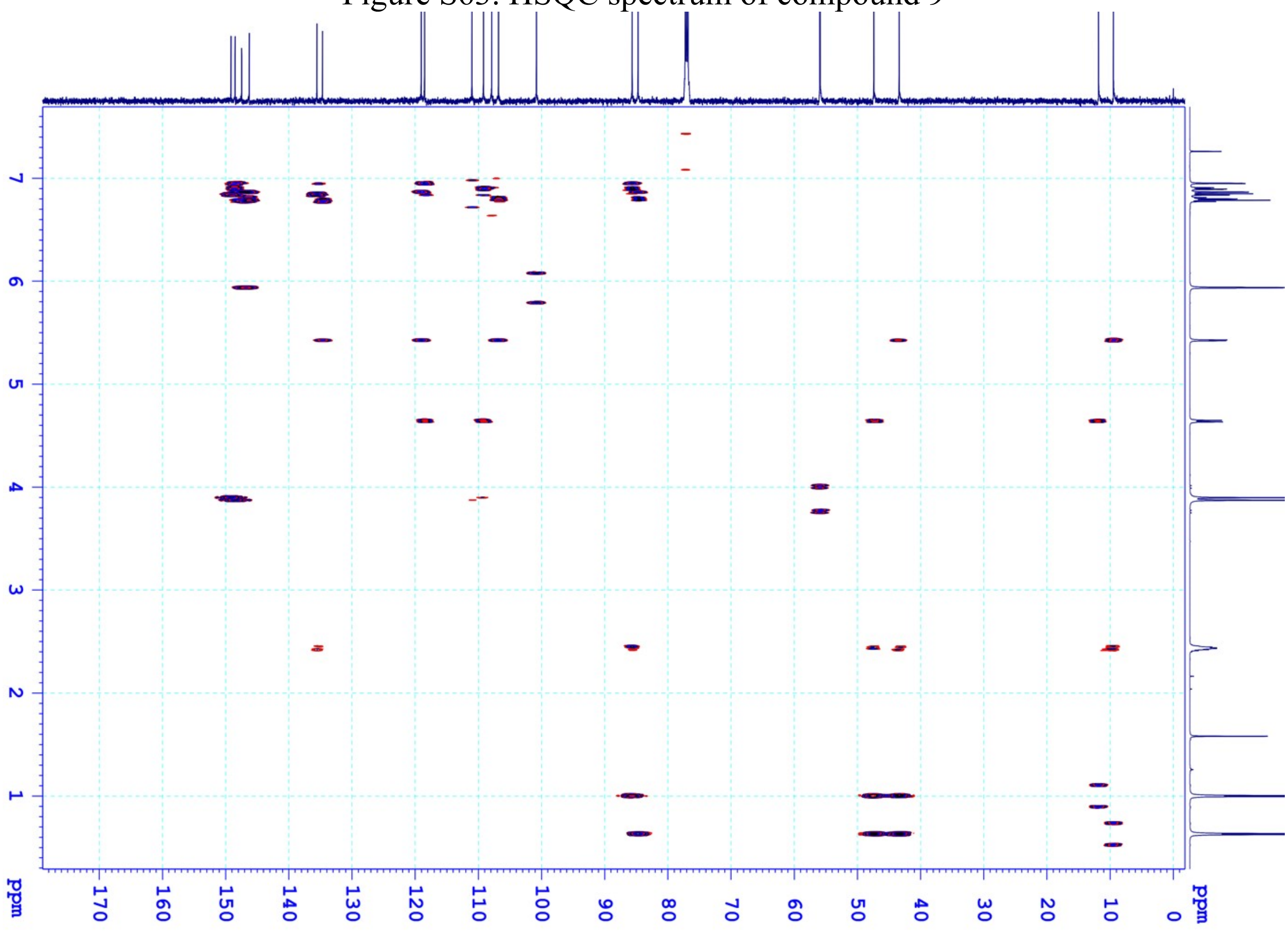

Figure S64. HMBC spectrum of compound 9

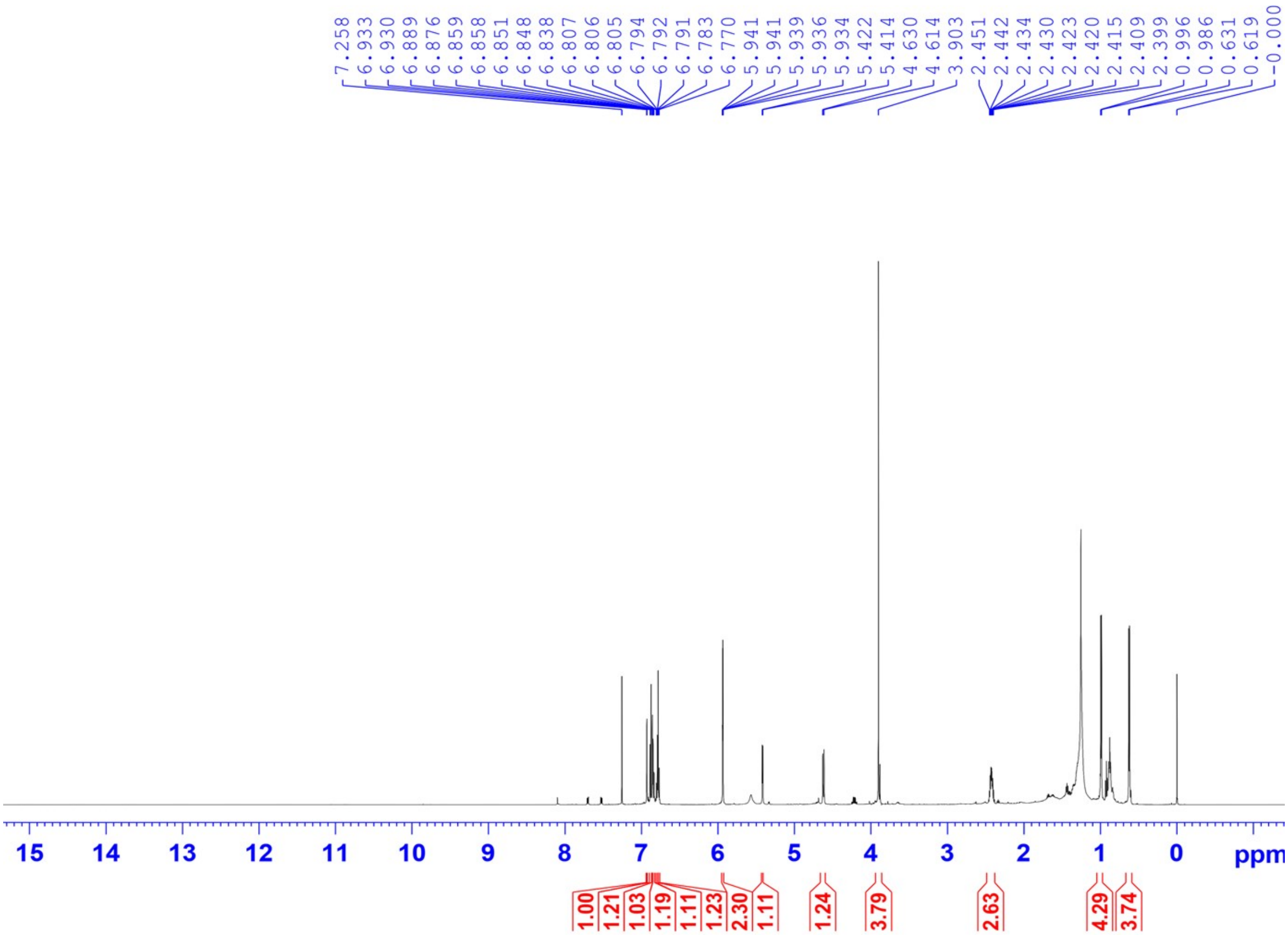

Figure S65. 1H-NMR spectrum of compound 10

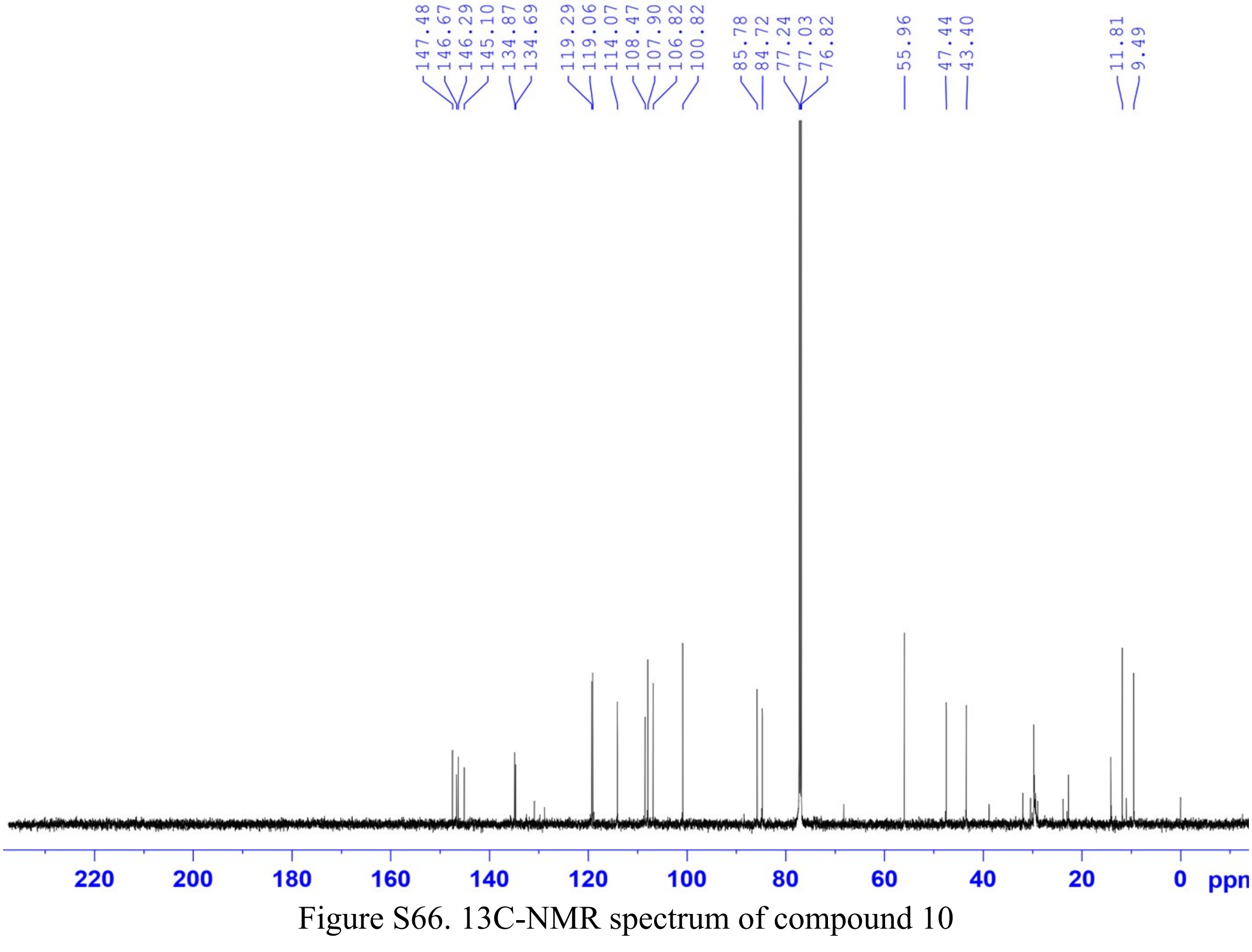

Figure S66. 13C-NMR spectrum of compound 10

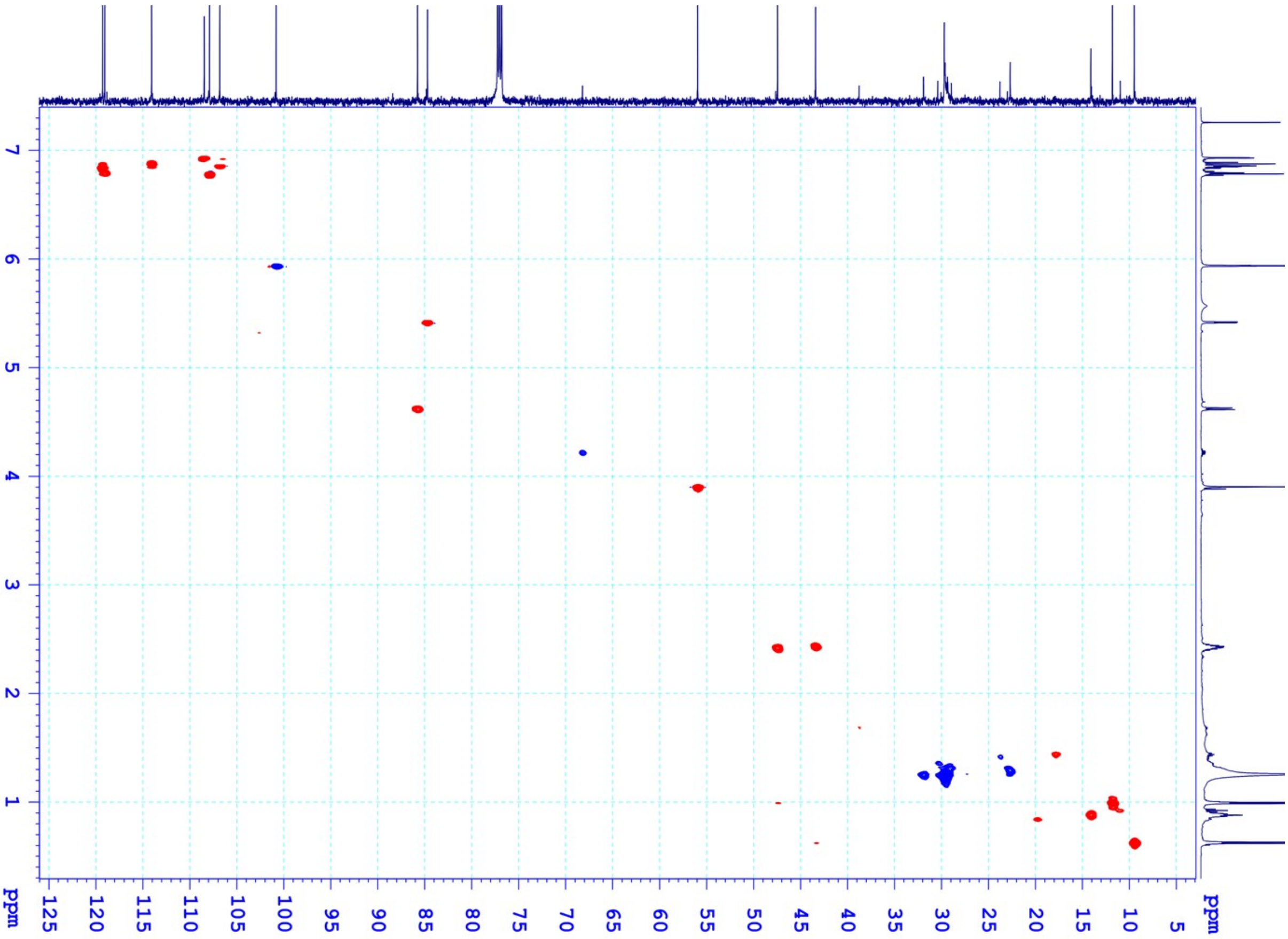

Figure S67. HSQC spectrum of compound 10

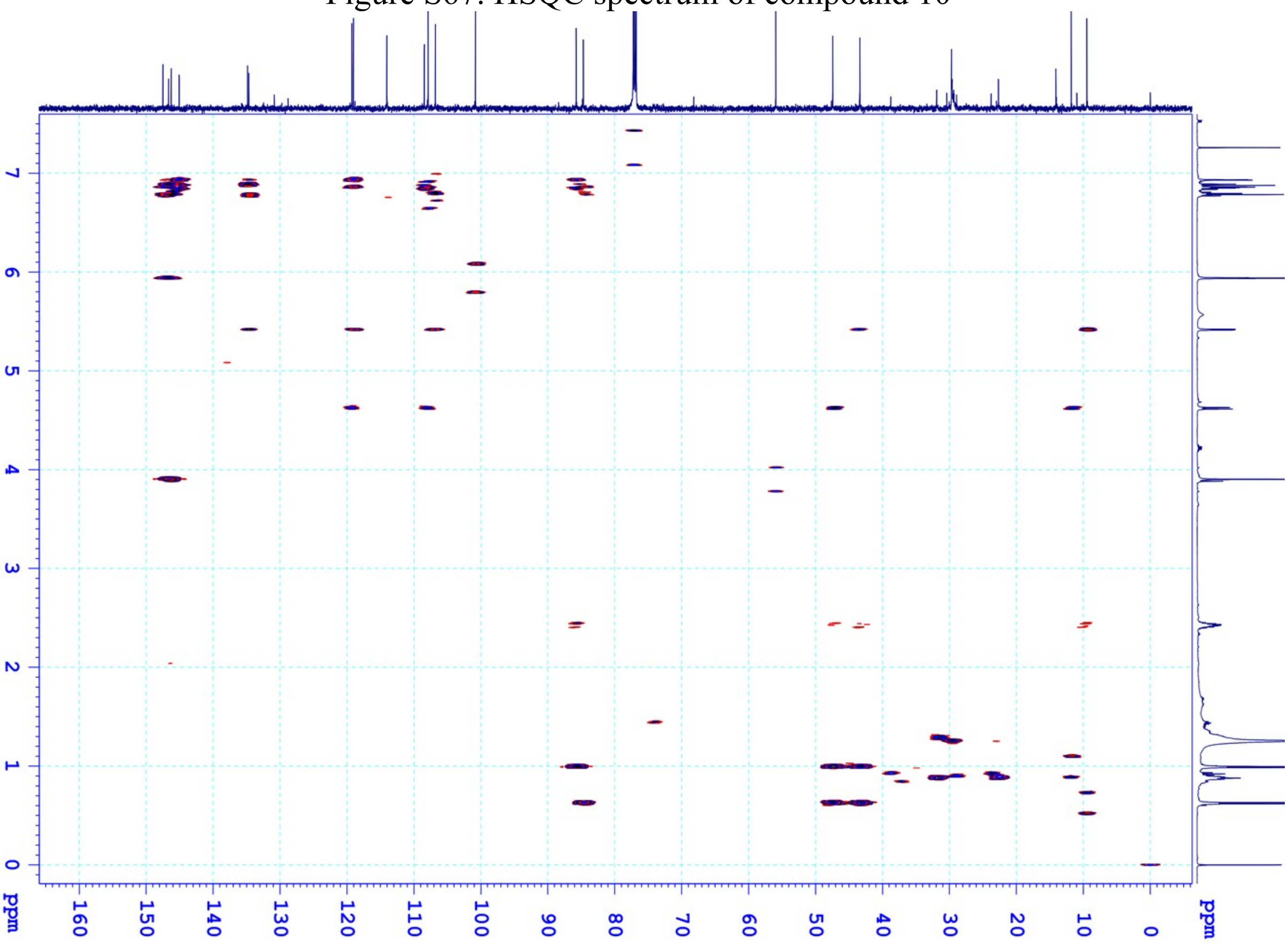

Figure S68. HMBC spectrum of compound 10

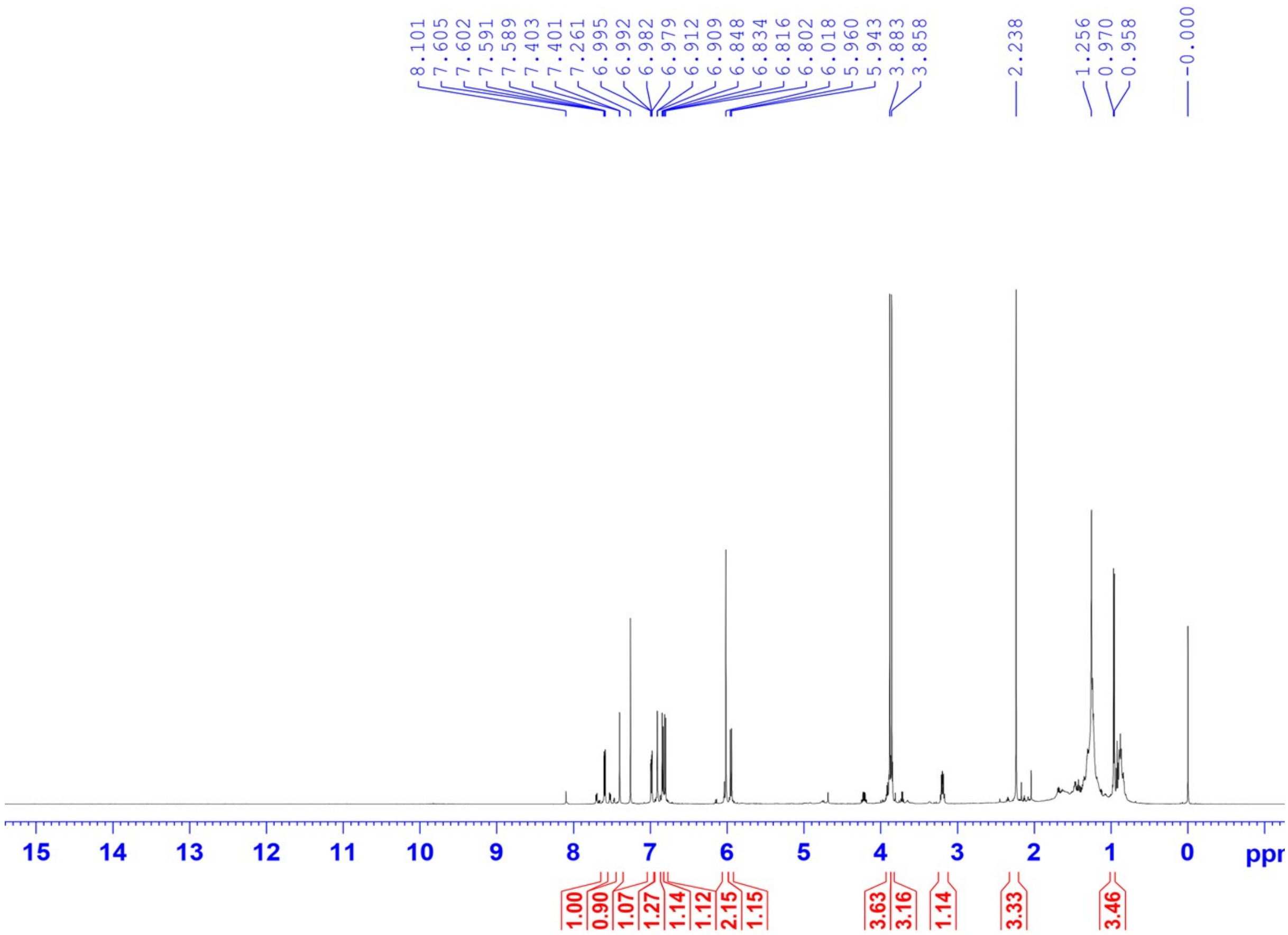

Figure S69. 1H-NMR spectrum of compound 11

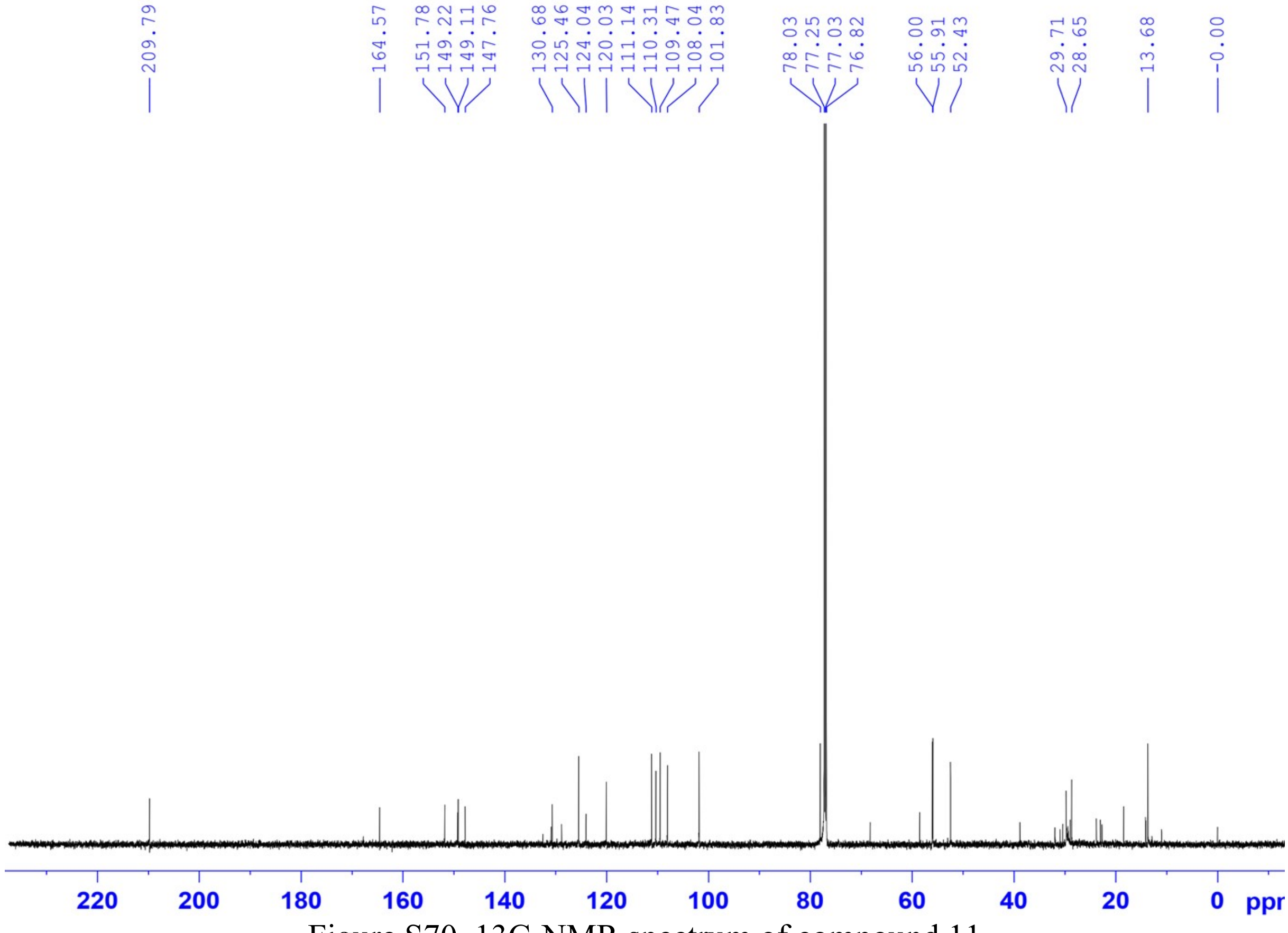

Figure S70. 13C-NMR spectrum of compound 11

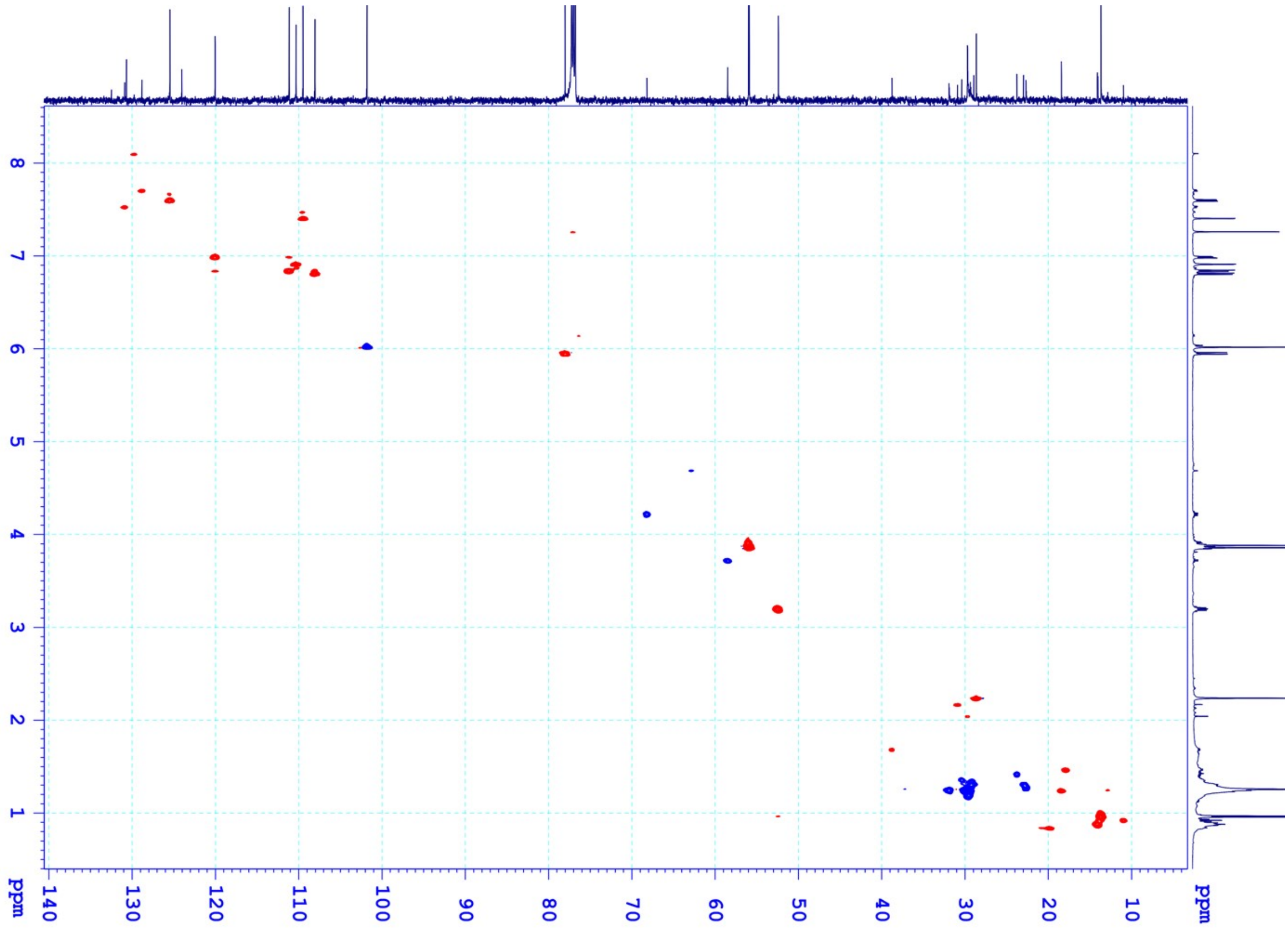

Figure S71. HSQC spectrum of compound 11

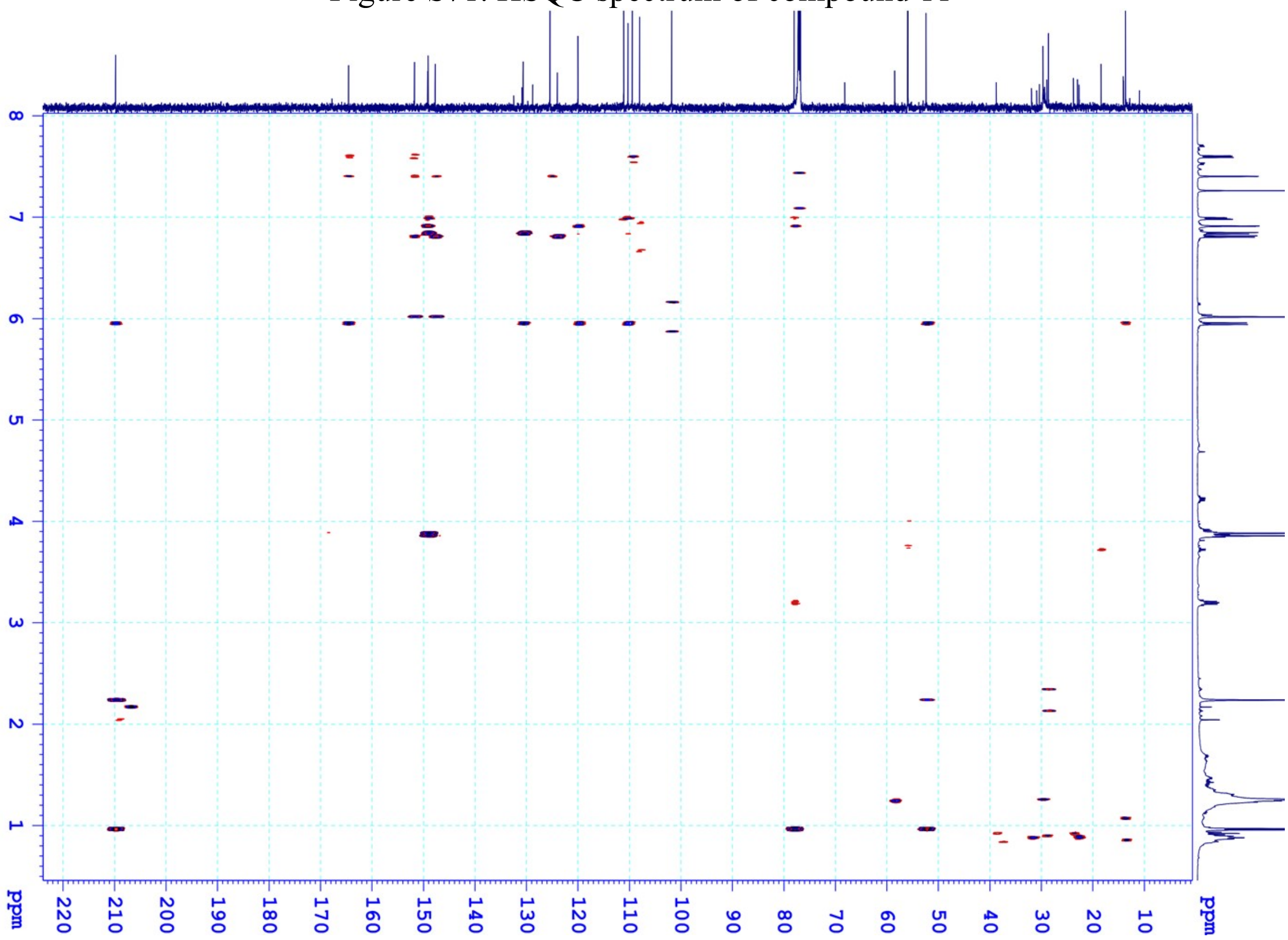

Figure S72. HMBC spectrum of compound 11

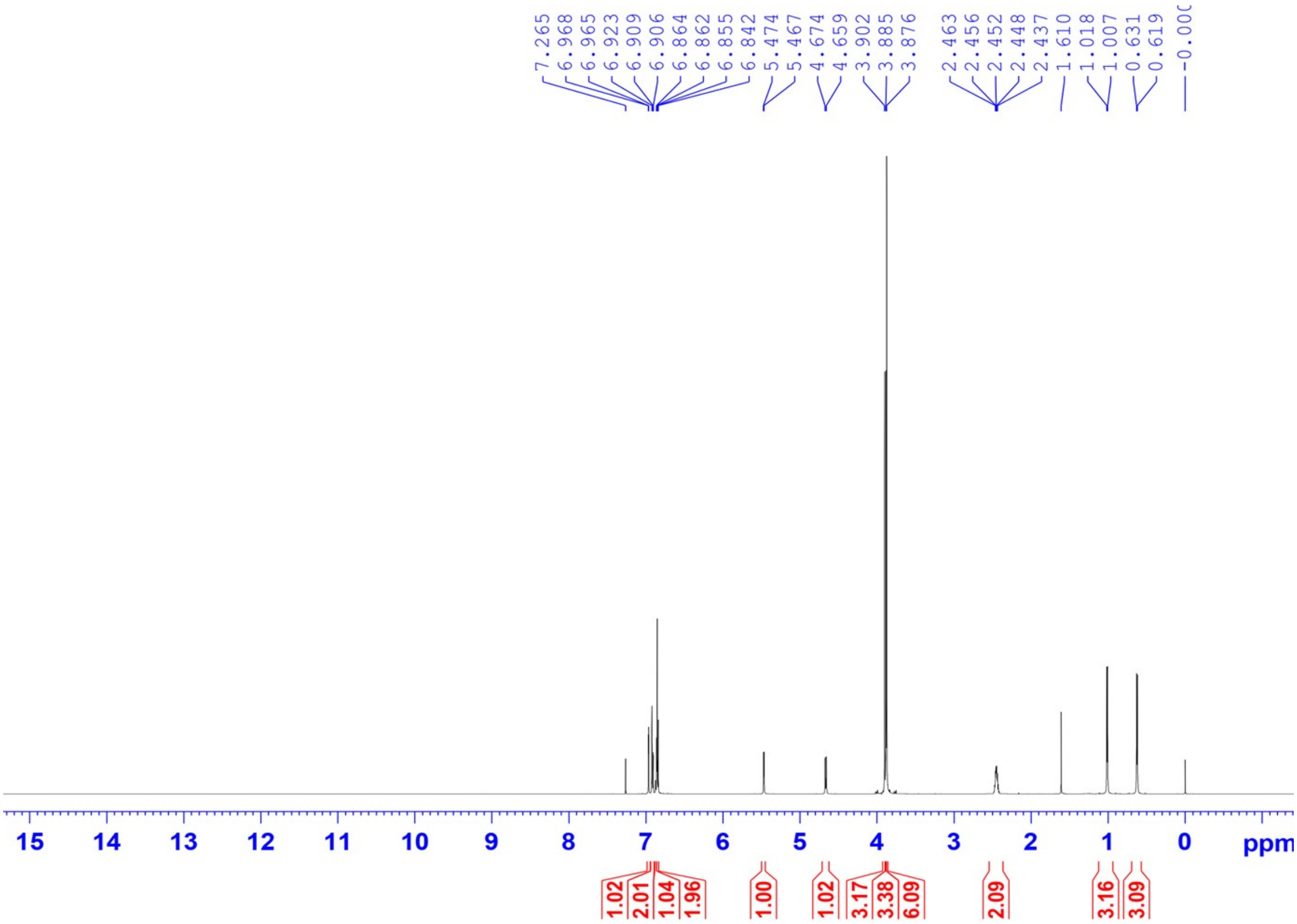

Figure S73. 1H-NMR spectrum of compound 12

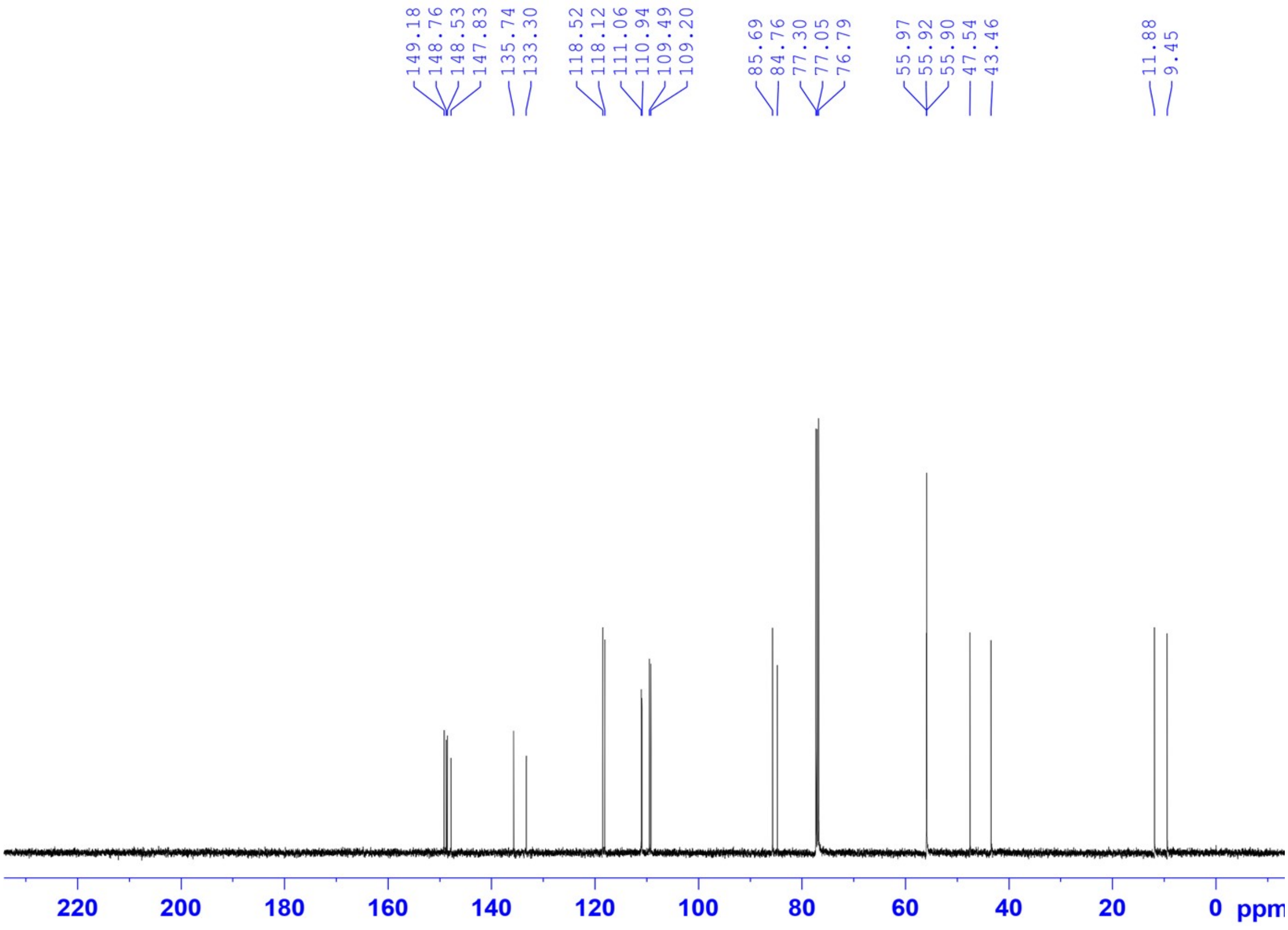

Figure S74. 13C-NMR spectrum of compound 12

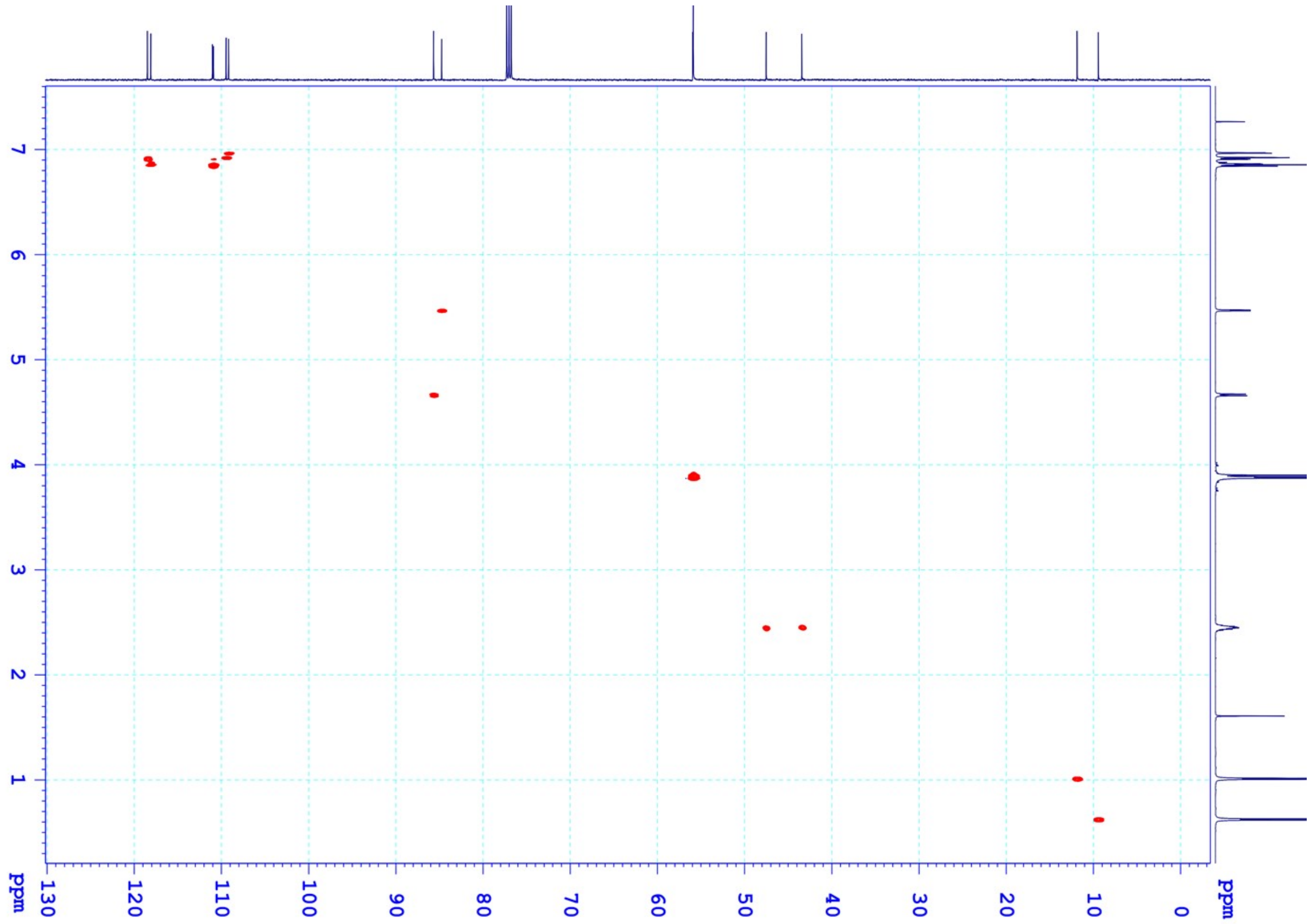

Figure S75. HSQC spectrum of compound 12

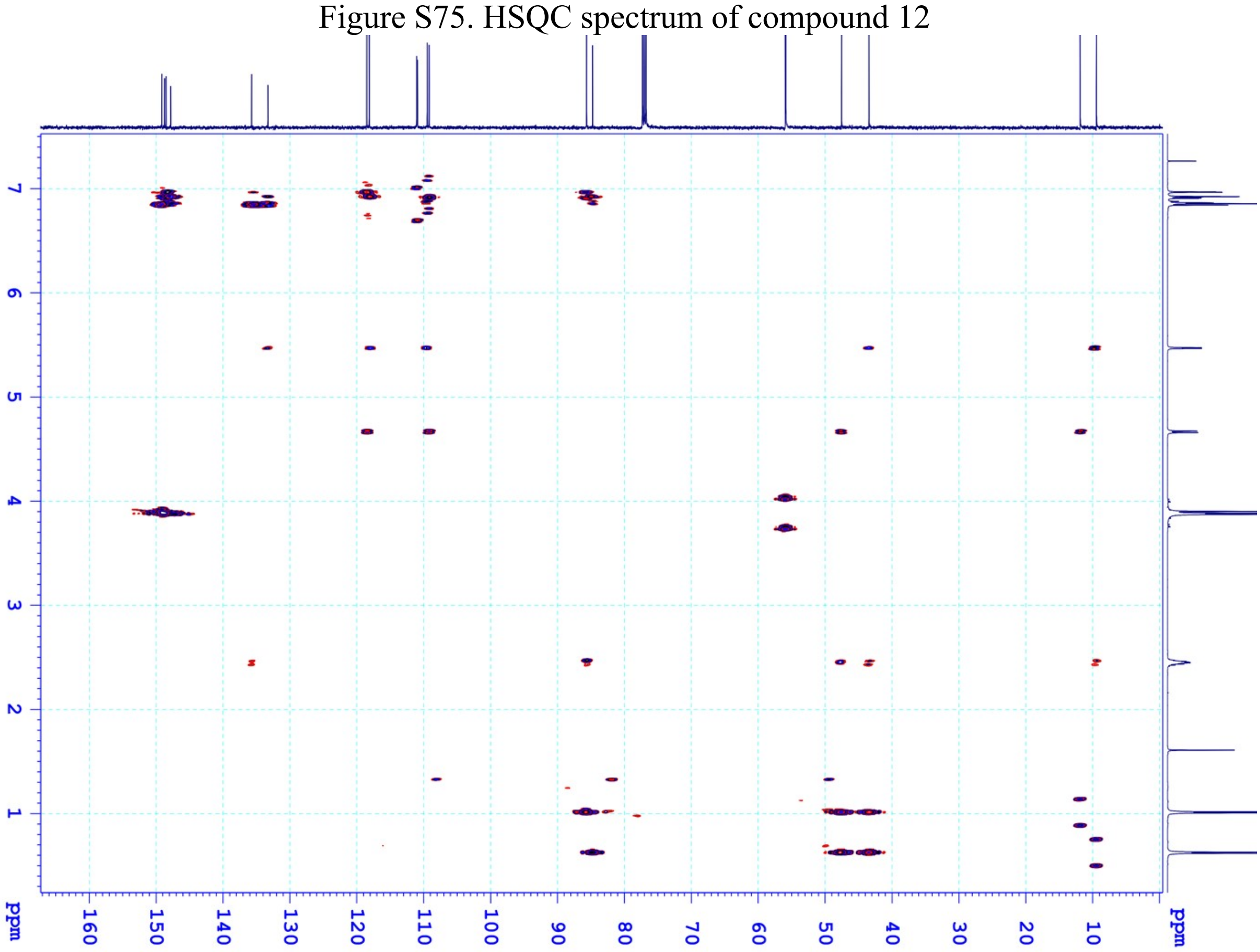

Figure S76. HMBC spectrum of compound 12

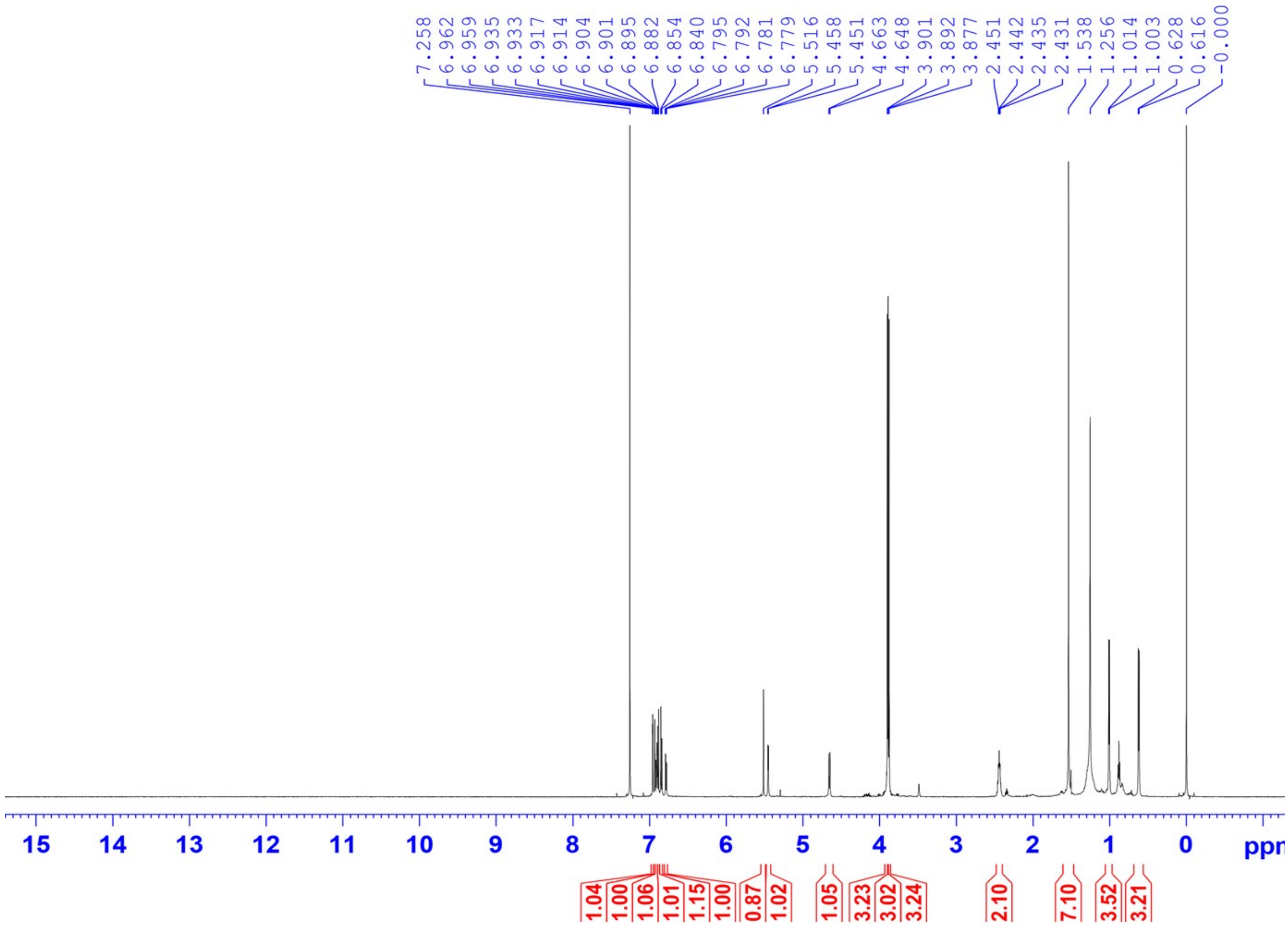

Figure S77. 1H-NMR spectrum of compound 13

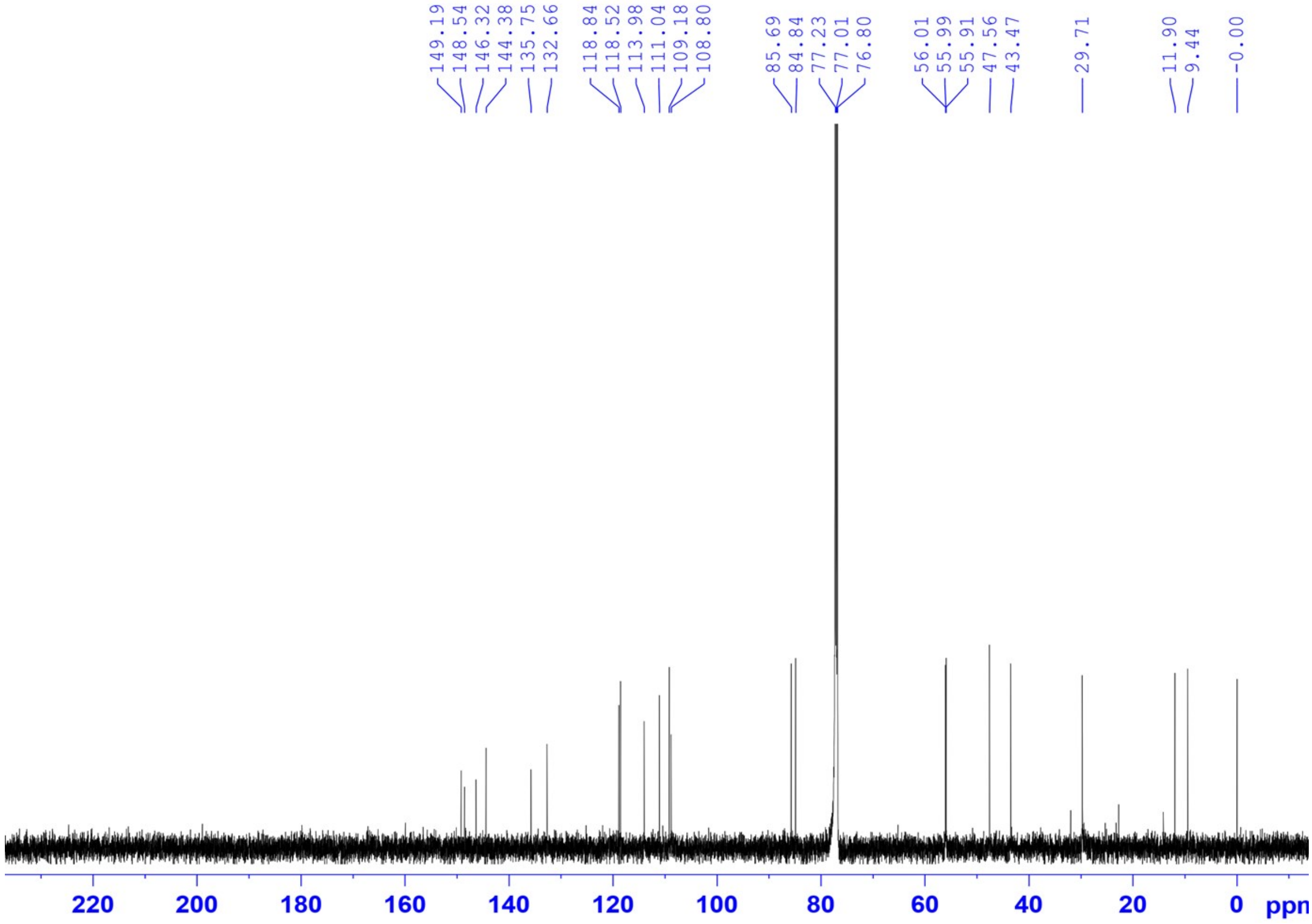

Figure S78. 13C-NMR spectrum of compound 13

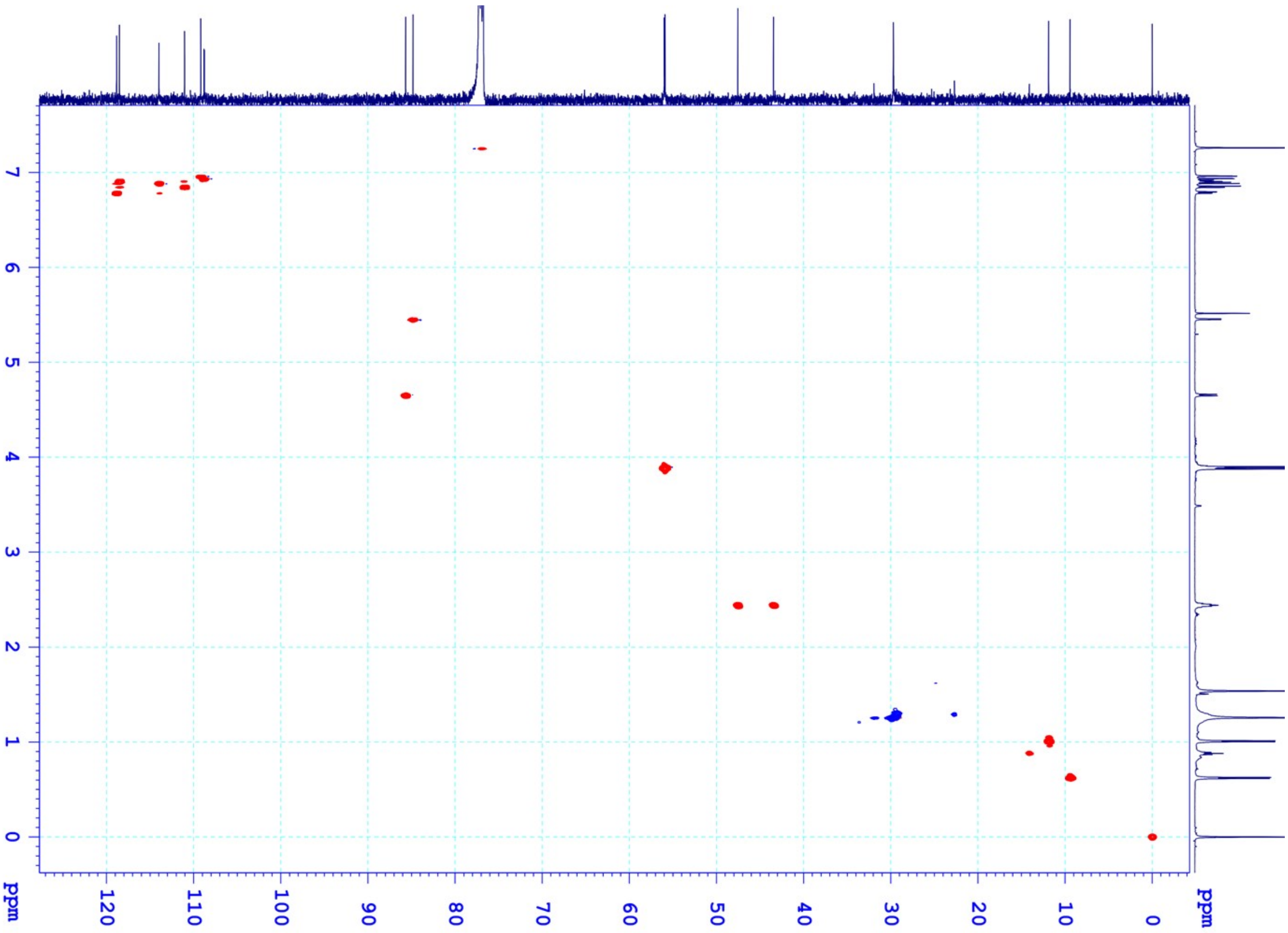

Figure S79. HSQC spectrum of compound 13

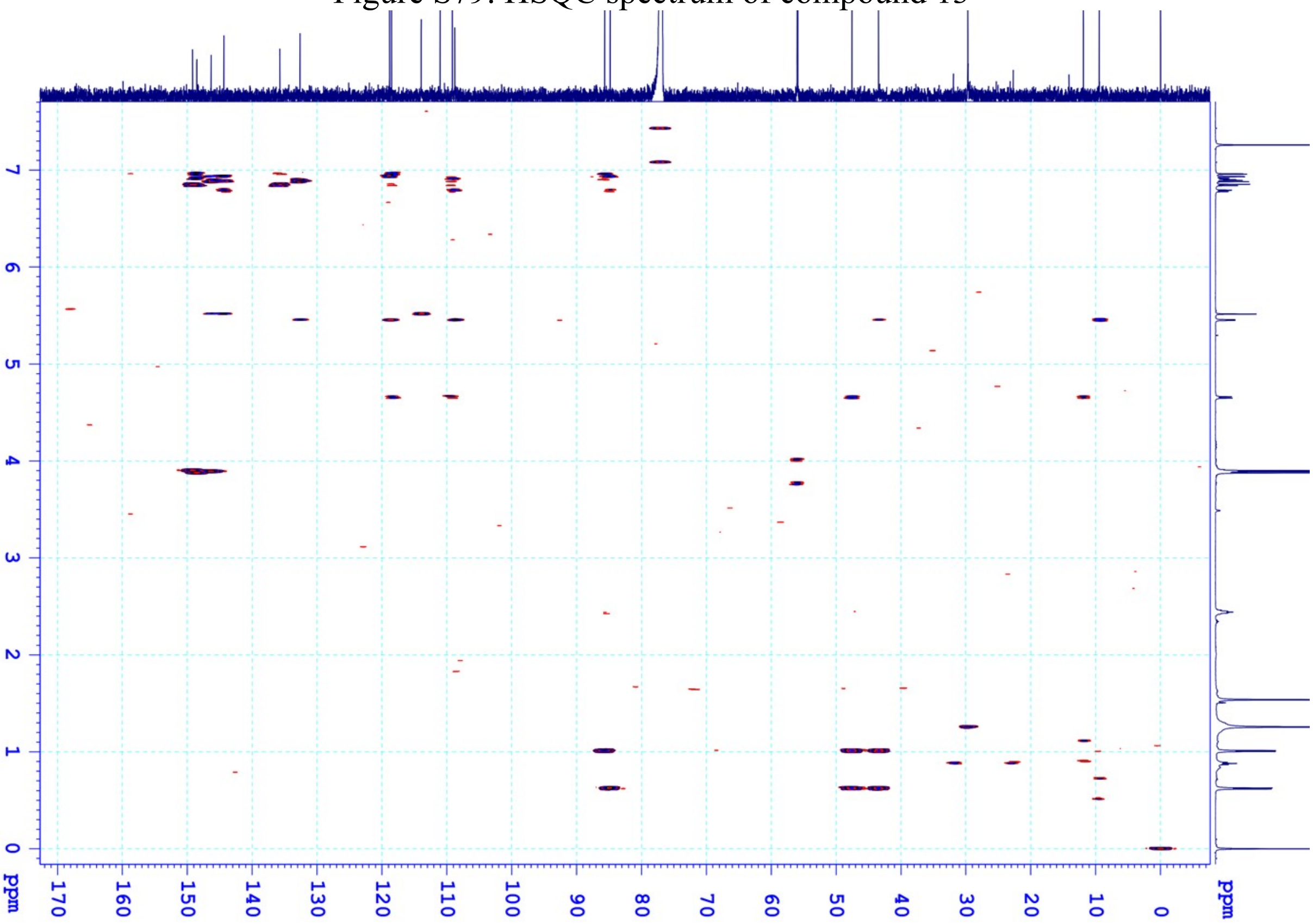

Figure S80. HMBC spectrum of compound 13

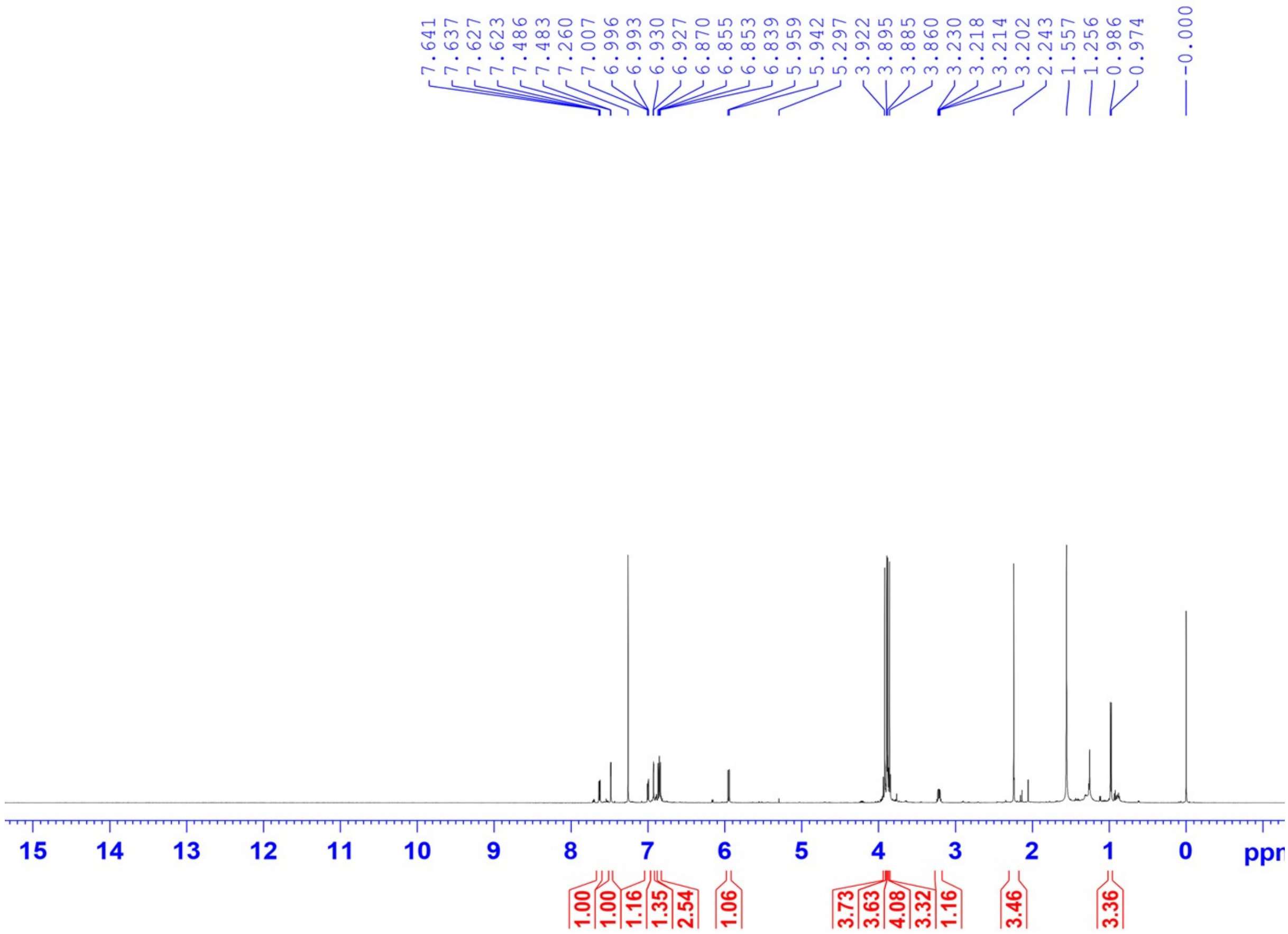

Figure S81. 1H-NMR spectrum of compound 14

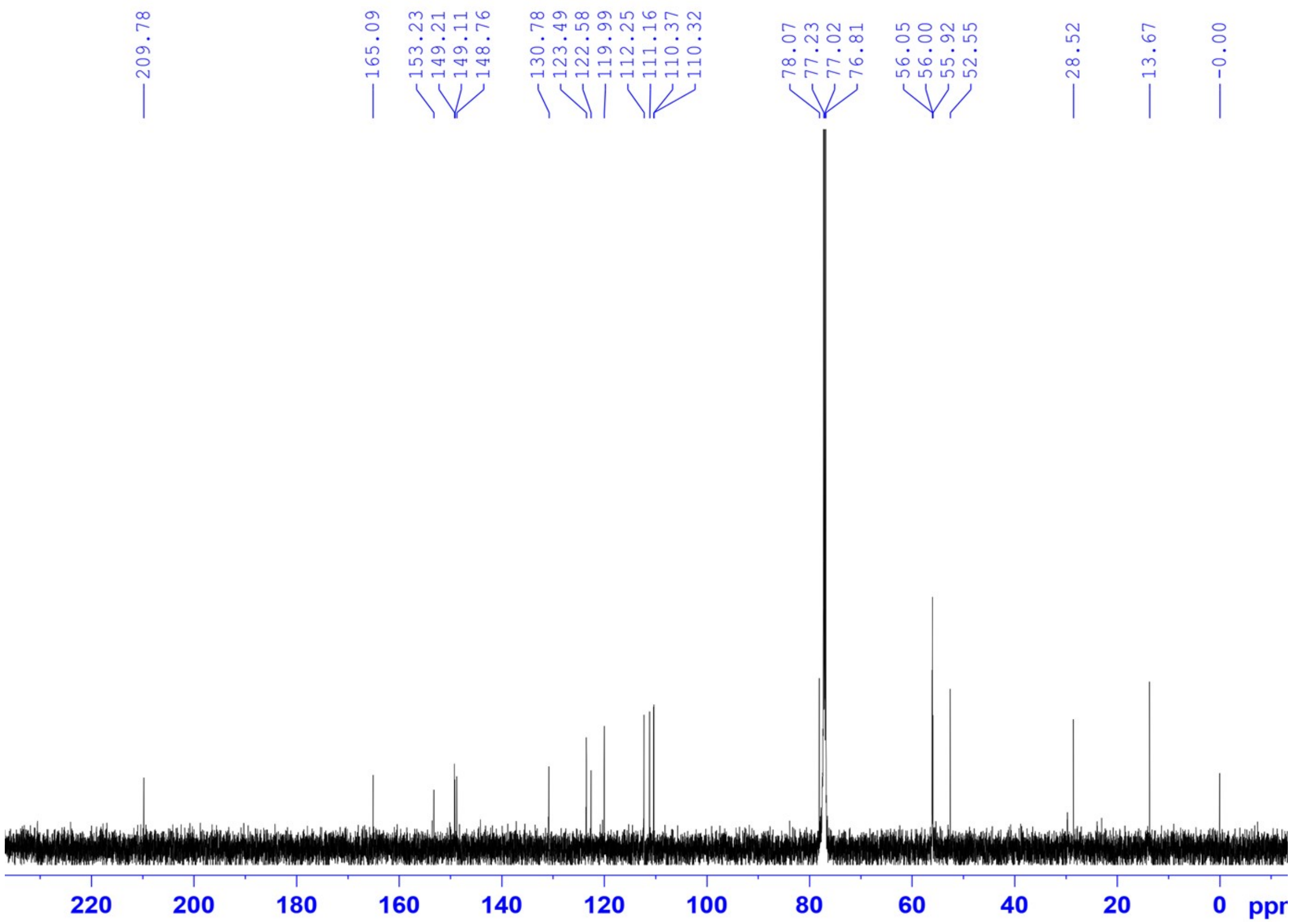

Figure S82. 13C-NMR spectrum of compound 14

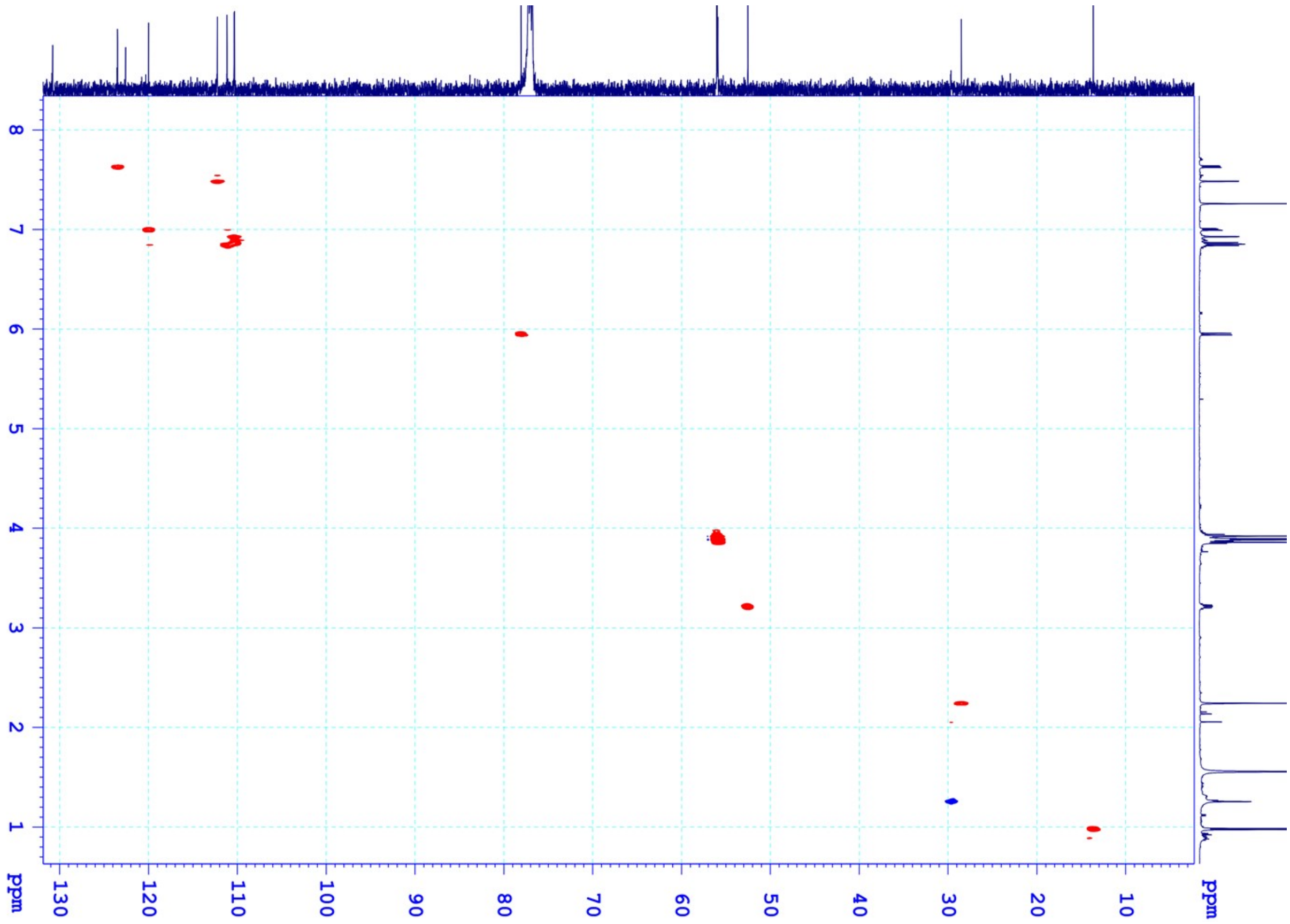

Figure S83. HSQC spectrum of compound 14

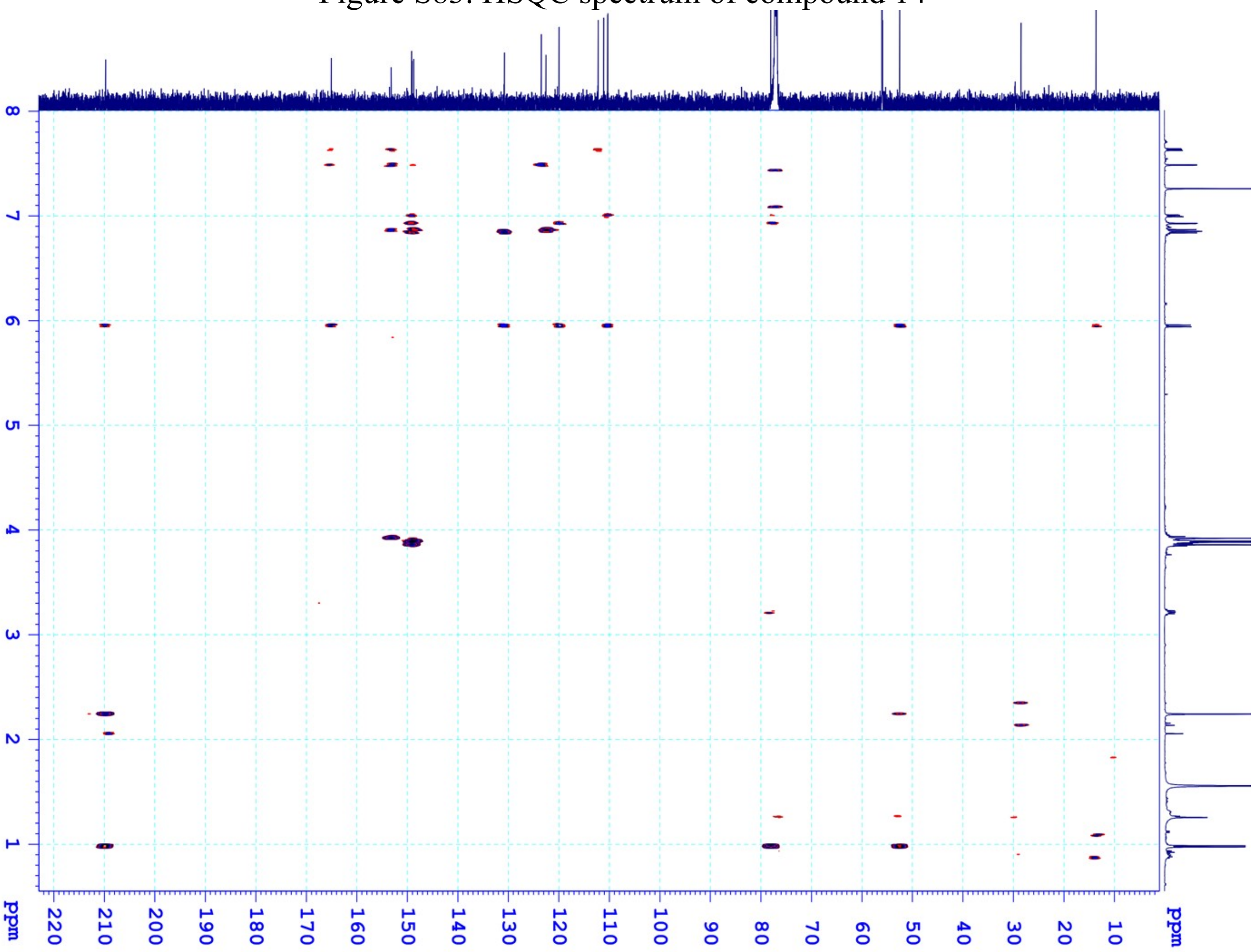

Figure S84. HMBC spectrum of compound 14

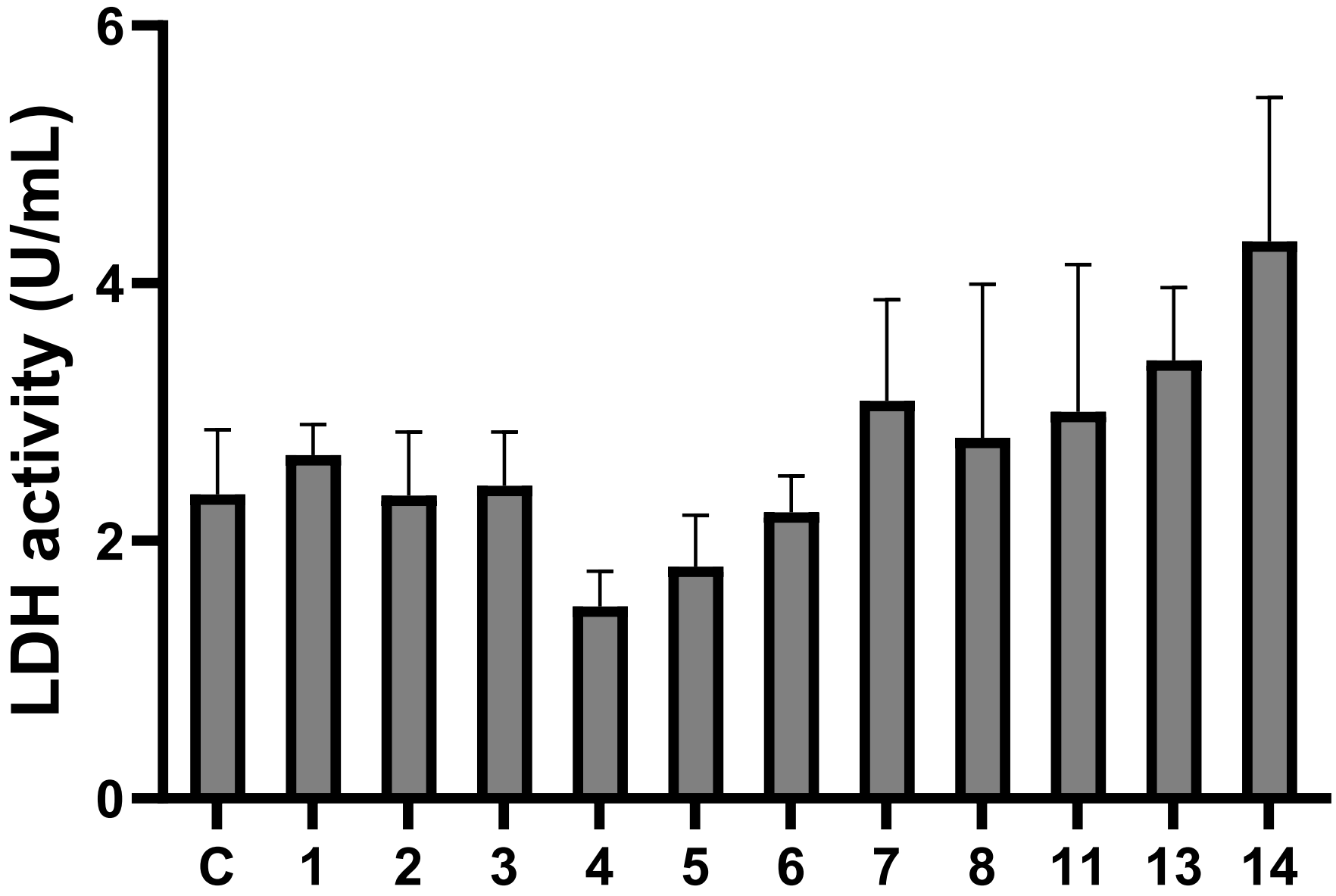

Figure S85. Effects of isolated compounds on LDH release in Raw 264.7 macrophages.

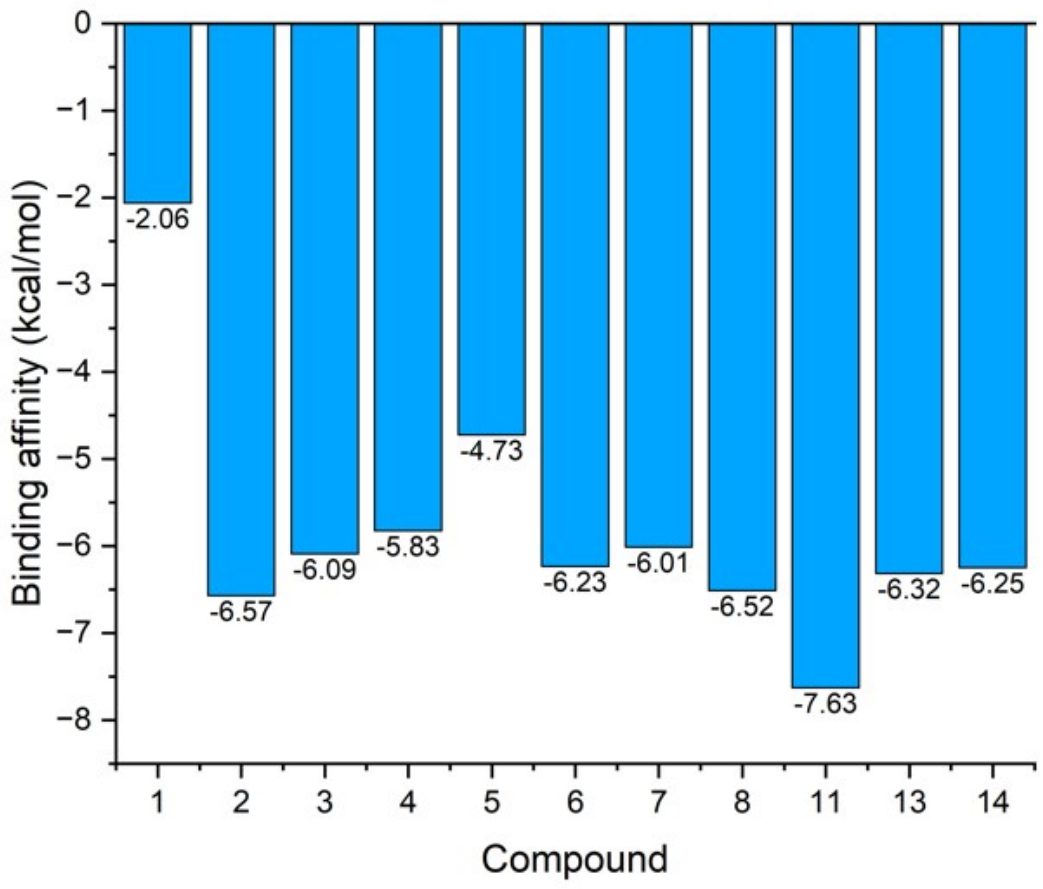

COX-2 (PDB ID: 5IKR)

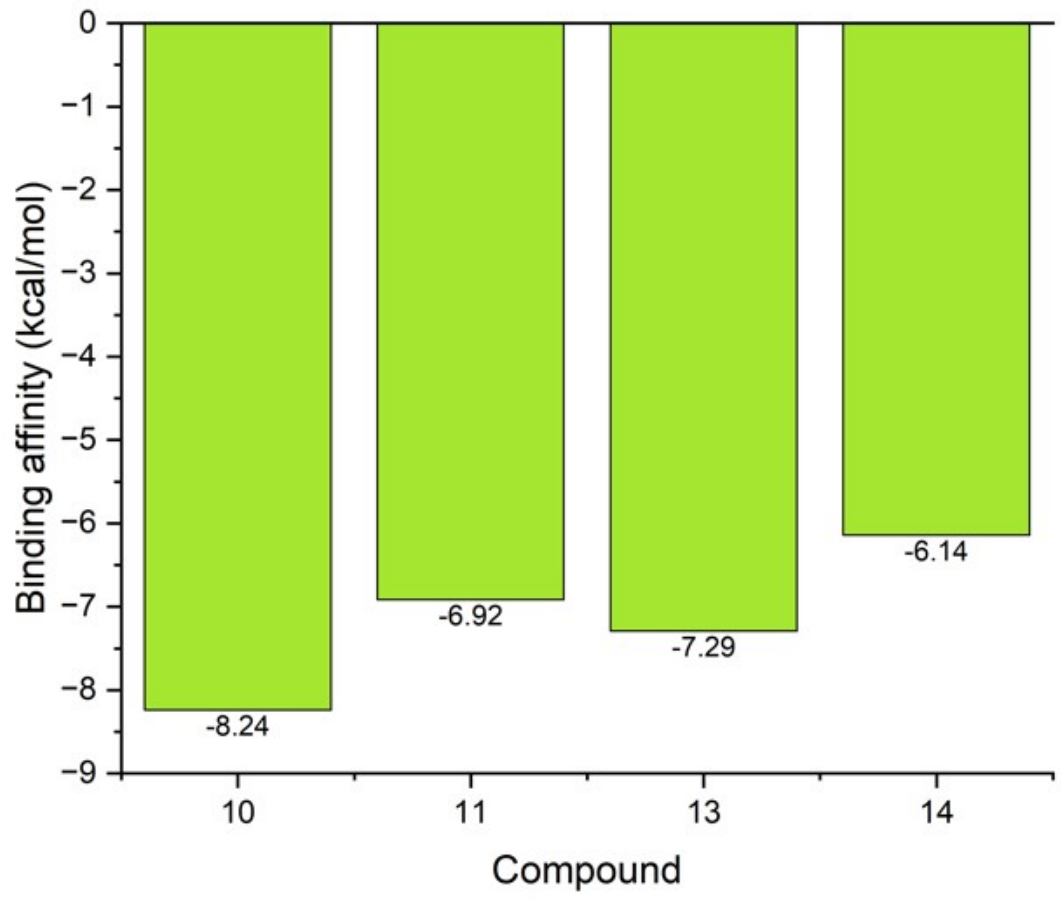

α-glucosidase (PDB ID: 2QMJ)

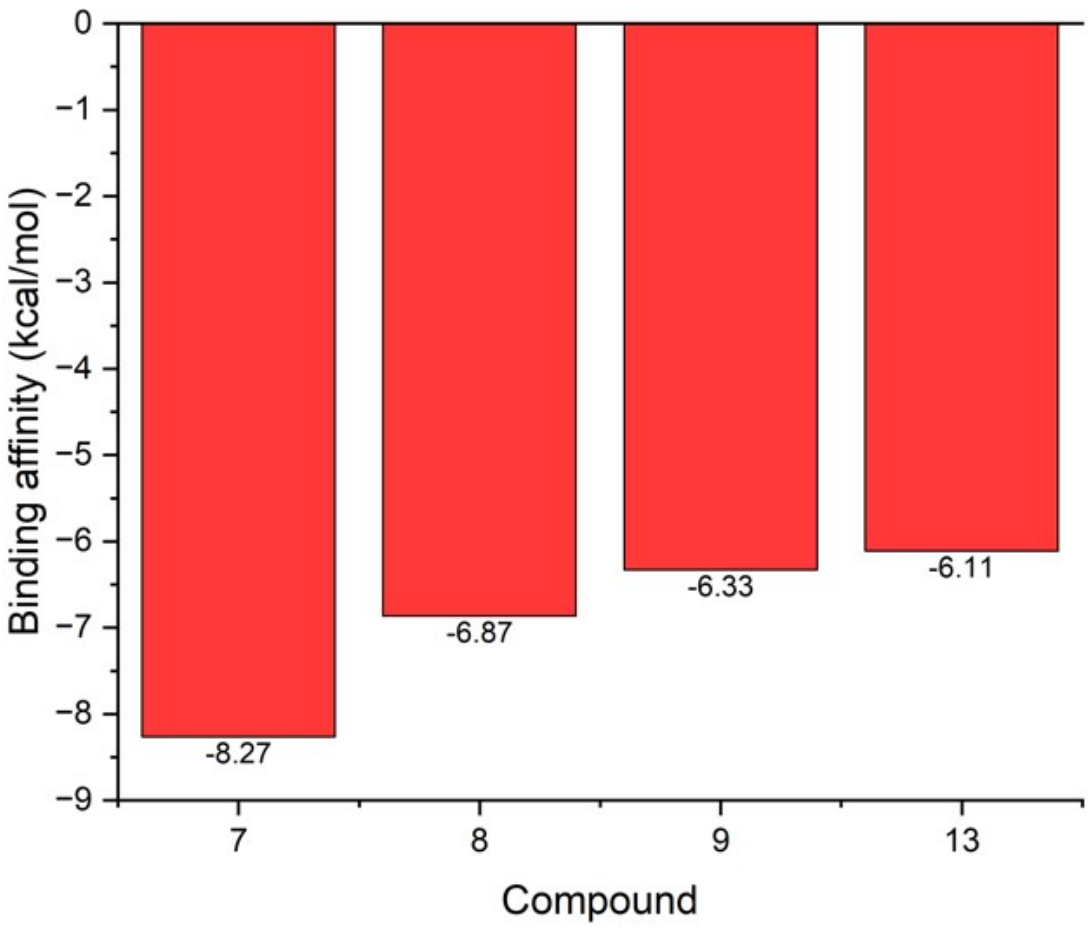

Xanthine oxidase (PDB ID: 3NVY)

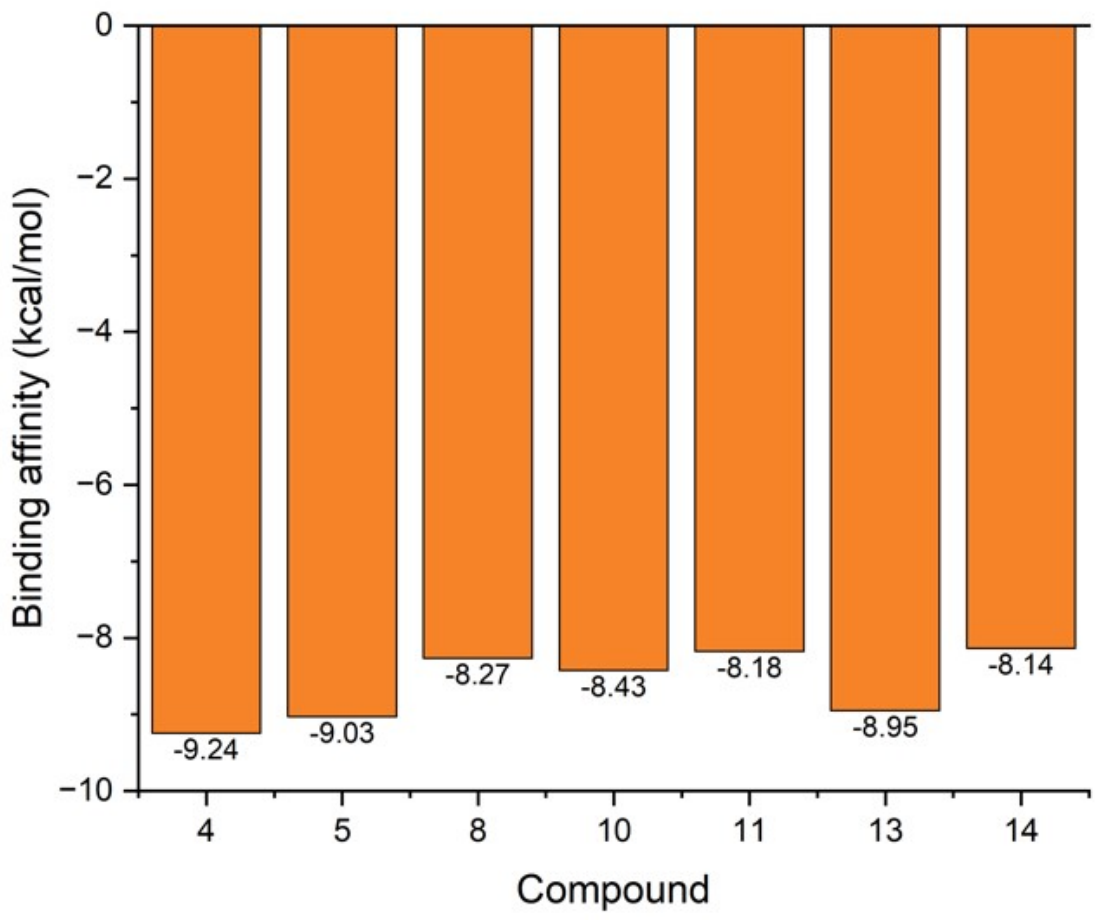

Keap1 (PDB ID: 4L7B)

Figure S86. Binding affinity (kcal/mol) of selected compounds with their corresponding target proteins.

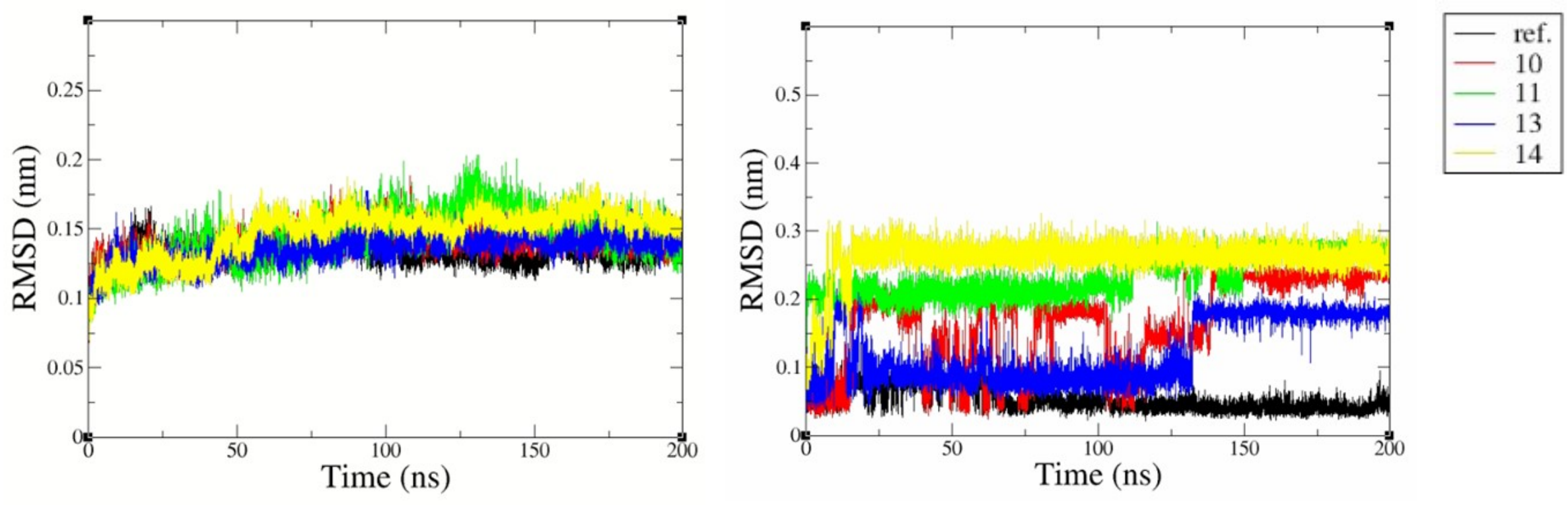

Backbone **Ligand**

2MQJ

Figure S87. Root-mean-square deviation (RMSD) profiles of the protein backbone and ligands during 200 ns molecular dynamics simulations of α-glucosidase (PDB ID: 2QMJ) complexes.

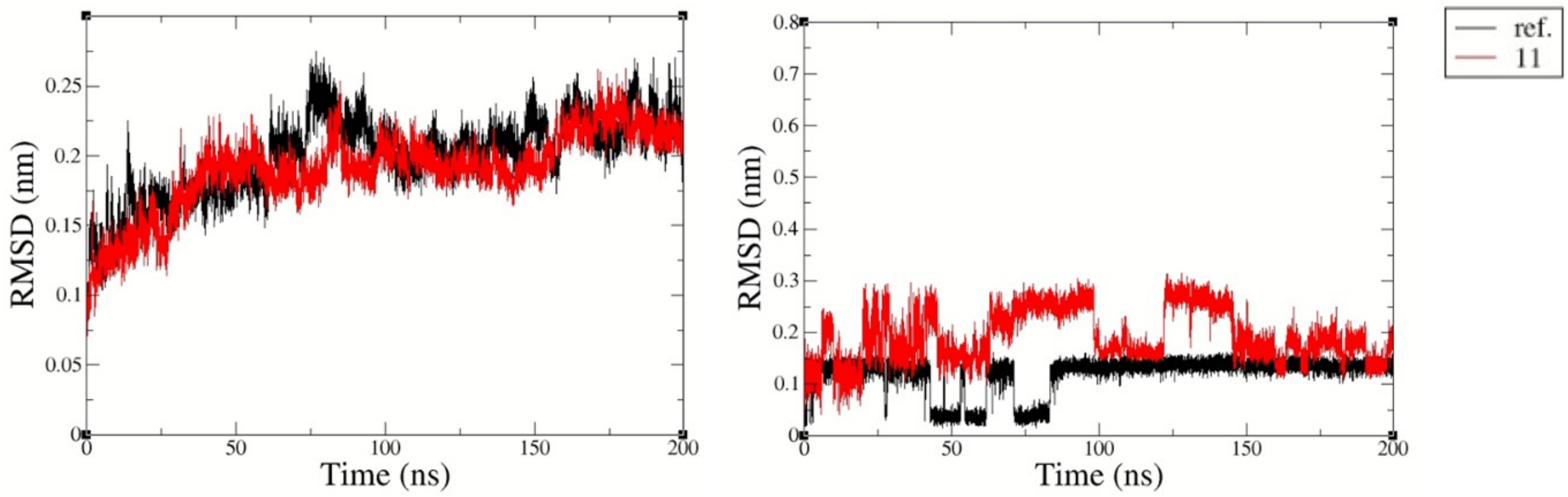

Backbone **Ligand**

5IKR

Figure S88. Root-mean-square deviation (RMSD) profiles of the protein backbone and ligands during 200 ns molecular dynamics simulations of COX-2 (PDB ID: 5IKR) complexes.

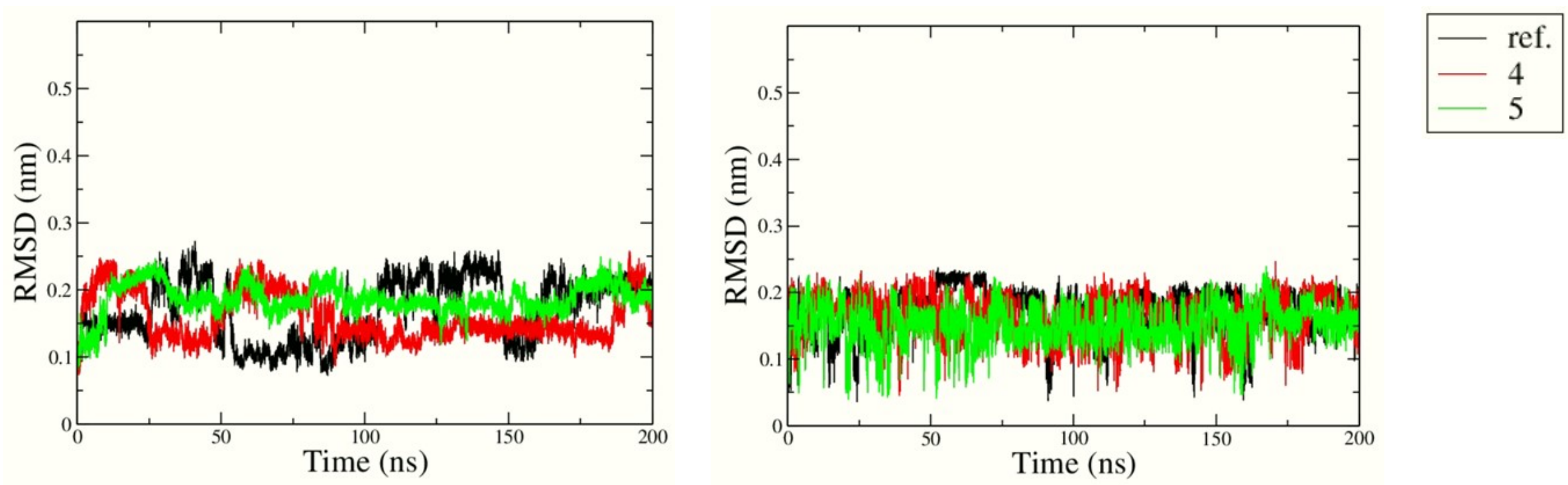

Backbone **Ligand**

4L7B

Figure S89. Root-mean-square deviation (RMSD) profiles of the protein backbone and ligands during 200 ns molecular dynamics simulations of Keap1 (PDB ID: 4L7B) complexes.

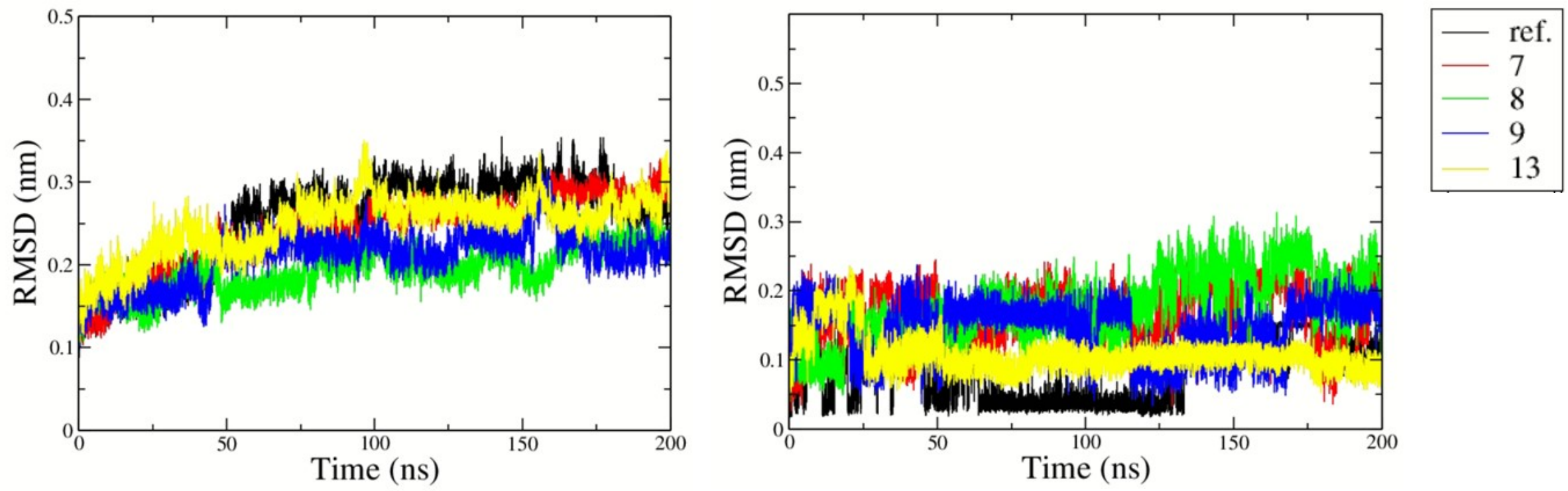

Backbone **Ligand**

3NVY

Figure S90. Root-mean-square deviation (RMSD) profiles of the protein backbone and ligands during 200 ns molecular dynamics simulations of XO (PDB ID: 3NVY) complexes.

Compound **1** (220 nm) 0-25 min: 30-100% ACN/ 0.1%FA in water

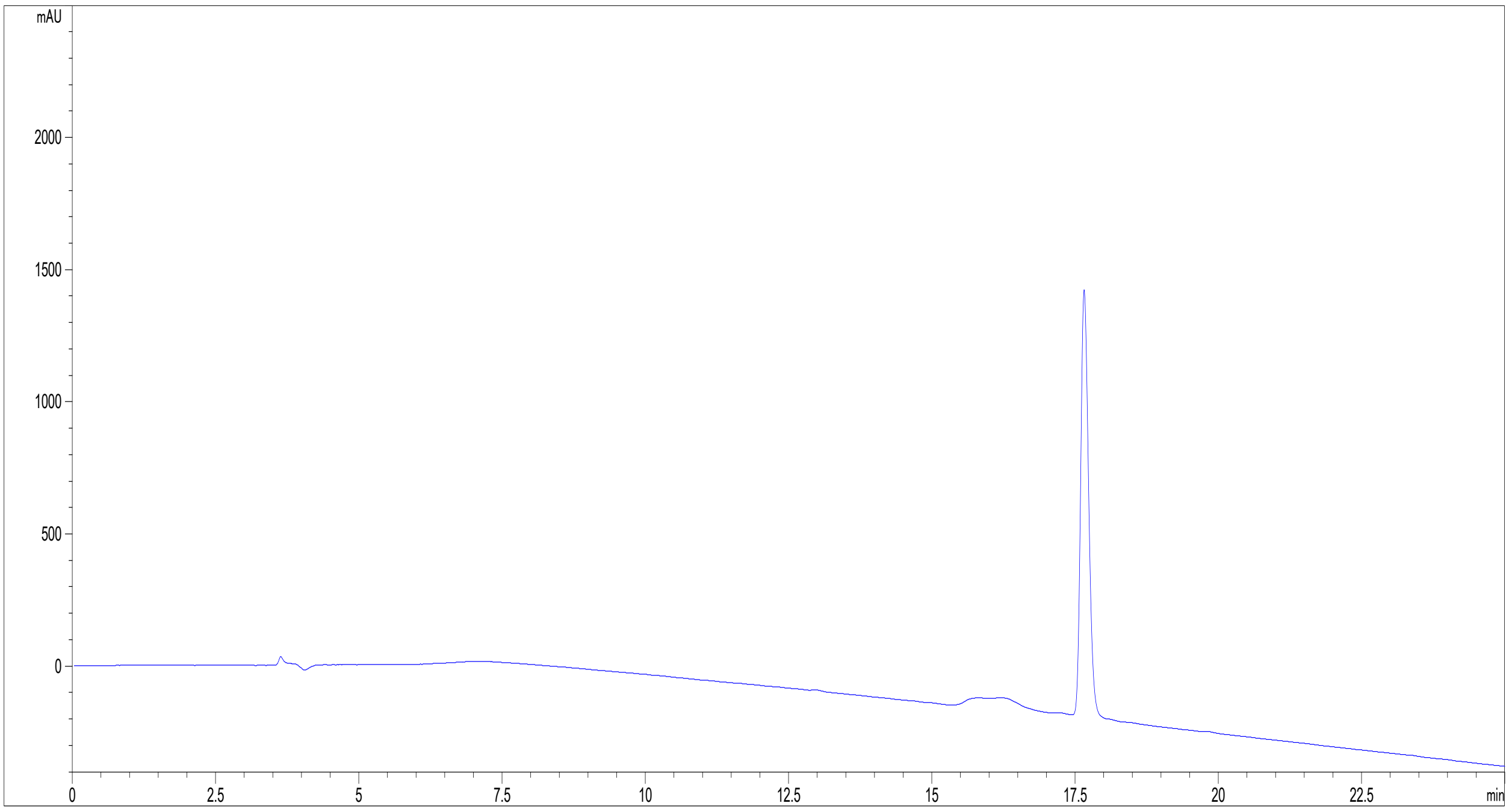

Compound **2** (205 nm) 0-20 min: 52% ACN/ 0.1%FA in water

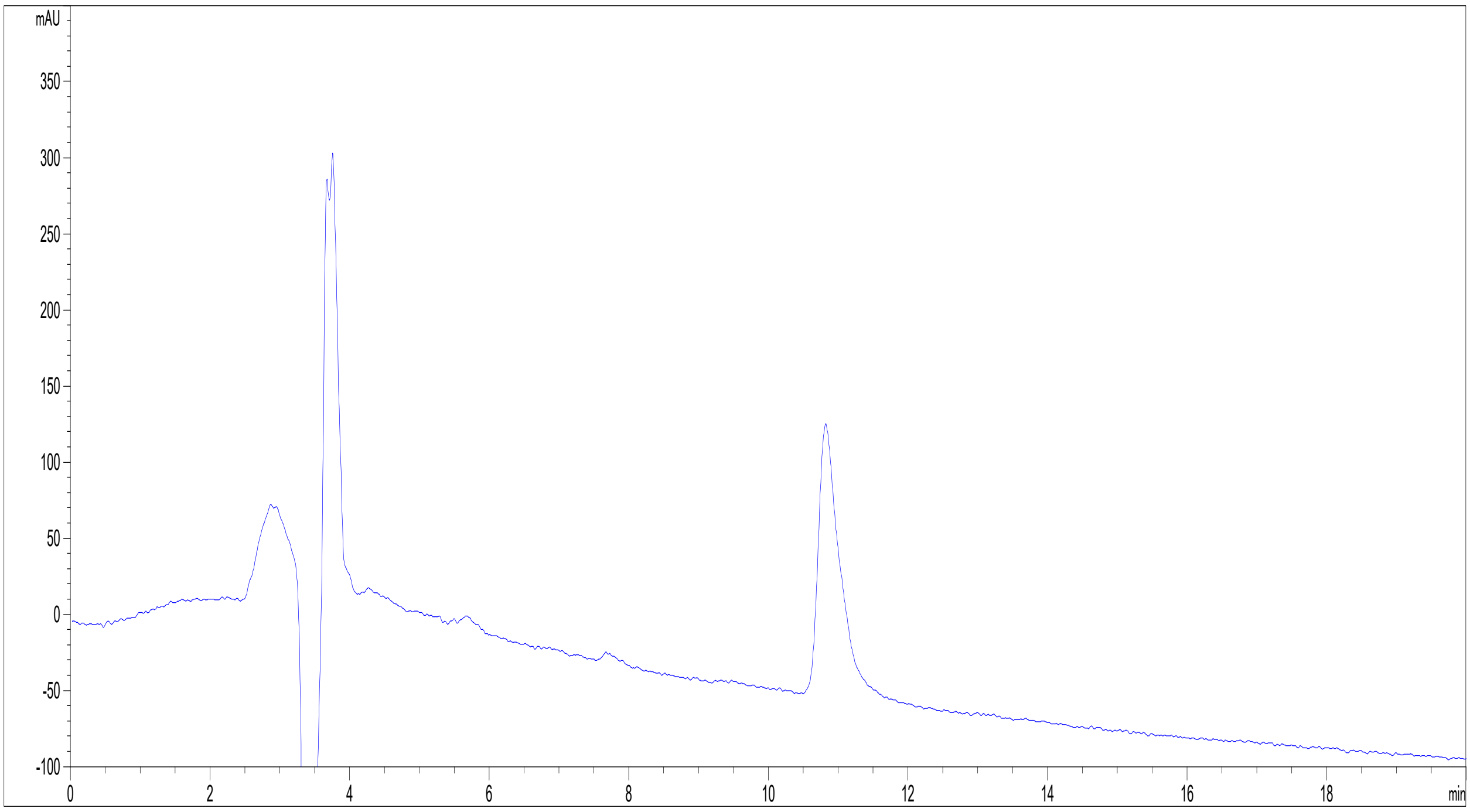

Compound **3** (254 nm) 0-25 min: 30-100% ACN/ 0.1%FA in water

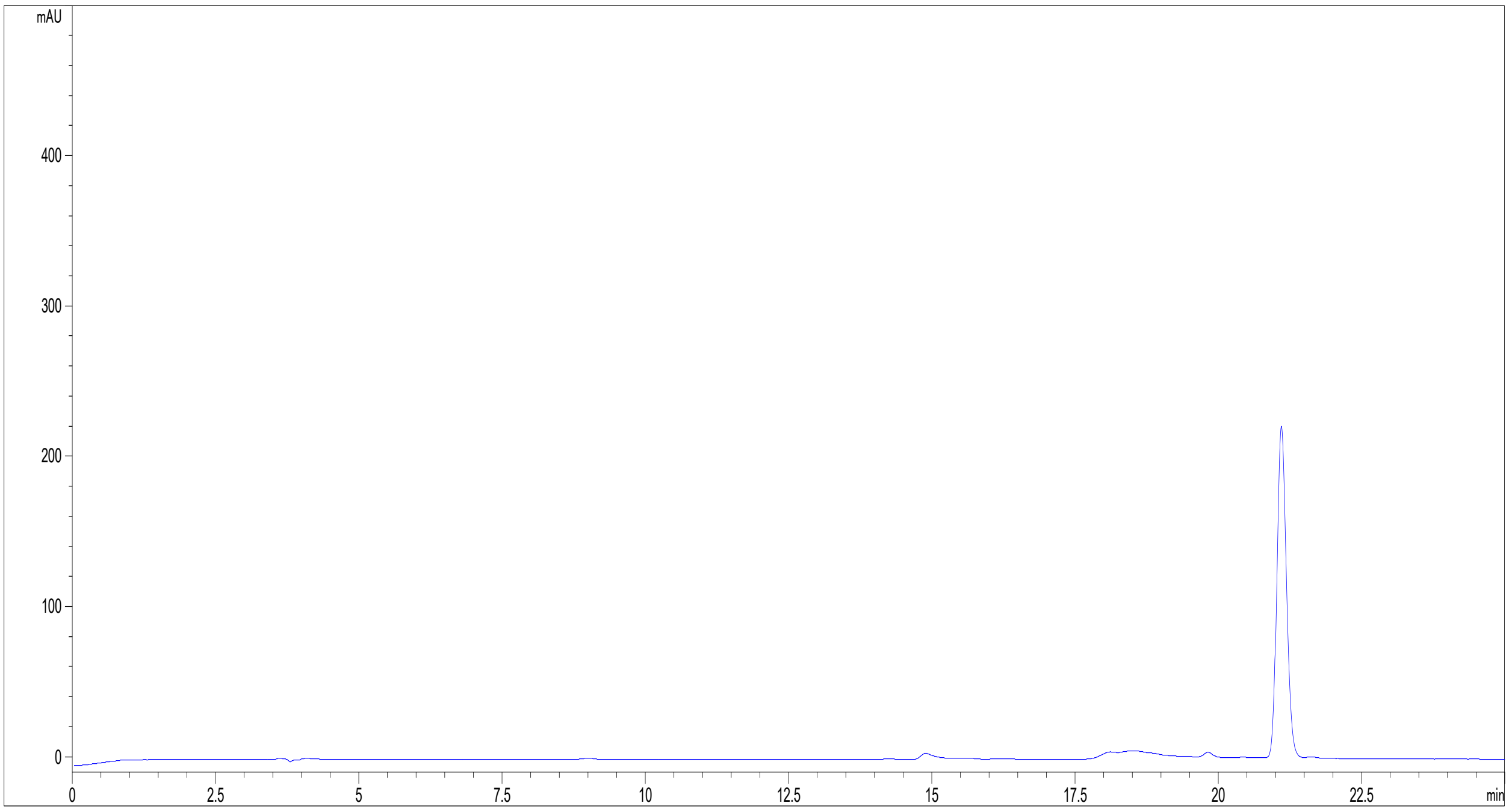

Compound **4** (254 nm) 0-25 min: 30-100% ACN/ 0.1%FA in water

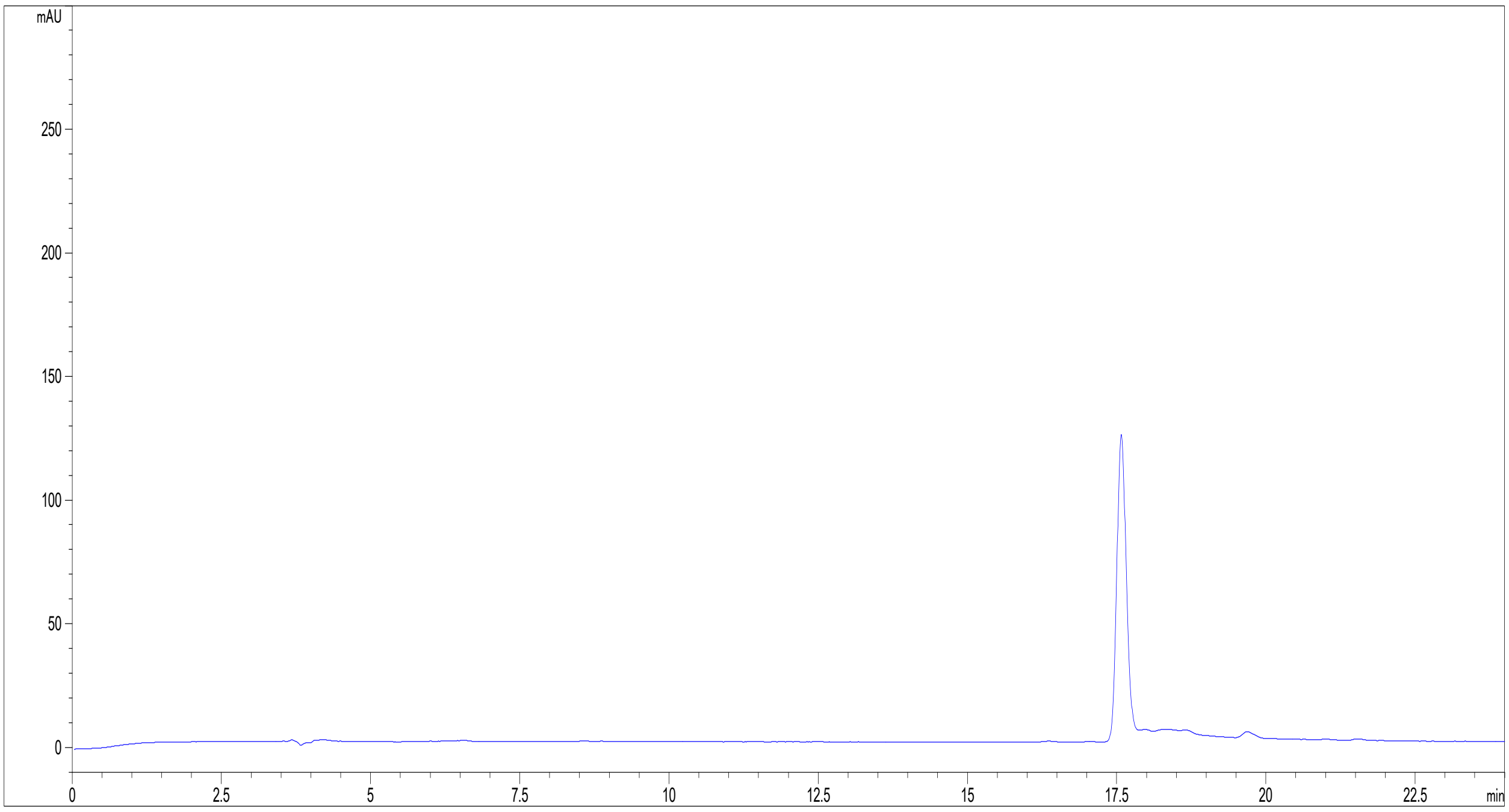

Compound **5** (254 nm) 0-25 min: 40-90% ACN/ 0.1%FA in water

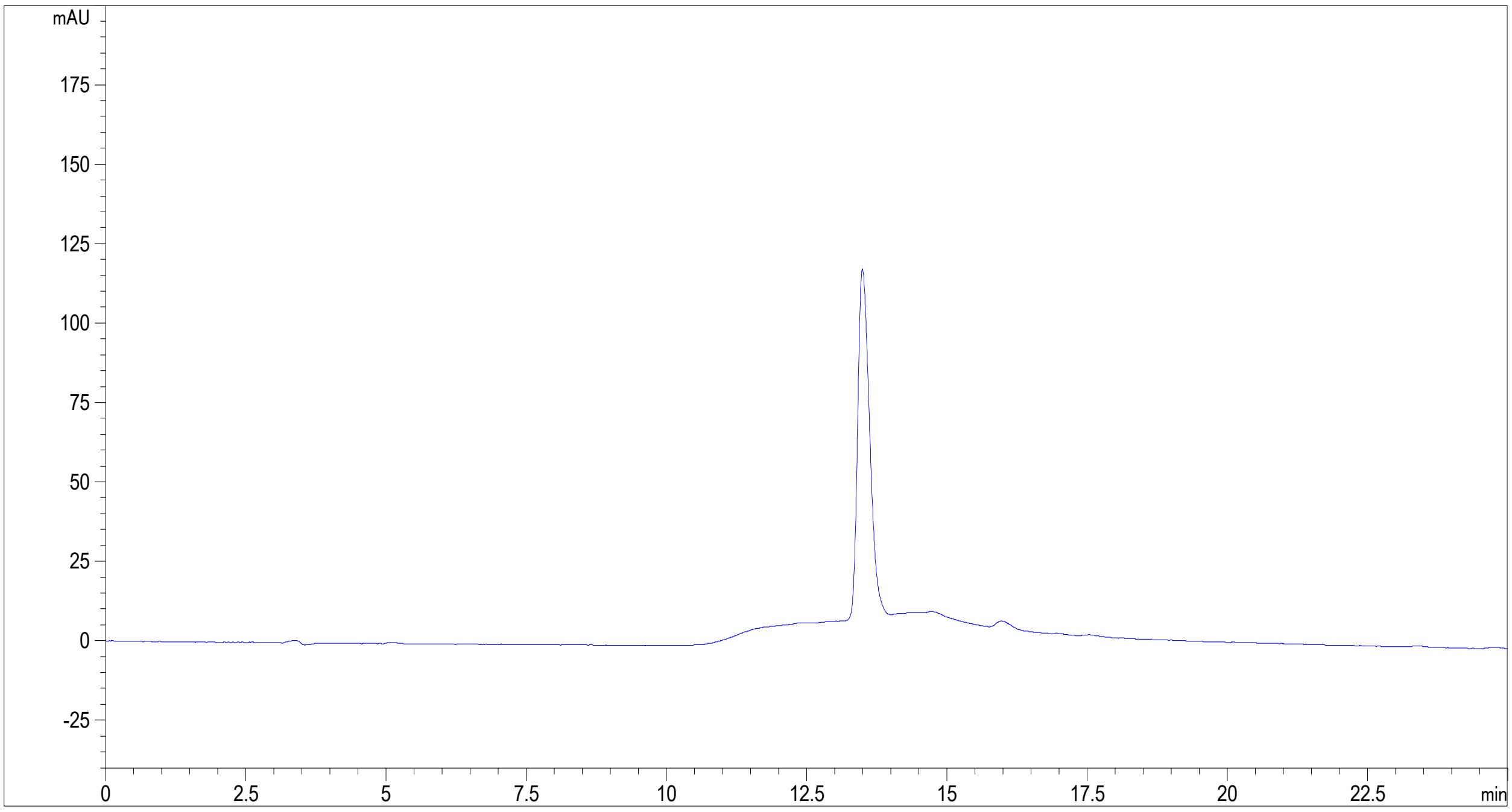

Compound **6** (254 nm) 0-25 min: 30-100% ACN/ 0.1%FA in water

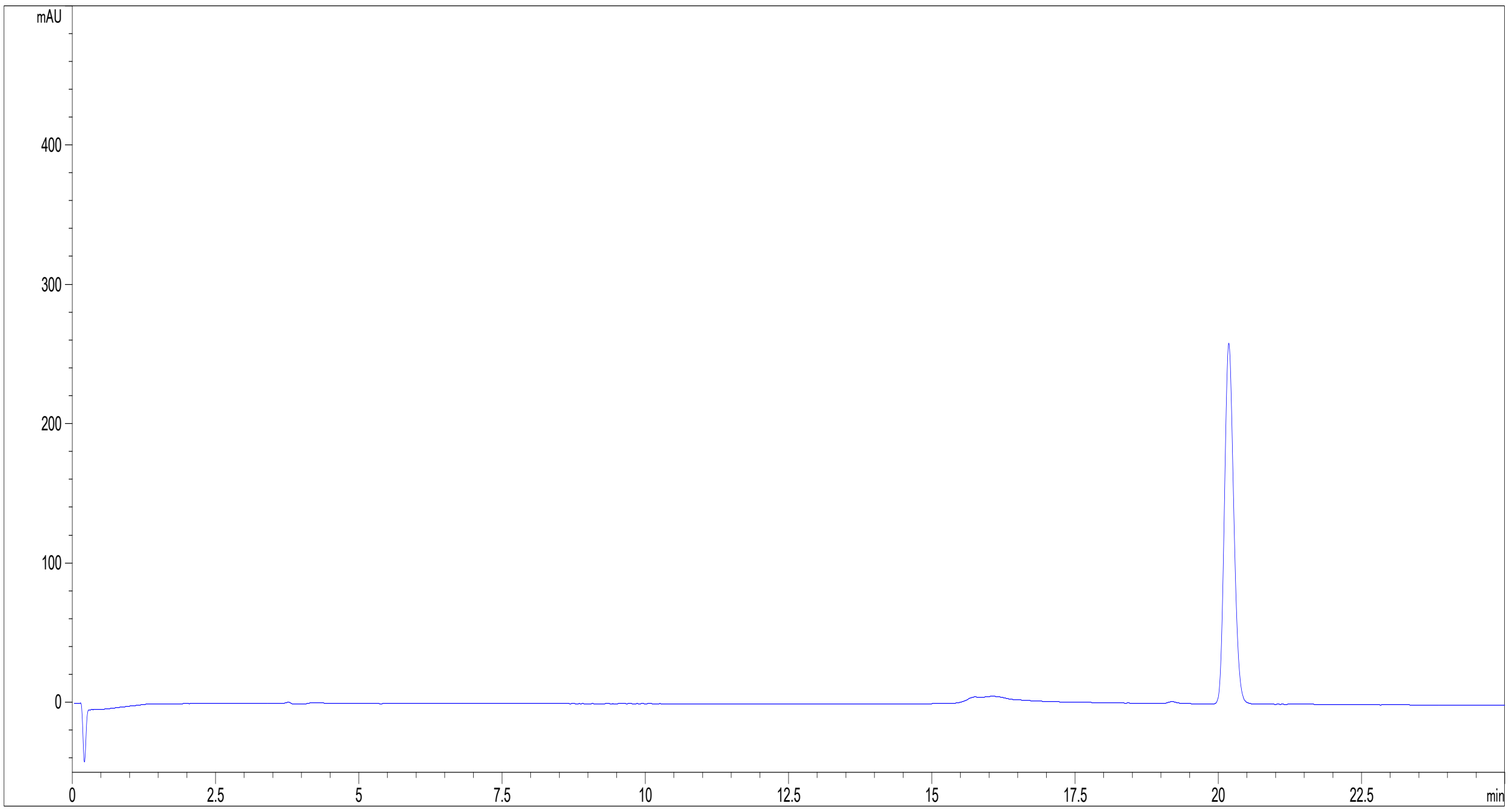

Compound **7** (254 nm) 0-25 min: 30-100% ACN/ 0.1%FA in water

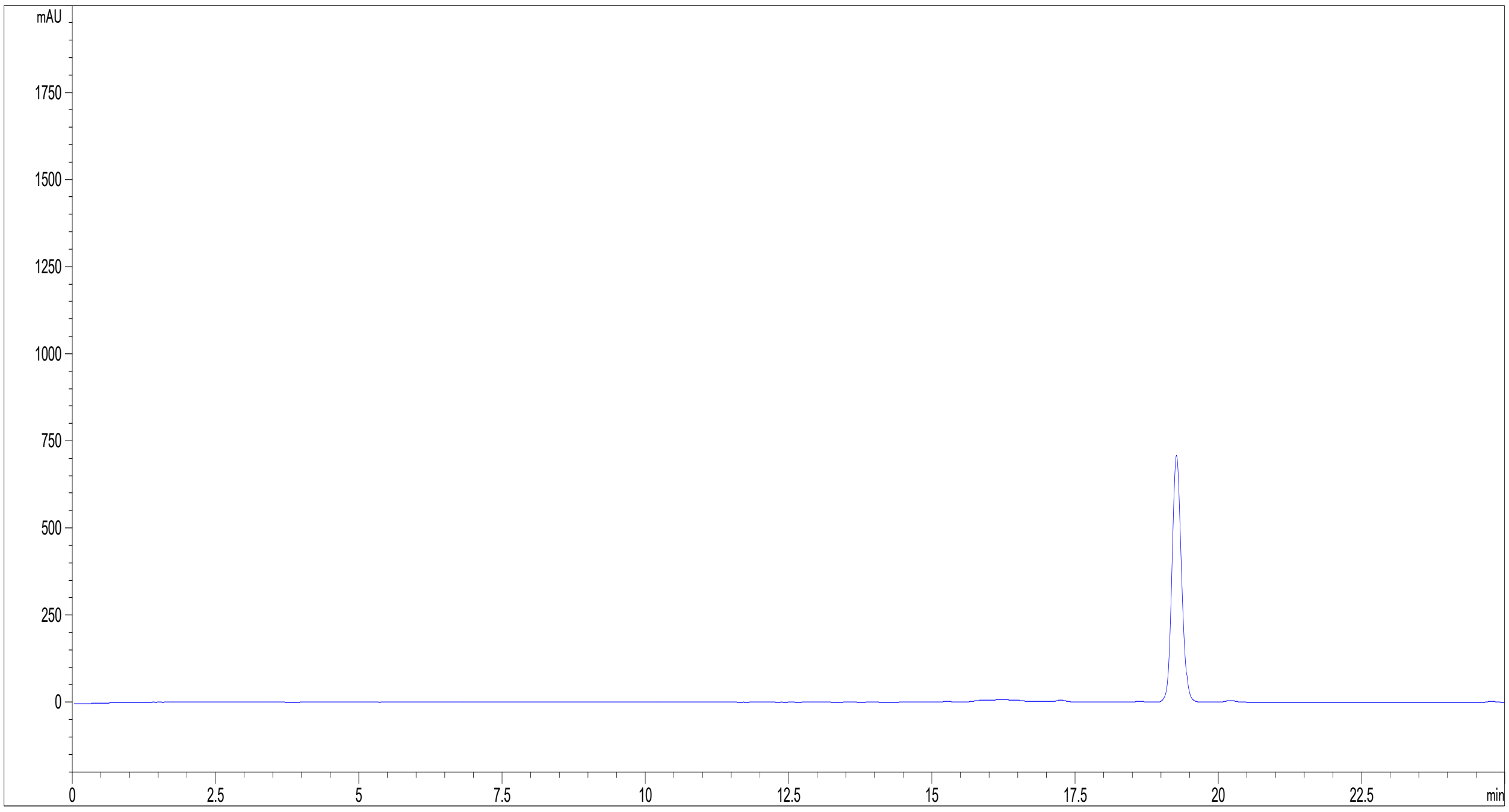

Compound **8** (254 nm) 0-25 min: 30-100% ACN/ 0.1%FA in water

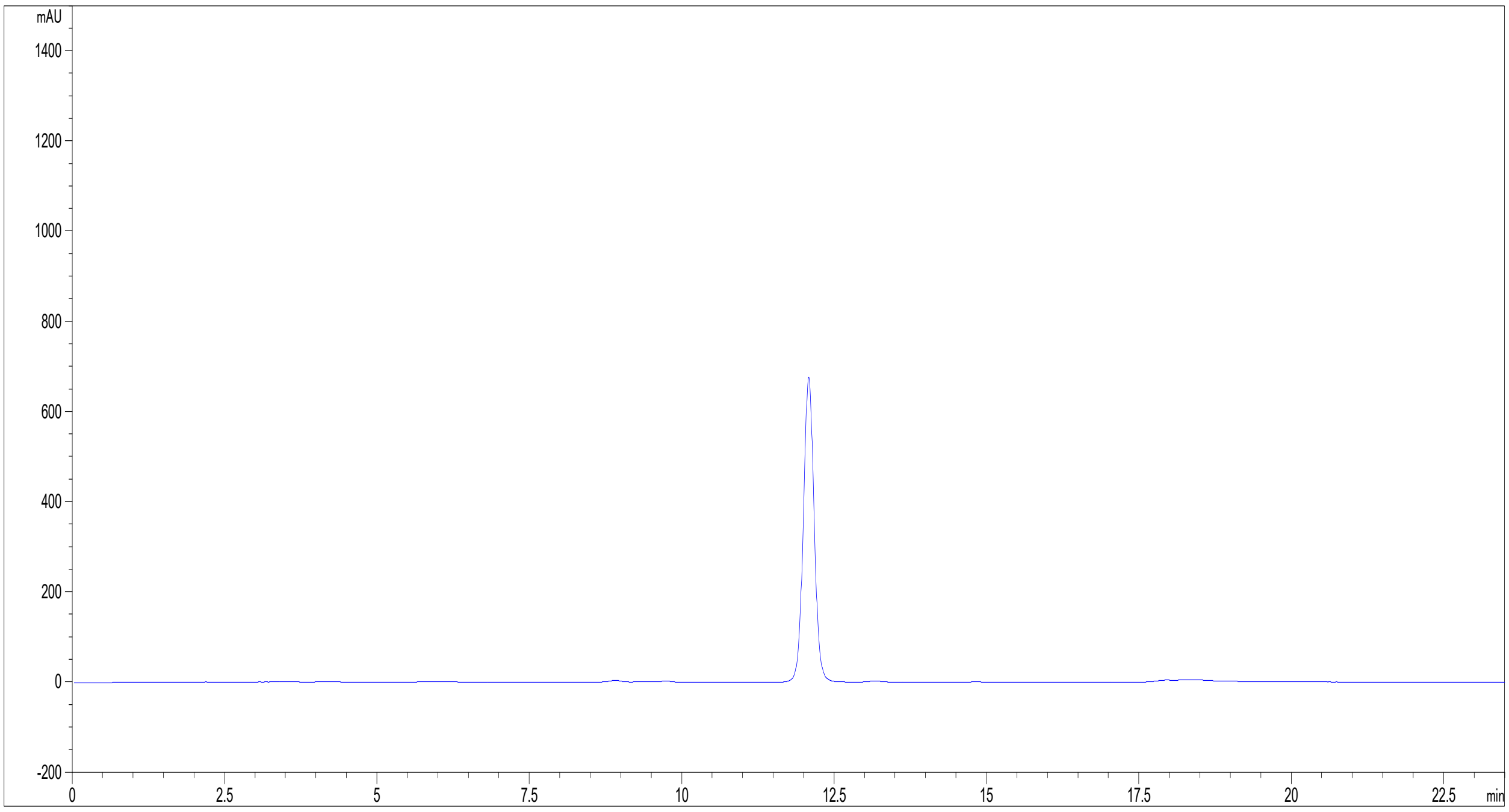

Compound **9** (254 nm) 0-25 min: 45-100% ACN/ 0.1%FA in water

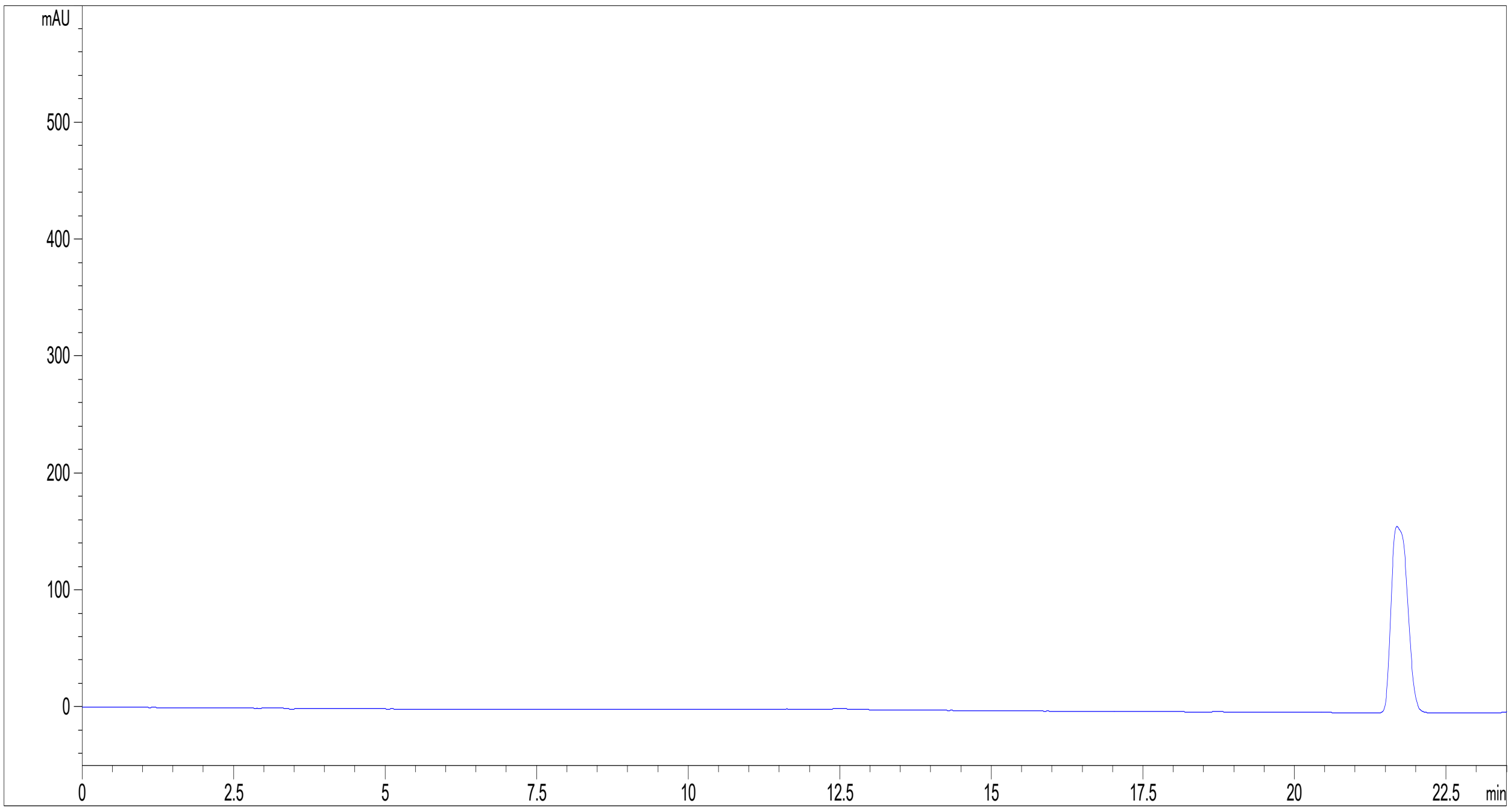

Compound **10** (254 nm) 0-25 min: 40-90% ACN/ 0.1%FA in water

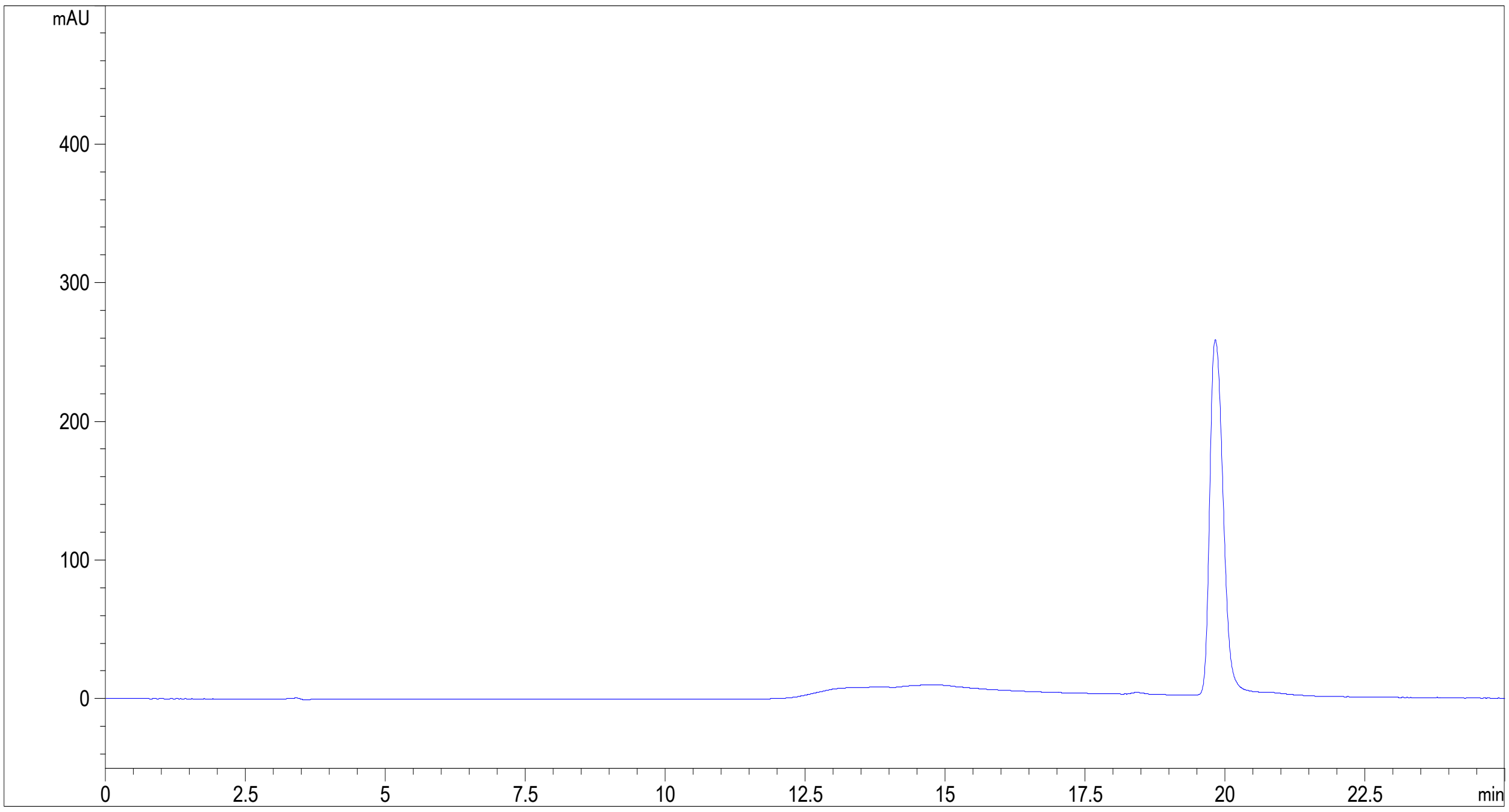

Compound **11** (254 nm) 0-25 min: 40-90% ACN/ 0.1%FA in water

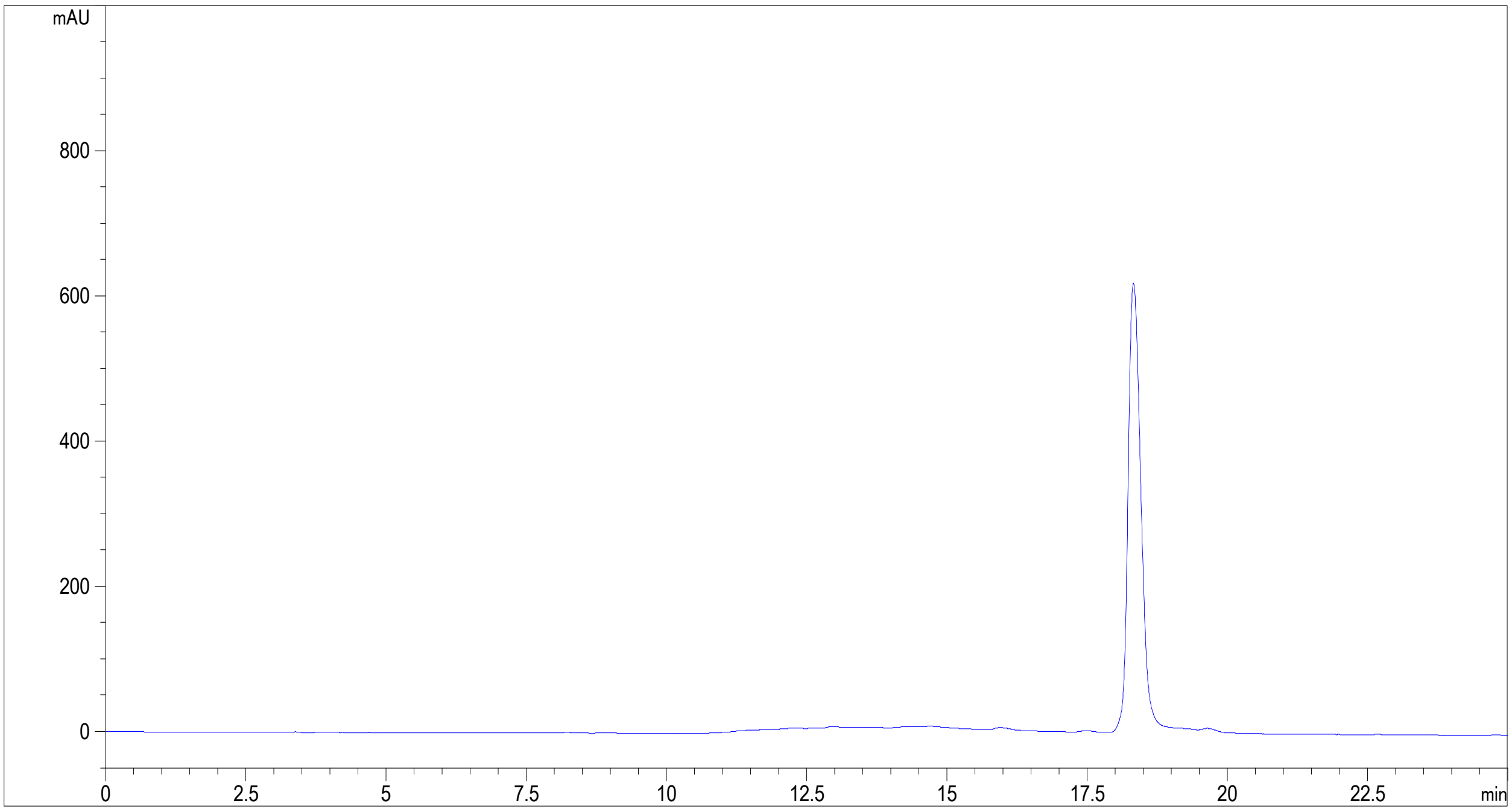

Compound **12** (280 nm) 0-25 min: 30-100% ACN/ 0.1%FA in water

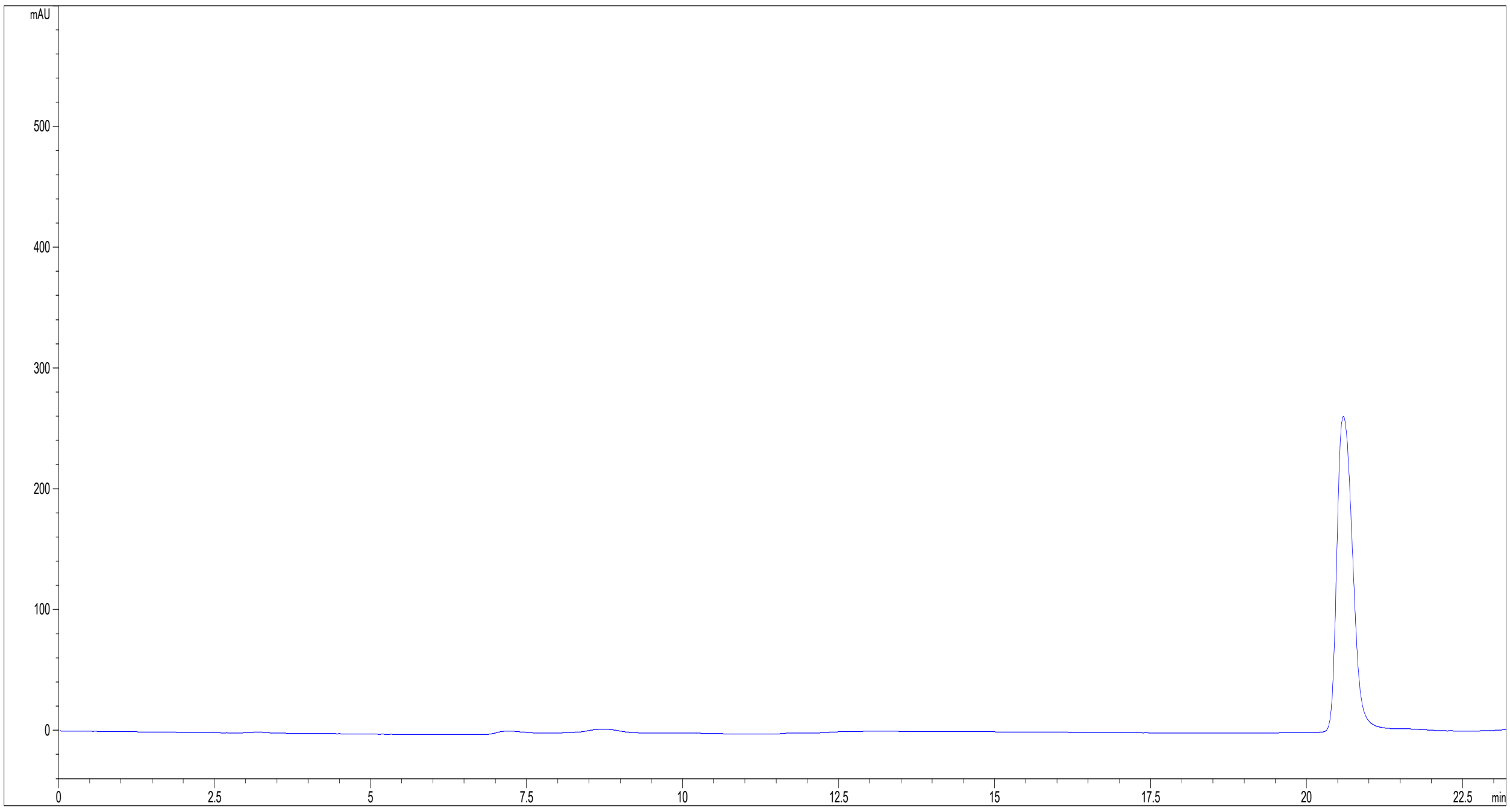

Compound **13** (254 nm) 0-25 min: 40-75% ACN/ 0.1%FA in water

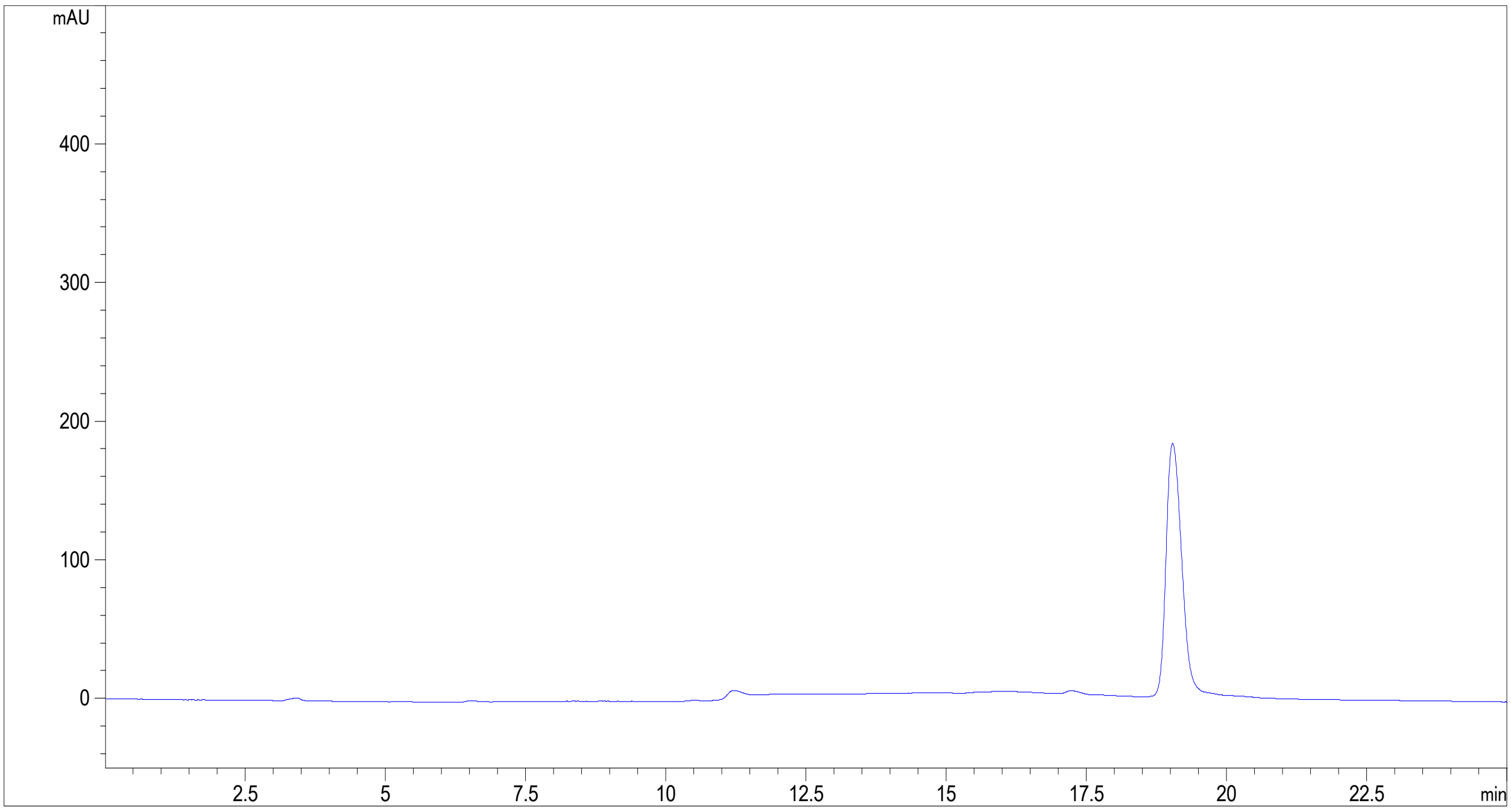

Compound **14** (254 nm) 0-25 min: 60% ACN/ 0.1%FA in water

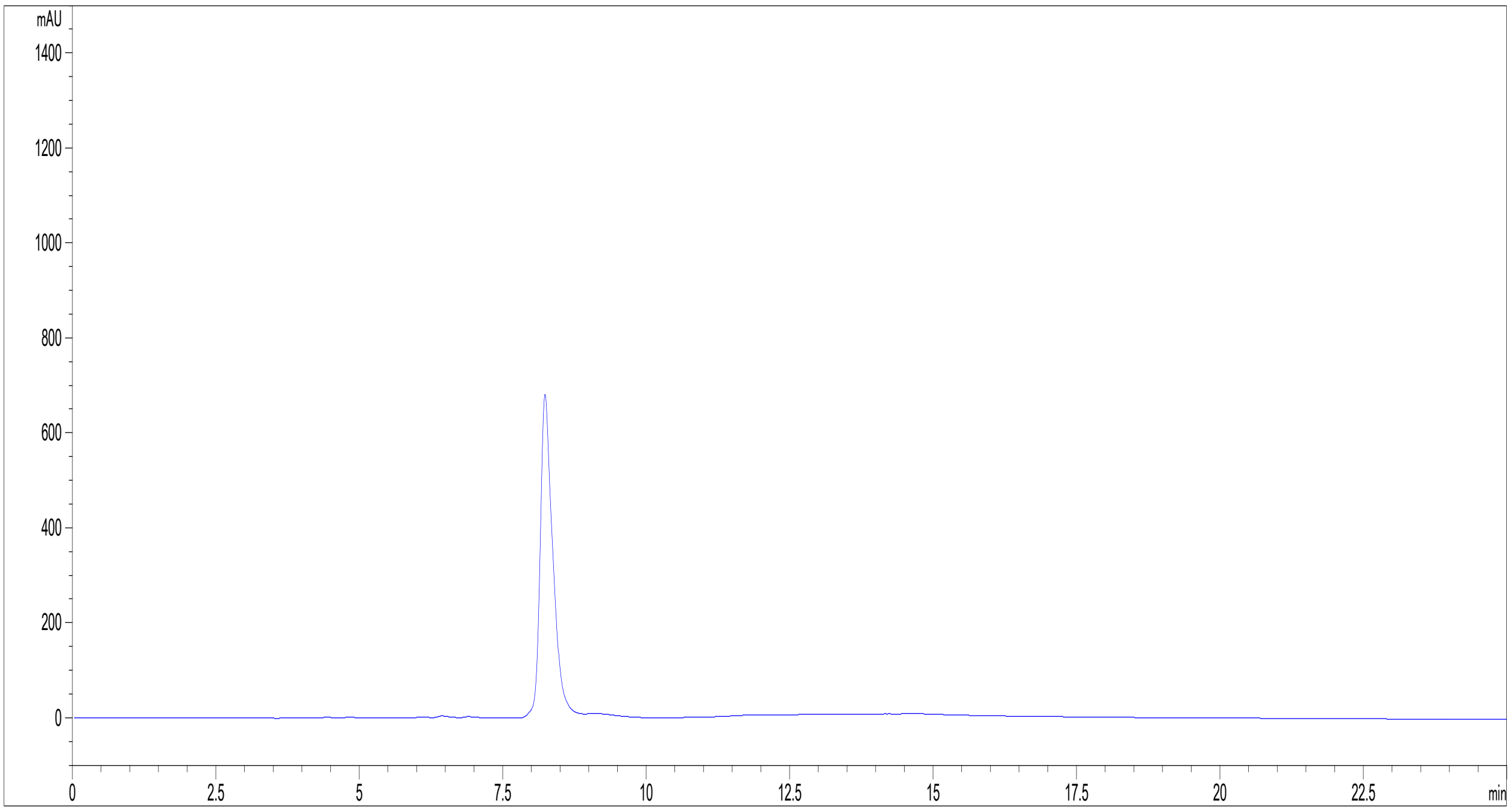

Figure S91. HPLC chromatograms of the isolated compounds 1–14.

Table S1. Cytotoxicity of isolated compounds in RAW 264.7 cells and their selectivity indices for NO inhibitory activity

Compounds	Cytotoxicity (CC_{50}, μM)	Selective index (SI)
1	> 300	>3.83
2	290.13 ± 2.56	5.25
3	294.83 ± 1.67	6.89
4	> 300	> 30.61
5	> 300	> 25.21
6	293.27 ± 2.02	17.05
7	265.07 ± 4.37	3.84
8	> 300	> 5.07
9	> 300	N.D
10	71.68 ± 2.81	N.D
11	38.12 ± 0.19	3.74
12	73.86 ± 0.58	N.D
13	74.33 ± 0.79	2.51
14	40.24 ± 0.42	1.76
Data are expressed as mean ± SE (n=3). N.D: Not be determined.		

Table S2. Similarity factor for experimental ECD spectrum of 1 and calculated curve of 1a

This algorithm intends a finetuning of a preoptimized UV shift only
Best Delta (ESI = 0.9789) was found for sigma =0.3 eV at 14 nm shift
Best Similarity factor (S = 0.9789) was found for sigma =0.3 eV at 14 nm shift
Similarity factor = 0.9789
Similarity factor Enantiomer = 0.0000
Delta value: 0.9789
Scaling factor for calculated ECD spectrum: 1.8251

Table S3. Similarity factor for experimental ECD spectrum of 2 and calculated curve of 2b

This algorithm intends a finetuning of a preoptimized UV shift only
Best Delta (ESI = 0.9091) was found for sigma =0.3 eV at 12 nm shift
Best Similarity factor (S = 0.9091) was found for sigma =0.3 eV at 12 nm shift
Similarity factor = 0.9091
Similarity factor Enantiomer = 0.0000
Delta value: 0.9091
Scaling factor for calculated ECD spectrum: 1.8071

Table S4. Gibbs Free Energy and Boltzmann Population of conformers of isomer 1a.

Conformer's name	Energy (a.u.)	Energy (kcal/mol)	Relative Energy (kcal/mol)	Boltzmann Factor	Equilibrium Mole Fraction	Number of Imaginary Frequencies
1a-1	-1304.06	-818307	0	1	0.620059	0
1a-2	-1304.05	-818307	0.425451	0.487299	0.302154	0
1a-3	-1304.05	-818306	1.394326	0.094802	0.058783	0
1a-4	-1304.05	-818305	2.062623	0.030648	0.019004	0

Table S5. Gibbs Free Energy and Boltzmann Population of conformers of isomer 1b

Conformer's name	Energy (a.u.)	Energy (kcal/mol)	Relative Energy (kcal/mol)	Boltzmann Factor	Equilibrium Mole Fraction	Number of Imaginary Frequencies
1b-1	-1304.07	-818317	0	1	0.454969	0
1b-2	-1304.07	-818317	0.057731	0.90706	0.412684	0
1b-3	-1304.07	-818316	1.250626	0.120856	0.054986	0
1b-4	-1304.07	-818316	1.048568	0.170036	0.077361	0

Table S6. Gibbs Free Energy and Boltzmann Population of conformers of isomer 2a

Conformer's name	Energy (a.u.)	Energy (kcal/mol)	Relative Energy (kcal/mol)	Boltzmann Factor	Equilibrium Mole Fraction	Number of Imaginary Frequencies
2a-1	-811.416	-509171	0.458709	0.460671	0.078894	0
2a-10	-811.417	-509172	0.245356	0.660622	0.113137	0
2a-11	-811.414	-509170	1.862448	0.042983	0.007361	0
2a-12	-811.414	-509170	1.972262	0.035704	0.006115	0
2a-13	-811.417	-509172	0.210843	0.700292	0.119931	0
2a-14	-811.413	-509169	2.407126	0.017124	0.002933	0
2a-15	-811.413	-509169	2.378888	0.017961	0.003076	0

2a-16	-811.413	-509169	2.496232	0.014731	0.002523	0
2a-17	-811.416	-509171	0.805722	0.256298	0.043893	0
2a-18	-811.413	-509169	2.533255	0.013837	0.00237	0
2a-19	-811.416	-509171	0.450552	0.467064	0.079989	0
2a-2	-811.416	-509171	0.874748	0.228083	0.039061	0
2a-20	-811.414	-509170	1.956574	0.036663	0.006279	0
2a-3	-811.415	-509171	1.106299	0.154233	0.026414	0
2a-4	-811.416	-509171	0.624372	0.348196	0.059632	0
2a-5	-811.416	-509171	0.825175	0.248011	0.042474	0
2a-6	-811.415	-509171	0.989582	0.187856	0.032172	0
2a-7	-811.417	-509172	0	1	0.171259	0
2a-8	-811.416	-509171	0.825802	0.247748	0.042429	0
2a-9	-811.417	-509172	0.210216	0.701035	0.120058	0

Table S7. Gibbs Free Energy and Boltzmann Population of conformers of isomer 2b

Conformer's name	Energy (a.u.)	Energy (kcal/mol)	Relative Energy (kcal/mol)	Boltzmann Factor	Equilibrium Mole Fraction	Number of Imaginary Frequencies
2b-1	-811.416	-509171	0.458082	0.461159	0.082474	0
2b-10	-811.414	-509170	1.862448	0.042983	0.007687	0
2b-11	-811.414	-509170	1.972262	0.035704	0.006385	0
2b-12	-811.417	-509172	0.210843	0.700292	0.12524	0
2b-13	-811.413	-509169	2.407126	0.017124	0.003062	0
2b-14	-811.413	-509169	2.378888	0.017961	0.003212	0
2b-15	-811.413	-509169	2.496232	0.014731	0.002634	0
2b-16	-811.416	-509171	0.805722	0.256298	0.045836	0
2b-17	-811.413	-509169	2.533255	0.013837	0.002475	0
2b-18	-811.416	-509171	0.450552	0.467064	0.08353	0
2b-19	-811.414	-509170	1.956574	0.036663	0.006557	0
2b-2	-811.416	-509171	0.874748	0.228083	0.04079	0
2b-3	-811.415	-509171	1.106299	0.154233	0.027583	0
2b-4	-811.416	-509171	0.624372	0.348196	0.062271	0
2b-5	-811.416	-509171	0.825802	0.247748	0.044307	0
2b-6	-811.41	-509171	0.989582	0.187856	0.033596	0

	5					
2b-7	-811.417	-509172	0	1	0.17884	0
2b-8	-811.417	-509172	0.210216	0.701035	0.125373	0
2b-9	-811.417	-509172	0.245356	0.660622	0.118146	0

Table S8. The Cartesian coordinates of the optimized conformers

1a_1

C	-0.17606400	-0.83191800	-0.45861200
C	0.06024300	0.53242000	-1.17622000
C	-1.19429300	1.41078600	-0.97011600
C	-2.48654200	0.72137100	-1.40490100
C	-2.66173600	-0.69553600	-0.86828800
C	-1.39621800	-1.50299400	-1.03193500
H	-0.40357100	-0.59142100	0.58532100
C	1.08358200	-1.71263400	-0.44501300
C	0.26235800	0.35411800	-2.68766100
C	1.26962000	1.26197200	-0.54964800
H	-1.06079400	2.32174100	-1.55463600
O	-1.26925300	1.74855400	0.42673100
H	-3.34825900	1.32096700	-1.10922800
H	-2.50028100	0.67007500	-2.49392300
O	-2.99868600	-0.57130800	0.53281500
H	-3.48545300	-1.16959800	-1.40031100
C	-1.40297600	-2.65421000	-1.69021000
H	1.50708500	2.16146900	-1.12225000
H	1.01481400	1.56465400	0.46496700
C	2.51809200	0.39868600	-0.44445300
H	0.89085100	-2.63456500	0.10301800
H	1.37115200	-1.98533100	-1.46454200
C	2.17094100	-0.92822000	0.18843000
O	3.44774300	0.99182800	0.48885100
O	3.11952300	0.31996500	-1.69091100
C	2.82686800	-1.07164500	1.33587800
C	3.64942700	0.14673500	1.52713200
O	4.38122000	0.42518900	2.44011200
C	2.79788300	-2.15752600	2.35042900
H	3.79476800	-2.58194100	2.48413300
H	2.47856100	-1.76695000	3.31852800
H	2.11819200	-2.95538600	2.05553700
C	4.22518200	-0.56740200	-1.80198800
H	4.60310100	-0.45238300	-2.81466300
H	5.01473100	-0.30910700	-1.09306000
H	3.92127700	-1.60558500	-1.64534500
H	0.38141800	1.32849200	-3.16581200
H	1.16190600	-0.21953200	-2.89542600
H	-0.57692200	-0.15775800	-3.15557400
H	-2.31965100	-3.05667900	-2.10540800
H	-0.50325600	-3.23401800	-1.85168200
C	-3.69360600	-1.50196100	1.20541400
C	-1.76289700	2.90843500	0.87819300
O	-1.83727800	3.06076500	2.07263900
C	-2.16420200	3.96687900	-0.11319400
H	-2.61614600	4.79045900	0.43129600
H	-1.28526900	4.33337600	-0.64684100
H	-2.87192100	3.58950700	-0.85079200
O	-3.83999300	-1.33891100	2.39170400
C	-4.27010300	-2.67126400	0.45763400

H	-4.80412500	-3.29970200	1.16397900
H	-4.96066900	-2.33525600	-0.31722200
H	-3.48109200	-3.25074100	-0.02081500

1a_2

C	0.01619200	-1.07057000	-0.08825000
C	0.10143500	0.00679800	-1.20986400
C	-1.23424900	0.78095600	-1.23970000
C	-2.44684600	-0.13081900	-1.40168000
C	-2.47694000	-1.31248500	-0.43373800
C	-1.13784200	-2.00237600	-0.34996700
H	-0.21935000	-0.53102300	0.83548000
C	1.36076200	-1.77753900	0.14607800
C	0.30523100	-0.62697200	-2.59335400
C	1.23273700	1.01252900	-0.90084300
H	-1.19451000	1.48004700	-2.07560200
O	-1.34761000	1.51563700	-0.00722400
H	-3.36052000	0.45631700	-1.29826600
H	-2.45238300	-0.52828900	-2.41713300
O	-2.78677800	-0.86519500	0.90649600
H	-3.22777200	-2.02998700	-0.75950200
C	-1.04716000	-3.31324200	-0.52553900
H	1.36845100	1.69756200	-1.74094100
H	0.95606400	1.59987200	-0.02624800
C	2.56619900	0.35370200	-0.57962300
H	1.27321500	-2.48946600	0.96626000
H	1.66095500	-2.33338200	-0.74704600
C	2.36731300	-0.73208400	0.45155700
O	3.43571500	1.29979000	0.08040600
O	3.16281500	-0.05659600	-1.76193700
C	3.04815600	-0.44179200	1.55601100
C	3.73819900	0.84891400	1.32067200
O	4.44670400	1.47089700	2.06749400
C	3.14725800	-1.14993300	2.85912300
H	4.18556200	-1.40968600	3.07416800
H	2.79822400	-0.50789000	3.67009200
H	2.55444300	-2.06321600	2.85868500
C	4.35406800	-0.82263200	-1.63405700
H	4.71325900	-0.99176500	-2.64596400
H	5.11594200	-0.28122100	-1.06913300
H	4.16032400	-1.78516400	-1.15370500
H	0.28629100	0.14547700	-3.36503200
H	1.27060400	-1.12231100	-2.65562900
H	-0.46423900	-1.36242100	-2.82342100
H	-1.92962000	-3.91310600	-0.71539900
H	-0.10118300	-3.83838400	-0.48897300
C	-4.04306700	-0.72866400	1.35272000
C	-1.95664000	2.70560300	0.08685100
O	-2.04479800	3.20626200	1.18050000
C	-2.46726100	3.36583800	-1.16502100
H	-3.13787700	2.71527100	-1.72594500
H	-2.99711300	4.27014000	-0.88141900

H	-1.63338000	3.63194800	-1.81709700
O	-4.20017000	-0.27246000	2.45842300
C	-5.18356300	-1.18113200	0.48089300
H	-5.15746900	-0.71615000	-0.50417100
H	-5.14449400	-2.26327500	0.34315200
H	-6.11433900	-0.92115800	0.97591800

1a_3

C	0.21971600	-0.79847600	0.59080700
C	-0.12301200	0.65931800	1.02647000
C	1.07017400	1.57027600	0.66060900
C	2.40006300	1.07195800	1.22283000
C	2.68403300	-0.40425900	0.96568400
C	1.47729200	-1.25449800	1.28293800
H	0.44389900	-0.74930700	-0.48009500
C	-0.97209800	-1.75507800	0.75260500
C	-0.34259100	0.76355300	2.54245600
C	-1.36719200	1.16131600	0.26097300
H	0.86285400	2.56301600	1.06120700
O	1.14386800	1.64321500	-0.77430300
H	3.22146000	1.66654300	0.82139700
H	2.39774600	1.22882400	2.30192600
O	3.02978700	-0.52351900	-0.43365500
H	3.53279100	-0.70669000	1.57761700
C	1.55781300	-2.25214500	2.15301500
H	-1.64839100	2.15809200	0.60712600
H	-1.12118800	1.24698600	-0.79652200
C	-2.55449700	0.20482800	0.34297500
H	-0.70697300	-2.74761700	0.38904500
H	-1.25006000	-1.84901200	1.80585200
C	-2.11241300	-1.19175100	-0.00874000
O	-3.50770500	0.54535500	-0.68352900
O	-3.21588900	0.14623800	1.56463000
C	-2.78304300	-1.61916600	-1.07365600
C	-3.68857700	-0.52450700	-1.49490800
O	-4.46298600	-0.49887700	-2.41442800
C	-2.70940700	-2.89891700	-1.82675200
H	-1.96437000	-3.56724700	-1.39802700
H	-3.67661400	-3.40515400	-1.81465200
H	-2.45223300	-2.71371200	-2.87136000
C	-3.73349300	1.37359200	2.06475000
H	-4.39279300	1.10852300	2.88775300
H	-2.93807900	2.01983600	2.44223300
H	-4.30598800	1.90102300	1.29989600
H	-0.51094200	1.80432100	2.82711600
H	-1.21389600	0.18981400	2.85090100
H	0.51170700	0.39228500	3.10609300
H	2.49608900	-2.49872600	2.63625800
H	0.70011600	-2.85347400	2.42576800
C	3.78817700	-1.51858100	-0.92010600
C	1.55761200	2.73236300	-1.43476400
O	1.63841700	2.66409300	-2.63640400

C	1.86621500	3.98416400	-0.65876200
H	2.25574000	4.72583000	-1.34955000
H	0.95802200	4.37283200	-0.19436400
H	2.59706600	3.80581500	0.12972300
O	3.94164200	-1.56860600	-2.11558900
C	4.42020900	-2.49438100	0.03231600
H	5.00240000	-3.20674100	-0.54442800
H	5.07633800	-1.98257300	0.73767200
H	3.65833700	-3.02432900	0.60318400

1a_4

C	-0.01481300	-1.06366700	-0.39412100
C	0.14675100	0.31534100	-1.10087200
C	-1.15133400	1.12485600	-0.89086700
C	-2.40092300	0.37790100	-1.34745000
C	-2.50843000	-1.05021000	-0.81496300
C	-1.20538700	-1.79739000	-0.95352300
H	-0.24640200	-0.84258600	0.65343200
C	1.28747500	-1.87971700	-0.39627800
C	0.35926800	0.15670500	-2.61318900
C	1.31317000	1.10352500	-0.46565300
H	-1.05953200	2.05186200	-1.45762000
O	-1.26079500	1.43041000	0.51101300
H	-3.28824200	0.94742200	-1.06659600
H	-2.40062000	0.32982400	-2.43701500
O	-2.82525200	-1.04636900	0.59629300
H	-3.28482500	-1.58389900	-1.36009500
C	-1.17149400	-2.98107900	-1.54949900
H	1.46931300	2.04444000	-0.99745400
H	1.05171600	1.35558200	0.56134000
C	2.61154700	0.30366200	-0.38831300
H	1.14758200	-2.80934700	0.15470500
H	1.57726400	-2.13820100	-1.41844200
C	2.34520800	-1.04666800	0.22423200
O	3.50787900	0.94550400	0.54068100
O	3.28319100	0.10105700	-1.58876800
C	3.05711600	-1.18102900	1.33863000
C	3.81613700	0.07656500	1.53346100
O	4.57517200	0.36947100	2.41888700
C	3.13811900	-2.29614300	2.31866800
H	2.48720800	-3.12101700	2.03320900
H	4.16151000	-2.67002500	2.38763800
H	2.84972800	-1.95214600	3.31376800
C	3.64266100	1.26802600	-2.31925200
H	4.34134900	0.93984200	-3.08502300
H	2.77437400	1.72111700	-2.80265100
H	4.13170200	2.00137200	-1.67585400
H	0.38318100	1.13616900	-3.09569800
H	1.30321000	-0.34118500	-2.82372300
H	-0.43167200	-0.43053900	-3.07726200
H	-2.07621000	-3.43979100	-1.93105500
H	-0.25177900	-3.53652100	-1.68166000

C	-4.08281900	-0.99541100	1.05640700
C	-1.79519200	2.56718500	0.97811300
O	-1.88911400	2.69324400	2.17378100
C	-2.21831000	3.63078900	0.00171600
H	-2.69975400	4.43033400	0.55660400
H	-1.34642900	4.03330000	-0.51720200
H	-2.90846200	3.24579200	-0.74864300
O	-4.24183300	-0.91645400	2.24969700
C	-5.22474700	-1.07769800	0.07924600
H	-6.15230300	-0.94249000	0.62725900
H	-5.15360800	-0.32044100	-0.70096800
H	-5.23628500	-2.05702200	-0.40261700

1b_1

C	-0.12371000	0.33395200	-1.30820000
C	1.14901600	1.20518400	-1.28549400
C	2.40951700	0.43057800	-1.65901400
C	2.58918900	-0.87741000	-0.90039600
C	1.31071000	-1.68024200	-0.84399800
C	0.11434500	-0.89242000	-0.37565100
C	-0.37600500	-0.09751600	-2.75955100
C	-1.30213600	1.18573200	-0.78428900
O	1.27855800	1.74082000	0.04702400
H	1.02458100	2.04283000	-1.97385900
H	3.28582800	1.06132800	-1.51153400
H	2.36779100	0.19873700	-2.72369600
H	3.37982300	-1.46697800	-1.36122400
O	3.00766900	-0.53602100	0.44450000
C	1.29517400	-2.95184300	-1.21893900
H	0.37376700	-0.48073600	0.60596200
C	-1.15796800	-1.73142800	-0.17506800
H	-1.31091300	-0.64547000	-2.84548400
H	-0.45321000	0.78167100	-3.40266700
H	0.42184000	-0.73306800	-3.14073100
C	-2.55855200	0.38040300	-0.48687100
H	-1.00804900	1.66205600	0.15005000
H	-1.54715900	1.97168500	-1.50228400
H	-0.96355700	-2.55107500	0.51618400
C	-2.20843700	-0.82671500	0.35080600
H	-1.48235900	-2.16671000	-1.12479200
O	-3.21054900	0.10370800	-1.67842800
O	-3.44194400	1.14488900	0.36455700
C	-2.82018200	-0.75664700	1.52929200
C	3.75996500	-1.41540400	1.11640400
C	1.99577700	2.85193700	0.22496300
C	2.06535900	3.21866500	1.67712500
O	2.51869400	3.46312300	-0.67618500
H	1.06370700	3.24992700	2.10536800
H	2.63087300	2.45277500	2.21034400
H	2.55421600	4.18186900	1.79333500
C	4.05501800	-0.91696600	2.50069000
O	4.14706400	-2.46110200	0.65515200

H	4.55716300	0.04929400	2.44610000
H	3.12052600	-0.77461200	3.04451400
H	4.68283600	-1.63195000	3.02502500
H	0.39036700	-3.54662400	-1.21633600
H	2.20398100	-3.44494000	-1.54222300
C	-4.33251500	-0.76597600	-1.59548100
H	-4.75033900	-0.81513500	-2.59776600
H	-4.03737900	-1.76916500	-1.27738200
H	-5.08821100	-0.37525200	-0.91062100
C	-3.61410000	0.49465000	1.53908300
O	-4.30243000	0.94219500	2.41843200
C	-2.76840300	-1.65341300	2.71371700
H	-3.76412600	-2.03490800	2.94784900
H	-2.10549700	-2.49950700	2.53984900
H	-2.41538600	-1.10767400	3.59082500

1b_2

C	-0.12893400	-0.37989700	-1.20754000
C	1.22711800	0.29855500	-1.49002700
C	2.41179700	-0.65621300	-1.39036800
C	2.42989600	-1.49857100	-0.11664200
C	1.07714300	-2.09131500	0.18418900
C	-0.04436600	-1.08531500	0.17937100
C	-0.39137300	-1.38401100	-2.33905900
C	-1.22221300	0.71179400	-1.18482200
O	1.39205100	1.37194600	-0.53936900
H	1.20414200	0.73809200	-2.48814100
H	3.34226300	-0.09698400	-1.48120600
H	2.38038300	-1.33967600	-2.23983800
H	3.17767400	-2.28445300	-0.21258200
O	2.77639100	-0.68876300	1.03523700
C	0.94367600	-3.39353800	0.39439600
H	0.23008500	-0.30683200	0.89980500
C	-1.40145800	-1.64479500	0.63589000
H	-1.37089200	-1.84327000	-2.23410400
H	-0.37306100	-0.87291700	-3.30386300
H	0.35219900	-2.17942500	-2.35781000
C	-2.56419000	0.22495700	-0.65791800
H	-0.90256000	1.51976700	-0.52779400
H	-1.36351800	1.12560100	-2.18581600
H	-1.31123100	-2.08986400	1.62644200
C	-2.36674000	-0.51910200	0.64189400
H	-1.74332400	-2.42527300	-0.05004400
O	-3.20833500	-0.48968500	-1.65582500
O	-3.38539100	1.35462000	-0.28552800
C	-3.00267500	0.10776100	1.62705100
C	4.06700400	-0.41963500	1.24717800
C	2.14556200	2.41881500	-0.88800000
C	2.24379400	3.41442900	0.22888500
O	2.68773900	2.52481300	-1.96180400
H	2.98737900	3.06037300	0.94569400
H	2.56412500	4.37623700	-0.16285900

H	1.29272500	3.50979200	0.74969900
C	4.26023800	0.38416900	2.49908200
O	4.95791400	-0.80464900	0.52782800
H	5.21034800	0.91033600	2.45431800
H	3.44013400	1.08450000	2.64514700
H	4.27624200	-0.29915400	3.35055200
H	-0.01667300	-3.85349900	0.59047300
H	1.80428700	-4.05186500	0.37355700
C	-4.41610400	-1.14221900	-1.28496500
H	-4.80701200	-1.59156400	-2.19428700
H	-4.23566900	-1.92495700	-0.54365900
H	-5.14741500	-0.43208900	-0.89294800
C	-3.66025400	1.29844300	1.03859400
O	-4.32355800	2.13942300	1.58631400
C	-3.08139400	-0.18400700	3.08253400
H	-4.11905600	-0.33566200	3.38593700
H	-2.51211800	-1.07631300	3.33798600
H	-2.69310400	0.65562500	3.66218300

1b_3

C	-0.18792800	0.68202000	-1.07538700
C	1.02888000	1.57250200	-0.75109700
C	2.32361800	1.06736300	-1.38019200
C	2.60505500	-0.40825800	-1.13316700
C	1.38166700	-1.26454400	-1.36155800
C	0.14934600	-0.77744100	-0.64294000
C	-0.45634400	0.77833600	-2.58371500
C	-1.39863800	1.20797900	-0.27260200
O	1.16214300	1.60924300	0.68383000
H	0.83357800	2.58909700	-1.09681200
H	3.16305600	1.66413500	-1.02417600
H	2.26572200	1.22259400	-2.45805200
H	3.41880500	-0.74230200	-1.77435500
O	3.03641900	-0.53697700	0.24451000
C	1.43790100	-2.32088300	-2.16097700
H	0.40637000	-0.72441700	0.42087000
C	-1.06117100	-1.71762700	-0.75674100
H	-1.35940100	0.23569300	-2.85510300
H	-0.59202000	1.82176000	-2.87619100
H	0.36292800	0.36546700	-3.17027800
C	-2.60097700	0.26771200	-0.30225300
H	-1.11430900	1.30327300	0.77451300
H	-1.67810900	2.20450800	-0.62046700
H	-0.79957000	-2.71148600	-0.39423100
C	-2.16491000	-1.13273900	0.04136700
H	-1.37486700	-1.81518900	-1.79965300
O	-3.31122400	0.21139800	-1.49609700
O	-3.50941600	0.62769100	0.75913800
C	-2.79843500	-1.54325100	1.13558200
C	3.86502600	-1.54078100	0.55545300
C	1.80946200	2.63443900	1.24153100
C	1.90262900	2.46565000	2.72839500

O	2.26145800	3.56204500	0.61366500
H	0.91995400	2.24732500	3.14569400
H	2.55313200	1.61749200	2.94839100
H	2.31187100	3.36558500	3.17886900
C	4.15811100	-1.55542300	2.02694300
O	4.31162200	-2.31910100	-0.25130400
H	4.87040500	-2.34378200	2.25376600
H	4.55877300	-0.58977600	2.33564900
H	3.23287100	-1.72272900	2.57980700
H	0.57363500	-2.93984800	-2.36661300
H	2.36804700	-2.60466400	-2.63835100
C	-3.82494200	1.44414600	-1.98699100
H	-4.52221600	1.18455900	-2.77991600
H	-3.03316200	2.07114300	-2.40262700
H	-4.35561000	1.98977700	-1.20487600
C	-3.67095700	-0.43350600	1.58486100
O	-4.40569200	-0.39054400	2.53622800
C	-2.71144300	-2.81852100	1.89506200
H	-2.40531300	-2.63008800	2.92587700
H	-3.68595400	-3.30938600	1.93001400
H	-1.99679900	-3.50181800	1.43904400

1b_4

C	-0.17843100	0.04584100	-1.19957800
C	1.14159800	0.84255700	-1.22549700
C	2.36123900	-0.01956000	-1.53091000
C	2.45697000	-1.28607000	-0.68298300
C	1.13856300	-2.01286000	-0.60923400
C	-0.01897000	-1.13913400	-0.19954500
C	-0.45114900	-0.45125900	-2.62648600
C	-1.30527100	0.99059900	-0.72666100
O	1.30599600	1.46826400	0.06439600
H	1.06564800	1.63375700	-1.97262600
H	3.26778800	0.57440900	-1.41932600
H	2.31992100	-0.32313400	-2.57781200
H	3.22934300	-1.93700700	-1.09000500
O	2.81444300	-0.96740000	0.68540800
C	1.06066600	-3.29941100	-0.92014500
H	0.25333800	-0.68909800	0.76164200
C	-1.33646000	-1.89749000	0.02692800
H	-1.41183900	-0.95814300	-2.68727100
H	-0.47238300	0.39113000	-3.32106600
H	0.31113200	-1.15085100	-2.96594800
C	-2.61482500	0.26676300	-0.42297500
H	-0.99934000	1.46778600	0.20364600
H	-1.46337700	1.78631700	-1.45747800
H	-1.19543700	-2.68126900	0.77082600
C	-2.35196800	-0.91445000	0.47446700
H	-1.67008000	-2.37306600	-0.89934000
O	-3.33845200	-0.18604200	-1.52028400
O	-3.46108700	1.13012000	0.36425300
C	-3.02123700	-0.76888200	1.61376200

C	4.10076400	-0.73821100	0.96071900
C	1.99935400	2.60842700	0.13295600
C	2.12259500	3.08903600	1.54782000
O	2.48031700	3.15772600	-0.82896300
H	2.97878200	2.59009700	2.00668300
H	2.30060900	4.16140100	1.55695400
H	1.23488700	2.84137300	2.12623300
C	4.30851400	-0.46835000	2.42178900
O	4.97911600	-0.77374600	0.13205200
H	3.46252500	0.06941100	2.84516700
H	4.39579500	-1.42476400	2.94135300
H	5.22922000	0.09184700	2.56388600
H	0.12590700	-3.84537100	-0.89942800
H	1.94354700	-3.85583700	-1.21257800
C	-3.69934900	0.79141400	-2.48929600
H	-4.43466100	0.31731500	-3.13492900
H	-2.84039900	1.09339500	-3.09239400
H	-4.14620300	1.66839200	-2.01782100
C	-3.74547500	0.52155600	1.54033200
O	-4.46028100	1.03217200	2.36183100
C	-3.08631400	-1.62753300	2.82587300
H	-2.75869200	-1.07186700	3.70656700
H	-4.11244300	-1.95199000	3.00941200
H	-2.45887600	-2.51087900	2.71810800

2a_1

C	0.39432900	-0.77403100	0.45193000
C	1.84390100	-1.24991700	0.22479600
C	2.87612800	-0.16773700	0.58482500
C	2.57751700	1.17381100	-0.11002100
C	1.14338700	1.58212900	0.11659200
C	0.16043500	0.53622600	-0.34196300
C	0.17527500	-0.57518100	1.95839600
C	-0.60535100	-1.80765400	-0.07953600
O	2.09629700	-1.71260300	-1.10059900
H	2.02361400	-2.12022600	0.86118800
O	4.18312200	-0.60577400	0.25279600
H	2.86096000	0.00290500	1.66158400
H	3.25175600	1.93448300	0.29353600
O	2.78892300	1.05112500	-1.52186000
C	0.83996200	2.72188100	0.72267700
H	0.42625700	0.32029000	-1.38554700
C	-1.28824100	0.93409900	-0.35344200
H	0.30265600	-1.52565500	2.48187000
H	0.86588000	0.14730700	2.39087000
H	-0.83067200	-0.20884600	2.16051700
C	-2.05239400	-1.37078300	0.14449300
H	-0.42798600	-1.94716800	-1.14759600
H	-0.43020000	-2.77429400	0.40153100
C	-2.29670800	0.08552400	-0.16606200
H	-2.35006900	-1.56266200	1.18084000
H	-2.71452600	-1.99224300	-0.46397700

H	-1.51012400	1.97168900	-0.58729300
C	-3.73407900	0.55550700	-0.24569300
H	-3.70785900	1.64552800	-0.33620500
C	-4.44404800	0.00731500	-1.48826400
C	-4.51974900	0.21088200	1.02269100
H	-5.45240400	0.42080500	-1.56234500
H	-3.90033000	0.26877900	-2.39808500
H	-4.53679300	-1.08068600	-1.44771300
H	-5.51584800	0.65682900	0.98509500
H	-4.64509400	-0.86930400	1.13003000
H	-4.01226000	0.58371200	1.91509500
H	-0.18494900	3.00406100	0.93060800
H	1.61506100	3.41000400	1.04012500
H	2.09365700	-0.94350300	-1.68498900
H	4.08511900	-1.24654400	-0.46140500
H	3.72521800	0.87033400	-1.64893500

2a_10

C	0.47102900	-0.85280500	0.50621600
C	1.94619900	-1.19158000	0.19098200
C	2.88198300	-0.01097400	0.50389300
C	2.43113400	1.27284900	-0.20768400
C	0.97707900	1.55914600	0.09712200
C	0.06591500	0.41909000	-0.28089300
C	0.32010700	-0.65820000	2.02144900
C	-0.45368400	-1.98351700	0.04070600
O	2.15568600	-1.63201300	-1.14675400
H	2.24530500	-2.03538500	0.81802700
O	4.22593900	-0.32802000	0.20801700
H	2.85391000	0.18767000	1.57429600
H	3.05991500	2.10150500	0.12986400
O	2.63875900	1.05696900	-1.60967500
C	0.61192900	2.69218000	0.68293700
H	0.28521500	0.20638700	-1.33574600
C	-1.41067800	0.68753600	-0.20337700
H	-0.71328700	-0.43713400	2.28568400
H	0.61623000	-1.56978000	2.54608100
H	0.92347600	0.16490200	2.40194200
C	-1.92292000	-1.67823100	0.32754100
H	-0.31336900	-2.12609900	-1.03227700
H	-0.16636100	-2.92064100	0.52648500
C	-2.32148800	-0.25170500	0.03903800
H	-2.16441900	-1.89199200	1.37539600
H	-2.55282400	-2.35704400	-0.25405500
H	-1.72921000	1.70275400	-0.41310300
C	-3.81334400	0.01680900	0.05036800
H	-4.23313600	-0.60548500	0.84977200
C	-4.19111900	1.46558400	0.34733300
C	-4.45431800	-0.43835000	-1.26778300
H	-5.27435800	1.55630400	0.45010700
H	-3.72831600	1.81758800	1.27117800

H	-3.88403400	2.13260300	-0.46204800
H	-4.22259700	-1.48003200	-1.49636100
H	-5.54114300	-0.33991400	-1.22122500
H	-4.09152000	0.17853400	-2.09399400
H	-0.41938300	2.89875200	0.94187300
H	1.34416400	3.45189900	0.93271000
H	2.11914900	-0.85548300	-1.72059400
H	4.24236600	-0.68199600	-0.68668900
H	2.35422100	1.83959700	-2.08711900

2a_11

C	0.38831200	-0.80412200	0.47010400
C	1.81759300	-1.30854200	0.21684000
C	2.88316000	-0.23703600	0.53063800
C	2.58443600	1.16591300	-0.02241700
C	1.13638900	1.54982500	0.16330200
C	0.17938200	0.50176400	-0.33800300
C	0.19524700	-0.58372000	1.97685600
C	-0.64228500	-1.82379300	-0.03129200
O	1.93118700	-1.75698400	-1.12281500
H	2.00655700	-2.15476500	0.89247800
O	4.15202700	-0.67277800	0.06729200
H	2.95080900	-0.14684800	1.61575900
H	3.22539900	1.87782900	0.50682800
O	2.95441400	1.14750000	-1.40478600
C	0.80759900	2.69246000	0.75274500
H	0.50403800	0.27150600	-1.36058000
C	-1.26466600	0.90916200	-0.39827400
H	-0.77719800	-0.13556200	2.18108100
H	0.24792200	-1.53938300	2.50362000
H	0.94401600	0.08149100	2.40494500
C	-2.07460900	-1.35174800	0.20577200
H	-0.48564600	-1.98143000	-1.09948600
H	-0.47892700	-2.78613200	0.46286000
C	-2.29143300	0.09089900	-0.17770500
H	-2.35222400	-1.48464800	1.25697700
H	-2.76181100	-1.98952700	-0.35642400
H	-1.46821400	1.93714500	-0.68690900
C	-3.71907400	0.58184900	-0.29597400
H	-3.67288300	1.66555900	-0.43972900
C	-4.42819400	-0.01446600	-1.51663600
C	-4.52144300	0.31350500	0.98034800
H	-5.42878700	0.41153600	-1.62007300
H	-3.87247600	0.19304400	-2.43311900
H	-4.53978900	-1.09751100	-1.42405400
H	-4.66040700	-0.75819100	1.14244400
H	-4.01781600	0.72622500	1.85726800
H	-5.51181300	0.76828600	0.90980300
H	-0.22465900	2.98027900	0.91064700
H	1.56998300	3.37823100	1.10558000
H	2.86797700	-1.90870900	-1.27870100
H	4.30982400	-0.19714600	-0.75841300

H	3.02263700	2.05512300	-1.70815300

2a_12

C	0.39736600	-0.76721300	0.48804200
C	1.83798500	-1.26273800	0.26577600
C	2.89119000	-0.17241100	0.52748900
C	2.57735700	1.21056600	-0.08719000
C	1.13126900	1.58203200	0.12351000
C	0.17971000	0.51566800	-0.35068300
C	0.19194900	-0.52080000	1.98894900
C	-0.61643300	-1.80862600	-0.00178400
O	2.07380200	-1.73477000	-1.06124200
H	2.03475000	-2.08199600	0.96750100
O	4.15983200	-0.61588500	0.07554100
H	2.96437700	-0.02884900	1.60652500
H	3.22032400	1.94211200	0.41609400
O	2.84608100	1.23230000	-1.48194600
C	0.79080500	2.71700200	0.71975100
H	0.50315200	0.26372900	-1.36867400
C	-1.26844500	0.90825600	-0.41632500
H	-0.78025400	-0.06558200	2.17775700
H	0.23727100	-1.46719700	2.53304500
H	0.94087900	0.14915900	2.40904400
C	-2.05543200	-1.35122200	0.22382100
H	-0.46462300	-1.97239100	-1.07170400
H	-0.44537700	-2.76442600	0.50342200
C	-2.28676600	0.08345900	-0.18153900
H	-2.33229200	-1.47295100	1.27635400
H	-2.73272900	-2.00556800	-0.33093900
H	-1.48133000	1.93002100	-0.71952500
C	-3.71974400	0.55714000	-0.30438300
H	-3.68495600	1.63876000	-0.46541400
C	-4.42529100	-0.06591900	-1.51364800
C	-4.51628800	0.30074100	0.97810300
H	-5.43016800	0.34861200	-1.62175300
H	-3.87350100	0.13207000	-2.43457600
H	-4.52634500	-1.14834500	-1.40317400
H	-5.51113900	0.74482100	0.90310200
H	-4.64475700	-0.76963400	1.15695000
H	-4.01451400	0.73174800	1.84721700
H	-0.24434900	2.98041800	0.90100600
H	1.54531000	3.42038300	1.05358900
H	1.76192500	-2.63860900	-1.12971000
H	3.97569600	-1.25900700	-0.62192100
H	3.68953800	0.78396000	-1.60236700

2a_13

C	0.39502800	-0.77287600	0.44733800
C	1.84660100	-1.24175900	0.21405700
C	2.87267200	-0.16375500	0.58579300
C	2.59196400	1.16548200	-0.14139300
C	1.15331900	1.58173900	0.09564900

C	0.16132300	0.53706900	-0.34635300
C	0.17658700	-0.58401300	1.95438300
C	-0.60620700	-1.80439400	-0.08935500
O	2.11570900	-1.55392000	-1.15720800
H	2.03985300	-2.12903600	0.82641200
O	4.19148800	-0.61741000	0.36645500
H	2.80105200	0.02735500	1.65541100
H	3.26385300	1.92361700	0.26304500
O	2.90248600	1.07997900	-1.53018500
C	0.85784600	2.73217100	0.68623000
H	0.41963800	0.30328000	-1.38770900
C	-1.28679000	0.93701300	-0.34877900
H	-0.84241100	-0.26047700	2.16240200
H	0.34710800	-1.52704400	2.47927200
H	0.83777000	0.16893600	2.38068100
C	-2.05380500	-1.36991300	0.13840000
H	-0.44695300	-1.92806400	-1.16416300
H	-0.43217500	-2.77706300	0.38070800
C	-2.29606800	0.08948900	-0.16015700
H	-2.35106600	-1.57296500	1.17237200
H	-2.71457600	-1.98595500	-0.47677300
H	-1.50913300	1.97608800	-0.57497800
C	-3.73323400	0.56205000	-0.22734300
H	-3.70526200	1.65232000	-0.31372500
C	-4.45392900	0.02042000	-1.46659000
C	-4.51011100	0.21440100	1.04569800
H	-5.46156600	0.43710700	-1.53170000
H	-3.91619400	0.28368600	-2.37945000
H	-4.54976200	-1.06745200	-1.42940300
H	-5.50497600	0.66378300	1.01784200
H	-4.63851700	-0.86579600	1.14944200
H	-3.99443400	0.58172200	1.93568700
H	-0.16378700	3.02776700	0.89264400
H	1.63946500	3.41912200	0.99125100
H	1.71274500	-2.39157600	-1.39014400
H	4.24734900	-0.89083800	-0.55435300
H	2.55334500	0.24353700	-1.86273200

2a_14

C	0.43315900	-0.94420900	0.31209900
C	1.91734400	-1.26557800	0.07674600
C	2.84833000	-0.14228100	0.58104200
C	2.42403900	1.28442600	0.19596800
C	0.93641100	1.49204600	0.34753500
C	0.11937200	0.42757800	-0.33444200
C	0.15638100	-0.94428100	1.82211900
C	-0.46388400	-1.98373300	-0.37125000
O	2.13527100	-1.52299600	-1.29979100
H	2.16675900	-2.16894300	0.65119300
O	4.17450700	-0.38331400	0.13652700
H	2.86158600	-0.18969400	1.67084200
H	2.96171100	1.98039100	0.84734700

O	2.85209300	1.48832600	-1.15435700
C	0.46124100	2.50806700	1.05683200
H	0.50910300	0.36827000	-1.35852000
C	-1.35531300	0.69867200	-0.42742700
H	-0.86218800	-0.61657600	2.03010900
H	0.27928700	-1.95265900	2.22418000
H	0.81914100	-0.27720100	2.37174300
C	-1.94387800	-1.68810200	-0.14491200
H	-0.25174900	-1.97872000	-1.44144500
H	-0.22067400	-2.98362500	0.00027600
C	-2.30102600	-0.23596200	-0.35720800
H	-2.23747600	-1.98941300	0.86582700
H	-2.54774300	-2.30441500	-0.81804300
H	-1.63431100	1.73022200	-0.61083300
C	-3.78242100	0.06581000	-0.47167200
H	-4.17474400	-0.59577800	-1.25412600
C	-4.51186200	-0.29168500	0.83101100
C	-4.10887400	1.49987000	-0.87585100
H	-5.58169500	-0.09494000	0.73512400
H	-4.38992500	-1.34303100	1.09418100
H	-4.12768700	0.31263600	1.65747500
H	-5.18664400	1.61197900	-1.00828500
H	-3.79506600	2.21036600	-0.10637500
H	-3.62330400	1.77717500	-1.81332000
H	-0.60207400	2.66618100	1.19052600
H	1.12905400	3.21535100	1.53610500
H	3.08869000	-1.55863900	-1.42044200
H	4.31548200	0.21047500	-0.61199100
H	2.83538600	2.43062800	-1.33411700

2a_15

C	0.44178900	-0.91292400	0.33220500
C	1.93485900	-1.22329100	0.12107100
C	2.85282800	-0.07787200	0.58220800
C	2.41305400	1.33913300	0.14721700
C	0.92751500	1.52635700	0.32600100
C	0.11850100	0.44346000	-0.33766000
C	0.15470900	-0.89885000	1.83996500
C	-0.43584900	-1.97156900	-0.34621700
O	2.27059100	-1.47933500	-1.24316800
H	2.19274700	-2.10805400	0.71501800
O	4.17787800	-0.32050400	0.13979200
H	2.87104800	-0.08140000	1.67303300
H	2.95098100	2.05378400	0.78109200
O	2.73344700	1.58792600	-1.21432800
C	0.43990500	2.52701000	1.04747800
H	0.50851400	0.37041300	-1.36083400
C	-1.35916200	0.69701900	-0.43516200
H	-0.86428700	-0.56662600	2.03837300
H	0.27286600	-1.90345500	2.25300400
H	0.81677100	-0.22755200	2.38505300
C	-1.92076200	-1.69386200	-0.12945900

H	-0.22868300	-1.96656800	-1.41935500
H	-0.18328600	-2.96716100	0.03191400
C	-2.29403300	-0.24779500	-0.35657300
H	-2.21254200	-1.99031400	0.88303500
H	-2.51311400	-2.32424600	-0.79940100
H	-1.64906500	1.72371100	-0.62823300
C	-3.77884700	0.03462500	-0.47500700
H	-4.16385700	-0.64373900	-1.24658600
C	-4.50340200	-0.31020400	0.83384000
C	-4.12136700	1.45826800	-0.90195300
H	-4.36825800	-1.35534900	1.11478800
H	-4.12672700	0.31293300	1.64967200
H	-5.57564800	-0.12870700	0.73504100
H	-5.20020600	1.55572200	-1.03717600
H	-3.81685000	2.18437500	-0.14346800
H	-3.63777000	1.72629000	-1.84311000
H	-0.62479900	2.65457000	1.20278200
H	1.09785000	3.25177600	1.51358200
H	2.06399600	-2.39264200	-1.44773700
H	4.09041500	-0.87262900	-0.64858900
H	3.62615200	1.25314200	-1.34800700

2a_16

C	0.46200800	-0.88002600	0.52473700
C	1.91438200	-1.25149800	0.18634000
C	2.89331900	-0.08608000	0.44100600
C	2.43541900	1.28108900	-0.09178600
C	0.97162200	1.53110900	0.17966300
C	0.08606200	0.39840300	-0.26631300
C	0.33887600	-0.67414100	2.04085100
C	-0.49726000	-1.99255700	0.08293500
O	1.99044600	-1.69004800	-1.15931700
H	2.21926400	-2.07555700	0.84684800
O	4.16876500	-0.40507300	-0.09380800
H	3.01372900	0.01399300	1.52061200
H	3.03841500	2.05083700	0.39981600
O	2.72346600	1.29087600	-1.49356800
C	0.57453700	2.64066200	0.78999400
H	0.36884700	0.19543400	-1.30683700
C	-1.38989600	0.67717600	-0.24017900
H	-0.65759200	-0.31939700	2.30382300
H	0.51299800	-1.61888600	2.56114800
H	1.04536500	0.06018700	2.42562000
C	-1.95059700	-1.64868600	0.39522800
H	-0.38518900	-2.14273200	-0.99184500
H	-0.22045700	-2.93273600	0.56919700
C	-2.32185000	-0.23213300	0.03402300
H	-2.16671500	-1.80054000	1.45935300
H	-2.60825000	-2.34451500	-0.13310100
H	-1.68958500	1.68562900	-0.50512200
C	-3.80858700	0.06192300	0.00649800
H	-4.25144400	-0.51081100	0.83028600

C	-4.16529300	1.53032900	0.22170700
C	-4.43735400	-0.45038600	-1.29640500
H	-5.24814500	1.64475200	0.30314600
H	-3.70943500	1.92261800	1.13273700
H	-3.83483000	2.14861000	-0.61662600
H	-5.52302400	-0.33249000	-1.27255100
H	-4.05165900	0.11714700	-2.14726200
H	-4.21945100	-1.50601400	-1.46756400
H	-0.46884700	2.83260900	1.00914300
H	1.28993000	3.39490000	1.09871900
H	2.92636100	-1.75823000	-1.36941100
H	4.23532200	0.07828500	-0.92727000
H	2.69064100	2.19978200	-1.79910200

2a_17

C	0.43491900	-0.90528900	0.29783100
C	1.93710800	-1.19569500	0.09859500
C	2.82456000	-0.07408700	0.65319200
C	2.43856500	1.29923800	0.07035000
C	0.95413100	1.53768800	0.26771500
C	0.10108400	0.46031100	-0.35097900
C	0.12590800	-0.92429300	1.80086400
C	-0.42681000	-1.95534700	-0.41541600
O	2.30196700	-1.31068200	-1.28130000
H	2.19399900	-2.12723800	0.61423600
O	4.19291200	-0.36342600	0.46098000
H	2.68347500	-0.02283800	1.73168100
H	3.00629400	2.06570500	0.59950500
O	2.82180400	1.41466700	-1.29795800
C	0.50946500	2.57176200	0.96960700
H	0.43281900	0.38198600	-1.39487000
C	-1.37887000	0.71759700	-0.39683300
H	-0.92918600	-0.72486500	1.98352500
H	0.36389800	-1.90458600	2.22071500
H	0.68681900	-0.17113800	2.35203300
C	-1.91901900	-1.69380200	-0.21592500
H	-0.20836200	-1.92702300	-1.48654900
H	-0.17397900	-2.95756600	-0.05631100
C	-2.30394600	-0.23908800	-0.35468400
H	-2.23203700	-2.05807000	0.76736000
H	-2.49219800	-2.28260900	-0.93824000
H	-1.67947800	1.75063700	-0.52756000
C	-3.79299400	0.03817000	-0.42498400
H	-4.19428200	-0.61373500	-1.21075100
C	-4.48312200	-0.35656300	0.88856300
C	-4.15281900	1.47461900	-0.79104800
H	-5.55790000	-0.17516300	0.82311900
H	-4.33899900	-1.41063700	1.12849000
H	-4.08762700	0.23825800	1.71644300
H	-5.23480300	1.56951700	-0.90011500
H	-3.83749200	2.17400900	-0.01218600
H	-3.69052500	1.78095700	-1.73122200

H	-0.54679400	2.73538500	1.14694700
H	1.19741800	3.29135600	1.39941800
H	1.99730600	-2.14796800	-1.63421900
H	4.32097500	-0.51496500	-0.48047300
H	2.57823500	0.59477800	-1.74583800

2a_18

C	0.47153500	-0.84197000	0.54309900
C	1.93608500	-1.20237300	0.23285200
C	2.89800600	-0.01852400	0.43286300
C	2.42032700	1.32760700	-0.15737000
C	0.96236200	1.56388600	0.14502300
C	0.08578700	0.41390700	-0.27548000
C	0.33473900	-0.61410700	2.05469000
C	-0.46843100	-1.97377500	0.11006600
O	2.13372700	-1.65535000	-1.10699600
H	2.24984600	-1.99764000	0.91948700
O	4.17279300	-0.34408700	-0.09563800
H	3.02285200	0.13462100	1.50581300
H	3.02364400	2.11690100	0.30616600
O	2.59629200	1.36994500	-1.56640900
C	0.55576700	2.66198500	0.76831300
H	0.36953100	0.19123000	-1.31189500
C	-1.39337900	0.67674400	-0.25279400
H	0.49722200	-1.55223000	2.59066200
H	1.04439500	0.12047600	2.43281400
H	-0.66118300	-0.24784700	2.30338900
C	-1.92722500	-1.64655500	0.41521200
H	-0.36285200	-2.12682100	-0.96697100
H	-0.18233200	-2.90806800	0.60318400
C	-2.31482100	-0.23901500	0.03513500
H	-2.14064000	-1.78838200	1.48102000
H	-2.57399600	-2.35799800	-0.10525400
H	-1.70333200	1.67864300	-0.53014400
C	-3.80492600	0.03661700	0.00575800
H	-4.23987300	-0.52933500	0.83839300
C	-4.17808100	1.50377000	0.20048400
C	-4.42960300	-0.50148700	-1.28872600
H	-5.26207200	1.60699700	0.28139800
H	-3.72591800	1.91408100	1.10537200
H	-3.85523600	2.11367400	-0.64688000
H	-4.19980200	-1.55682700	-1.44558700
H	-5.51651700	-0.39597300	-1.26446400
H	-4.05203800	0.05855900	-2.14812400
H	-0.48629700	2.82743100	1.01480200
H	1.26079700	3.43256100	1.05917800
H	1.90456200	-2.58477900	-1.15743600
H	4.00748200	-1.00064800	-0.78521400
H	3.47041000	1.00704400	-1.74211700

2a_19

C	0.46842300	-0.83881600	0.51966200
C	1.94318900	-1.17559500	0.21399000

C	2.88070600	0.00365500	0.50233000
C	2.43550300	1.27942300	-0.23862600
C	0.97866800	1.56502100	0.06998200
C	0.06735500	0.41984300	-0.29000400
C	0.31659200	-0.62842200	2.03202500
C	-0.45718600	-1.97587000	0.06692600
O	2.16537800	-1.49853300	-1.16322700
H	2.25328000	-2.02387100	0.83361200
O	4.22300600	-0.32992900	0.21945800
H	2.84911700	0.21737900	1.56957500
H	3.05295000	2.10851800	0.10938600
O	2.67735800	1.18374600	-1.64036600
C	0.60766900	2.69888500	0.65013700
H	0.28944200	0.18089900	-1.33850700
C	-1.40976900	0.68795900	-0.21894700
H	-0.71660900	-0.40249700	2.29178300
H	0.61070000	-1.53389600	2.56821700
H	0.92080700	0.19880000	2.40134100
C	-1.92516800	-1.66703900	0.35647200
H	-0.34131400	-2.12100000	-1.01070700
H	-0.16934600	-2.91175400	0.55527300
C	-2.32170900	-0.24515000	0.04235500
H	-2.15946500	-1.86344900	1.40895500
H	-2.55652700	-2.35639300	-0.21049500
H	-1.72757800	1.69912900	-0.44783100
C	-3.81321400	0.02460800	0.04996900
H	-4.23274600	-0.58017700	0.86285200
C	-4.18844800	1.47988600	0.31669900
C	-4.45662200	-0.45701900	-1.25764400
H	-5.27138800	1.57432100	0.41896600
H	-3.72384100	1.85083500	1.23217300
H	-3.88137600	2.12893700	-0.50713800
H	-5.54316400	-0.35565800	-1.21159100
H	-4.09381900	0.14190600	-2.09693300
H	-4.22728400	-1.50369000	-1.46491100
H	-0.42463100	2.90285500	0.90826700
H	1.33571800	3.46452100	0.89433700
H	1.82935100	-2.37522500	-1.35507700
H	4.25547900	-0.62060400	-0.69713900
H	2.39037200	0.30963100	-1.93328600

2a_2

C	0.43489000	-0.90913700	0.29771100
C	1.93576900	-1.20574900	0.10144700
C	2.82881000	-0.08081300	0.65191800
C	2.42262900	1.30101500	0.10630500
C	0.94393100	1.53263600	0.29350300
C	0.10037700	0.45951800	-0.34504000
C	0.12525000	-0.92500200	1.80139200
C	-0.42558100	-1.95856200	-0.41484000
O	2.29817600	-1.46664200	-1.25322800
H	2.17688300	-2.12805500	0.63622500

O	4.18947600	-0.34173800	0.35040500
H	2.74328600	-0.04915300	1.73840900
H	2.99209300	2.06856100	0.63786400
O	2.71194000	1.37857200	-1.29521000
C	0.49215900	2.55135700	1.01227300
H	0.43662200	0.40386300	-1.38921200
C	-1.37954800	0.71612400	-0.39788200
H	-0.92118600	-0.68426900	1.98596100
H	0.32347300	-1.91773000	2.21234400
H	0.71722000	-0.20101600	2.35930800
C	-1.91731400	-1.69511800	-0.21705600
H	-0.18792000	-1.94000100	-1.48008500
H	-0.17276000	-2.95791700	-0.04891200
C	-2.30365800	-0.24152900	-0.36097800
H	-2.23177800	-2.05364500	0.76823500
H	-2.49172900	-2.28669900	-0.93636300
H	-1.67928300	1.74917700	-0.53116900
C	-3.79229300	0.03518600	-0.44136300
H	-4.18740800	-0.61643100	-1.23048800
C	-4.49121200	-0.36152700	0.86688100
C	-4.15091400	1.47163500	-0.80863600
H	-5.56563900	-0.18070800	0.79428400
H	-4.34779500	-1.41576100	1.10639200
H	-4.10183500	0.23253400	1.69823500
H	-5.23221700	1.56575000	-0.92499700
H	-3.84147300	2.17067100	-0.02707700
H	-3.68253200	1.77917300	-1.74540500
H	-0.56587800	2.70130900	1.18982900
H	1.17326200	3.26818800	1.45644800
H	2.24748800	-0.63092400	-1.73531500
H	4.19486600	-0.89928500	-0.43658200
H	3.66772300	1.32061800	-1.38749000

2a_20

C	0.39613200	-0.77864100	0.43082100
C	1.84373900	-1.23760000	0.19354300
C	2.87032100	-0.15389200	0.56144800
C	2.58509200	1.17628900	-0.14874300
C	1.14770000	1.58259200	0.12143600
C	0.15689100	0.54509300	-0.33957500
C	0.17870600	-0.60850000	1.94124900
C	-0.60202200	-1.80618600	-0.11744300
O	2.02034100	-1.59402500	-1.17924900
H	2.03196400	-2.12156900	0.81662200
O	4.17934200	-0.58624600	0.21261500
H	2.81744300	0.03725300	1.63557400
H	3.26182600	1.93255000	0.25191800
O	2.85071600	1.11489200	-1.54523100
C	0.85481200	2.71885300	0.73934200
H	0.41266800	0.33347800	-1.38570100
C	-1.29211000	0.94114800	-0.33088800
H	-0.83293100	-0.26310900	2.15008200

H	0.32328100	-1.56434100	2.45016200
H	0.85660300	0.12022000	2.38328300
C	-2.04979200	-1.37650600	0.11837100
H	-0.42743900	-1.92492100	-1.18800700
H	-0.42374000	-2.78070600	0.34644200
C	-2.29805300	0.08653500	-0.15690400
H	-2.34682700	-1.59409400	1.14979200
H	-2.71087000	-1.98463600	-0.50461100
H	-1.51768600	1.98359800	-0.53815300
C	-3.73687500	0.55544200	-0.21591800
H	-3.71292700	1.64727100	-0.28217800
C	-4.45469800	0.03424600	-1.46558600
C	-4.51369200	0.18150000	1.04964300
H	-5.46433200	0.44726500	-1.52332300
H	-3.91778200	0.31734100	-2.37297700
H	-4.54511200	-1.05460200	-1.44916900
H	-5.51018300	0.62774800	1.02931700
H	-4.63812000	-0.90090600	1.13311500
H	-4.00010300	0.53395300	1.94685100
H	-0.16614600	3.00776000	0.95835900
H	1.63740100	3.40016800	1.05448900
H	2.93447900	-1.88275900	-1.26839000
H	4.47678400	-1.21254800	0.87656200
H	2.54163500	0.25638700	-1.85992500

2a_3

C	0.46749100	-0.84123600	0.52146200
C	1.94174700	-1.18357300	0.22261100
C	2.88361100	-0.00025900	0.50232100
C	2.42196100	1.28783500	-0.20447700
C	0.96986700	1.56505100	0.09479800
C	0.06656200	0.41996900	-0.28491700
C	0.31259000	-0.62554500	2.03348000
C	-0.45513800	-1.97880300	0.06881500
O	2.16549700	-1.65288300	-1.10554800
H	2.23793500	-2.01693800	0.86497700
O	4.20731400	-0.32273500	0.11001000
H	2.90765800	0.19739500	1.57430500
H	3.04165700	2.11911200	0.14324500
O	2.56646100	1.14764200	-1.62334100
C	0.59315800	2.68637600	0.69430500
H	0.29383600	0.20165800	-1.33708100
C	-1.41141200	0.68577800	-0.22138900
H	-0.71228400	-0.35566500	2.28546900
H	0.56324400	-1.54459600	2.56843600
H	0.94987700	0.17283400	2.41085300
C	-1.92340000	-1.66933000	0.35399400
H	-0.31731800	-2.13376100	-1.00291500
H	-0.16675800	-2.91003900	0.56495600
C	-2.32237100	-0.24946100	0.03509900
H	-2.16047800	-1.86019600	1.40724700
H	-2.55449400	-2.36104800	-0.21070000

H	-1.72916300	1.69643800	-0.45302600
C	-3.81438800	0.01750200	0.03360300
H	-4.23740100	-0.58860100	0.84368000
C	-4.19465100	1.47180300	0.29850000
C	-4.44835400	-0.46538500	-1.27807900
H	-5.27842000	1.56374800	0.39422800
H	-3.73648900	1.84345800	1.21695000
H	-3.88408000	2.12192000	-0.52319700
H	-5.53547800	-0.36724400	-1.23874500
H	-4.08215800	0.13491200	-2.11492300
H	-4.21446100	-1.51126200	-1.48421200
H	-0.44088200	2.87626300	0.95533800
H	1.31547600	3.45219000	0.95293200
H	2.06092800	-0.90090800	-1.70288200
H	4.13377900	-0.98940900	-0.58307300
H	3.50764500	1.05920900	-1.80158800

2a_4

C	0.38975300	-0.78194000	0.44999000
C	1.83149200	-1.25734800	0.20794300
C	2.86883200	-0.19065000	0.59657400
C	2.58964400	1.15581700	-0.09170600
C	1.14735000	1.57152700	0.10263400
C	0.15998200	0.52632000	-0.34978800
C	0.18202400	-0.57504200	1.95679900
C	-0.61946300	-1.81406600	-0.06888800
O	1.98867800	-1.58703600	-1.17597000
H	2.01217600	-2.15428400	0.81087400
O	4.17780500	-0.63999400	0.28693600
H	2.83364700	-0.04137700	1.67567300
H	3.24997300	1.91132900	0.33555100
O	2.95584400	1.07546900	-1.47201600
C	0.84416700	2.73010300	0.67310100
H	0.42046600	0.29152500	-1.39036100
C	-1.28719300	0.92988000	-0.36067500
H	-0.83258500	-0.23755400	2.16451400
H	0.34293800	-1.51556800	2.48892500
H	0.85310600	0.17345200	2.37523800
C	-2.06306600	-1.36716200	0.15717600
H	-0.44759100	-1.96274100	-1.13603800
H	-0.44780600	-2.77708800	0.42080600
C	-2.29957100	0.08813000	-0.16337900
H	-2.36014800	-1.55059900	1.19516400
H	-2.73010900	-1.98877600	-0.44580600
H	-1.50590400	1.96598400	-0.60344800
C	-3.73463200	0.56552600	-0.24270400
H	-3.70267600	1.65477700	-0.34036300
C	-4.45053200	0.01331800	-1.48008100
C	-4.51938400	0.23363500	1.02964500
H	-5.45691700	0.43156900	-1.55427600
H	-3.90769700	0.26635400	-2.39281900
H	-4.54878700	-1.07391900	-1.43259700

H	-4.65111600	-0.84505500	1.14409200
H	-4.00755100	0.60891300	1.91854800
H	-5.51284400	0.68541400	0.99159700
H	-0.18047200	3.03257800	0.85159100
H	1.62143700	3.41641500	0.98996400
H	2.81560200	-2.06768500	-1.26436300
H	4.40238300	-0.27058600	-0.57478600
H	2.52544500	0.29111000	-1.83911800

2a_5

C	0.43122500	-0.91830600	0.29651400
C	1.92440600	-1.21421300	0.08245600
C	2.82477900	-0.10714400	0.65628500
C	2.43448200	1.28191000	0.12382800
C	0.94869500	1.52278400	0.28186800
C	0.10092900	0.44947500	-0.35185800
C	0.13475000	-0.92409200	1.80282600
C	-0.44207500	-1.96856100	-0.40115400
O	2.17807500	-1.34939200	-1.31952300
H	2.17150500	-2.15749300	0.58231600
O	4.18597900	-0.38021600	0.36675100
H	2.72815700	-0.10097500	1.74190400
H	2.99030400	2.03865700	0.67861200
O	2.86987200	1.41667700	-1.23193800
C	0.49652900	2.56269800	0.97022000
H	0.43462800	0.37494300	-1.39540600
C	-1.37792400	0.71202500	-0.40458900
H	-0.91568700	-0.70515200	1.99059300
H	0.35798200	-1.90705800	2.22421100
H	0.71198200	-0.17943900	2.34851500
C	-1.93058800	-1.69385600	-0.19543600
H	-0.21331600	-1.96002700	-1.46790900
H	-0.19275600	-2.96582200	-0.02705200
C	-2.30778000	-0.23953400	-0.35433500
H	-2.24196100	-2.03950600	0.79538100
H	-2.51294600	-2.28959700	-0.90486800
H	-1.67296300	1.74459900	-0.55076500
C	-3.79490000	0.04551800	-0.43503500
H	-4.19515900	-0.61166500	-1.21693100
C	-4.49390300	-0.33407000	0.87820200
C	-4.14577000	1.48022000	-0.81631700
H	-5.56751800	-0.14855100	0.80535600
H	-4.35537700	-1.38652600	1.12826700
H	-4.10021900	0.26649900	1.70282100
H	-3.83050900	2.18545600	-0.04269400
H	-3.67765200	1.77529400	-1.75722300
H	-5.22674800	1.57954700	-0.93140900
H	-0.56207200	2.73481500	1.12232500
H	1.17972700	3.27778000	1.41450300
H	3.05846100	-1.72121400	-1.41669400
H	4.40654200	0.11914000	-0.42793000
H	2.53704700	0.65062600	-1.71908300

2a_6

C	0.46230500	-0.85119300	0.51884600
C	1.92679100	-1.19613100	0.20265000
C	2.87967700	-0.02985700	0.51335300
C	2.43783800	1.26958600	-0.18034400
C	0.97552300	1.55329200	0.08760200
C	0.06669400	0.41115500	-0.28966900
C	0.32051800	-0.63027200	2.03130000
C	-0.47244600	-1.98488300	0.07827000
O	2.03792300	-1.53989000	-1.18230400
H	2.22362100	-2.05923000	0.80907000
O	4.20551000	-0.36272200	0.13560300
H	2.89110100	0.13922700	1.59001500
H	3.04632600	2.09232800	0.19674600
O	2.73517700	1.19379500	-1.57739500
C	0.59659600	2.69108700	0.65481900
H	0.29048500	0.17701300	-1.33885200
C	-1.40986900	0.68469000	-0.22576400
H	0.60022400	-1.53891200	2.56960200
H	0.93920800	0.18756000	2.39732400
H	-0.70842200	-0.38580200	2.29159400
C	-1.93683900	-1.66272700	0.36872800
H	-0.34236500	-2.14693900	-0.99278200
H	-0.18820100	-2.91441600	0.58012600
C	-2.32633800	-0.24261400	0.03998000
H	-2.57500000	-2.35375400	-0.18885800
H	-2.17202600	-1.84466800	1.42392300
H	-1.72323800	1.69449100	-0.46651800
C	-3.81666100	0.03435600	0.03828200
H	-4.24276400	-0.56288500	0.85330300
C	-4.18716500	1.49304000	0.29286500
C	-4.45531700	-0.45407100	-1.26907400
H	-3.87379100	2.13502300	-0.53412900
H	-5.27017700	1.59267400	0.38954400
H	-3.72515100	1.86860300	1.20778900
H	-4.22887900	-1.50309900	-1.46744100
H	-5.54169200	-0.34807500	-1.22952800
H	-4.08571900	0.13723500	-2.11080900
H	-0.44055000	2.90085000	0.88648900
H	1.32213000	3.45345400	0.91555100
H	2.90272700	-1.93802600	-1.31011700
H	4.34559200	0.00918300	-0.74286600
H	2.35983700	0.36595500	-1.90747900

2a_7

C	0.39684700	-0.78974800	0.42898600
C	1.84770800	-1.26049300	0.17827300
C	2.87357600	-0.18696700	0.58211800
C	2.58674600	1.15730800	-0.10238300
C	1.15215100	1.57365700	0.13845300
C	0.15958000	0.54049400	-0.32962700
C	0.18154900	-0.62995800	1.94049500

C	-0.60542200	-1.80803200	-0.12658300
O	2.08695700	-1.67729600	-1.16203600
H	2.03014200	-2.14859200	0.78868900
O	4.19501100	-0.62380100	0.34307500
H	2.80666900	-0.02128900	1.65643300
H	3.27224000	1.90938400	0.29822600
O	2.84899900	0.96833200	-1.49905200
C	0.86483200	2.71657900	0.74769100
H	0.41640500	0.34267600	-1.37891100
C	-1.28774200	0.94250200	-0.32028400
H	-0.83699100	-0.31034800	2.15797800
H	0.35243900	-1.58416900	2.44464300
H	0.84408500	0.11262200	2.38313600
C	-2.05294700	-1.37650400	0.10680500
H	-0.42610400	-1.92193700	-1.19723200
H	-0.43171900	-2.78629700	0.33117000
C	-2.29651200	0.08946600	-0.15541500
H	-2.35484500	-1.60254600	1.13500600
H	-2.71383700	-1.97671600	-0.52409000
H	-1.51019500	1.98723600	-0.51948700
C	-3.73368000	0.56348200	-0.21353500
H	-3.70631200	1.65581700	-0.26971100
C	-4.44993400	0.05590900	-1.46971300
C	-4.51472500	0.18030900	1.04663700
H	-5.45812200	0.47259300	-1.52614500
H	-3.90991800	0.34557300	-2.37320400
H	-4.54371500	-1.03273700	-1.46344400
H	-5.50967800	0.63003400	1.02801800
H	-4.64286500	-0.90238900	1.11971500
H	-4.00216400	0.52270000	1.94830500
H	-0.15483900	3.01354800	0.96051400
H	1.65124400	3.39280500	1.06385900
H	2.15360200	-0.88364700	-1.70902200
H	4.22531400	-0.95095000	-0.56143400
H	2.66926200	1.79072000	-1.96041900

2a_8

C	0.43120000	-0.91837400	0.29633700
C	1.92438600	-1.21418200	0.08229000
C	2.82462000	-0.10714300	0.65639100
C	2.43440700	1.28195500	0.12388200
C	0.94857500	1.52280800	0.28160800
C	0.10085900	0.44942100	-0.35205000
C	0.13491300	-0.92406900	1.80265700
C	-0.44210700	-1.96871000	-0.40119300
O	2.17816700	-1.34920800	-1.31975900
H	2.17155300	-2.15751200	0.58199900
O	4.18594200	-0.38009400	0.36744700
H	2.72763200	-0.10104600	1.74197800
H	2.99005300	2.03870000	0.67883800
O	2.87018100	1.41681400	-1.23174600
C	0.49628000	2.56274800	0.96982500

H	0.43444400	0.37491700	-1.39562100
C	-1.37801300	0.71191400	-0.40461100
H	-0.91550300	-0.70515800	1.99055700
H	0.35825700	-1.90699100	2.22408600
H	0.71223900	-0.17934500	2.34815800
C	-1.93062400	-1.69402100	-0.19548700
H	-0.21337600	-1.96028700	-1.46794400
H	-0.19277700	-2.96596000	-0.02703800
C	-2.30782400	-0.23968200	-0.35429000
H	-2.24203400	-2.03980500	0.79526700
H	-2.51294900	-2.28967800	-0.90501600
H	-1.67317600	1.74447900	-0.55064100
C	-3.79493100	0.04538900	-0.43481600
H	-4.19541300	-0.61205300	-1.21638400
C	-4.49368100	-0.33357000	0.87873200
C	-4.14569400	1.47997900	-0.81658000
H	-5.56729000	-0.14795400	0.80605700
H	-4.35523500	-1.38594300	1.12919400
H	-4.09975100	0.26726900	1.70303900
H	-5.22667500	1.57937700	-0.93155600
H	-3.83022900	2.18548600	-0.04327900
H	-3.67767400	1.77463200	-1.75767100
H	-0.56234400	2.73484200	1.12179600
H	1.17938700	3.27790700	1.41413100
H	3.05817900	-1.72186600	-1.41697700
H	4.40645700	0.11849800	-0.42773400
H	2.53770400	0.65061400	-1.71892600

2a_9

C	0.43789700	-0.91740200	0.28242800
C	1.94052200	-1.21075800	0.06962300
C	2.82685100	-0.09492200	0.65064900
C	2.43358800	1.28473300	0.10296800
C	0.95262400	1.52620100	0.29725800
C	0.09967300	0.45938100	-0.34048600
C	0.13293400	-0.95327300	1.78661800
C	-0.42595500	-1.95680800	-0.44072700
O	2.28730700	-1.44316600	-1.29170600
H	2.18515900	-2.14126300	0.58815400
O	4.19709100	-0.36710300	0.44484900
H	2.69135000	-0.06266000	1.73072800
H	3.01447900	2.04929300	0.62647700
O	2.78492100	1.28723100	-1.28695700
C	0.51479500	2.55854300	1.00627600
H	0.42890500	0.40939000	-1.38707800
C	-1.37981600	0.71919000	-0.38017900
H	-0.92222200	-0.75837000	1.97526500
H	0.37393300	-1.93847100	2.19307800
H	0.69379400	-0.20691700	2.34747700
C	-1.91813700	-1.69497600	-0.24130200
H	-0.18681400	-1.92696100	-1.50525300
H	-0.17462400	-2.96052900	-0.08565300

C	-2.30416900	-0.23871100	-0.35641100
H	-2.23625500	-2.07344900	0.73533200
H	-2.49141900	-2.27112100	-0.97401300
H	-1.68061800	1.75437600	-0.49332800
C	-3.79314400	0.04020400	-0.42425500
H	-4.19403000	-0.60255600	-1.21768200
C	-4.48434000	-0.36941400	0.88412200
C	-4.15283100	1.48078400	-0.77419200
H	-4.08989500	0.21641300	1.71891200
H	-5.55920600	-0.18789500	0.81984800
H	-4.33960700	-1.42595100	1.11241800
H	-5.23468000	1.57678700	-0.88383400
H	-3.83902100	2.17131800	0.01315000
H	-3.68904500	1.79794200	-1.71005800
H	-0.54038400	2.72648000	1.18450200
H	1.20755700	3.26948900	1.44250300
H	2.29530700	-0.58871800	-1.74258600
H	4.30689200	-0.57524400	-0.48826800
H	2.54294400	2.13629500	-1.66391600

2b_1

C	-0.16042400	0.53615800	-0.34206000
C	-0.39430800	-0.77403200	0.45195800
C	-1.84388700	-1.24996200	0.22473700
C	-2.87606700	-0.16777800	0.58485200
C	-2.57753400	1.17379800	-0.10997800
C	-1.14336800	1.58212600	0.11637800
H	-0.42625200	0.32006300	-1.38560500
C	1.28826200	0.93403700	-0.35356500
C	-0.17531800	-0.57502000	1.95838400
C	0.60540200	-1.80769500	-0.07935000
H	-2.02362500	-2.12026300	0.86114700
O	-2.09616700	-1.71265900	-1.10064100
O	-4.18317000	-0.60564400	0.25288900
H	-2.86070500	0.00279900	1.66162600
O	-2.78926900	1.05117900	-1.52180800
H	-3.25166600	1.93448100	0.29373000
C	-0.83992600	2.72195400	0.72231800
H	-0.30328900	-1.52532200	2.48203100
H	0.83081400	-0.20922500	2.16056300
H	-0.86557700	0.14794600	2.39062600
H	0.43025400	-2.77428100	0.40183300
H	0.42806400	-1.94733100	-1.14739200
C	2.05242000	-1.37077200	0.14470100
C	2.29673100	0.08550400	-0.16597900
H	2.71458400	-1.99227500	-0.46369800
H	2.35005800	-1.56255700	1.18107300
H	1.51017900	1.97156500	-0.58765100
C	3.73410700	0.55547100	-0.24583100
C	4.44406900	0.00681600	-1.48820200
H	3.70785100	1.64545300	-0.33679000
C	4.51985000	0.21144500	1.02267900

H	3.90029000	0.26785400	-2.39811200
H	5.45238500	0.42036000	-1.56250000
H	4.53690100	-1.08115900	-1.44720000
H	4.01226100	0.58444100	1.91496000
H	4.64547000	-0.86866800	1.13037800
H	5.51584200	0.65762600	0.98495200
H	-1.61502900	3.41006200	1.03979300
H	0.18498000	3.00417600	0.93020900
H	-3.72555600	0.87004400	-1.64853800
H	-4.08540000	-1.24759300	-0.46025700
H	-2.09405500	-0.94350200	-1.68495800

2b_10

C	-0.17938200	0.50176400	-0.33800400
C	-0.38831200	-0.80412200	0.47010400
C	-1.81759200	-1.30854200	0.21684000
C	-2.88316000	-0.23703600	0.53063700
C	-2.58443600	1.16591300	-0.02241700
C	-1.13638900	1.54982500	0.16330100
H	-0.50403800	0.27150500	-1.36058000
C	1.26466600	0.90916200	-0.39827500
C	-0.19524700	-0.58372000	1.97685600
C	0.64228500	-1.82379200	-0.03129100
H	-2.00655700	-2.15476500	0.89247800
O	-1.93118700	-1.75698400	-1.12281500
O	-4.15202600	-0.67277800	0.06729100
H	-2.95080900	-0.14684800	1.61575900
O	-2.95441400	1.14750000	-1.40478600
H	-3.22539900	1.87782900	0.50682800
C	-0.80759800	2.69246000	0.75274500
H	-0.24792200	-1.53938200	2.50362000
H	0.77719700	-0.13556100	2.18108100
H	-0.94401700	0.08149200	2.40494400
H	0.47892700	-2.78613100	0.46286000
H	0.48564700	-1.98143100	-1.09948600
C	2.07460900	-1.35174800	0.20577400
C	2.29143300	0.09089900	-0.17770600
H	2.76181100	-1.98952800	-0.35642200
H	2.35222300	-1.48464600	1.25697900
H	1.46821400	1.93714400	-0.68691100
C	3.71907400	0.58184800	-0.29597400
C	4.42819500	-0.01446700	-1.51663600
H	3.67288300	1.66555900	-0.43973000
C	4.52144300	0.31350600	0.98034700
H	4.53979000	-1.09751200	-1.42405200
H	3.87247600	0.19304100	-2.43312000
H	5.42878700	0.41153500	-1.62007400
H	5.51181300	0.76828700	0.90980300
H	4.01781500	0.72622700	1.85726700
H	4.66040700	-0.75819000	1.14244500
H	-1.56998300	3.37823100	1.10558000
H	0.22466000	2.98027800	0.91064800

H	-3.02263800	2.05512300	-1.70815200
H	-4.30982400	-0.19714600	-0.75841200
H	-2.86797700	-1.90870700	-1.27870100

2b_11

C	-0.17971100	0.51566800	-0.35068300
C	-0.39736700	-0.76721300	0.48804200
C	-1.83798500	-1.26273700	0.26577600
C	-2.89119000	-0.17241100	0.52748900
C	-2.57735700	1.21056600	-0.08719100
C	-1.13126900	1.58203100	0.12351100
H	-0.50315200	0.26372900	-1.36867400
C	1.26844500	0.90825500	-0.41632500
C	-0.19194900	-0.52079900	1.98894800
C	0.61643300	-1.80862600	-0.00178300
H	-2.03475000	-2.08199600	0.96750100
O	-2.07380200	-1.73476900	-1.06124300
O	-4.15983200	-0.61588700	0.07554200
H	-2.96437700	-0.02884800	1.60652400
O	-2.84608000	1.23230000	-1.48194700
H	-3.22032400	1.94211200	0.41609300
C	-0.79080500	2.71700100	0.71975200
H	-0.94088100	0.14915900	2.40904300
H	-0.23727000	-1.46719600	2.53304500
H	0.78025200	-0.06557900	2.17775700
H	0.44537700	-2.76442600	0.50342300
H	0.46462300	-1.97239200	-1.07170400
C	2.05543200	-1.35122200	0.22382200
C	2.28676600	0.08345900	-0.18154000
H	2.73272900	-2.00556800	-0.33093700
H	2.33229100	-1.47294900	1.27635500
H	1.48133000	1.93002100	-0.71952600
C	3.71974400	0.55714000	-0.30438300
C	4.42529100	-0.06592000	-1.51364800
H	3.68495600	1.63876000	-0.46541500
C	4.51628800	0.30074200	0.97810300
H	4.52634500	-1.14834600	-1.40317500
H	3.87350400	0.13207000	-2.43457700
H	5.43016900	0.34861000	-1.62175100
H	5.51113900	0.74482000	0.90310200
H	4.01451400	0.73175000	1.84721700
H	4.64475500	-0.76963300	1.15695100
H	-1.54531000	3.42038200	1.05359100
H	0.24434900	2.98041700	0.90100800
H	-3.68953900	0.78396500	-1.60236900
H	-3.97569500	-1.25900300	-0.62192500
H	-1.76192600	-2.63860900	-1.12971000

2b_12

C	-0.16132300	0.53706900	-0.34635400
C	-0.39502800	-0.77287600	0.44733800
C	-1.84660100	-1.24175900	0.21405700

C	-2.87267100	-0.16375500	0.58579400
C	-2.59196400	1.16548200	-0.14139300
C	-1.15331900	1.58173900	0.09564800
H	-0.41963900	0.30327900	-1.38771000
C	1.28679000	0.93701300	-0.34878100
C	-0.17658700	-0.58401300	1.95438300
C	0.60620700	-1.80439400	-0.08935500
H	-2.03985200	-2.12903600	0.82641300
O	-2.11570800	-1.55392100	-1.15720800
O	-4.19148800	-0.61740900	0.36645600
H	-2.80105100	0.02735600	1.65541100
O	-2.90248600	1.07997800	-1.53018500
H	-3.26385300	1.92361700	0.26304500
C	-0.85784600	2.73217100	0.68622900
H	-0.83777000	0.16893600	2.38068100
H	-0.34710800	-1.52704300	2.47927300
H	0.84241100	-0.26047700	2.16240200
H	0.43217600	-2.77706300	0.38071000
H	0.44695300	-1.92806500	-1.16416300
C	2.05380500	-1.36991300	0.13840000
C	2.29606800	0.08948900	-0.16015700
H	2.71457600	-1.98595500	-0.47677300
H	2.35106600	-1.57296400	1.17237200
H	1.50913300	1.97608700	-0.57498000
C	3.73323400	0.56205000	-0.22734300
C	4.45393000	0.02041900	-1.46658900
H	3.70526100	1.65232000	-0.31372700
C	4.51011000	0.21440300	1.04569800
H	4.54976300	-1.06745300	-1.42940000
H	3.91619600	0.28368300	-2.37945000
H	5.46156700	0.43710600	-1.53169900
H	5.50497500	0.66378600	1.01784300
H	3.99443200	0.58172600	1.93568700
H	4.63851500	-0.86579400	1.14944400
H	-1.63946500	3.41912200	0.99125000
H	0.16378700	3.02776800	0.89264200
H	-2.55334500	0.24353600	-1.86273100
H	-4.24735300	-0.89082600	-0.55435500
H	-1.71274100	-2.39157500	-1.39014400

2b_13

C	-0.11937000	0.42757500	-0.33444100
C	-0.43316000	-0.94421200	0.31210000
C	-1.91734400	-1.26557900	0.07673800
C	-2.84833200	-0.14228200	0.58103700
C	-2.42403700	1.28442700	0.19596900
C	-0.93640900	1.49204300	0.34753700
H	-0.50910600	0.36826600	-1.35851700
C	1.35531400	0.69866800	-0.42743100
C	-0.15639600	-0.94427900	1.82212300
C	0.46388600	-1.98374000	-0.37124000
H	-2.16676200	-2.16894800	0.65118000

O	-2.13526300	-1.52299300	-1.29980100
O	-4.17451200	-0.38331400	0.13652400
H	-2.86158600	-0.18969700	1.67083600
O	-2.85208900	1.48833200	-1.15435400
H	-2.96170800	1.98039200	0.84735100
C	-0.46124000	2.50806400	1.05683500
H	-0.27931000	-1.95265500	2.22418700
H	0.86217300	-0.61657800	2.03012200
H	-0.81915800	-0.27719400	2.37173700
H	0.22067900	-2.98363000	0.00029300
H	0.25174800	-1.97873500	-1.44143500
C	1.94388100	-1.68810500	-0.14491100
C	2.30102700	-0.23596600	-0.35721100
H	2.54774300	-2.30441800	-0.81804600
H	2.23748600	-1.98941700	0.86582600
H	1.63431300	1.73021700	-0.61084000
C	3.78242200	0.06580700	-0.47167000
C	4.51185700	-0.29167300	0.83102100
H	4.17475200	-0.59578600	-1.25411700
C	4.10887100	1.49986600	-0.87585700
H	4.12768000	0.31266300	1.65747400
H	4.38991200	-1.34301400	1.09420500
H	5.58169100	-0.09493500	0.73513500
H	5.18664000	1.61197700	-1.00829000
H	3.62330100	1.77716500	-1.81332800
H	3.79506000	2.21036600	-0.10638600
H	-1.12905500	3.21534700	1.53610800
H	0.60207400	2.66617700	1.19053400
H	-2.83528400	2.43062600	-1.33414800
H	-4.31550900	0.21051000	-0.61196300
H	-3.08868200	-1.55859500	-1.42046100

2b_14

C	-0.11850100	0.44345900	-0.33766100
C	-0.44178900	-0.91292400	0.33220500
C	-1.93485900	-1.22329100	0.12107000
C	-2.85282800	-0.07787200	0.58220800
C	-2.41305400	1.33913400	0.14721700
C	-0.92751500	1.52635700	0.32600100
H	-0.50851400	0.37041200	-1.36083400
C	1.35916200	0.69701800	-0.43516400
C	-0.15470900	-0.89884900	1.83996400
C	0.43584900	-1.97157000	-0.34621600
H	-2.19274700	-2.10805400	0.71501600
O	-2.27058900	-1.47933300	-1.24316900
O	-4.17787800	-0.32050600	0.13979400
H	-2.87104800	-0.08140000	1.67303300
O	-2.73344700	1.58792700	-1.21432800
H	-2.95098000	2.05378400	0.78109300
C	-0.43990400	2.52700800	1.04748000
H	-0.81677000	-0.22754800	2.38505100
H	-0.27286900	-1.90345200	2.25300500
H	0.86428800	-0.56662600	2.03837300

H	0.18328700	-2.96716100	0.03191600
H	0.22868300	-1.96657000	-1.41935400
C	1.92076300	-1.69386200	-0.12945900
C	2.29403300	-0.24779500	-0.35657400
H	2.51311400	-2.32424600	-0.79940100
H	2.21254400	-1.99031300	0.88303500
H	1.64906600	1.72371000	-0.62823600
C	3.77884700	0.03462400	-0.47500700
C	4.50339900	-0.31020200	0.83384400
H	4.16385900	-0.64374200	-1.24658200
C	4.12136800	1.45826700	-0.90195500
H	5.57564400	-0.12870300	0.73504700
H	4.12672000	0.31293500	1.64967400
H	4.36825600	-1.35534700	1.11479300
H	5.20020700	1.55571900	-1.03717900
H	3.63777100	1.72628800	-1.84311200
H	3.81685200	2.18437500	-0.14347100
H	-1.09785000	3.25177400	1.51358400
H	0.62479900	2.65456700	1.20278600
H	-3.62615400	1.25315000	-1.34800600
H	-4.09041500	-0.87262600	-0.64859100
H	-2.06400000	-2.39264100	-1.44773700

2b_15

C	-0.08606300	0.39840200	-0.26631400
C	-0.46200800	-0.88002700	0.52473600
C	-1.91438200	-1.25149900	0.18633800
C	-2.89331900	-0.08608100	0.44100600
C	-2.43542000	1.28108800	-0.09178400
C	-0.97162200	1.53110900	0.17966200
H	-0.36884800	0.19543300	-1.30683800
C	1.38989600	0.67717500	-0.24018000
C	-0.33887700	-0.67414100	2.04085000
C	0.49726100	-1.99255800	0.08293500
H	-2.21926300	-2.07555800	0.84684500
O	-1.99044500	-1.69004800	-1.15931900
O	-4.16876600	-0.40507300	-0.09380800
H	-3.01372800	0.01399000	1.52061300
O	-2.72347000	1.29087800	-1.49356600
H	-3.03841500	2.05083600	0.39982100
C	-0.57453600	2.64066100	0.78999300
H	-0.51299900	-1.61888500	2.56114800
H	0.65759100	-0.31939600	2.30382300
H	-1.04536700	0.06018700	2.42561800
H	0.22045800	-2.93273600	0.56919700
H	0.38519100	-2.14273200	-0.99184500
C	1.95059800	-1.64868600	0.39522900
C	2.32185000	-0.23213300	0.03402400
H	2.60825100	-2.34451500	-0.13309800
H	2.16671400	-1.80053800	1.45935400
H	1.68958400	1.68562900	-0.50512300
C	3.80858800	0.06192300	0.00649900

C	4.16529200	1.53033100	0.22170500
H	4.25144300	-0.51080800	0.83028800
C	4.43735600	-0.45038700	-1.29640200
H	3.70943300	1.92262200	1.13273400
H	5.24814400	1.64475400	0.30314600
H	3.83483000	2.14861000	-0.61663100
H	5.52302600	-0.33248800	-1.27254800
H	4.21945700	-1.50601500	-1.46755900
H	4.05166100	0.11714200	-2.14726100
H	-1.28992800	3.39489900	1.09871800
H	0.46884900	2.83260900	1.00914000
H	-2.69064000	2.19978400	-1.79909800
H	-4.23532100	0.07828400	-0.92727000
H	-2.92636000	-1.75823400	-1.36941300

2b_16

C	-0.10108300	0.46031100	-0.35098100
C	-0.43491900	-0.90528900	0.29782900
C	-1.93710900	-1.19569400	0.09859600
C	-2.82455900	-0.07408500	0.65319300
C	-2.43856400	1.29923900	0.07034900
C	-0.95412900	1.53768900	0.26771200
H	-0.43282000	0.38198500	-1.39487100
C	1.37887100	0.71759600	-0.39683500
C	-0.12590700	-0.92429300	1.80086200
C	0.42680900	-1.95534900	-0.41541800
H	-2.19400000	-2.12723700	0.61423700
O	-2.30197100	-1.31068200	-1.28129800
O	-4.19291100	-0.36342400	0.46098700
H	-2.68347000	-0.02283500	1.73168200
O	-2.82180300	1.41466600	-1.29796000
H	-3.00629300	2.06570600	0.59950300
C	-0.50946400	2.57176400	0.96960400
H	-0.36389800	-1.90458500	2.22071500
H	0.92918800	-0.72486900	1.98352200
H	-0.68681400	-0.17113600	2.35203200
H	0.17397800	-2.95756700	-0.05631200
H	0.20836100	-1.92702400	-1.48655100
C	1.91901900	-1.69380400	-0.21592800
C	2.30394700	-0.23909000	-0.35468600
H	2.49219600	-2.28260900	-0.93824600
H	2.23203800	-2.05807400	0.76735500
H	1.67947900	1.75063600	-0.52756100
C	3.79299500	0.03816800	-0.42498100
C	4.48311500	-0.35655700	0.88857300
H	4.19428700	-0.61374200	-1.21074200
C	4.15282200	1.47461500	-0.79105100
H	4.33898600	-1.41062900	1.12850700
H	5.55789400	-0.17516200	0.82313300
H	4.08761800	0.23827100	1.71644600
H	5.23480700	1.56951200	-0.90011400
H	3.69053100	1.78094800	-1.73122800

H	3.83749300	2.17400900	-0.01219400
H	-1.19741700	3.29135800	1.39941400
H	0.54679500	2.73538700	1.14694300
H	-2.57823500	0.59477400	-1.74583700
H	-4.32098200	-0.51495300	-0.48046700
H	-1.99730300	-2.14796500	-1.63422100

2b_17

C	-0.08578700	0.41390700	-0.27548000
C	-0.47153400	-0.84197000	0.54309900
C	-1.93608500	-1.20237200	0.23285400
C	-2.89800600	-0.01852300	0.43286300
C	-2.42032700	1.32760700	-0.15737200
C	-0.96236200	1.56388600	0.14502300
H	-0.36953100	0.19123000	-1.31189600
C	1.39337900	0.67674500	-0.25279400
C	-0.33473800	-0.61410700	2.05469000
C	0.46843000	-1.97377500	0.11006500
H	-2.24984600	-1.99763800	0.91949000
O	-2.13372800	-1.65535200	-1.10699400
O	-4.17279300	-0.34408600	-0.09563600
H	-3.02285100	0.13462400	1.50581300
O	-2.59629100	1.36994300	-1.56641100
H	-3.02364400	2.11690200	0.30616200
C	-0.55576800	2.66198400	0.76831500
H	-1.04439500	0.12047500	2.43281500
H	-0.49721900	-1.55223000	2.59066200
H	0.66118300	-0.24784600	2.30338900
H	0.18233200	-2.90806800	0.60318300
H	0.36285200	-2.12682100	-0.96697200
C	1.92722400	-1.64655400	0.41521100
C	2.31482100	-0.23901500	0.03513500
H	2.57399500	-2.35799700	-0.10525400
H	2.14063900	-1.78838200	1.48101900
H	1.70333200	1.67864300	-0.53014400
C	3.80492600	0.03661800	0.00575800
C	4.17808100	1.50377000	0.20048400
H	4.23987300	-0.52933500	0.83839300
C	4.42960300	-0.50148700	-1.28872600
H	3.72591800	1.91408100	1.10537200
H	5.26207200	1.60699700	0.28139800
H	3.85523700	2.11367400	-0.64688000
H	4.19980000	-1.55682800	-1.44558600
H	4.05204000	0.05855800	-2.14812400
H	5.51651700	-0.39597500	-1.26446200
H	-1.26079700	3.43256000	1.05917900
H	0.48629600	2.82742800	1.01480500
H	-3.47041000	1.00704600	-1.74211900
H	-4.00748200	-1.00064700	-0.78521300
H	-1.90456400	-2.58478000	-1.15743200

2b_18

C	-0.06735600	0.41984300	-0.29000400
C	-0.46842300	-0.83881600	0.51966200
C	-1.94318900	-1.17559600	0.21398900
C	-2.88070600	0.00365500	0.50233000
C	-2.43550400	1.27942300	-0.23862600
C	-0.97866900	1.56502100	0.06998200
H	-0.28944300	0.18089900	-1.33850700
C	1.40976900	0.68795900	-0.21894700
C	-0.31659200	-0.62842300	2.03202500
C	0.45718600	-1.97587000	0.06692600
H	-2.25328000	-2.02387200	0.83361100
O	-2.16537600	-1.49853300	-1.16322800
O	-4.22300600	-0.32992900	0.21945900
H	-2.84911700	0.21737900	1.56957500
O	-2.67735900	1.18374500	-1.64036600
H	-3.05295000	2.10851800	0.10938600
C	-0.60767000	2.69888500	0.65013800
H	-0.61070600	-1.53389600	2.56821700
H	0.71660900	-0.40250400	2.29178400
H	-0.92080300	0.19880100	2.40134100
H	0.16934700	-2.91175400	0.55527400
H	0.34131200	-2.12100000	-1.01070700
C	1.92516800	-1.66703900	0.35647100
C	2.32170900	-0.24514900	0.04235400
H	2.55652600	-2.35639300	-0.21049600
H	2.15946600	-1.86344900	1.40895400
H	1.72757700	1.69912900	-0.44783100
C	3.81321400	0.02460800	0.04997000
C	4.18844600	1.47988800	0.31669800
H	4.23274500	-0.58017500	0.86285300
C	4.45662400	-0.45701900	-1.25764300
H	3.72383700	1.85083800	1.23217100
H	5.27138600	1.57432300	0.41896800
H	3.88137600	2.12893700	-0.50714000
H	5.54316600	-0.35565600	-1.21158700
H	4.22728800	-1.50369000	-1.46490900
H	4.09382200	0.14190600	-2.09693100
H	-1.33571900	3.46452000	0.89433900
H	0.42463000	2.90285400	0.90826900
H	-2.39037100	0.30963100	-1.93328600
H	-4.25548100	-0.62059700	-0.69714100
H	-1.82934100	-2.37522200	-1.35508000

2b_19

C	-0.15689100	0.54509300	-0.33957300
C	-0.39613300	-0.77864000	0.43082300
C	-1.84374000	-1.23760000	0.19354500
C	-2.87032200	-0.15389200	0.56144700
C	-2.58509100	1.17628900	-0.14874500
C	-1.14770100	1.58259200	0.12143700
H	-0.41266800	0.33347800	-1.38569900
C	1.29211000	0.94114900	-0.33088600

C	-0.17870800	-0.60849800	1.94125100
C	0.60202200	-1.80618500	-0.11743900
H	-2.03196500	-2.12156700	0.81662600
O	-2.02034200	-1.59402700	-1.17924700
O	-4.17934200	-0.58624700	0.21261100
H	-2.81744700	0.03725500	1.63557200
O	-2.85071000	1.11488900	-1.54523400
H	-3.26182700	1.93255000	0.25191300
C	-0.85481300	2.71885400	0.73934100
H	-0.85661200	0.12021500	2.38328600
H	-0.32327300	-1.56434100	2.45016300
H	0.83292600	-0.26309600	2.15008300
H	0.42373900	-2.78070500	0.34644500
H	0.42744200	-1.92492100	-1.18800300
C	2.04979200	-1.37650600	0.11837600
C	2.29805300	0.08653600	-0.15690000
H	2.71087200	-1.98463600	-0.50460300
H	2.34682500	-1.59409100	1.14979800
H	1.51768600	1.98359800	-0.53815300
C	3.73687500	0.55544200	-0.21592100
C	4.45469200	0.03423900	-1.46559000
H	3.71292800	1.64727100	-0.28218400
C	4.51369700	0.18150300	1.04963900
H	4.54510500	-1.05460800	-1.44916900
H	3.91777200	0.31733200	-2.37297900
H	5.46432700	0.44725700	-1.52333300
H	5.51018900	0.62774800	1.02930600
H	4.00011300	0.53396100	1.94684800
H	4.63812300	-0.90090200	1.13311400
H	-1.63740300	3.40017000	1.05448700
H	0.16614500	3.00776200	0.95835900
H	-2.54162800	0.25638400	-1.85992600
H	-4.47678900	-1.21254200	0.87656100
H	-2.93448200	-1.88275400	-1.26838700

2b_2

C	-0.10037700	0.45951800	-0.34504000
C	-0.43489000	-0.90913700	0.29771100
C	-1.93576900	-1.20574900	0.10144700
C	-2.82881000	-0.08081300	0.65191800
C	-2.42262900	1.30101500	0.10630500
C	-0.94393100	1.53263600	0.29350300
H	-0.43662200	0.40386300	-1.38921200
C	1.37954800	0.71612400	-0.39788200
C	-0.12525000	-0.92500200	1.80139200
C	0.42558000	-1.95856200	-0.41484000
H	-2.17688300	-2.12805500	0.63622500
O	-2.29817600	-1.46664100	-1.25322800
O	-4.18947600	-0.34173700	0.35040600
H	-2.74328600	-0.04915300	1.73840900
O	-2.71193900	1.37857200	-1.29521000
H	-2.99209300	2.06856100	0.63786400

C	-0.49215900	2.55135700	1.01227300
H	-0.71721900	-0.20101500	2.35930800
H	-0.32347300	-1.91773000	2.21234400
H	0.92118600	-0.68427000	1.98596000
H	0.17276000	-2.95791700	-0.04891200
H	0.18792000	-1.94000100	-1.48008500
C	1.91731400	-1.69511800	-0.21705700
C	2.30365800	-0.24152900	-0.36097800
H	2.49172900	-2.28669900	-0.93636400
H	2.23177800	-2.05364600	0.76823400
H	1.67928300	1.74917600	-0.53116900
C	3.79229300	0.03518500	-0.44136200
C	4.49121100	-0.36152600	0.86688200
H	4.18740900	-0.61643200	-1.23048700
C	4.15091400	1.47163400	-0.80863700
H	4.10183400	0.23253700	1.69823500
H	4.34779300	-1.41576000	1.10639400
H	5.56563900	-0.18070800	0.79428500
H	3.68253200	1.77917200	-1.74540600
H	3.84147300	2.17067200	-0.02707800
H	5.23221700	1.56574900	-0.92499800
H	-1.17326200	3.26818800	1.45644800
H	0.56587800	2.70130900	1.18982900
H	-3.66772300	1.32061900	-1.38749000
H	-4.19486500	-0.89928300	-0.43658300
H	-2.24748800	-0.63092300	-1.73531500

2b_3

C	-0.06656200	0.41996900	-0.28491700
C	-0.46749100	-0.84123500	0.52146300
C	-1.94174700	-1.18357200	0.22261200
C	-2.88361100	-0.00025800	0.50232100
C	-2.42196100	1.28783500	-0.20447800
C	-0.96986800	1.56505100	0.09479800
H	-0.29383600	0.20165800	-1.33708100
C	1.41141200	0.68577800	-0.22138900
C	-0.31258900	-0.62554400	2.03348000
C	0.45513700	-1.97880300	0.06881600
H	-2.23793400	-2.01693700	0.86497900
O	-2.16549700	-1.65288400	-1.10554600
O	-4.20731300	-0.32273500	0.11000800
H	-2.90765800	0.19739600	1.57430500
O	-2.56646100	1.14764000	-1.62334100
H	-3.04165700	2.11911200	0.14324400
C	-0.59315800	2.68637600	0.69430500
H	-0.56324600	-1.54459400	2.56843700
H	0.71228600	-0.35566600	2.28546900
H	-0.94987300	0.17283700	2.41085300
H	0.16675700	-2.91003900	0.56495700
H	0.31731700	-2.13376100	-1.00291400
C	1.92339900	-1.66933000	0.35399500
C	2.32237100	-0.24946100	0.03509900

H	2.55449400	-2.36104900	-0.21070000
H	2.16047800	-1.86019500	1.40724700
H	1.72916400	1.69643800	-0.45302800
C	3.81438800	0.01750200	0.03360200
C	4.19465200	1.47180100	0.29850200
H	4.23740200	-0.58860300	0.84367700
C	4.44835200	-0.46538400	-1.27808200
H	3.73649300	1.84345300	1.21695500
H	5.27842100	1.56374600	0.39422800
H	3.88407900	2.12192200	-0.52319100
H	5.53547600	-0.36724100	-1.23875100
H	4.21446100	-1.51126100	-1.48421300
H	4.08215300	0.13491200	-2.11492500
H	-1.31547600	3.45219000	0.95293100
H	0.44088300	2.87626200	0.95533700
H	-3.50764500	1.05921000	-1.80158900
H	-4.13377700	-0.98941200	-0.58307200
H	-2.06093100	-0.90090900	-1.70288100

2b_4

C	-0.15998200	0.52632100	-0.34978700
C	-0.38975300	-0.78194000	0.44998900
C	-1.83149300	-1.25734700	0.20794300
C	-2.86883100	-0.19064800	0.59657300
C	-2.58964300	1.15581800	-0.09171000
C	-1.14735000	1.57152800	0.10263600
H	-0.42046700	0.29152700	-1.39036000
C	1.28719300	0.92988100	-0.36067300
C	-0.18202300	-0.57504500	1.95679900
C	0.61946200	-1.81406600	-0.06889100
H	-2.01217800	-2.15428300	0.81087300
O	-1.98868100	-1.58703400	-1.17597100
O	-4.17780200	-0.64000100	0.28693900
H	-2.83364200	-0.04137000	1.67567100
O	-2.95583600	1.07547200	-1.47202200
H	-3.24997300	1.91133100	0.33554500
C	-0.84416800	2.73010200	0.67310600
H	-0.85310300	0.17344900	2.37523900
H	-0.34293800	-1.51557200	2.48892300
H	0.83258700	-0.23755900	2.16451300
H	0.44780500	-2.77708800	0.42080200
H	0.44758800	-1.96274000	-1.13604100
C	2.06306500	-1.36716300	0.15717200
C	2.29957100	0.08813000	-0.16338000
H	2.73010700	-1.98877600	-0.44581300
H	2.36014900	-1.55060300	1.19515800
H	1.50590400	1.96598500	-0.60344300
C	3.73463100	0.56552600	-0.24270300
C	4.45053300	0.01332100	-1.48008000
H	3.70267500	1.65477700	-0.34035900
C	4.51938300	0.23363100	1.02964600
H	5.45691700	0.43157400	-1.55427400
H	4.54879000	-1.07391600	-1.43259800

H	3.90769800	0.26635700	-2.39281800
H	5.51284200	0.68541100	0.99160100
H	4.00754800	0.60890700	1.91855000
H	4.65111500	-0.84505900	1.14409100
H	-1.62143900	3.41641400	0.98996900
H	0.18047100	3.03257700	0.85159900
H	-2.52545000	0.29110200	-1.83911600
H	-4.40240900	-0.27055900	-0.57476000
H	-2.81560600	-2.06768200	-1.26436200

2b_5

C	-0.10093300	0.44949500	-0.35186200
C	-0.43121400	-0.91828900	0.29651100
C	-1.92440200	-1.21420900	0.08251200
C	-2.82477300	-0.10711900	0.65630800
C	-2.43448800	1.28191600	0.12379400
C	-0.94870700	1.52280600	0.28184800
H	-0.43462700	0.37495900	-1.39541200
C	1.37791600	0.71205000	-0.40459400
C	-0.13465800	-0.92410700	1.80280700
C	0.44205900	-1.96853000	-0.40120600
H	-2.17148300	-2.15746600	0.58242900
O	-2.17810900	-1.34945900	-1.31944800
O	-4.18597400	-0.38022400	0.36679400
H	-2.72814300	-0.10090600	1.74192500
O	-2.86984200	1.41662000	-1.23199000
H	-2.99032900	2.03868000	0.67853500
C	-0.49655000	2.56273100	0.97018900
H	-0.35782200	-1.90709600	2.22417400
H	0.91578100	-0.70512600	1.99052100
H	-0.71188700	-0.17949800	2.34855600
H	0.19273600	-2.96580200	-0.02713300
H	0.21328400	-1.95995500	-1.46795800
C	1.93057500	-1.69383800	-0.19550100
C	2.30776900	-0.23951300	-0.35437100
H	2.51292400	-2.28956500	-0.90495100
H	2.24195700	-2.03950600	0.79530800
H	1.67295100	1.74462800	-0.55074900
C	3.79489100	0.04553300	-0.43504000
C	4.49386400	-0.33417300	0.87817800
H	4.19514900	-0.61159900	-1.21697900
C	4.14578600	1.48025900	-0.81620900
H	4.10020900	0.26637300	1.70283000
H	4.35526800	-1.38663500	1.12818000
H	5.56749100	-0.14871600	0.80535300
H	3.83056600	2.18544000	-0.04251900
H	5.22676300	1.57956900	-0.93132100
H	3.67764700	1.77542200	-1.75707700
H	-1.17975300	3.27781300	1.41446400
H	0.56205100	2.73485000	1.12229300
H	-2.53697800	0.65056700	-1.71910300
H	-4.40659100	0.11917600	-0.42784400

H	-3.05855400	-1.72115800	-1.41656400

2b_6

C	-0.06669300	0.41115400	-0.28966700
C	-0.46230500	-0.85119400	0.51884800
C	-1.92679100	-1.19613100	0.20265100
C	-2.87967800	-0.02985400	0.51334700
C	-2.43783500	1.26958800	-0.18034800
C	-0.97552000	1.55329200	0.08760400
H	-0.29048700	0.17701300	-1.33885000
C	1.40987000	0.68468600	-0.22577000
C	-0.32052300	-0.63027300	2.03130400
C	0.47244500	-1.98488600	0.07827400
H	-2.22362200	-2.05922700	0.80907400
O	-2.03792100	-1.53989400	-1.18230200
O	-4.20551000	-0.36272400	0.13559700
H	-2.89110100	0.13923300	1.59000900
O	-2.73516600	1.19380500	-1.57740100
H	-3.04632100	2.09233200	0.19674300
C	-0.59659300	2.69108300	0.65483000
H	-0.93922000	0.18755500	2.39732700
H	0.70841400	-0.38579400	2.29159900
H	-0.60022300	-1.53891400	2.56960500
H	0.18820000	-2.91441700	0.58013500
H	0.34236000	-2.14694600	-0.99277700
C	1.93683900	-1.66273000	0.36872700
C	2.32633800	-0.24262000	0.03997000
H	2.17202800	-1.84466600	1.42392200
H	2.57499700	-2.35376100	-0.18885800
H	1.72324100	1.69448700	-0.46652100
C	3.81666000	0.03435300	0.03828000
C	4.18715600	1.49303500	0.29287900
H	4.24276400	-0.56289600	0.85329400
C	4.45531700	-0.45405600	-1.26908300
H	3.87376500	2.13502800	-0.53410200
H	5.27016900	1.59267700	0.38954400
H	3.72515200	1.86858200	1.20781500
H	4.22888200	-1.50308300	-1.46746400
H	4.08571800	0.13725900	-2.11081100
H	5.54169200	-0.34805800	-1.22953400
H	-1.32212600	3.45345000	0.91556200
H	0.44055300	2.90084200	0.88650700
H	-2.35985000	0.36595200	-1.90748400
H	-4.34561600	0.00921900	-0.74285100
H	-2.90272200	-1.93803900	-1.31011300

2b_7

C	-0.15958000	0.54049400	-0.32962800
C	-0.39684800	-0.78974600	0.42898700
C	-1.84770900	-1.26049100	0.17827600
C	-2.87357700	-0.18696300	0.58211800
C	-2.58674500	1.15731000	-0.10238600

C	-1.15215100	1.57365800	0.13845200
H	-0.41640500	0.34267600	-1.37891200
C	1.28774300	0.94250200	-0.32028500
C	-0.18154800	-0.62995700	1.94049500
C	0.60542000	-1.80803200	-0.12658300
H	-2.03014400	-2.14858900	0.78869500
O	-2.08696200	-1.67729600	-1.16203200
O	-4.19501100	-0.62380000	0.34307500
H	-2.80666900	-0.02128200	1.65643300
O	-2.84899500	0.96833300	-1.49905600
H	-3.27224000	1.90938700	0.29821900
C	-0.86483200	2.71657900	0.74769100
H	-0.35243600	-1.58416800	2.44464400
H	0.83699200	-0.31034500	2.15797600
H	-0.84408400	0.11262300	2.38313700
H	0.43171700	-2.78629700	0.33117000
H	0.42610100	-1.92193700	-1.19723200
C	2.05294600	-1.37650500	0.10680400
C	2.29651100	0.08946600	-0.15541600
H	2.71383400	-1.97671700	-0.52409300
H	2.35484500	-1.60254800	1.13500400
H	1.51019500	1.98723600	-0.51948700
C	3.73368000	0.56348000	-0.21353500
C	4.44993500	0.05590500	-1.46971100
H	3.70631300	1.65581600	-0.26971300
C	4.51472300	0.18031000	1.04663800
H	3.90991900	0.34556500	-2.37320300
H	5.45812300	0.47259100	-1.52614400
H	4.54371800	-1.03274100	-1.46343900
H	4.00216200	0.52270400	1.94830600
H	4.64286200	-0.90238800	1.11972000
H	5.50967700	0.63003300	1.02801900
H	-1.65124500	3.39280600	1.06385900
H	0.15483900	3.01354800	0.96051600
H	-2.66922900	1.79071300	-1.96042600
H	-4.22530000	-0.95099500	-0.56141900
H	-2.15360400	-0.88364800	-1.70901900

2b_8

C	-0.09967400	0.45938100	-0.34048500
C	-0.43789700	-0.91740300	0.28242900
C	-1.94052200	-1.21075900	0.06962100
C	-2.82685100	-0.09492400	0.65064800
C	-2.43358800	1.28473200	0.10296900
C	-0.95262400	1.52620000	0.29725900
H	-0.42890600	0.40939000	-1.38707700
C	1.37981600	0.71919000	-0.38017800
C	-0.13293500	-0.95327400	1.78661900
C	0.42595700	-1.95680900	-0.44072500
H	-2.18515900	-2.14126400	0.58815100
O	-2.28730300	-1.44316800	-1.29170800
O	-4.19709200	-0.36710300	0.44484700

H	-2.69135200	-0.06266500	1.73072700
O	-2.78492200	1.28723000	-1.28695600
H	-3.01448000	2.04929100	0.62647900
C	-0.51479600	2.55854200	1.00627800
H	-0.37393100	-1.93847300	2.19307800
H	0.92221900	-0.75836600	1.97526800
H	-0.69379900	-0.20692000	2.34747800
H	0.17462600	-2.96053000	-0.08565100
H	0.18681800	-1.92696200	-1.50525200
C	1.91813900	-1.69497600	-0.24129800
C	2.30416900	-0.23871100	-0.35641000
H	2.49142200	-2.27112200	-0.97400600
H	2.23625500	-2.07344500	0.73533700
H	1.68061700	1.75437600	-0.49332900
C	3.79314400	0.04020600	-0.42425700
C	4.48434300	-0.36941500	0.88411700
H	4.19402900	-0.60255100	-1.21768600
C	4.15282900	1.48078700	-0.77419100
H	4.08989800	0.21640800	1.71891100
H	4.33961300	-1.42595300	1.11240900
H	5.55920900	-0.18789300	0.81984200
H	5.23467800	1.57679200	-0.88383300
H	3.68904300	1.79794600	-1.71005700
H	3.83901800	2.17131900	0.01315200
H	-1.20755800	3.26948800	1.44250500
H	0.54038300	2.72647900	1.18450400
H	-2.54296100	2.13630000	-1.66391200
H	-4.30689800	-0.57521000	-0.48827800
H	-2.29530600	-0.58872000	-1.74258800

2b_9

C	-0.06591500	0.41909000	-0.28089400
C	-0.47102900	-0.85280500	0.50621600
C	-1.94619900	-1.19158000	0.19098300
C	-2.88198300	-0.01097300	0.50389300
C	-2.43113400	1.27285000	-0.20768300
C	-0.97707800	1.55914600	0.09712200
H	-0.28521500	0.20638700	-1.33574600
C	1.41067800	0.68753600	-0.20337700
C	-0.32010600	-0.65820000	2.02144900
C	0.45368400	-1.98351800	0.04070500
H	-2.24530600	-2.03538400	0.81802700
O	-2.15568600	-1.63201300	-1.14675400
O	-4.22593900	-0.32802000	0.20801700
H	-2.85390900	0.18767100	1.57429600
O	-2.63876000	1.05697200	-1.60967500
H	-3.05991400	2.10150600	0.12986500
C	-0.61192800	2.69218100	0.68293600
H	-0.61622900	-1.56978100	2.54608100
H	0.71328700	-0.43713500	2.28568300
H	-0.92347500	0.16490100	2.40194200
H	0.16636100	-2.92064200	0.52648300

H	0.31336800	-2.12609900	-1.03227800
C	1.92292000	-1.67823100	0.32754100
C	2.32148800	-0.25170500	0.03903900
H	2.55282400	-2.35704400	-0.25405600
H	2.16441900	-1.89199300	1.37539600
H	1.72921000	1.70275400	-0.41310400
C	3.81334400	0.01680800	0.05036700
C	4.19112000	1.46558100	0.34733900
H	4.23313700	-0.60549000	0.84976700
C	4.45431500	-0.43834700	-1.26778700
H	3.88403400	2.13260400	-0.46203700
H	3.72832000	1.81758000	1.27118700
H	5.27436000	1.55630000	0.45011000
H	5.54114000	-0.33991300	-1.22123200
H	4.22259100	-1.48002700	-1.49637000
H	4.09151600	0.17854300	-2.09399500
H	-1.34416200	3.45189900	0.93271000
H	0.41938500	2.89875100	0.94187200
H	-2.35421800	1.83959900	-2.08711800
H	-4.24236400	-0.68200800	-0.68668400
H	-2.11914900	-0.85548200	-1.72059300
